# Synthesis and Sar Study of Diarylpentanoid Analogues as New Anti-Inflammatory Agents

**DOI:** 10.3390/molecules191016058

**Published:** 2014-10-09

**Authors:** Sze Wei Leong, Siti Munirah Mohd Faudzi, Faridah Abas, Mohd Fadhlizil Fasihi Mohd Aluwi, Kamal Rullah, Lam Kok Wai, Mohd Nazri Abdul Bahari, Syahida Ahmad, Chau Ling Tham, Khozirah Shaari, Nordin H. Lajis

**Affiliations:** 1 Laboratory of Natural Products, Institute of Bioscience, Universiti Putra Malaysia, 43400 Serdang, Selangor, Malaysia; E-Mails: frederick_leong@hotmail.com (S.W.L.); munifaudzi@gmail.com (S.M.M.F.); 2 Department of Food Science, Faculty of Food Science and Technology, Universiti Putra Malaysia, 43400 Serdang, Selangor, Malaysia; 3 Drug and Herbal Research Centre Faculty of Pharmacy, Universiti Kebangsaan Malaysia, Jalan Raja Muda Abd. Aziz, 50300 Kuala Lumpur, Malaysia; E-Mails: fadhfasihi@yahoo.com (M.F.F.M.A.); kamalrullah@yahoo.co.id (K.R.); david_lam_98@yahoo.com (L.K.W.); 4 Department of Biochemistry, Faculty of Biotechnology and Biomolecular Sciences, Universiti Putra Malaysia, 43400 Serdang, Selangor, Malaysia; E-Mails: nazribahari89@gmail.com (M.N.A.B.); syahida@upm.edu.my (S.A.); 5 Department of Biomedical Science, Faculty of Medicine and Health Sciences, Universiti Putra Malaysia, 43400 Serdang, Selangor, Malaysia; E-Mail: chauling@upm.edu.my; 6 Department of Chemistry, Faculty of Science, Universiti Putra Malaysia, 43400 Serdang, Selangor, Malaysia; E-Mail: khozirah@upm.edu.my; 7 Al-Moalim BinLaden Chair for Scientific Miracles of Prophetic Medicine, Scientific Chairs Unit, Taibah University, P.O. Box 30001, Madinah al Munawarah 41311, Saudi Arabia

**Keywords:** anti-inflammatory, diarylpentanoid, RAW 264.7, curcumin, SAR, pharmacophore

## Abstract

A series of ninety-seven diarylpentanoid derivatives were synthesized and evaluated for their anti-inflammatory activity through NO suppression assay using interferone gamma (IFN-γ)/lipopolysaccharide (LPS)-stimulated RAW264.7 macrophages. Twelve compounds (**9**, **25**, **28**, **43**, **63**, **64**, **81**, **83**, **84**, **86**, **88** and **97**) exhibited greater or similar NO inhibitory activity in comparison with curcumin (14.7 ± 0.2 µM), notably compounds **88** and **97**, which demonstrated the most significant NO suppression activity with IC_50_ values of 4.9 ± 0.3 µM and 9.6 ± 0.5 µM, respectively. A structure–activity relationship (SAR) study revealed that the presence of a hydroxyl group in both aromatic rings is critical for bioactivity of these molecules. With the exception of the polyphenolic derivatives, low electron density in ring-A and high electron density in ring-B are important for enhancing NO inhibition. Meanwhile, pharmacophore mapping showed that hydroxyl substituents at both *meta-* and *para*-positions of ring-B could be the marker for highly active diarylpentanoid derivatives.

## 1. Introduction

**Figure 1:**
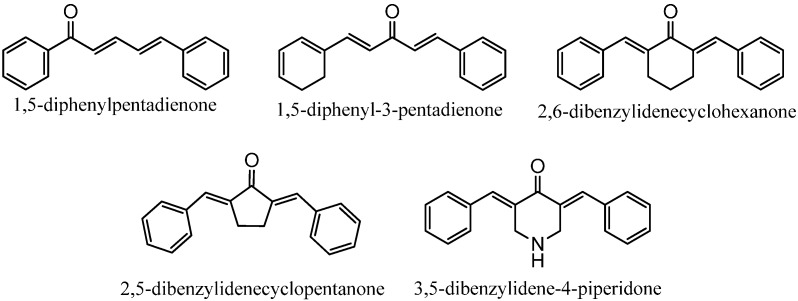
General structures of diarylpentanoid derivatives.

Diarylpentanoids may be considered as analogues of curcumin, the major constituent of *Curcuma domestica*, differing structurally by the replacement of the heptane bridge with a shorter, pentane bridge. They may also be considered as analogues of zerumbone, a natural sesquiterpenoid isolated from *Zingiber zerumbet*, based on the common feature of a dienone moiety. Naturally occurring diarylpentanoids have been reported as minor constituents from *Curcuma domestica* and found to exhibit strong antioxidant activity [1]. Diarylpentanoids and zerumbone have gained increasing attention for their excellent pharmacological activities and better bioavailability compared to curcumin. A great number of evidence has supported the significant anti-inflammatory property of diarylpentanoids and zerumbone through their remarkable inhibition of various proinflammatory cytokines and mediators such as nitric oxide (NO), tumor necrosis factor-alpha (TNF-α), interleukin-6 (IL-6), IL-10 and monocyte chemoattractant protein-1 (MCP-1) [2,3,4]. We have previously shown that 2,6-bis-(4-hydroxy-3-methoxybenzylidene)cyclohexanone (BHMC), a diarylpentanoid synthesized by our group, is a potent anti-inflammatory agent in preventing lethality of cecal ligation and puncture (CLP)-induced sepsis [5]. The compound was further shown to exhibit anti-nociceptive activity in a mouse model [6]. In addition, diarylpentanoids were also proven to have great potential as anti-cancer agents based on their excellent anti-proliferative and anti-angiogenetic properties [7,8]. Surprisingly, some of the diarylpentanoid derivatives were shown to possess significant anti-melanogenic activity on B16 melanoma cells although they do not inhibit mushroom tyrosinase, indicating them to be potential candidates for further development into skin-whitening agents in cosmetic products [2,9]. The general structures of some diarylpentanoid systems which have been studied by our group and other researches for their anti-oxidant [2,3], anti-inflammatory [2,3,7,10,11], anti-proliferation [7], and anti-tyrosinase [2] properties are presented in Figure 1.

The inflammatory process is an early immune response to threats, which constitutes processes of communication between cells/tissues/organs. It involves mediators (families of protein and lipid molecules) which helps relay new information, based on which the body’s immune system will respond and decide on how to interact with the invading pathogen or stimuli. Orchestration of immune/inflammatory responses depends upon communication by soluble molecules including inflammatory cytokines and mediators such as nitric oxide (NO) [12], interleukin (IL-6) [13], prostaglandins (PGs) [14], and tumor necrosis factor (TNF-α) [15] released by various activated phagocytes and lymphocytes such as polymorphonuclear neutrophils, mast cells, dendritic cells, macrophages, endothelial cells, hepatocytes, and natural killer (NK) cells in response to pathogenic invasion and tissue injury [16]. Appropriate levels of the released proinflammatory cytokines and inflammatory mediators are responsible for the immune system’s defense against the invading stimuli. Excessive production can cause oxidative damage of cellular components, eventually leading to healthy tissue damage [17]. Therefore, inflammation is a double-edged sword, which must be regulated at optimal levels in disease treatment and prevention.

Nitric oxide is a key molecular signaling constituent involved in the inflammatory process. It is biosynthesized endogenously from L-arginine, oxygen, and NADPH, and catalyzed by various nitric oxide synthase (NOS) enzymes. In chronic inflammation, the presence of lipopolysaccharide (LPS) or other proinflammatory cytokines activates macrophages and induces high levels of NO production through inducible-NOS (iNOS) induction. An elevated level of NO production is one of the most important factors which contributes to various chronic degenerative diseases including cancer [18], cardiovascular disorder [19], asthma [20], arthritis, neurodegenerative diseases [21], multiple sclerosis [22], ulcerative colitis, and Crohn’s disease [23]. Hence, pharmacological intervention of NO production is a promising strategy in developing potent drugs for such diseases.

**Figure 2:**
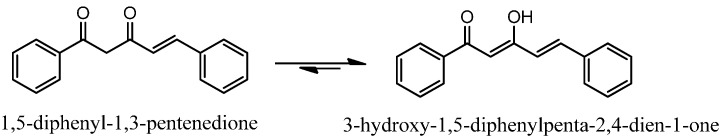
General structure of target compound.

Previously, our studies on diarylpentanoids were restricted to those with a mono carbonyl moiety. In our continuing search for new anti-inflammatory agents, we have now synthesized a series of novel diarylpentanoid derivatives in which we have preserved the potent β-diketone moiety, with the anticipation that it will provide additional hydrogen-bond donors or acceptors, leading to enhanced bioactivity. The general structure of the targeted compound is shown in Figure 2.

## 2. Results and Discussion

### 2.1. Chemistry

**Scheme 1:**
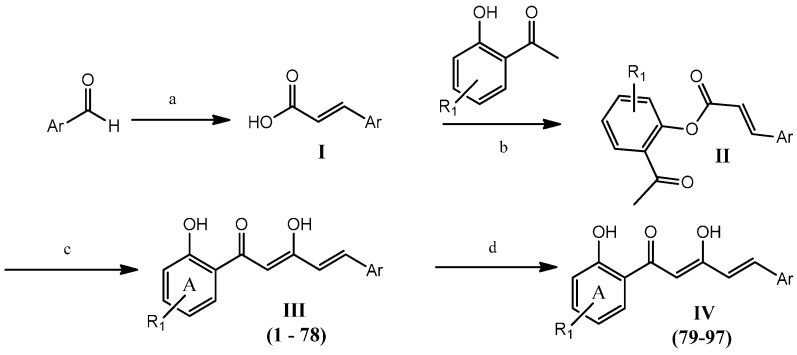
General synthetic steps for compounds **1**–**97**^1^.

Notes: ^1^ *Reagents and conditions*: (a) malonic acid, pyridine, reflux (4 h); (b) POCl_3_, pyridine, RT (overnight); (c) KOH, pyridine, RT (overnight); (d) BBr_3_, CH_2_Cl_2_, 0 °C (8 h).

Synthesis of ninety-seven (**1**–**97**) analogues of 1,5-diphenyl-1,3-pentenedione was carried out through a series of reactions which included Knoevenagel reaction, phenolic esterification, Baker-Venkataraman rearrangements, and demethylation (Scheme 1). Eighty-seven of the synthesized compounds are new. The new and known compounds are differentiated by the presence of a reference melting point as presented in Table S1 in Supplementary Data. The respective commercially available aromatic aldehydes were reacted with malonic acid in the presence of a catalytic amount of piperidine in pyridine, under reflux to afford the respective acrylic acids **I** [24]. Aqueous workup of the crude products afforded the desired compounds without any further purification. All benzaldehydes and naphthaldehydes were successfully converted into the respective acrylic acids with greater than 85% conversion. The presence of an electron withdrawing group in the aromatic rings improved the yield by up to 96%, regardless of their substitution pattern. However, the product yields for the five-membered heterocyclic aldehydes were found to be relatively low (33%–65%) compared with the benzaldehydes and naphthaldehydes. This may be due to the electron-rich character of heterocyclic rings.

The resulting acrylic acid intermediates were then reacted with the selected 2'-hydroxyacetophenones at room temperature to provide the phenolic esters (**II)**, by employing phosphoryl chloride as *in situ* chlorinating reagent of the cinnamic acid [25]. Pyridine was chosen as the reaction medium due to its ability to remove the hydrochloric acid produced from the reaction. The desired phenolic esters were obtained in high yields (>90%) regardless of their ring substituents.

The phenolic ester intermediates were subjected to Baker-Venkataraman rearrangements by stirring with potassium hydroxide in pyridine at ambient temperature to produce the desired respective Products **III** (**1**–**78**). The product yields obtained were in the range of 11% to 82%. In general, higher yields were obtained when an electron-deficient aromatic ring was present. Surprisingly, intermediates with a thiofuran moiety, an electron-rich heterocyclic ring, gave better yields (greater than 70%) of the expected products.

Methoxy-containing diarylpentanoids were further demethylated using boron tribromide in dichloromethane at 0 °C to produce polyhydroxylated diarylpentenedione Analogues **IV** (**79**–**97**) [26]. The yields obtained (15% to 46%) were inversely proportional to the number of methoxy groups present. All the purified diarylpentenediones were characterized by ^1^H-NMR, ^13^C-NMR, (Figure S1 in Supplementary Data) and mass spectrometry. The spectrometric data is presented in the Table S1 in Supplementary Data. The ^1^H-NMR spectra of all diarylpentenediones exhibited an intense and sharp singlet at 14–15 ppm, indicative of the chelated hydroxyl groups. It is thus concluded that these compounds are more stable in the keto-enol rather than in their diketo forms. In addition, the large coupling constant (*J*) values of the double bond signals (15–16 Hz) indicated that all the compounds existed as the *trans* isomer. All purified compounds used for bioassay were of 95% to 99% purity based on their respective high performance liquid chromatography (HPLC) profiles.

### 2.2. NO Suppression in IFN-γ/LPS-Stimulated Macrophages

The synthesized compounds were screened for NO suppression activity in IFN-γ/LPS-stimulated RAW 264.7 macrophages at a 50 µM test concentration. Fifty-seven compounds were found to significantly inhibit NO production, suggesting that the diarylpentenedione system possesses anti-inflammatory properties and is an interesting candidate for further investigations. The IC_50_ values of the fifty-seven bioactive compounds were determined and compared to that of the positive control, curcumin. An MTT assay was also carried out to confirm that NO inhibition was not due to cytotoxicity. Compounds exhibiting significant bioactivities are listed in Table 1, Table 2, Table 3 and Table 4, grouped according to their functional or structural features. The complete bioassay results of all the compounds are provided as Table S2 in Supplementary Data.

**Table 1:** Nitric oxide (NO) suppression activity and cytotoxicity of active compounds in diarylpentenedione series on RAW 264.7 cells. 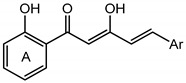

Compounds	Ar (Ring B)	NO Inhibition at 50 µM (%) ± S.E.M	NO Inhibition IC_50_ (µM) ± S.E.M	Cytotoxicity IC_50_ (µM) ± S.E.M
Curcumin	-	99.3 ± 0.2	14.7 ± 0.2	28.8 ± 0.8
**1**	phenyl	94.7 ± 1.2	22.6 ± 0.5	56.2 ± 1.1
**2**	2-chlorophenyl	91.0 ± 2.9	27.3 ± 0.2	>100
**3**	3-chlorophenyl	93.0 ± 3.4	26.7 ± 0.7	>100
**4**	3-bromophenyl	88.4 ± 3.2	29.4 ± 0.7	>100
**5**	2-methoxyphenyl	91.8 ± 1.5	25.7 ± 0.6	>100
**6**	3-methoxyphenyl	81.6 ± 4.0	31.6 ± 0.6	>100
**8**	3,4-dimethoxyphenyl	94.7 ± 1.7	24.6 ± 0.7	>100
**9**	3,4,5-trimethoxyphenyl	96.6 ± 1.4	16.6 ± 1.1	>100
**12**	furan-2-yl	82.0 ± 3.2	49.0 ± 1.0	>100
**14**	2,5-dimethoxyphenyl	92.9 ± 0.2	28.4 ± 0.4	>100
**16**	thiophen-2-yl	79.1 ± 4.6	42.6 ± 0.1	>100
**17**	5-chlorothiophen-2-yl	63.8 ± 2.6	36.4 ± 1.8	67.9 ± 2.9
**18**	5-methylthiophen-2-yl	58.9 ± 5.8	39.6 ± 4.1	>100
**19**	naphthalen-1-yl	85.8 ± 3.9	23.0 ± 1.7	>100

**Table 2:** NO suppression activity and cytotoxicity of active compounds in halogenated diarylpentenedione series on RAW 264.7 cells. 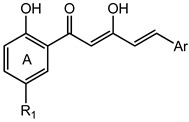

Compounds	R_1_ (Ring A)	Ar (Ring B)	NO inhibition at 50 µM (%) ± S.E.M	NO inhibition IC_50_ (µM) ± S.E.M	Cytotoxicity IC_50_ (µM) ± S.E.M
**21**	Cl	Phenyl	85.3 ± 6.8	47.6 ± 1.1	>100
**22**	Cl	2-chlorophenyl	82.5 ± 4.3	30.0 ± 2.1	>100
**23**	Cl	3-chlorophenyl	66.6 ± 4.1	36.2 ± 0.9	68.4 ± 2.2
**24**	Cl	2-methoxyphenyl	70.4 ± 1.3	36.0 ± 0.5	>100
**25**	Cl	3-methoxyphenyl	76.3 ± 6.5	17.4 ± 0.4	>100
**28**	Cl	3,4,5-trimethoxyphenyl	89.5 ± 5.9	13.6 ± 0.5	>100
**35**	Cl	thiophen-2-yl	68.8 ± 4.2	32.2 ± 0.2	>100
**36**	Cl	5-chlorothiophen-2-yl	71.6 ± 3.2	25.3 ± 1.7	>100
**37**	Cl	5-methylthiophen-2-yl	56.0 ± 2.6	62.4 ± 2.3	>100
**38**	Cl	5-methylfuran-2-yl	73.8 ± 1.1	25.5 ± 1.0	>100
**41**	Br	Phenyl	79.9 ± 8.7	20.8 ± 0.5	>100
**43**	Br	3-chlorophenyl	81.2 ± 5.2	19.8 ± 0.9	>100

**Table 3:** NO suppression activity and cytotoxicity of active compounds in methoxylated diarylpentenedione series on RAW 264.7 cells. 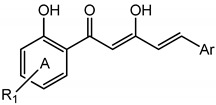

Compounds	R1 (Ring A)	Ar (Ring B)	NO inhibition at 50 µM (%) ± S.E.M	NO inhibition IC_50_ (µM) ± S.E.M	Cytotoxicity IC_50_ (µM) ± S.E.M
**45**	4-OMe	phenyl	87.9 ± 5.1	29.5 ± 0.5	>100
**46**	4-OMe	2-chlorophenyl	76.2 ± 4.6	58.5 ± 2.5	>100
**48**	4-OMe	2-methoxyphenyl	66.3 ± 6.8	39.0 ± 1.2	88.6 ± 2.5
**49**	4-OMe	3-methoxyphenyl	57.5 ± 6.5	45.8 ± 0.8	>100
**51**	4-OMe	3,4-dimethoxyphenyl	58.9 ± 2.8	21.7 ± 0.5	>100
**52**	4-OMe	3,4,5-trimethoxyphenyl	96.4 ± 0.4	29.3 ± 0.3	>100
**58**	5-OMe	phenyl	92.8 ± 1.8	25.9 ± 0.3	>100
**59**	5-OMe	3-chlorophenyl	89.1 ± 3.6	35.7 ± 0.8	>100
**61**	5-OMe	2-methoxyphenyl	65.8 ± 2.4	27.4 ± 0.2	>100
**62**	5-OMe	3-methoxyphenyl	92.6 ± 1.2	35.1 ± 0.2	>100
**63**	5-OMe	3,4-dimethoxyphenyl	92.2 ± 0.8	19.8 ± 0.4	>100
**64**	5-OMe	3,4,5-trimethoxyphenyl	95.2 ± 1.1	18.4 ± 0.2	>100
**67**	5-OMe	furan-2-yl	72.1 ± 3.1	71.5 ± 2.5	>100
**68**	5-OMe	2,4-dimethoxyphenyl	79.5 ± 4.7	33.4 ± 0.7	>100
**71**	5-OMe	thiophen-2-yl	85.0 ± 2.7	35.6 ± 0.6	>100

**Table 4:** NO suppression activity and cytotoxicity of active polyphenolic diarylpentenedione analogues on RAW 264.7 cells. 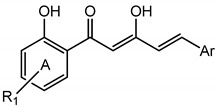

Compounds	R1 (Ring A)	Ar (Ring B)	NO inhibition at 50 µM (%) ± S.E.M	NO inhibition IC50 (µM) ± S.E.M	Cytotoxicity IC_50_ (µM) ± S.E.M
**79**	H	2-hydroxyphenyl	95.5 ± 0.6	28.9 ± 1.5	56.4 ± 1.2
**80**	H	3-hydroxyphenyl	97.2 ± 0.7	30.8 ± 0.7	67.3 ± 0.6
**81**	H	4-hydroxyphenyl	91.5 ± 3.9	19.1 ± 0.6	>100
**83**	H	2,5-dihydroxyphenyl	94.3 ± 1.8	16.7 ± 0.6	>100
**84**	5-Cl	2-hydroxyphenyl	95.2 ± 0.9	15.9 ± 0.9	53.1 ± 1.0
**85**	5-Cl	3-hydroxyphenyl	95.9 ± 0.5	26.9 ± 0.8	89.1 ± 1.1
**86**	5-Cl	4-hydroxyphenyl	90.3 ± 2.7	18.7 ± 0.8	66.0 ± 1.2
**87**	5-Cl	2,5-dihydroxyphenyl	96.5 ± 1.4	32.3 ± 0.9	49.5 ± 1.7
**88**	5-Cl	3,4-dihydroxyphenyl	99.7 ± 1.2	4.9 ± 0.3	40.9 ± 1.6
**89**	5-OH	2-hydroxyphenyl	83.6 ± 2.0	31.0 ± 2.0	72.6 ± 2.6
**90**	5-OH	3-hydroxyphenyl	90.9 ± 2.0	35.7 ± 0. 9	85.0 ± 1.4
**91**	5-OH	furan-2-yl	83.6 ± 2.8	75.0 ± 4.9	94.1 ±0.9
**93**	4-OH	phenyl	94.7 ± 2.3	29.9 ± 0.4	59.5 ± 0.5
**94**	4-OH	3-hydroxyphenyl	95.4 ± 1.5	24.8 ± 0.4	70.1 ± 1.8
**95**	4-OH	thiophen-2-yl	60.4 ± 2.0	44.8 ± 1.6	>100
**97**	H	3,4-dihydroxyphenyl	98.9 ± 1.6	9.6 ± 0.5	>100

Two compounds (**88** and **97**) were found to exhibit the most potent anti-inflammatory activity, giving IC_50_ values of 4.9 μM and 9.6 μM, respectively. Meanwhile ten other compounds (**9**, **25**, **28**, **43**, **63**, **64**, **81**, **83**, **84**, and **86**) exhibited comparable NO inhibitory activity to curcumin, with IC_50_ values of less than 20 µM. Based on these results, it appears that polyphenolic and poly(methoxyphenyl) moieties are important for NO suppression activity. The presence of a catechol moiety, as represented by compounds **88** and **97**, appeared to be an important contributing factor since it resulted in a two- to four-fold improvement in bioactivity.

This observation is consistent with previous reports in which it was shown that the presence of a catechol moiety in flavonoids [27], curcuminoids [28], and aurones [29] enhanced the NO inhibition activity significantly. Furthermore, it has also been reported that catechol moiety containing compounds are frequently bioactive against various inflammatory enzymes, such as COX-2 and iNOS, through inhibition of NF-κB activation [30]. Notably, the unexpected synergistic effect of catechol and α,β-unsaturated carbonyl moieties on NF-κB inactivation was presented by Chiang and co-workers [31]. On this account, compounds **88** and **97**, the catechol and α,β-unsaturated carbonyl moieties containing diarylpentenediones, may represent new candidates for further investigation of their effects towards inflammatory mediators including the NF-κB (LPS-induced pathway) and Jak-STAT (IFN-γ-induced pathway) inactivation analysis and direct modulation of iNOS.

Comparisons between compounds within the same and between different groups were conducted to identify correlations between the structural features with NO suppression activity. For the diarylpentenedione series (Table 1), the trimethoxylated compound **9**, exhibited highest NO suppression which suggests that high electron density in ring-B is an important factor in improving NO inhibition. A similar trend was also observed for the halogenated and methoxylated diarylpentenedione series listed in Table 2 and Table 3, respectively. In the respective groups, the trimethoxylated compounds **28** and **64** exhibited the highest NO suppression. In contrast, analogues with low electron density ring-B, as in the dihalogenated diaryl analogues (**15**, **37** and **70**), further supported our conclusion.

Further structure-activity comparison between all the series of analogues showed that low electron density of ring-A was important in enhancing the NO inhibition activity. This was clearly demonstrated by compounds **6**, **25**, **49**, and **62**, in which the presence of an electron donating group (methoxy) in ring-A was accompanied by reduced NO suppression, while the presence of an electron withdrawing group (chloro) enhanced the bioactivity. The same conclusion could also be drawn based on the bioactivity displayed by the Analogues **9**, **28**, **52** and **64**, where the diarylpentenedione analogue with halogenated ring-A (**28**) exhibited the highest activity. In contrast, the methoxylated compounds **49** and **52** exhibited the weakest activity. Further comparison between the Analogues **23**, **25**, **59** and **62** further supported the electron density-related effect.

Demethylation of methoxylated diarylpentenediones to form the hydroxylated analogues increased the bioactivity significantly. Comparison of compounds **7** (See Table 2 in Supplementary Data), **14** and **27** with their respective demethylated analogues (compounds **81**, **83** and **88**) showed that NO inhibition improved very significantly consistent with the decrease in the electron density of ring-B. This could be rationalized by the higher affinity for hydrogen bonding by the hydroxyl groups as compared to methoxyl. This observation suggested that hydrogen bonding is an additional contributing factor for the bioactivity enhancement along with electron density. Thus, the polyphenolic analogues, with their higher hydrogen bonding capacity, are the more potent group in this class of compounds.

Apart from this, it has also been found that the substitution position of functionalities at ring-B plays a pivotal role in influencing the NO inhibitory activity. The comparison of compounds **79**, **80**, **81**, **82**, **83** and **97** shows that the substitution of hydroxyl groups at both *meta*- and *para*-positions is essential for bioactive molecules as compound **97**, a *meta*- and *para*-hydroxylated analogue has displayed much better activity than any other mono- or di-hydroxylated derivative. The suggested trend was further supported by similar observations made in the comparisons of compounds **84**, **85**, **86**, **87** and **88** of which compound **88** with di-substitution at both *meta*- and *para*-positions possessed the highest activity. Interestingly, the same conclusion can also been drawn from the comparisons of poly(methoxyphenyl) containing analogues. Compounds with a *meta*- and *para*-dimethoxylated phenyl ring (**8**, **9**, **28**, **51**, **52**, **63** and **64**) exhibited significantly better activity than those with other substitution patterns. Thus, it is confirmed that the substitution at both *meta*- and *para*-positions contributes to NO inhibitory activity. In contrast, replacing the aryl ring-B with heterocyclic aromatic rings such as thiophene and furan (see compounds **16** and **31**) was found to be undesirable. Although higher in electron density, it dramatically decreased NO suppression activity.

### 2.3. Quantitative Structure Activity Relationship (QSAR) Analysis

Quantitative structure activity relationship (QSAR) analysis is a statistical approach commonly used to explore, explain, and rationalize the significant correlation between the experimental biological activity or chemical reactivity of a series of drugs with their molecular geometry and physicochemical properties. Currently, there are six types of QSAR models including 1D, 2D, 3D, 4D, 5D, and 6D QSAR with 2D and 3D QSAR being the most commonly used models. In the present study, 2D and 3D QSAR analyses were employed to understand the physicochemical properties and molecular descriptors or features, which could contribute to the NO suppression activity.

#### 2.3.1. 2D-QSAR

Genetic function approximation (GFA) analysis is one of the most common algorithms used in QSAR models. Sivakumar and co-workers reported that GFA analysis had been successfully applied to study the anti-tuberculosis properties of selected chalcones and flavonoids with excellent predictive models [32]. The r^2^ (conventional correlation coefficient) and q^2^ (cross-validation correlation coefficient) values achieved by the group were between 0.85 to 0.97 and, between 0.79 to 0.94, respectively. These values imply that GFA analysis is very accurate in predicting the bioactivity of small molecules. Thus, in this study, GFA was selected as the analytical method to establish a 2D QSAR model for the anti-inflammatory activity of diarylpentenedione system as a family of small molecules.

GFA analysis was carried out by using Discovery Studio 3.1 based on thermodynamic, constitutional, and topological descriptors including ALogP (lipophilicity), EPFP_6 (fingerprint), number of hydrogen donors and molecular fractional polar surface area (MFPSA) where pIC_50_ (−log IC_50_) is the dependent variable. GFA equation represented the best equation generated by GFA analysis and it was further evaluated with randomization test to ensure its reliability as presented in Table 5.

GFA equation:

pIC_50_ = 4.2787 − 0.12059 * Count (EPFP_6: −66437561) + 0.27918 * Count (EPFP_6: −1317016190) + 0.29619 * Num_H_Donors − 1.3738 * Molecular_Fractional Polar Surface Area − 0.67967 (3.324 − Alog P) (1)

Parameter values generated: n = 57; r^2^ = 0.8639; r^2^ (adj) = 0.8432; r^2^ (pred) = 0.8152; q^2^ = 0.558; RMS Residual Error = 0.07841; Friedman L.O.F. = 0.01223 where: n = number of compounds involve in analysis; r^2^ = coefficient of determination; r^2^ (adj) = r^2^ adjusted = the number of terms in the model; r^2^ (pred) = prediction (PRESS) r^2^, and equivalent to q^2^ from a leave-1-out cross-validation; q^2^ = cross-validation correlation coefficient; RMS = root mean square residual error; Friedman L.O.F. = Friedman lack-of-fit score.

**Table 5:** Randomization test of genetic function approximation (GFA) equation.

Eq. No.	(1)		
*r^2^* from nonrandom model	0.8639		
Confidence level	90%	95%	98%
Total trials	9	19	49
Nonrandom r^2^ < random r^2^	0	0	0
Mean value of *r^2^* from random trials	0.105	0.129	0.138
Standard deviation	±0.070	±0.121	±0.131

Analysis based on this equation found the r^2^ value to be 0.8639 with low Friedman L.O.F value of 0.01223, thus indicating that the model is acceptable. The Friedman L.O.F value is a measurement that estimates the most appropriate number of features, resists overfitting, and allows control over the smoothness of fit. The lower the value of Friedman L.O.F, the less likely it is that the GFA model is overfitting the data, thus the more reliable is the model. Further randomization test at 90%, 95%, and 98% confidence level were performed to confirm the equation’s reliability. The randomization was done by repeatedly permuting the activity values of the training set to obtain subsequent GFA scores. If the score of the original GFA model is better than those from the permuted data sets, the model is considered statistically significant. All equations using the scrambled data notably displayed lower r^2^ values (Table 5) compared to the non-random model, which increased the significance and confidence in the training compounds and suggested that the descriptors used were highly selective.

**Figure 3:**
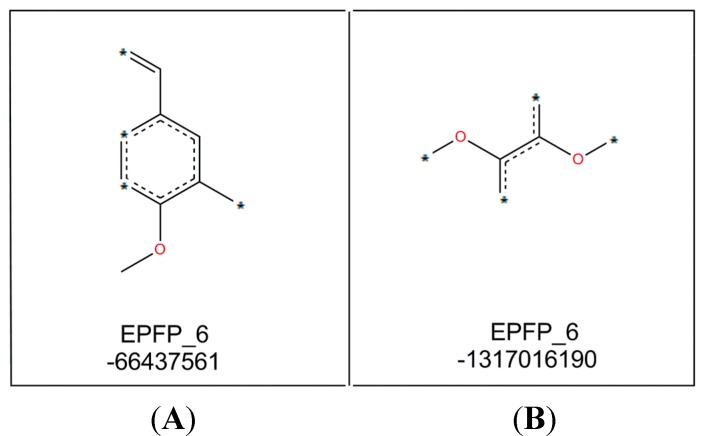
Molecular fragments generated by GFA analysis.

As stated in the GFA equation, two important molecular fragments (as shown in Figure 3) were determined by this model. Molecular fragment B (EPFP_6: −1317016190) is important for activity of compounds while molecular fragment A (EPFP_6: −66437561) tend to reduce it.

The positive effects of fragment B may be explained by comparing the structures and bioactivities of compounds **7**–**9**, which indicated that a higher number of methoxyl groups in the ring increases the NO suppression activity of the diarylpentenedione system. This trend is also observed for the Analogues **62**, **63** and **64** where bioactivity for the 3,4,5-trimethoxylated compound **64** was the most active. The significant role of fragment B was also revealed when by the bioactivities of Analogues **83** and **87** were compared to those of **88** and **97**. The *ortho-*dihydroxylated compounds **88** and **97** were seven- and two-fold better inhibitors of NO production than their para-hydroxylated counterparts **87** and **83**, respectively, which indicated that the presence of the group was much more important than non-adjacent dihydroxylated analogues.

The decrease in NO inhibition due to the presence of molecular fragment A may be represented by the analogues containing a single methoxyl group, especially that with *p*-methoxylated phenyl ring. This trend is clearly demonstrated by compounds **5**–**7**, of which compound **7** with *p*-methoxy group in ring-B exhibited the weakest activity. A similar trend is also detected in compounds **45**–**56**, where the NO inhibition of these *p-*methoxylated ring-A containing analogues are generally weaker.

The number of H-donors present in the molecule is also an important factor influencing the activity. GFA analysis suggested that a higher number of H-donors present in the diarylpentenedione system increases the bioactivity of the compounds. This correlation was clearly demonstrated by comparing the bioactivities of Analogues **21**, **86** and **88**, which showed that the increase in the number of hydrogen donors led to higher NO inhibition. The same trend was also shown by the analogue Series **1**, **80** and **97**.

Apart from number of H-donors, AlogP and MFPSA are two other important factors influencing bioactivity of the analogues. AlogP represents lipophilicity of the respective compound while MFPSA represents the fraction of polar surface to total areas. Based on the GFA equation (1), higher lipophilicity and lower MFPSA are preferable for potency of the compounds. Thus, the analogues containing a heterocyclic aromatic ring were found to gradually decrease in bioactivity due to their lower lipophilicity and higher MFPSA characteristics. All of the calculated parameters are listed in Table 6.

**Table 6:** IC_50_, pIC_50_, ALogP, number of hydrogen donors and molecular fractional polar surface area (MFPSA) values of selected active compounds.

Compounds	IC_50_		pIC_50_		ALogP		Number of Hydrogen Donors		MFPSA	
**1**	22.6 ± 0.5		4.65		3.4		2		0.2	
**2**	27.3 ± 0.2		4.56		4.1		2		0.2	
**3**	26.7 ± 0.7		4.57		4.1		2		0.2	
**4**	29.4 ± 0.7		4.53		4.1		2		0.2	
**5**	25.7 ± 0.6		4.59		3.4		2		0.2	
**6**	31.6 ± 0.6		4.5		3.4		2		0.2	
**8**	24.6 ± 0.7		4.61		3.4		2		0.2	
**9**	16.6 ± 1.1		4.78		3.3		2		0.2	
**12**	49.0 ± 1.0		4.31		2.8		2		0.3	
**14**	28.4 ± 0.4		4.55		3.4		2		0.2	
**16**	42.6 ± 0.1		4.37		3.3		2		0.3	
**17**	36.4 ± 1.8		4.44		3.8		2		0.3	
**18**	39.6 ± 4.1		4.4		3.5		2		0.3	
**19**	23.0 ± 1.7		4.64		4.3		2		0.2	
**21**	47.6 ± 1.1		4.32		4.1		2		0.2	
**22**	30.0 ± 2.1		4.52		4.7		2		0.2	
**23**	36.2 ± 0.9		4.44		4.7		2		0.2	
**24**	36.0 ± 0.5		4.44		4.0		2		0.2	
**25**	17.4 ± 0.4		4.76		4.0		2		0.2	
**28**	13.6 ± 0.5		4.87		4.0		2		0.2	
**35**	32.2 ± 0.2		4.49		4.0		2		0.3	
**36**	25.3 ± 1.7		4.6		4.5		2		0.3	
**37**	62.4 ± 2.3		4.2		4.2		2		0.3	
**38**	25.5 ± 1.0		4.59		3.6		2		0.2	
**41**	20.8 ± 0.5		4.68		4.1		2		0.2	
**43**	19.8 ± 0.9		4.7		4.8		2		0.2	
**45**	29.5 ± 0.5		4.53		3.4		2		0.2	
**46**	58.5 ± 2.4		4.23		4.0		2		0.2	
**48**	39.0 ± 1.2		4.41		3.4		2		0.2	
**49**	45.8 ± 0.8		4.34		3.4		2		0.2	
**51**	21.7 ± 0.5		4.66		3.3		2		0.2	
**52**	29.3 ± 0.3		4.53		3.3		2		0.2	
**58**	25.9 ± 0.3		4.59		3.4		2		0.2	
**59**	35.7 ± 0.8		4.45		4.0		2		0.2	
**61**	27.4 ± 0.2		4.56		3.4		2		0.2	
**62**	35.1 ± 0.2		4.46		3.4		2		0.2	
**63**	19.8 ± 0.4		4.7		3.3		2		0.2	
**64**	18.4 ± 0.2		4.74		3.3		2		0.2	
**67**	71.5 ± 2.5		4.15		2.8		2		0.3	
**68**	33.4 ± 0.7		4.48		3.3		2		0.2	
**71**	35.6 ± 0.6		4.45		3.3		2		0.3	
**79**	28.9 ± 1.5		4.54		3.1		3		0.3	
**80**	30.8 ± 0.7		4.51		3.1		3		0.3	
**81**	19.1 ± 0.6		4.72		3.1		3		0.3	
**83**	16.7 ± 0.6		4.78		2.9		4		0.3	
**84**	15.9 ± 0.9		4.8		3.8		3		0.3	
**85**	26.9 ± 0.8		4.57		3.8		3		0.3	
**86**	18.7 ± 0.8		4.73		3.8		3		0.3	
**87**	32.3 ± 0.9		4.49		3.6		4		0.3	
**88**	4.9 ± 0.3		5.31		3.6		4		0.3	
**89**	35.7 ± 0.9		4.51		2.9		4		0.3	
**90**	31.0 ± 2.0		4.45		2.9		4		0.3	
**91**	75.0 ± 4.9		4.13		2.5		3		0.3	
**93**	29.9 ± 0.4		4.52		3.1		3		0.3	
**94**	24.8 ± 0.4		4.61		2.9		4		0.3	
**95**	44.8 ± 1.6		4.35		3.1		3		0.4	
**97**	9.6 ± 0.5		5.02		2.9		4		0.3	

#### 2.3.2. 3D-QSAR

**Figure 4:**
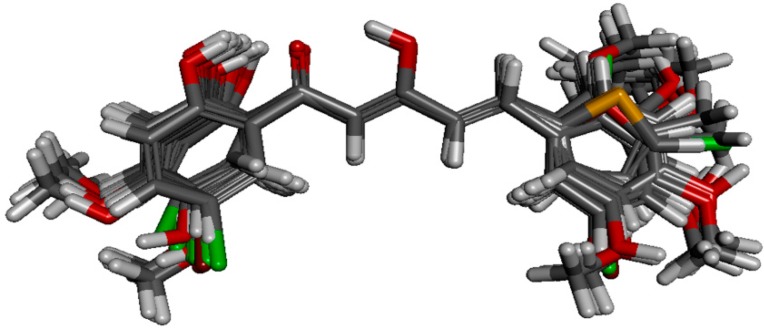
Structural alignment of the derivatives by template-based method according to the core of compound **88**.

Comparative molecular field analysis (CoMFA) was employed to investigate the correlation between NO suppression activity of the diarylpentenedione analogues to their 3D structures, as well as to their electrostatic and steric grid map. Based on their pIC_50_ values, fifty-seven of the analogues were selected as model data set. Structural alignment is critical in determining the accuracy of results and reliability in CoMFA analysis so that the best compound could be selected as template molecule. Compound **88** was chosen due to its highest potency in inhibiting NO production. The alignment of the selected analogues at the common α,β-unsaturated β-diketone fragment with compound **88** is shown in Figure 4.

**Figure 5:**
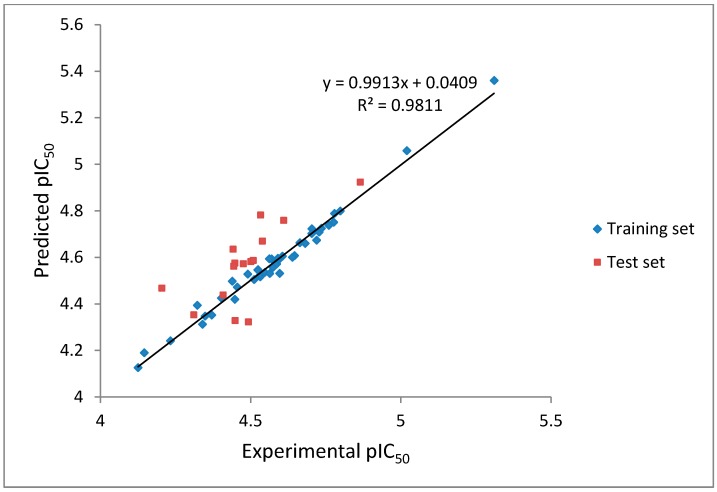
The experimental pIC_50_ *versus* predicted activity plot of training set and test set compounds.

**Figure 6:**
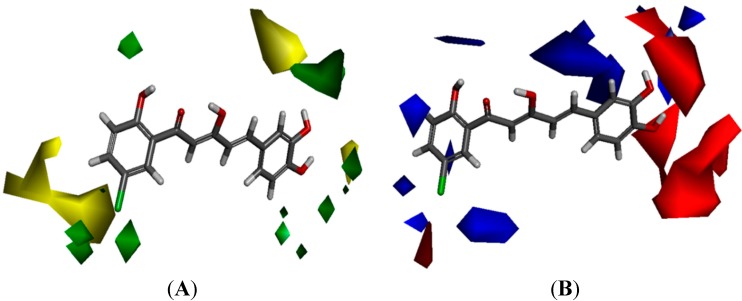
(**A**) Van der Waals contour maps generated by 3D-Quantitative structure activity relationship (QSAR) modeling. Green contours indicate the regions where bulky groups are favorable in enhancing activity, whereas yellow contours indicate regions where bulky groups are disfavorable and reduce activity. (**B**) Electrostatic contour maps generated by 3D-QSAR modeling. Blue contours indicate regions where electronegative groups are favorable in increasing activity, whereas red contours indicate regions where electropositive groups are favorable in improving activity. The most potent candidate, compound **88** was chosen as reference molecule.

CoMFA analysis is considered valid if and only if the r^2^ and q^2^ values are greater than 0.8 and 0.5, respectively. The r^2^ and q^2^ values of our CoMFA model were found to be 0.981 and 0.562, respectively, which implied that the generated correlations were acceptable. A plot depicting the experimental *versus* predicted pIC_50_ values for training and test set compounds are shown in Figure 5. The CoMFA contour maps generated were presented together with the most potent compound (**88**) in Figure 6A,B representing the van der Waals and electrostatic contour maps, respectively.

The yellow regions in Figure 6A indicate where a sterically bulkier moiety is unfavorable while the green regions indicate where a sterically bulkier moiety is preferable in enhancing the bioactivity. For the electrostatic contour maps, the red regions represent the space where a hydrogen acceptor is preferable, while the blue regions represent the space where a hydrogen donor is preferable in improving potency.

Based on the contour map in Figure 6A, bulkier groups were more preferred at the C-2' and C-5' position of ring-A while they were undesirable at the *para* position of ring-A. This explaines why compound **88**, possessing these features, is a potent NO inhibitor while the *m*-methoxylated ring-A analogues are relatively poor NO inhibitors. These 3D-QSAR predictions further support the conclusion that the presence of *p*-methoxylation of the phenyl ring will lead to a reduction of potency. On the other hand, bulkier moieties are more favorable at the *meta-* and *para*-positions of ring-B which explaines the higher bioactivity exhibited by compound **9** in comparison with compounds **7** and **8**. Interestingly, the yellow regions are also observed at the *meta-* and *para*-positions of ring-B, which seems to contradict our real experimental results where demethylation of the methoxyl groups leads to enhancement of potency (**84** and **86** *vs.* **24** and **26**). As we have mentioned in the earlier section, this phenomenon could be rationalized by the increase in hydrogen bonding capacity after converting the methoxyl groups to hydroxyl. A hydroxyl group can act as both hydrogen bond donor and acceptor while a methoxyl group can only act as hydrogen bond acceptor.

The electrostatic contour maps (Figure 6B) shows that H-donors are preferable on both the phenyl rings. Therefore, presence of multiple hydrogen donors is important in preparing more bioactive molecules. This observation is in agreement with the 2D-QSAR results, which indicates that the number of hydrogen donors is directly proportional to the potency of compounds. Comparison of the bioactivities of compounds **21**, **86** and **88** revealed the importance of H-donor in bioactivity enhancement. Conversely, multiple hydrogen acceptors are only preferable at *meta-* and *para*-positions of ring-B. Thus, analogues with *o*,*m*-methoxylatedor, *o*,*para*-methoxylated ring-B (**68** and **69**) gave low NO inhibition, while those with *m*, *p*-methoxylated ring-B (**63** and **64**) showed stronger NO inhibition.

### 2.4. Pharmacophore Mapping

Pharmacophore mapping is an abstract concept to illustrate common interaction patterns of a series of active ligands with a biological receptor. It can also be defined as the common features which are responsible for pharmacological activities or chemical reactivity, possessed by a series of bioactive compounds [33]. Pharmacophore mapping has been widely used in drug discovery programs due to its ability to provide important information in designing highly active lead compounds. Recent studies have integrated pharmacophore structures with molecular docking, which further strengthen the hypothesis on the pharmacophore [34,35]. Another study by Dong and coworkers has proven the rationale and feasibility of pharmacophore utilization in drug development [36].

**Figure 7:**
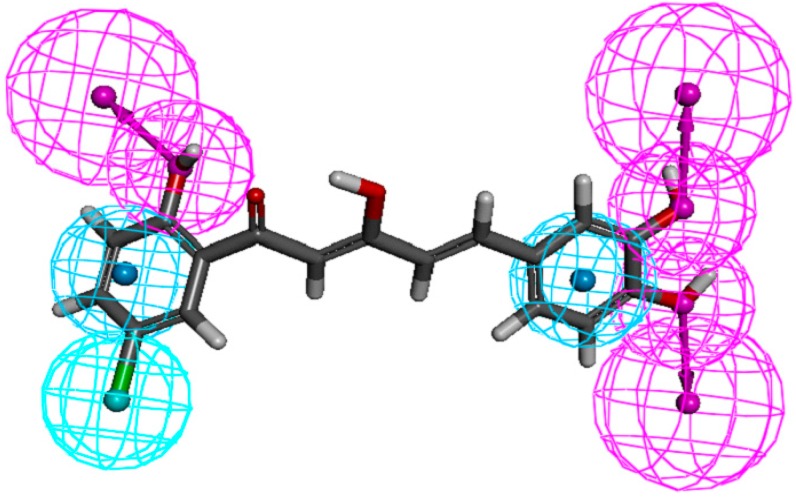
Pharmacophore mapping of compound **88**. Features are color-coded as follows: hydrophobic aromatic (HA), blue; hydrophobic (HYP), cyan; hydrogen-bond donor (HBD), pink.

In the present study, pharmacophore mapping was performed using Discovery Studio 3.1, based on selected chemical features, such as hydrophobicity (HYP), hydrophobic aromatic ring (HA), hydrogen bond acceptor (HBA), and hydrogen bond donor (HBD), based on NO suppression activities and common features of ten selected analogues (**9**, **12**, **28**, **37**, **46**, **67**, **84**, **88**, **91** and **97**). The pharmacophore map generated from this exercise is shown in Figure 7, where cyan, blue, and red represent aromatic hydrophobic, hydrophobic, and hydrogen-bond donor regions, respectively. As shown in Figure 7, important features of the diarylpentanoids, which were responsible for bioactivity, included the hydrogen-bond donor at *meta-* and *para*-positions of ring-B and *ortho* position of ring-A, the hydrophobic region at 5'-position of ring-A and the aromatic hydrophobic regions on both phenyl rings.

### 2.5. ADMET Analysis

ADMET analysis refers to the absorption, distribution, metabolism, excretion, and toxicity prediction of a molecule within an organism based on its molecular structure. It is one of the crucial steps in computer-aided drug design (CADD) due to its ability to filter out low bioavailability and toxic candidates, thus improving the efficiency and reduce the cost of research and development.

ADMET analysis was performed using Discovery Studio 3.1, based on aqueous solubility (AS), human intestinal absorption (HIA), blood brain barrier (BBB), cytochrome P450 2D6 (CYP2D6), plasma protein binding (PPB), and hepatotoxicity (HT) descriptors of ten selected compounds. A summary of the results from this experiment is presented in Table 7.

The poor bioavailability of oral drugs is related to their low solubility and low permeability. Therefore, sufficient levels of aqueous solubility and human intestinal absorption are important in improving drug delivery in the human body. From the data presented in this analysis, the Analogues **9**, **64**, **81**, **83**, **84**, **86**, **88** and **97** showed good aqueous solubility and intestinal absorption, indicating that they could be good candidates for oral drugs. Meanwhile, despite offering good human intestinal absorption property, the Analogues **25** and **28** have low aqueous solubility.

**Table 7:** Results of absorption, distribution, metabolism, excretion, and toxicity (ADMET) predictions on six important parameters.

Compounds	AS	HIA	BBB	CYP2D6	PPB	HT
**9**	Good	Good	Medium	Non-inhibit	Bound	Hepatotoxin
**25**	Low	Good	High	Non-inhibit	Bound	Hepatotoxin
**28**	Low	Good	Medium	Non-inhibit	Bound	Hepatotoxin
**64**	Good	Good	Low	Non-inhibit	Bound	Hepatotoxin
**81**	Good	Good	Low	Non-inhibit	Bound	Non-hepatotoxin
**83**	Good	Good	Low	Non-inhibit	Bound	Hepatotoxin
**84**	Good	Good	Medium	Non-inhibit	Bound	Hepatotoxin
**86**	Good	Good	Medium	Non-inhibit	Bound	Hepatotoxin
**88**	Good	Good	Undefined	Non-inhibit	Bound	Hepatotoxin
**97**	Good	Good	Low	Non-inhibit	Bound	Non-hepatotoxin

Notes: AS = Aqueous Solubility; HIA = Human Intestinal Absorption; BBB = Blood Brain Barrier; CYP2D6 = cytochrome P450 2D6; PPB = Plasma Protein Binding; HT = Hepatotoxicity.

The blood brain barrier (BBB) descriptor relates to the ability of a compound to cross the blood brain barrier. High BBB penetration is a much sought-after property for central nervous system (CNS) targeted drugs, but for CNS unrelated diseases, it is undesirable. Therefore, the BBB descriptor can acts as a filter to improve efficiency of drug developments for CNS related diseases. As shown in Table 7, the Analogues **9**, **25**, **28**, **84** and **86** could be potential candidates for use against CNS inflammatory disorders due to their moderate to high BBB penetration, while **64**, **81**, **83**, **88** and **97** could be more suitable for CNS unrelated diseases due to their low BBB penetration.

Cytochrome P450 2D6 (CYP2D6) encompasses a class of enzymes, which catalyze the oxidative metabolism of drugs in the liver. It can either metabolize a drug from its active form into its inactive metabolites or convert an inactive drug into its active metabolites. Therefore, CYP2D6 inhibitory factors should be considered in reducing toxicity caused by the inactive metabolites for the former case but it should be avoided to preserve drug efficiency for the latter. The ADMET analysis conducted on our diarylpentenedione analogues indicates that all the selected compounds are non-CYP2D6 inhibitors on account of their inactive behavior towards CYP2D6. On the other hand, plasma protein binding is an important factor that determines the drug efficiency since only the unbound fraction is responsible for pharmacological effects. As presented in Table 7, all selected compounds were expected to be highly bound to protein plasma, thus implying that a high dosage might be required to achieve therapeutic concentration in treatments.

Lastly, the hepatotoxicity descriptor was used to predict potential organ toxicity caused by the compounds. As displayed in Table 7, only two compounds (**81** and **97**) were non-hepatotoxic while the rest were calculated as hepatotoxin. On account of these, more studies must be carried out to investigate the hepatotoxic effect of the Analogues **9**, **25**, **28**, **64**, **83**, **84**, **86** and **88** in addition to their optimal therapeutic dosage.

### 2.6. TOPKAT Analysis

Toxicity Prediction by Komputer Assisted Technology (TOPKAT) is a common method used to predict the ecotoxicity, toxicity, mutagenicity, and reproductive or developmental toxicity of selected candidates. At the early research stage, the utilization of TOPKAT predictions may be useful in prioritizing promising compounds for further development and investigation. Besides, it can also act as a factor to accelerate optimization of lead compounds in terms of their therapeutic ratios in both animal and human models.

In this study, the ten selected analogues from ADMET analysis were further screened for toxicity prediction including aerobic biodegradability, mutagenicity, rodent carcinogenicity, ocular irritancy, skin irritancy, and skin sensitization parameters. From the results, it may be generalized that all compounds were non-mutagenic, non-carcinogen and non-skin irritant. Analogues **9** and **64** were predicted as biodegradable while **28**, **84**, **86** and **88** were found to be ocular irritants. Compound **84** is the only candidate expected to be a non-skin sensitizer. Integration of both ADMET and TOPKAT analyses revealed that the Analogues **81** and **97** could be good candidates for further investigation based on their low toxicity and good aqueous solubility and intestinal absorption. Of the two, **97** appeared to be the most potent candidate as it exhibited two-fold better properties than **81**. However, extensive toxicity studies should be carried out on **88** since it exhibited the strongest activity among the diarylpentenedione analogues. The results from this prediction exercise are presented in Table 8.

**Table 8:** Results of toxicity predictive test on six important parameters.

Compounds	AB	AM	RC	OI	SI	SS
**9**	Biodegradable	Non-mutagen	Non-carcinogen	Non-irritant	Non-irritant	Sensitizer
**25**	Non-biodegradable	Non-mutagen	Non-carcinogen	Non-irritant	Non-irritant	Sensitizer
**28**	Non-biodegradable	Non-mutagen	Non-carcinogen	Irritant	Non-irritant	Sensitizer
**64**	Biodegradable	Non-mutagen	Non-carcinogen	Non-irritant	Non-irritant	Sensitizer
**81**	Non-biodegradable	Non-mutagen	Non-carcinogen	Non-irritant	Non-irritant	Sensitizer
**83**	Non-biodegradable	Non-mutagen	Non-carcinogen	Non-irritant	Non-irritant	Sensitizer
**84**	Non-biodegradable	Non-mutagen	Non-carcinogen	Irritant	Non-irritant	Non-sensitizer
**86**	Non-biodegradable	Non-mutagen	Non-carcinogen	Irritant	Non-irritant	Sensitizer
**88**	Non-biodegradable	Non-mutagen	Non-carcinogen	Irritant	Non-irritant	Sensitizer
**97**	Non-biodegradable	Non-mutagen	Non-carcinogen	Non-irritant	Non-irritant	Sensitizer

Notes: AB = Aerobic biodegradability; AM = Ames mutagenicity; RC = Rodent carcinogenicity; OI = Ocular irritancy; SI = Skin irritancy; SS = Skin sensitization.

### 2.7. Chemical Stability Test

**Figure 8:**
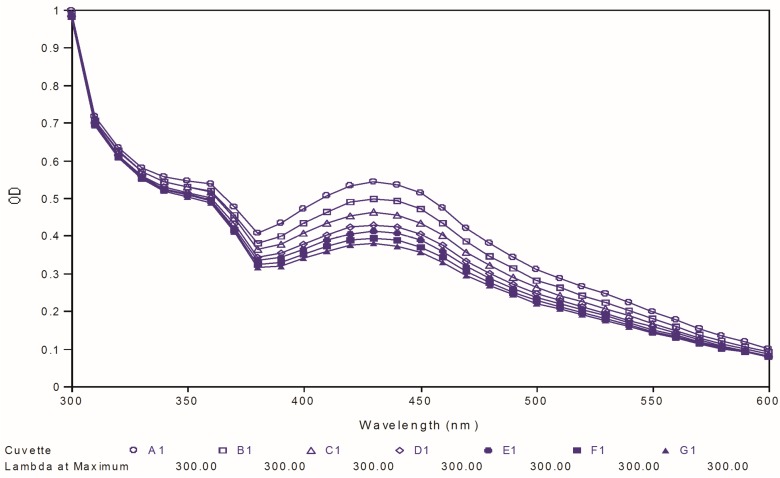
Ultraviolet-visible absorption spectra of curcumin.

**Figure 9:**
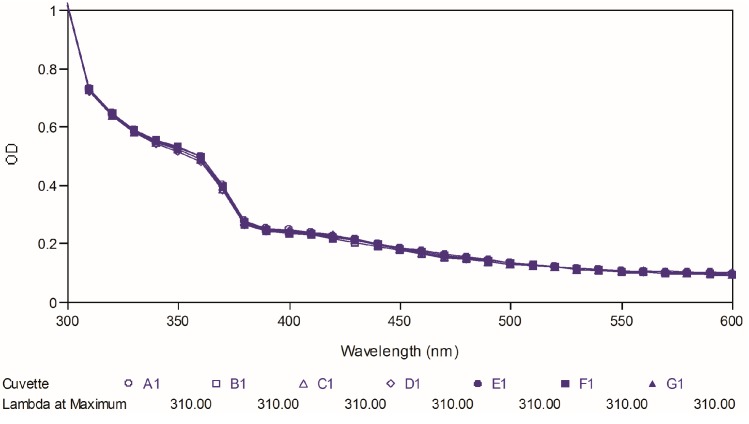
Ultraviolet-visible absorption spectra of compound **88**.

**Figure 10:**
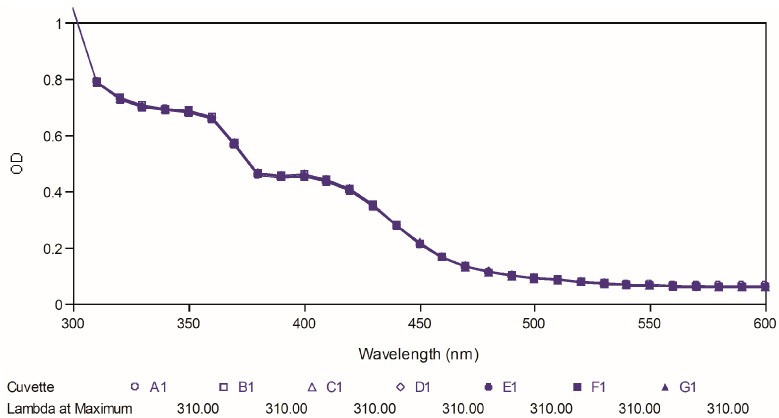
Ultraviolet-visible absorption spectra of compound **97**.

Poor chemical stability of curcumin has been proven as one of the important factors, which cause its low bioavailability. Therefore, in the present study, we further tested the two best compounds (**88** and **97**) for their chemical stability at physiological pH and compared it to that of curcumin used as reference. The test was carried out by observing the ultraviolet spectral changes of the targeted compounds for 30 minutes with 5-minutes intervals in phosphate buffer (pH 7.4). Figure 8, Figure 9 and Figure 10 depict the ultraviolet changes of curcumin, compound **88** and compound **97**, respectively. As shown in Figure 8, the maximal absorption peaks of curcumin gradually decreased over time. However, no significant changes were observed in the case of two best compounds, which indicated that our targeted compounds were chemically more stable than curcumin *in vitro*.

## 3. Experimental Section

### 3.1. Chemistry

All chemicals and reagents were purchased from Sigma-Aldrich and Merck. All solvents were dried and distilled before use. Reaction mixtures were extracted with organic solvents and dried over anhydrous magnesium sulfate followed by solvent evaporation with a rotary evaporator under reduced pressure. Analytical thin-layer chromatography (TLC) was routinely performed on 0.20 mm Merck silica gel 60 F_254_ TLC plate in every reaction step. Purification procedures were conducted using open column chromatography on Merck silica gel 60 (mesh 70–230). Melting points were determined using Fisher-Johns melting point apparatus and were uncorrected. Mass spectra were measured on a gas chromatography-mass spectrometry (GCMS)-QP5050A (Shimadzu, Kyoto, Japan) Mass Spectrometer. High-resolution electron ionization-mass spectrometry (HREI-MS) was determined using a DFS high resolution GC/MS (Thermo Scientific, San Jose, CA, USA). Nuclear Magnetic Resonance (NMR) spectra were recorded on a Varian 500 MHz NMR Spectrometer. For the bioassay, the purity of compounds was routinely checked based on a ThermoFinnigan Surveyor HPLC, utilizing Waters Xbridge C18 column (5 µm, 150 mm × 4.6 mm).

### 3.2. General Procedure for the Synthesis of **I**

A mixture of benzaldehyde (30 mmol), malonic acid (120 mmol), and piperidine (2 mL) in pyridine was refluxed in a 250 mL single neck round bottomed flask (SNRB) for 6 h. Upon completion, the reaction mixture was poured into a 1 L Erlenmeyer flask (EF) containing cold, dilute HCl (200 mL) and stirred for 10 min. The resulting precipitate was filtered and washed with cold water to afford **I**.

### 3.3. General Procedure for the Synthesis of **II**

Phosphoryl chloride (POCl_3_; 20 mmol) was added slowly to a 100 mL SNRB flask containing a mixture of **I** (5.5 mmol) and the appropriate 2'-hydroxyacetophenone (5 mmol) in pyridine (30 mL). The flask was placed in an ice bath and the reaction mixture was left overnight, with constant stirring at room temperature. The reaction mixture was then poured into 100 mL cold, dilute HCl in a 250 mL EF, followed by extraction with ethyl acetate (EA). The organic layer was dried over anhydrous magnesium sulfate and concentrated *in vacuo*. The crude product obtained was further purified by open column chromatography.

### 3.4. General Procedure for the Synthesis of **III**

Ground potassium hydroxide pellets (20 mmol) were added to a 100 mL SNRB flask containing a solution of **II** (4 mmol) in 30 mL pyridine. The reaction mixture was left overnight with constant stirring at room temperature. Upon completion, the reaction mixture was poured into 150 mL cold, diluted HCl in a 250 mL EF and stirred for 10 min. The solution was extracted with EA and dried over anhydrous magnesium sulfate, followed by solvent removal with rotatory evaporator under *vacuo*. The resulting crude product was further purified by open column chromatography.

### 3.5. General Procedure for Synthesis of **IV**

Boron tribromide (BBr_3_; 1.5 mL) was added to a 100 mL SNRB flask containing a solution of the methoxylated diarylpentanoid (0.3 mmol) in dry dichloromethane (30 mL) at 0 °C. The reaction mixture was stirred for 8 h at room temperature and then poured into 150 mL of cold water contained in a 250 mL EF. The solution was then extracted with EA and dried over anhydrous magnesium sulfate. The resulting organic layer was taken to dryness by rotary evaporation *in vacuo* and the product was further purified through open column chromatography.

### 3.6. Cell Culture

RAW 264.7 murine macrophages cells obtained from American Type Culture Collection (ATCC, Rockville, MD, USA) were grown in Dulbecco’s Modified Eagle’s Medium (DMEM) containing 10% fetal bovine serum (FBS) and 1% penicillin/streptomycin in a 95% air and 5% CO_2_ atmosphere at 37 °C.

### 3.7. Nitrite Determination

RAW 264.7 cells at 90%–95% confluency were detached and seeded (50,000 cells/well) into a 96-well culture plate with 50 μL of DMEM and incubated for 24 h. The cells were then stimulated in 5 mg/mL of LPS (*Escherichia coli*, serotype 0111:B4) and 1 ng/ml of interferon-gamma (IFN-γ) in the presence or absence of test compounds for 17 h. Nitrite concentration was then determined by Griess assay by reacting 50 μL of cell culture supernatant with 50 μL of Griess reagent (1% sulfanilamide and 0.1% *N*-(1-naphthyl)ethylenediamine dihydrochloride in 2.5% phosphoric acid) at room temperature. The optical density was measured at 550 nm after 5 min of incubation at room temperature with a microplate reader.

### 3.8. Cell Cytotoxicity Determination (MTT Assay)

Supernatant in each well was removed followed by addition of 100 µL DMEM. Subsequently, 20 µL of 3-(4,5-dimethylthiazol-2-yl)-2,5-diphenyltetrazolium bromide (MTT, 5 mg/mL) was then added and the plate was incubated in a 95% air and 5% CO_2_ atmosphere at 37 °C for 4 h. The mixture of culture media and MTT in all wells were removed and the purplish formazan crystals formed were dissolved in dimethyl sulfoxide (DMSO) and further incubated for 15 min at room temperature. The color intensity was then measured at 570 nm at room temperature.

### 3.9. 2D-QSAR

The genetic function approximation (GFA) technique was employed in 2D-QSAR analysis to study the correlation between structural features and their biological activities based on experimental pIC_50 _(−log IC_50_). Descriptors including the ALogP, number of the hydrogen bond donor, fingerprint EPFP_6, and molecular fractional polar surface area were used as the structural features. The equation generated was subjected to a randomization test if and only if its correlation coefficient, r^2^ was greater than eight and its cross-validation, q^2^, was greater than 0.5.

### 3.10. 3D-QSAR

Comparative molecular field analysis (CoMFA) was carried out to study the correlation between bioactive compounds and their 3D mapping, the electrostatic and van der Waals interactions. A total of fifty-seven compounds were selected and aligned at their common feature, the pentenedione fragment with compound **88** as template molecule. Then, the training set (42 compounds) and test set (15 compounds) were randomly generated after the minimization of ligands through a CHARMm force field. The electrostatic and van der Waals grid maps were created in a CHARMm force field with 2.0 grid spacing and five-fold cross validation.

### 3.11. Pharmacophore Mapping

Pharmacophore mapping was generated based on the activity of compounds and their common features through Discovery Studio 3.1. Five active and five inactive compounds were selected to inspect the validity of generated pharmacophore mapping.

### 3.12. ADMET and TOPKAT Analysis

The ten best compounds were selected for Discovery Studio 3.1 ADMET and TOPKAT analyses based on their pIC_50_. The ADMET analysis performed was on the aqueous solubility (AS), human intestinal absorption (HIA), blood brain barrier (BBB), cytochrome P450 2D6 (CYP2D6), plasma protein binding (PPB), and hepatotoxicity (HT) descriptors. Toxicity prediction was performed using TOPKAT analysis on the aspects of aerobic biodegradability, mutagenicity, rodent carcinogenicity, ocular irritancy, skin irritancy, and skin sensitization descriptors.

### 3.13. Chemical Stability Test

Absorbance readings were taken from 300–600 nm using a SpectraMax Plus 384 (Molecular Devices LLC, Sunnyvale, CA, USA). A stock solution of 50 mM curcumin or active compounds **88** and **97** were prepared and diluted by phosphate buffer (pH 7.4), containing 1% dimethyl sulfoxide (DMSO), to a final concentration of 20 μM. The ultraviolet absorption spectra were collected for over 30 min at 5-min intervals at 25 °C. All spectral measurements were carried out in a 1 cm path-length quartz cuvette.

## 4. Conclusions

In summary, most of the synthesized diarylpentenedione analogues were active in inhibiting NO production in IFN-γ/LPS-stimulated RAW 264.7 macrophages. This result supports our hypothesis that the diarylpentanoid structure with preserved ethylene and β-diketone moieties is an important lead template and should be investigated further towards finding new anti-inflammatory agents. The Analogues **88** and **97** with IC_50_ values of 4.9 and 9.6 µM, respectively, are the candidates with the most potential as they possess the highest activity and excellent chemical stability. The combination of computational exercises such as 2D-QSAR, 3D-QSAR, and pharmacophore mapping has provided better insights into the structure-activity relationship of the diarylpentenedione system. Further computational analysis such as ADMET and TOPKAT analyses also provided us with important information about drug efficiency and possible toxicity of the compounds. Phenolic diarylpentenediones are recommended for further investigation, while the presence of a 3,4-dihydroxyl moiety is suggested to be the most important functional group contributing to NO inhibition.

## Supplementary Materials

Supplementary Figure can be found at http://www.mdpi.com/1420-3049/19/10/16058/s1.

## Supplementary Files

Supplementary File 1
