# Supplementary material for: Synthesis and Sar Study of Diarylpentanoid Analogues as New Anti-Inflammatory Agents
Source: Molecules. 2014 Oct 9;19(10):16058–81. doi: 10.3390/molecules191016058 (PMC6271425; doi:10.3390/molecules191016058)

Supplementary Materials

Table S1. Spectrometric data of compounds **1–97**.

Compounds	Structure	^{1}H-NMR & ^{13}C-NMR	Calculated Mass	Mass Found	Colour	MP (˚C)	Ref MP (˚C)	Yield (%)
**1**	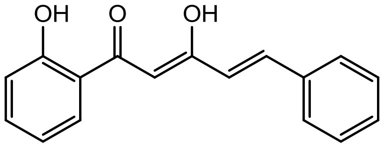	^{1}H-NMR (500 MHz, CHLOROFORM-*d*) δ ppm 6.32 (s, 1 H) 6.60 (d, *J* = 16.02 Hz, 1 H) 6.91 (t, *J* = 7.57 Hz, 1 H) 7.00 (d, *J* = 8.15 Hz, 1 H) 7.35–7.50 (m, 4 H) 7.56 (d, *J* = 7.28 Hz, 2 H) 7.66 (d, *J* = 16.02 Hz, 1 H) 7.70 (d, *J* = 7.86 Hz, 1 H) 12.24 (s, 1 H) 14.66 (s, 1 H)<br><br>^{13}C-NMR (126 MHz, CHLOROFORM-*d*) δ ppm 97.04, 118.76, 119.04, 119.07, 122.12, 128.03, 128.53, 128.98, 130.12, 134.99, 135.83, 139.90, 162.62, 174.45, 196.00	266.0943	266.0948	Yellow	129–131	131–132	55.21
**2**	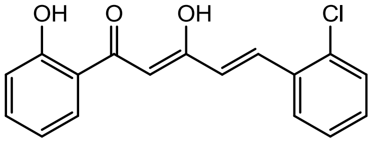	^{1}H-NMR (500 MHz, CHLOROFORM-*d*) δ ppm 6.33 (s, 1 H) 6.58 (d, *J* = 15.73 Hz, 1 H) 6.85–6.94 (m, 1 H) 6.99 (dd, *J* = 8.44, 0.87 Hz, 1 H) 7.29 (dd, *J* = 5.97, 3.64 Hz, 2 H) 7.39–7.48 (m, 2 H) 7.65 (dd, *J* = 5.68, 3.64 Hz, 1 H) 7.69 (dd, *J* = 8.01, 1.31 Hz, 1 H) 8.05 (d, *J* = 15.73 Hz, 1 H) 12.19 (s, 1 H) 14.55 (s, 1 H)<br><br>^{13}C-NMR (126 MHz, CHLOROFORM-*d*) δ ppm 97.46, 118.79, 119.01, 119.07, 124.62, 127.09, 127.38, 128.55, 130.29, 130.78, 133.19, 135.00, 135.48, 135.97, 162.67, 173.73, 196.20	300.0553	300.0558	Yellow	117–119	-	73.25

**3**	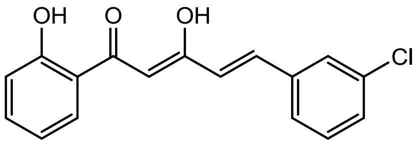	^{1}H-NMR (500 MHz, CHLOROFORM-*d*) δ ppm 6.31 (s, 1 H) 6.56 (d, *J* = 15.73 Hz, 1 H) 6.90 (t, *J* = 7.57 Hz, 1 H) 6.99 (d, *J* = 8.44 Hz, 1 H) 7.28–7.36 (m, 2 H) 7.40 (d, *J* = 6.41 Hz, 1 H) 7.45 (t, *J* = 7.86 Hz, 1 H) 7.53 (br. s., 1 H) 7.55 (d, *J* = 16.02 Hz, 1 H) 7.68 (d, *J* = 8.15 Hz, 1 H)<br><br>^{13}C-NMR (126 MHz, CHLOROFORM-*d*) δ ppm 97.50, 118.80, 118.99, 119.10, 123.53, 126.25, 127.54, 128.53, 129.85, 130.18, 134.99, 136.00, 136.83, 138.03, 162.68, 173.55, 196.20	300.0553	300.0558	Yellow	127–128	-	72.87
**4**	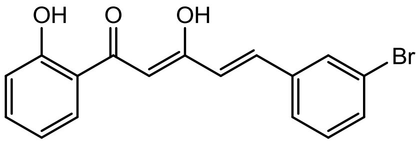	^{1}H-NMR (500 MHz, CHLOROFORM-*d*) δ ppm 6.32 (s, 1 H) 6.57 (d, *J* = 15.73 Hz, 1 H) 6.91 (t, *J* = 7.72 Hz, 1 H) 6.99 (d, *J* = 8.44 Hz, 1 H) 7.24–7.30 (m, 1 H) 7.41–7.51 (m, 3 H) 7.55 (d, *J* = 15.73 Hz, 1 H) 7.66–7.72 (m, 2 H) 12.17 (s, 1 H) 14.54 (s, 1 H)<br><br>^{13}C-NMR (126 MHz, CHLOROFORM-*d*) δ ppm 97.51, 118.80, 118.99, 119.10, 123.13, 123.56, 126.70, 128.53, 130.44, 130.48, 132.75, 136.01, 137.11, 137.93, 162.68, 173.51, 196.21	344.0048	344.0050	Yellow	118–119	-	82.32

**5**	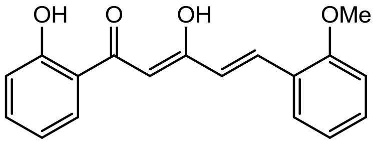	^{1}H-NMR (500 MHz, CHLOROFORM-*d*) δ ppm 3.92 (s, 3 H) 6.32 (s, 1 H) 6.71 (d, *J* = 16.02 Hz, 1 H) 6.90 (t, *J* = 7.57 Hz, 1 H) 6.94 (d, *J* = 8.15 Hz, 1 H) 6.96–7.03 (m, 2 H) 7.36 (t, *J* = 7.86 Hz, 1 H) 7.45 (t, *J* = 7.72 Hz, 1 H) 7.55 (d, *J* = 7.86 Hz, 1 H) 7.71 (d, *J* = 7.86 Hz, 1 H) 7.98 (d, *J* = 16.02 Hz, 1 H) 12.28 (s, 1 H) 14.72 (s, 1 H)<br><br>^{13}C-NMR (126 MHz, CHLOROFORM-*d*) δ ppm 55.55, 96.75, 111.23, 118.70, 118.96, 119.14, 120.81, 122.69, 123.95, 128.48, 128.78, 131.39, 135.56, 135.62, 158.39, 162.54, 175.40, 195.74	296.1049	296.1052	Yellow	100–102	-	52.45
**6**	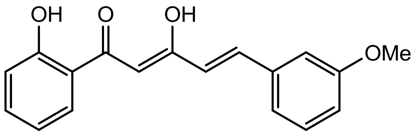	^{1}H-NMR (500 MHz, CHLOROFORM-*d*) δ ppm 3.85 (s, 3 H) 6.31 (s, 1 H) 6.57 (d, *J* = 15.73 Hz, 1 H) 6.88–6.92 (m, 1 H) 6.94 (ddd, *J* = 8.23, 2.55, 0.87 Hz, 1 H) 6.99 (dd, *J* = 8.30, 1.02 Hz, 1 H) 7.05–7.09 (m, 1 H) 7.15 (dt, *J* = 7.57, 0.73 Hz, 1 H) 7.29–7.34 (m, 1 H) 7.45 (ddd, *J* = 8.45, 6.99, 1.46 Hz, 1 H) 7.62 (d, *J* = 15.73 Hz, 1 H) 7.69 (dd, *J* = 8.15, 1.46 Hz, 1 H) 12.22 (s, 1 H) 14.63 (s, 1 H)<br><br>^{13}C-NMR (126 MHz, CHLOROFORM-*d*) δ ppm 55.31, 97.09, 112.98, 115.93, 118.76, 119.03, 119.05, 120.69, 122.42, 128.52, 129.95, 135.84, 136.36, 139.80, 159.98, 162.62, 174.32, 196.01	296.1049	296.1054	Yellow	101–103	NR	55.11

**7**	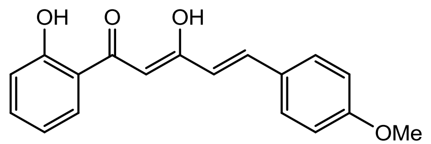	^{1}H-NMR (500 MHz, CHLOROFORM-*d*) δ ppm 3.85 (s, 3 H) 6.27 (s, 1 H) 6.45 (d, *J* = 15.73 Hz, 1 H) 6.86–6.95 (m, 3 H) 6.99 (d, *J* = 8.15 Hz, 1 H) 7.44 (t, *J* = 7.72 Hz, 1 H) 7.50 (d, *J* = 8.44 Hz, 2 H) 7.62 (d, *J* = 15.73 Hz, 1 H) 7.69 (d, *J* = 7.86 Hz, 1 H) 12.28 (s, 1 H) 14.75 (s, 1 H)<br><br>^{13}C-NMR (126 MHz, CHLOROFORM-*d*) δ ppm 55.41, 96.43, 114.47, 118.70, 118.97, 119.12, 119.65, 127.74, 128.43, 129.73, 135.60, 139.78, 161.37, 162.52, 175.19, 195.57	296.1049	296.1053	Yellow	116–117	128–130	57.21
**8**	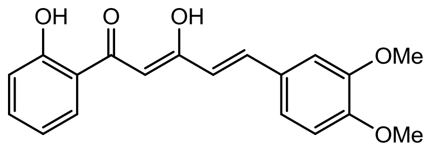	^{1}H-NMR (500 MHz, CHLOROFORM-*d*) δ ppm 3.92 (s, 3 H) 3.94 (s, 3 H) 6.29 (s, 1 H) 6.46 (d, *J* = 15.73 Hz, 1 H) 6.84–6.90 (m, 2 H) 6.98 (d, *J* = 8.15 Hz, 1 H) 7.07 (d, *J* = 1.46 Hz, 1 H) 7.13 (dd, *J* = 8.15, 1.46 Hz, 1 H) 7.40–7.45 (m, 1 H) 7.60 (d, *J* = 15.73 Hz, 1 H) 7.68 (d, *J* = 8.15 Hz, 1 H) 12.26 (s, 1 H) 14.73 (s, 1 H)<br><br>^{13}C-NMR (126 MHz, CHLOROFORM-*d*) δ ppm 55.92, 56.01, 96.50, 109.68, 111.18, 118.71, 118.96, 119.10, 119.87, 122.60, 128.00, 128.40, 135.63, 139.98, 149.29, 151.12, 162.53, 175.01, 195.58	326.1154	326.1157	Orange	123–125	126–128	32.46

**9**	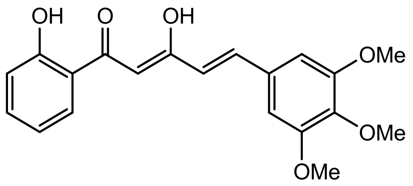	^{1}H-NMR (500 MHz, CHLOROFORM-*d*) δ ppm 3.90 (s, 3 H) 3.91 (d, *J* = 0.58 Hz, 6 H) 6.32 (s, 1 H) 6.49 (d, *J* = 15.73 Hz, 1 H) 6.77 (s, 2 H) 6.88 (t, *J* = 7.57 Hz, 1 H) 6.98 (d, *J* = 8.15 Hz, 1 H) 7.44 (t, *J* = 7.86 Hz, 1 H) 7.56 (d, *J* = 15.73 Hz, 1 H) 7.68 (d, *J* = 8.15 Hz, 1 H) 12.22 (s, 1 H) 14.65 (s, 1 H)<br><br>^{13}C-NMR (126 MHz, CHLOROFORM-*d*) δ ppm 56.16, 56.18, 61.02, 96.89, 105.21, 118.74, 119.00, 119.06, 121.37, 128.44, 130.51, 135.78, 139.88, 153.49, 162.59, 174.44, 195.83	356.1260	356.1265	Yellow	128–129	-	15.64
**10**	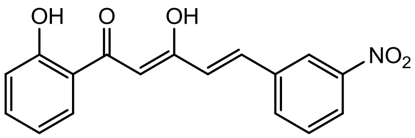	^{1}H-NMR (500 MHz, acetone) δ ppm 6.88–7.03 (m, 3 H) 7.21 (d, *J* = 16.02 Hz, 1 H) 7.47 (s, 1 H) 7.72 (dd, *J* = 12.09, 3.06 Hz, 2 H) 7.85 (d, *J* = 7.57 Hz, 1 H) 8.11–8.24 (m, 2 H) 8.56 (s, 1 H) 11.35 (br. s., 1 H) 14.69–15.94 (br. s., 1 H)<br><br>^{13}C-NMR (126 MHz, acetone) δ ppm 101.04, 117.74, 119.43, 120.59, 122.25, 124.14, 126.23, 129.52, 130.46, 134.28, 135.34, 136.56, 136.73, 148.39, 159.69, 175.42, 192.41	311.0794	311.0799	Yellow	184–186	187–194	81.67
**11**	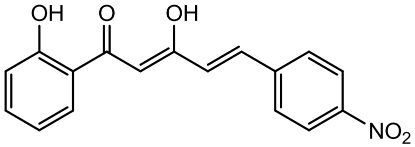	^{1}H-NMR (500 MHz, CHLOROFORM-*d*) δ ppm 6.40 (s, 1 H) 6.72 (d, *J* = 14.56 Hz, 1 H) 6.85–7.09 (m, 2 H) 7.41–7.82 (m, 5 H) 8.27 (s, 2 H) 12.10 (br. s., 1 H) 14.45 (br. s., 1 H)<br><br>^{13}C-NMR (126 MHz, CHLOROFORM-*d*) δ ppm 98.37, 118.91, 119.17, 124.26, 126.44, 128.39, 128.58, 136.32, 136.48, 141.20, 148.19, 162.80, 172.36, 196.54	311.0794	311.0799	Orange	191–193	198–199	82.53

**12**	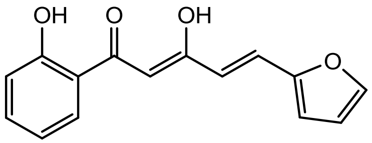	^{1}H-NMR (500 MHz, CHLOROFORM-*d*) δ ppm 6.29 (s, 1 H) 6.47–6.53 (m, 2 H) 6.62 (d, *J* = 3.20 Hz, 1 H) 6.89 (t, *J* = 7.57 Hz, 1 H) 6.98 (d, *J* = 8.15 Hz, 1 H) 7.41 (d, *J* = 15.43 Hz, 1 H) 7.43–7.47 (m, 1 H) 7.51 (s, 1 H) 7.68 (d, *J* = 7.86 Hz, 1 H) 12.24 (s, 1 H) 14.60 (s, 1 H)<br><br>^{13}C-NMR (126 MHz, CHLOROFORM-*d*) δ ppm 97.02, 112.61, 114.76, 118.71, 119.01, 119.10, 119.98, 126.22, 128.48, 135.72, 144.84, 151.66, 162.57, 174.28, 195.72	256.0736	256.0738	Orange	116–117	-	32.87
**13**	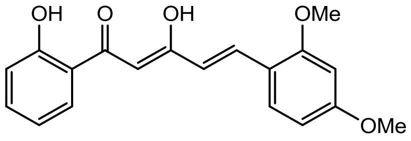	^{1}H-NMR (500 MHz, CHLOROFORM-*d*) δ ppm 3.84 (s, 3 H) 3.89 (s, 3 H) 6.26 (s, 1 H) 6.46 (d, *J* = 2.04 Hz, 1 H) 6.52 (dd, *J* = 8.59, 2.18 Hz, 1 H) 6.60 (d, *J* = 16.02 Hz, 1 H) 6.88 (t, *J* = 7.57 Hz, 1 H) 6.98 (d, *J* = 8.15 Hz, 1 H) 7.42 (t, *J* = 7.72 Hz, 1 H) 7.47 (d, *J* = 8.74 Hz, 1 H) 7.68 (d, *J* = 7.86 Hz, 1 H) 7.89 (d, *J* = 15.73 Hz, 1 H) 12.34 (s, 1 H) 14.82 (s, 1 H)<br><br>^{13}C-NMR (126 MHz, CHLOROFORM-*d*) δ ppm 55.50, 55.54, 96.18, 98.44, 105.52, 117.15, 118.62, 118.90, 119.21, 120.02, 128.40, 130.38, 135.38, 135.74, 159.95, 162.44, 162.81, 176.24, 195.21	326.1154	326.1157	Orange	134–135	-	30.64

**14**	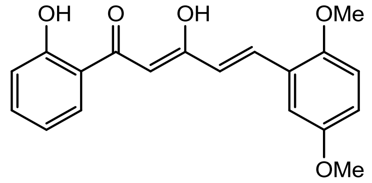	^{1}H-NMR (500 MHz, CHLOROFORM-*d*) δ ppm 3.81 (d, *J* = 1.46 Hz, 3 H) 3.86 (s, 3 H) 6.30 (s, 1 H) 6.67 (d, *J* = 16.02 Hz, 1 H) 6.82–6.92 (m, 3 H) 6.98 (d, *J* = 8.45 Hz, 1 H) 7.07 (s, 1 H) 7.44 (t, *J* = 7.72 Hz, 1 H) 7.69 (d, *J* = 8.15 Hz, 1 H) 7.94 (d, *J* = 16.02 Hz, 1 H) 12.27 (s, 1 H) 14.68 (s, 1 H)<br><br>^{13}C-NMR (126 MHz, CHLOROFORM-*d*) δ ppm 55.81, 56.09, 96.87, 112.44, 113.22, 116.94, 118.68, 118.97, 119.12, 122.87, 124.48, 128.50, 135.26, 135.66, 152.92, 153.58, 162.55, 175.18, 195.75	326.1154	326.1157	Yellow	89–90	-	27.89
**15**	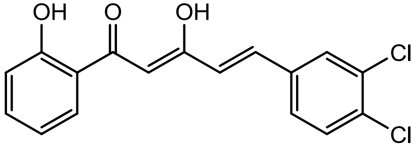	^{1}H-NMR (500 MHz, CHLOROFORM-*d*) δ ppm 6.33 (s, 1 H) 6.57 (d, *J* = 14.56 Hz, 1 H) 6.78–7.08 (m, 2 H) 7.31–7.56 (m, 4 H) 7.58–7.73 (m, 2 H) 12.14 (br. s., 1 H) 14.51 (br. s., 1 H)<br><br>^{13}C-NMR (126 MHz, CHLOROFORM-*d*) δ ppm 97.66, 118.83, 118.96, 119.10, 123.95, 126.97, 128.51, 129.32, 130.93, 133.31, 133.85, 135.09, 136.09, 136.83, 162.71, 173.15, 196.29	334.0163	334.0169	Yellow	172–174	-	75.11
**16**	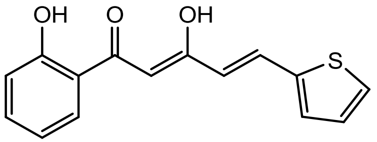	^{1}H-NMR (500 MHz, CHLOROFORM-*d*) δ ppm 6.27 (s, 1 H) 6.38 (d, *J* = 15.73 Hz, 1 H) 6.85–6.93 (m, 1 H) 6.99 (d, *J* = 8.15 Hz, 1 H) 7.07 (dd, *J* = 4.95, 3.79 Hz, 1 H) 7.26 (d, *J* = 3.20 Hz, 1 H) 7.39 (d, *J* = 4.95 Hz, 1 H) 7.42–7.48 (m, 1 H) 7.64–7.71 (m, 1 H) 7.76 (d, *J* = 15.43 Hz, 1 H) 12.24 (s, 1 H) 14.64 (s, 1 H)<br><br>^{13}C-NMR (126 MHz, CHLOROFORM-*d*) δ ppm 96.83, 118.73, 119.02, 119.07, 121.11, 128.34, 128.48, 128.51, 130.74, 132.55, 135.76, 140.56, 162.59, 174.16, 195.74	272.0507	272.0513	Orange	124–125	-	72.55

**17**	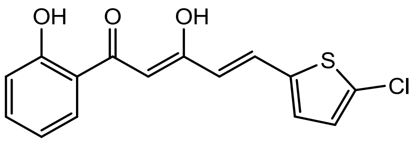	^{1}H-NMR (500 MHz, CHLOROFORM-*d*) δ ppm 6.25 (d, *J* = 15.73 Hz, 1 H) 6.27 (s, 1 H) 6.87–6.93 (m, 2 H) 6.99 (d, *J* = 8.15 Hz, 1 H) 7.04 (d, *J* = 3.49 Hz, 1 H) 7.42–7.49 (m, 1 H) 7.61 (d, *J* = 15.43 Hz, 1 H) 7.69 (d, *J* = 8.15 Hz, 1 H) 12.19 (s, 1 H) 14.59 (s, 1 H)<br><br>^{13}C-NMR (126 MHz, CHLOROFORM-*d*) δ ppm 97.06, 118.75, 119.01, 119.03, 121.16, 127.56, 128.47, 130.20, 131.81, 133.21, 135.86, 139.27, 162.60, 173.54, 195.85	306.0117	306.0123	Yellow	129–130	-	71.12
**18**	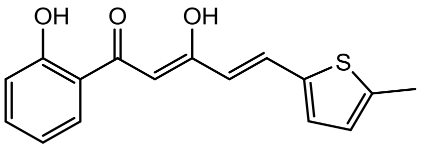	^{1}H-NMR (500 MHz, CHLOROFORM-*d*) δ ppm 2.53 (s, 3 H) 6.21–6.29 (m, 2 H) 6.72–6.76 (m, 1 H) 6.89 (t, *J* = 7.57 Hz, 1 H) 6.98 (d, *J* = 8.44 Hz, 1 H) 7.08 (d, *J* = 3.49 Hz, 1 H) 7.40–7.48 (m, 1 H) 7.65–7.73 (m, 2 H) 12.26 (s, 1 H) 14.70 (s, 1 H)<br><br>^{13}C-NMR (126 MHz, CHLOROFORM-*d*) δ ppm 15.91, 96.41, 118.68, 118.95, 119.10, 119.66, 126.81, 128.40, 131.59, 133.09, 135.59, 138.57, 144.31, 162.50, 174.65, 195.45	286.0664	286.0668	Orange	116–118	-	64.33

**19**	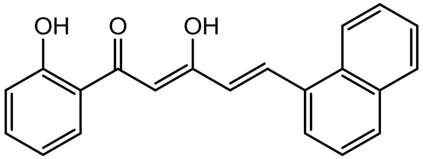	^{1}H-NMR (500 MHz, CHLOROFORM-*d*) δ ppm 6.38 (s, 1 H) 6.70 (d, *J* = 15.43 Hz, 1 H) 6.87–6.95 (m, 1 H) 7.01 (d, *J* = 8.44 Hz, 1 H) 7.45–7.50 (m, 1 H) 7.50–7.58 (m, 2 H) 7.59–7.63 (m, 1 H) 7.73 (dd, *J* = 8.15, 1.16 Hz, 1 H) 7.82 (d, *J* = 7.28 Hz, 1 H) 7.91 (t, *J* = 7.43 Hz, 2 H) 8.26 (d, *J* = 8.45 Hz, 1 H) 8.51 (d, *J* = 15.73 Hz, 1 H) 12.24 (s, 1 H) 14.71 (s, 1 H)<br><br>^{13}C-NMR (126 MHz, CHLOROFORM-*d*) δ ppm 97.20, 118.79, 119.05, 119.09, 123.42, 124.65, 124.80, 125.50, 126.30, 126.93, 128.53, 128.78, 130.45, 131.49, 132.39, 133.78, 135.87, 136.69, 162.64, 174.25, 196.14	316.1099	316.1100	Yellow	105–106	-	53.81
**20**	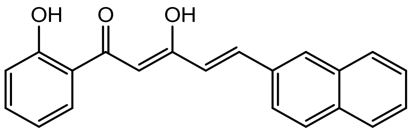	^{1}H-NMR (500 MHz, CHLOROFORM-*d*) δ ppm 6.38 (s, 1 H) 6.73 (d, *J* = 15.73 Hz, 1 H) 6.92 (t, *J* = 7.57 Hz, 1 H) 7.01 (d, *J* = 8.45 Hz, 1 H) 7.43–7.49 (m, 1 H) 7.50–7.56 (m, 2 H) 7.73 (dd, *J* = 7.43, 5.39 Hz, 2 H) 7.79–7.91 (m, 4 H) 7.98 (s, 1 H) 12.24 (s, 1 H) 14.69 (s, 1 H)<br><br>^{13}C-NMR (126 MHz, CHLOROFORM-*d*) δ ppm 97.06, 118.79, 119.04, 119.12, 122.36, 123.46, 126.79, 127.27, 127.82, 128.50, 128.59, 128.77, 129.86, 132.51, 133.44, 134.15, 135.82, 140.00, 162.64, 174.49, 195.97	316.1099	316.1100	Yellow	173–175	-	49.21

**21**	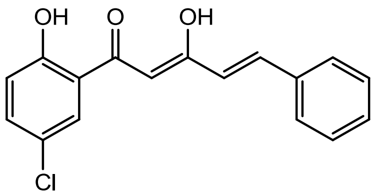	^{1}H-NMR (500 MHz, CHLOROFORM-*d*) δ ppm 6.23 (s, 1 H) 6.59 (d, *J* = 16.02 Hz, 1 H) 6.93 (d, *J* = 9.03 Hz, 1 H) 7.37 (dd, *J* = 8.88, 2.18 Hz, 1 H) 7.39–7.44 (m, 3 H) 7.55 (d, *J* = 6.70 Hz, 2 H) 7.63 (d, *J* = 2.33 Hz, 1 H) 7.67 (d, *J* = 15.73 Hz, 1 H) 12.16 (s, 1 H) 14.55 (s, 1 H)<br><br>^{13}C-NMR (126 MHz, CHLOROFORM-*d*) δ ppm 96.78, 119.75, 120.30, 121.79, 123.75, 127.70, 128.12, 129.02, 130.33, 134.82, 135.47, 140.65, 161.06, 175.28, 194.61	300.0553	300.0557	Yellow	132–134	-	76.59
**22**	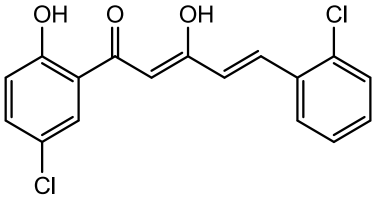	^{1}H-NMR (500 MHz, CHLOROFORM-*d*) δ ppm 6.27 (s, 1 H) 6.60 (d, *J* = 15.73 Hz, 1 H) 6.95 (d, *J* = 8.74 Hz, 1 H) 7.28–7.35 (m, 2 H) 7.36–7.49 (m, 2 H) 7.60–7.70 (m, 2 H) 8.08 (d, *J* = 16.02 Hz, 1 H) 12.11 (s, 1 H) 14.46 (s, 1 H)<br><br>^{13}C-NMR (126 MHz, CHLOROFORM-*d*) δ ppm 97.25, 119.71, 120.36, 123.79, 124.29, 127.13, 127.42, 127.73, 130.35, 130.97, 133.05, 135.13, 135.65, 136.25, 161.12, 174.56, 194.91	334.0163	334.0169	Yellow	149–150	-	80.56
**23**	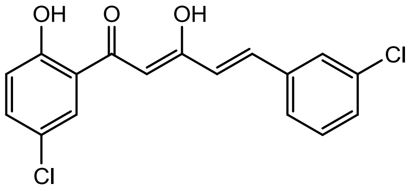	^{1}H-NMR (500 MHz, CHLOROFORM-*d*) δ ppm 6.26 (s, 1 H) 6.59 (d, *J* = 15.73 Hz, 1 H) 6.94 (d, *J* = 9.03 Hz, 1 H) 7.31–7.45 (m, 4 H) 7.54 (s, 1 H) 7.59 (d, *J* = 15.73 Hz, 1 H) 7.64 (d, *J* = 2.33 Hz, 1 H) 12.09 (s, 1 H) 14.45 (s, 1 H)<br><br>^{13}C-NMR (126 MHz, CHLOROFORM-*d*) δ ppm 97.24, 119.67, 120.35, 123.23, 123.83, 126.29, 127.67, 127.73, 130.06, 130.22, 135.06, 135.68, 136.66, 138.78, 161.11, 174.40, 194.91	334.0163	334.0169	Yellow	157–159	-	82.31

**23**	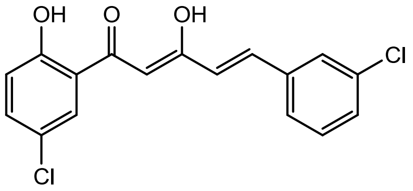	^{1}H-NMR (500 MHz, CHLOROFORM-*d*) δ ppm 6.26 (s, 1 H) 6.59 (d, *J* = 15.73 Hz, 1 H) 6.94 (d, *J* = 9.03 Hz, 1 H) 7.31–7.45 (m, 4 H) 7.54 (s, 1 H) 7.59 (d, *J* = 15.73 Hz, 1 H) 7.64 (d, *J* = 2.33 Hz, 1 H) 12.09 (s, 1 H) 14.45 (s, 1 H)<br><br>^{13}C-NMR (126 MHz, CHLOROFORM-*d*) δ ppm 97.24, 119.67, 120.35, 123.23, 123.83, 126.29, 127.67, 127.73, 130.06, 130.22, 135.06, 135.68, 136.66, 138.78, 161.11, 174.40, 194.91	334.0163	334.0169	Yellow	157–159	-	82.31
**24**	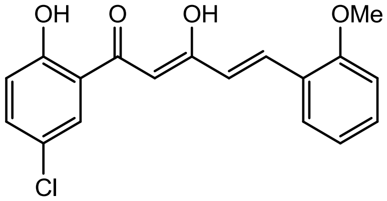	^{1}H-NMR (500 MHz, CHLOROFORM-*d*) δ ppm 3.93 (s, 3 H) 6.23 (s, 1 H) 6.73 (d, *J* = 16.02 Hz, 1 H) 6.89–6.96 (m, 2 H) 6.99 (s, 1 H) 7.34–7.40 (m, 2 H) 7.54 (dd, *J* = 7.57, 1.46 Hz, 1 H) 7.65 (d, *J* = 2.33 Hz, 1 H) 7.99 (d, *J* = 15.73 Hz, 1 H) 12.21 (s, 1 H) 14.63 (s, 1 H)<br><br>^{13}C-NMR (126 MHz, CHLOROFORM-*d*) δ ppm 55.56, 96.54, 111.25, 119.87, 120.24, 120.85, 122.41, 123.66, 123.77, 127.68, 129.03, 131.62, 135.27, 136.41, 158.52, 160.99, 176.30, 194.32	330.0659	330.0662	Yellow	134–136	-	46.34
**25**	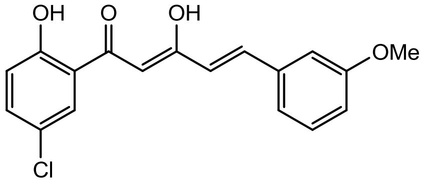	^{1}H-NMR (500 MHz, CHLOROFORM-*d*) δ ppm 3.85 (s, 3 H) 6.24 (s, 1 H) 6.58 (d, *J* = 15.73 Hz, 1 H) 6.91–6.97 (m, 2 H) 7.07 (s, 1 H) 7.15 (d, *J* = 7.57 Hz, 1 H) 7.32 (t, *J* = 7.86 Hz, 1 H) 7.38 (dd, *J* = 8.88, 2.48 Hz, 1 H) 7.60–7.67 (m, 2 H) 12.14 (s, 1 H) 14.53 (s, 1 H)<br><br>^{13}C-NMR (126 MHz, CHLOROFORM-*d*) δ ppm 55.31, 96.84, 112.93, 116.24, 119.75, 120.30, 120.83, 122.08, 123.75, 127.70, 129.99, 135.49, 136.18, 140.57, 160.00, 161.05, 175.18, 194.64	330.0659	330.0662	Yellow	99–100	-	48.96

**25**	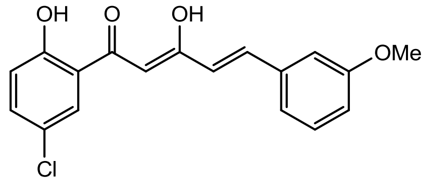	^{1}H-NMR (500 MHz, CHLOROFORM-*d*) δ ppm 3.85 (s, 3 H) 6.24 (s, 1 H) 6.58 (d, *J* = 15.73 Hz, 1 H) 6.91–6.97 (m, 2 H) 7.07 (s, 1 H) 7.15 (d, *J* = 7.57 Hz, 1 H) 7.32 (t, *J* = 7.86 Hz, 1 H) 7.38 (dd, *J* = 8.88, 2.48 Hz, 1 H) 7.60–7.67 (m, 2 H) 12.14 (s, 1 H) 14.53 (s, 1 H)<br><br>^{13}C-NMR (126 MHz, CHLOROFORM-*d*) δ ppm 55.31, 96.84, 112.93, 116.24, 119.75, 120.30, 120.83, 122.08, 123.75, 127.70, 129.99, 135.49, 136.18, 140.57, 160.00, 161.05, 175.18, 194.64	330.0659	330.0662	Yellow	99–100	-	48.96
**26**	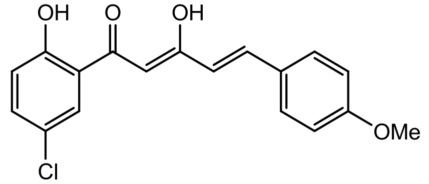	^{1}H-NMR (500 MHz, CHLOROFORM-*d*) δ ppm 3.85 (s, 3 H) 6.19 (s, 1 H) 6.46 (d, *J* = 15.73 Hz, 1 H) 6.93 (d, *J* = 8.74 Hz, 3 H) 7.36 (dd, *J* = 8.88, 2.48 Hz, 1 H) 7.51 (d, *J* = 8.74 Hz, 2 H) 7.60–7.67 (m, 2 H) 12.21 (s, 1 H) 14.66 (s, 1 H)<br><br>^{13}C-NMR (126 MHz, CHLOROFORM-*d*) δ ppm 55.43, 96.19, 114.51, 119.31, 119.84, 120.23, 123.67, 127.57, 127.61, 129.86, 135.24, 140.55, 160.96, 161.55, 176.04, 194.13	330.0659	330.0662	Orange	129–130	-	58.12
**27**	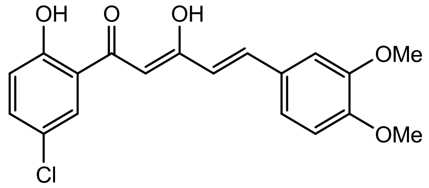	^{1}H-NMR (500 MHz, CHLOROFORM-*d*) δ ppm 3.93 (s, 3 H) 3.94 (s, 3 H) 6.22 (s, 1 H) 6.47 (d, *J* = 15.73 Hz, 1 H) 6.89 (d, *J* = 8.45 Hz, 1 H) 6.93 (d, *J* = 8.74 Hz, 1 H) 7.07 (s, 1 H) 7.14 (d, *J* = 8.15 Hz, 1 H) 7.36 (dd, *J* = 8.88, 1.89 Hz, 1 H) 7.56–7.69 (m, 2 H) 12.19 (s, 1 H) 14.64 (s, 1 H)<br><br>^{13}C-NMR (126 MHz, CHLOROFORM-*d*) δ ppm 55.92, 56.02, 96.26, 109.60, 111.17, 119.53, 119.83, 120.25, 122.87, 123.67, 127.60, 127.84, 135.27, 140.76, 149.33, 151.32, 160.97, 175.86, 194.17	360.0765	360.0770	Yellow	172–173	-	52.11

**28**	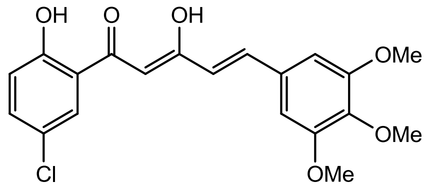	^{1}H-NMR (500 MHz, CHLOROFORM-*d*) δ ppm 3.89–3.94 (m, 9 H) 6.26 (s, 1 H) 6.51 (d, *J* = 15.73 Hz, 1 H) 6.79 (s, 2 H) 6.94 (d, *J* = 8.74 Hz, 1 H) 7.38 (dd, *J* = 8.88, 2.18 Hz, 1 H) 7.60 (d, *J* = 15.43 Hz, 1 H) 7.65 (d, *J* = 2.33 Hz, 1 H) 12.15 (s, 1 H) 14.57 (br. s., 1 H)<br><br>^{13}C-NMR (126 MHz, CHLOROFORM-*d*) δ ppm 56.17, 56.20, 61.05, 96.64, 105.29, 119.78, 120.31, 121.04, 123.73, 127.65, 130.31, 135.44, 140.32, 140.66, 153.53, 161.03, 175.30, 194.47	390.0870	390.0874	Orange	158–159	-	23.73
**29**	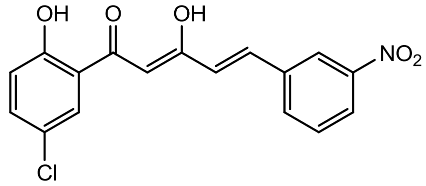	^{1}H-NMR (500 MHz, acetone) δ ppm 6.96 (s, 1 H) 7.03 (d, *J* = 8.45 Hz, 1 H) 7.23 (d, *J* = 15.73 Hz, 1 H) 7.49 (d, *J* = 7.86 Hz, 1 H) 7.68–7.85 (m, 3 H) 8.17 (d, *J* = 7.28 Hz, 1 H) 8.22 (d, *J* = 7.86 Hz, 1 H) 8.58 (s, 1 H) 11.38 (br. s., 1 H) 15.46 (br. s., 1 H)<br><br>^{13}C-NMR (126 MHz, acetone) δ ppm 101.72, 119.63, 122.33, 122.41, 123.10, 124.24, 126.25, 128.56, 130.48, 134.37, 136.67, 137.06, 148.42, 157.83, 176.63, 189.81	345.0404	345.0407	Yellow	223–224	-	82.17
**30**	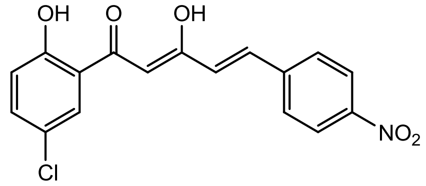	^{1}H-NMR (500 MHz, acetone) δ ppm 6.96 (s, 1 H) 7.02 (d, *J* = 8.44 Hz, 1 H) 7.21 (d, *J* = 15.27 Hz, 1 H) 7.49 (d, *J* = 7.57 Hz, 1 H) 7.70 (d, *J* = 15.73 Hz, 1 H) 7.81 (s, 1 H) 7.98 (d, *J* = 8.15 Hz, 2 H) 8.25 (d, *J* = 8.74 Hz, 2 H) 11.38 (s, 1 H) 15.39 (br. s., 1 H)<br><br>^{13}C-NMR (126 MHz, acetone) δ ppm 102.13, 119.62, 122.50, 123.10, 124.06, 127.67, 128.58, 129.16, 134.40, 136.74, 141.30, 147.71, 157.85, 176.01, 190.06	345.0404	345.0407	Yellow	243–245	-	80.98

**31**	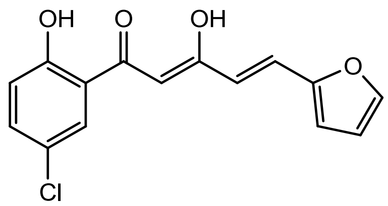	^{1}H-NMR (500 MHz, CHLOROFORM-*d*) δ ppm 6.21 (s, 1 H) 6.44–6.58 (m, 2 H) 6.64 (br. s., 1 H) 6.93 (d, *J* = 8.74 Hz, 1 H) 7.37 (d, *J* = 8.15 Hz, 1 H) 7.42 (d, *J* = 15.43 Hz, 1 H) 7.52 (br. s., 1 H) 7.62 (br. s., 1 H) 12.17 (s, 1 H) 14.51 (br. s., 1 H)<br><br>^{13}C-NMR (126 MHz, CHLOROFORM-*d*) δ ppm 96.77, 112.69, 115.23, 119.64, 119.80, 120.25, 123.72, 126.84, 127.67, 135.37, 145.10, 151.55, 161.01, 175.15, 194.31	290.0346	290.0350	Yellow	140–141	-	50.23
**32**	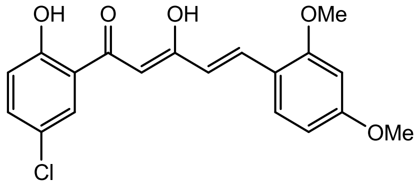	^{1}H-NMR (500 MHz, CHLOROFORM-*d*) δ ppm 3.85 (s, 3 H) 3.91 (s, 3 H) 6.17 (s, 1 H) 6.46 (d, *J* = 2.33 Hz, 1 H) 6.52 (d, *J* = 8.44 Hz, 1 H) 6.62 (d, *J* = 15.73 Hz, 1 H) 6.92 (d, *J* = 9.03 Hz, 1 H) 7.34 (d, *J* = 2.33 Hz, 1 H) 7.46 (d, *J* = 8.74 Hz, 1 H) 7.63 (d, *J* = 2.33 Hz, 1 H) 7.91 (d, *J* = 16.02 Hz, 1 H) 12.28 (s, 1 H) 14.73 (s, 1 H)<br><br>^{13}C-NMR (126 MHz, CHLOROFORM-*d*) δ ppm 55.52, 55.55, 95.97, 98.48, 105.54, 117.03, 119.74, 119.96, 120.15, 123.57, 127.58, 130.69, 134.99, 136.60, 160.12, 160.89, 163.00, 177.11, 193.73	360.0765	360.0770	Orange	136–138	-	42.12

**33**	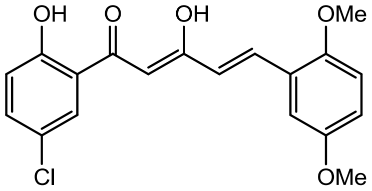	^{1}H-NMR (500 MHz, CHLOROFORM-*d*) δ ppm 3.82 (s, 3 H) 3.88 (s, 3 H) 6.24 (s, 1 H) 6.70 (d, *J* = 15.73 Hz, 1 H) 6.84–6.89 (m, 1 H) 6.90–6.96 (m, 2 H) 7.07 (d, *J* = 2.91 Hz, 1 H) 7.37 (dd, *J* = 8.88, 2.48 Hz, 1 H) 7.65 (d, *J* = 2.62 Hz, 1 H) 7.96 (d, *J* = 16.02 Hz, 1 H) 12.20 (s, 1 H) 14.60 (s, 1 H)<br><br>^{13}C-NMR (126 MHz, CHLOROFORM-*d*) δ ppm 55.82, 56.10, 96.63, 112.48, 113.23, 117.36, 119.85, 120.25, 122.59, 123.67, 124.28, 127.67, 135.30, 136.11, 153.06, 153.59, 161.00, 176.07, 194.37	360.0765	360.0770	Orange	146–148	-	48.22
**34**	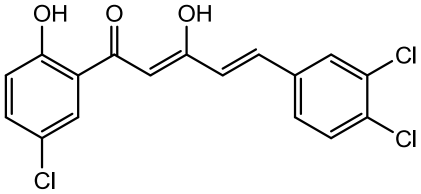	^{1}H-NMR (500 MHz, CHLOROFORM-*d*) δ ppm 6.28 (s, 1 H) 6.59 (d, *J* = 15.85 Hz, 1 H) 6.95 (d, *J* = 7.86 Hz, 1 H) 7.34–7.51 (m, 3 H) 7.56 (d, *J* = 15.73 Hz, 1 H) 7.65 (s, 2 H) 12.07 (s, 1 H) 14.42 (s, 1 H)<br><br>^{13}C-NMR (126 MHz, CHLOROFORM-*d*) δ ppm 97.43, 119.67, 120.39, 123.65, 123.87, 127.02, 127.74, 129.45, 130.99, 133.40, 134.12, 134.91, 135.77, 137.57, 161.14, 174.01, 195.02	367.9773	367.9780	Yellow	192–193	-	78.36

**35**	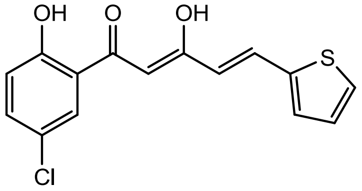	^{1}H-NMR (500 MHz, CHLOROFORM-*d*) δ ppm 6.20 (s, 1 H) 6.39 (d, *J* = 15.43 Hz, 1 H) 6.93 (d, *J* = 8.74 Hz, 1 H) 7.08 (dd, *J* = 5.10, 3.64 Hz, 1 H) 7.28 (d, *J* = 3.49 Hz, 1 H) 7.37 (dd, *J* = 8.88, 2.48 Hz, 1 H) 7.42 (d, *J* = 4.95 Hz, 1 H) 7.63 (d, *J* = 2.62 Hz, 1 H) 7.78 (d, *J* = 15.43 Hz, 1 H) 12.16 (s, 1 H) 14.54 (s, 1 H)<br><br>^{13}C-NMR (126 MHz, CHLOROFORM-*d*) δ ppm 96.57, 119.78, 120.27, 120.75, 123.74, 127.67, 128.40, 128.89, 131.08, 133.27, 135.41, 140.42, 161.01, 175.03, 194.35	306.0117	306.0121	Yellow	134–135	-	74.35
**36**	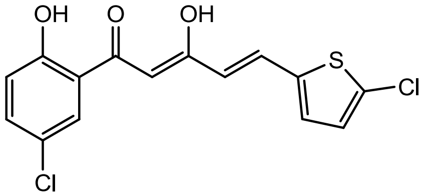	^{1}H-NMR (500 MHz, CHLOROFORM-*d*) δ ppm 6.21 (s, 1 H) 6.26 (d, *J* = 15.43 Hz, 1 H) 6.91 (d, *J* = 4.08 Hz, 1 H) 6.94 (d, *J* = 8.74 Hz, 1 H) 7.06 (d, *J* = 3.79 Hz, 1 H) 7.39 (dd, *J* = 8.88, 2.48 Hz, 1 H) 7.61–7.67 (m, 2 H) 12.12 (s, 1 H) 14.51 (s, 1 H)<br><br>^{13}C-NMR (126 MHz, CHLOROFORM-*d*) δ ppm 96.82, 119.74, 120.31, 120.81, 123.80, 127.66, 130.59, 132.52, 133.68, 135.55, 139.14, 161.05, 174.45, 194.52	339.9728	339.9736	Yellow	160–161	-	59.33

**37**	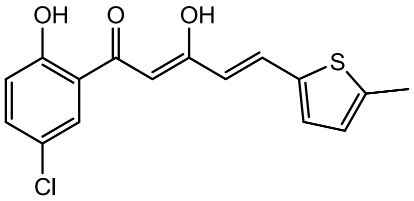	^{1}H-NMR (500 MHz, CHLOROFORM-*d*) δ ppm 2.54 (s, 3 H) 6.19 (s, 1 H) 6.26 (d, *J* = 15.43 Hz, 1 H) 6.75 (br. s., 1 H) 6.93 (d, *J* = 9.03 Hz, 1 H) 7.10 (d, *J* = 2.91 Hz, 1 H) 7.38 (d, *J* = 8.74 Hz, 1 H) 7.64 (s, 1 H) 7.72 (d, *J* = 15.43 Hz, 1 H) 12.20 (s, 1 H) 14.62 (s, 1 H)<br><br>^{13}C-NMR (126 MHz, CHLOROFORM-*d*) δ ppm 15.94, 96.21, 119.29, 119.84, 120.22, 123.68, 126.91, 127.61, 131.99, 133.83, 135.24, 138.44, 144.82, 160.93, 175.54, 194.03	320.0274	320.0276	Orange	130–132	-	55.11
**38**	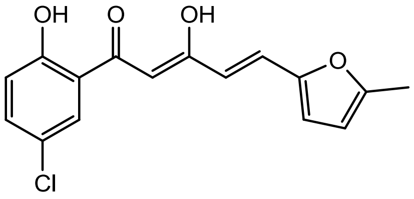	^{1}H-NMR (500 MHz, CHLOROFORM-*d*) δ ppm 2.39 (s, 3 H) 6.09–6.15 (m, 1 H) 6.20 (s, 1 H) 6.44 (d, *J* = 15.43 Hz, 1 H) 6.56 (d, *J* = 3.20 Hz, 1 H) 6.93 (d, *J* = 8.74 Hz, 1 H) 7.32–7.40 (m, 2 H) 7.62 (d, *J* = 2.33 Hz, 1 H) 12.23 (s, 1 H) 14.57 (s, 1 H)<br><br>^{13}C-NMR (126 MHz, CHLOROFORM-*d*) δ ppm 13.99, 96.37, 109.39, 117.25, 117.80, 119.89, 120.19, 123.65, 127.01, 127.60, 135.16, 150.19, 156.12, 160.92, 175.76, 193.90	304.0502	304.0507	Orange	115–116	-	38.22

**39**	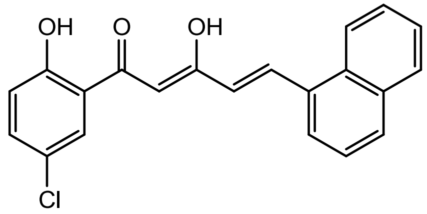	^{1}H-NMR (500 MHz, CHLOROFORM-*d*) δ ppm 6.32 (s, 1 H) 6.72 (d, *J* = 15.43 Hz, 1 H) 6.97 (d, *J* = 8.74 Hz, 1 H) 7.41 (dd, *J* = 8.88, 2.48 Hz, 1 H) 7.50–7.59 (m, 2 H) 7.59–7.64 (m, 1 H) 7.68 (d, *J* = 2.62 Hz, 1 H) 7.83 (d, *J* = 7.28 Hz, 1 H) 7.87–7.94 (m, 2 H) 8.25 (d, *J* = 8.45 Hz, 1 H) 8.55 (d, *J* = 15.73 Hz, 1 H) 12.17 (s, 1 H) 14.63 (s, 1 H)<br><br>^{13}C-NMR (126 MHz, CHLOROFORM-*d*) δ ppm 96.99, 119.80, 120.34, 123.34, 123.79, 124.28, 124.94, 125.52, 126.34, 127.01, 127.74, 128.82, 130.69, 131.50, 132.18, 133.79, 135.55, 137.43, 161.08, 175.10, 194.81	350.0710	350.0713	Orange	200–201	-	62.36
**40**	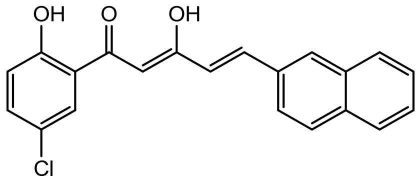	^{1}H-NMR (500 MHz, CHLOROFORM-*d*) δ ppm 6.31 (s, 1 H) 6.73 (d, *J* = 15.73 Hz, 1 H) 6.96 (d, *J* = 8.74 Hz, 1 H) 7.40 (dd, *J* = 8.88, 2.18 Hz, 1 H) 7.51–7.56 (m, 2 H) 7.68 (d, *J* = 2.04 Hz, 1 H) 7.72 (d, *J* = 8.74 Hz, 1 H) 7.82–7.91 (m, 4 H) 7.98 (s, 1 H) 12.18 (s, 1 H) 14.60 (s, 1 H)<br><br>^{13}C-NMR (126 MHz, CHLOROFORM-*d*) δ ppm 96.84, 119.82, 120.32, 122.02, 123.41, 123.77, 126.83, 127.39, 127.71, 127.82, 128.62, 128.83, 130.11, 132.33, 133.40, 134.24, 135.48, 140.76, 161.06, 175.35, 194.60	350.0710	350.0714	Orange	157–158	-	57.49

**41**	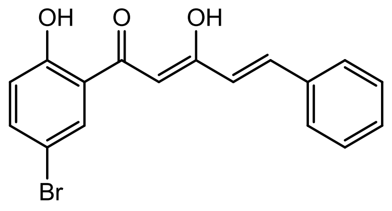	^{1}H-NMR (500 MHz, CHLOROFORM-*d*) δ ppm 6.24 (s, 1 H) 6.60 (d, *J* = 16.02 Hz, 1 H) 6.89 (d, *J* = 8.74 Hz, 1 H) 7.37–7.45 (m, 3 H) 7.50 (dd, *J* = 8.74, 1.16 Hz, 1 H) 7.56 (d, *J* = 7.28 Hz, 2 H) 7.68 (d, *J* = 16.02 Hz, 1 H) 7.78 (d, *J* = 1.16 Hz, 1 H) 12.17 (s, 1 H) 14.54 (s, 1 H)<br><br>^{13}C-NMR (126 MHz, CHLOROFORM-*d*) δ ppm 96.79, 110.71, 120.40, 120.71, 121.80, 128.12, 129.02, 130.33, 130.72, 134.82, 138.25, 140.68, 161.50, 175.30, 194.53	344.0048	344.0053	Yellow	129–131	-	77.85
**42**	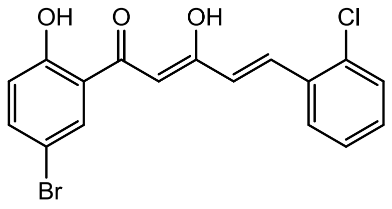	^{1}H-NMR (500 MHz, CHLOROFORM-*d*) δ ppm 6.27 (s, 1 H) 6.60 (d, *J* = 15.73 Hz, 1 H) 6.90 (d, *J* = 8.74 Hz, 1 H) 7.27–7.35 (m, 2 H) 7.39–7.47 (m, 1 H) 7.52 (d, *J* = 9.03 Hz, 1 H) 7.60–7.71 (m, 1 H) 7.79 (s, 1 H) 8.08 (d, *J* = 15.73 Hz, 1 H) 12.13 (s, 1 H) 14.44 (s, 1 H)<br><br>^{13}C-NMR (126 MHz, CHLOROFORM-*d*) δ ppm 97.27, 110.73, 120.35, 120.76, 124.29, 127.13, 127.42, 130.35, 130.76, 130.98, 133.04, 135.14, 136.27, 138.41, 161.56, 174.57, 194.82	377.9658	377.9662	Yellow	149–151	-	75.19

**43**	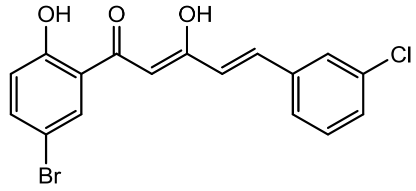	^{1}H-NMR (500 MHz, CHLOROFORM-*d*) δ ppm 6.27 (s, 1 H) 6.61 (d, *J* = 15.73 Hz, 1 H) 6.90 (dd, *J* = 8.88, 2.18 Hz, 1 H) 7.36 (d, *J* = 0.87 Hz, 2 H) 7.42 (d, *J* = 4.66 Hz, 1 H) 7.52 (d, *J* = 9.03 Hz, 1 H) 7.55 (s, 1 H) 7.60 (d, *J* = 16.02 Hz, 1 H) 7.79 (s, 1 H) 12.12 (s, 1 H) 14.44 (s, 1 H)<br><br>^{13}C-NMR (126 MHz, CHLOROFORM-*d*) δ ppm 97.26, 110.76, 120.34, 120.76, 123.25, 126.30, 127.68, 130.07, 130.23, 130.77, 135.07, 136.67, 138.45, 138.82, 161.55, 174.42, 194.83	377.9658	377.9662	Yellow	172–173	-	79.22
**44**	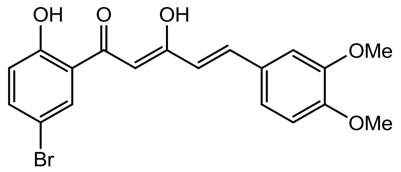	^{1}H-NMR (500 MHz, CHLOROFORM-*d*) δ ppm 3.93 (s, 3 H) 3.95 (s, 3 H) 6.22 (s, 1 H) 6.47 (d, *J* = 15.73 Hz, 1 H) 6.84–6.93 (m, 2 H) 7.08 (s, 1 H) 7.15 (d, *J* = 8.15 Hz, 1 H) 7.49 (d, *J* = 7.57 Hz, 1 H) 7.63 (d, *J* = 15.73 Hz, 1 H) 7.78 (s, 1 H) 12.22 (s, 1 H) 14.62 (s, 1 H)<br><br>^{13}C-NMR (126 MHz, CHLOROFORM-*d*) δ ppm 55.91, 56.01, 96.25, 109.59, 110.63, 111.18, 119.53, 120.48, 120.67, 122.90, 127.84, 130.62, 138.04, 140.79, 149.34, 151.33, 161.42, 175.88, 194.07	404.0259	404.0267	Yellow	179–180	-	65.78

**45**	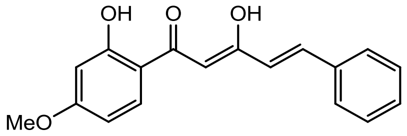	^{1}H-NMR (500 MHz, CHLOROFORM-*d*) δ ppm 3.83 (s, 3 H) 6.19 (s, 1 H) 6.43–6.48 (m, 2 H) 6.57 (d, J = 15.73 Hz, 1 H) 7.36–7.42 (m, 3 H) 7.52–7.56 (m, 2 H) 7.59 (s, 1 H) 7.61 (d, J = 6.99 Hz, 1 H) 12.69 (s, 1 H) 14.52 (s, 1 H)<br><br>^{13}C-NMR (126 MHz, CHLOROFORM-*d*) δ ppm 55.53, 96.80, 101.31, 107.89, 112.75, 122.34, 127.90, 128.93, 129.88, 130.13, 135.15, 139.02, 165.43, 165.90, 173.11, 194.65	296.1049	296.1053	Yellow	101–103	105–107	24.63
**46**	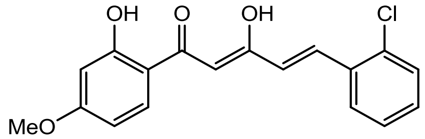	^{1}H-NMR (500 MHz, CHLOROFORM-*d*) δ ppm 3.84 (s, 3 H) 6.21 (s, 1 H) 6.42–6.48 (m, 2 H) 6.57 (d, J = 15.73 Hz, 1 H) 7.29 (dd, J = 5.68, 3.64 Hz, 2 H) 7.40–7.45 (m, 1 H) 7.60 (d, J = 8.74 Hz, 1 H) 7.63–7.67 (m, 1 H) 8.00 (d, J = 15.73 Hz, 1 H) 12.65 (s, 1 H) 14.43 (s, 1 H)<br><br>^{13}C-NMR (126 MHz, CHLOROFORM-*d*) δ ppm 55.59, 97.28, 101.30, 107.98, 112.73, 124.89, 127.05, 127.31, 130.15, 130.26, 130.56, 133.38, 134.68, 134.89, 165.53, 166.01, 172.44, 194.79	330.0659	330.0662	Yellow	151–152	-	39.72
**47**	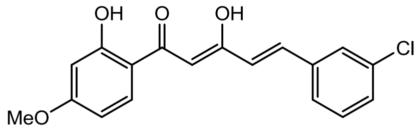	^{1}H-NMR (500 MHz, CHLOROFORM-*d*) δ ppm 3.84 (s, 3 H) 6.19 (s, 1 H) 6.41–6.46 (m, 2 H) 6.55 (d, J = 16.02 Hz, 1 H) 7.30–7.35 (m, 2 H) 7.36–7.42 (m, 1 H) 7.47–7.55 (m, 2 H) 7.58 (d, J = 8.74 Hz, 1 H) 12.63 (s, 1 H) 14.42 (s, 1 H)<br><br>^{13}C-NMR (126 MHz, CHLOROFORM-*d*) δ ppm 55.58, 97.33, 101.31, 107.99, 112.71, 123.76, 126.15, 127.43, 129.62, 130.14, 132.29, 134.94, 137.01, 137.19, 165.52, 166.04, 172.25, 194.77	330.0659	330.0662	Yellow	133–134	-	36.75

**48**	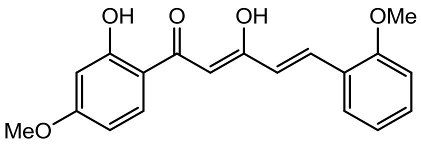	^{1}H-NMR (500 MHz, CHLOROFORM-*d*) δ ppm 3.84 (s, 3 H) 3.92 (s, 3 H) 6.19 (s, 1 H) 6.42–6.48 (m, 2 H) 6.69 (d, *J* = 16.02 Hz, 1 H) 6.93 (d, *J* = 8.45 Hz, 1 H) 6.98 (t, *J* = 7.43 Hz, 1 H) 7.34 (t, *J* = 7.86 Hz, 1 H) 7.54 (d, *J* = 7.86 Hz, 1 H) 7.61 (d, *J* = 8.44 Hz, 1 H) 7.93 (d, *J* = 16.02 Hz, 1 H) 12.74 (s, 1 H) 14.57 (s, 1 H)<br><br>^{13}C-NMR (126 MHz, CHLOROFORM-*d*) δ ppm 55.53, 55.55, 96.53, 101.28, 107.79, 111.20, 112.81, 120.78, 122.90, 124.11, 128.63, 130.09, 131.14, 134.65, 158.29, 165.33, 165.75, 174.05, 194.49	326.1154	326.1158	Yellow	134–135	-	22.18
**49**	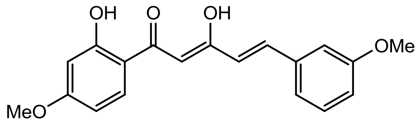	^{1}H-NMR (500 MHz, CHLOROFORM-*d*) δ ppm 3.84 (s, 3 H) 3.85 (s, 3 H) 6.19 (s, 1 H) 6.41–6.48 (m, 2 H) 6.55 (d, *J* = 15.73 Hz, 1 H) 6.92 (d, *J* = 8.15 Hz, 1 H) 7.06 (s, 1 H) 7.14 (d, *J* = 7.57 Hz, 1 H) 7.31 (t, *J* = 7.86 Hz, 1 H) 7.54–7.62 (m, 2 H) 12.68 (s, 1 H) 14.50 (s, 1 H)<br><br>^{13}C-NMR (126 MHz, CHLOROFORM-*d*) δ ppm 55.32, 55.57, 96.89, 101.29, 107.90, 112.75, 112.86, 115.71, 120.58, 122.65, 129.91, 130.12, 136.53, 138.92, 159.95, 165.44, 165.91, 173.00, 194.66	326.1154	326.1158	Yellow	124–125	-	28.97

**50**	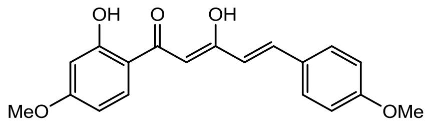	^{1}H-NMR (500 MHz, CHLOROFORM-*d*) δ ppm 3.83 (s, 3 H) 3.84 (s, 3 H) 6.15 (s, 1 H) 6.39–6.49 (m, 3 H) 6.92 (d, *J* = 8.74 Hz, 2 H) 7.49 (d, *J* = 8.74 Hz, 2 H) 7.54–7.61 (m, 2 H) 12.73 (s, 1 H) 14.60 (s, 1 H)<br><br>^{13}C-NMR (126 MHz, CHLOROFORM-*d*) δ ppm 55.40, 55.54, 96.19, 101.29, 107.79, 112.78, 114.42, 119.91, 127.90, 129.56, 130.02, 138.88, 161.18, 165.31, 165.73, 173.83, 194.35	326.1154	326.1159	Yellow	109–111	-	25.87
**51**	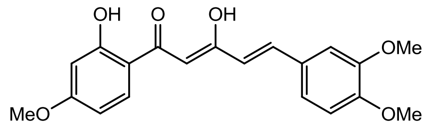	^{1}H-NMR (500 MHz, CHLOROFORM-*d*) δ ppm 3.83 (s, 3 H) 3.91 (s, 3 H) 3.93 (s, 3 H) 6.16 (s, 1 H) 6.40–6.47 (m, 3 H) 6.87 (d, *J* = 8.45 Hz, 1 H) 7.06 (s, 1 H) 7.12 (d, *J* = 8.15 Hz, 1 H) 7.55 (d, *J* = 15.73 Hz, 1 H) 7.58 (d, *J* = 8.74 Hz, 1 H) 12.71 (s, 1 H) 14.58 (s, 1 H)<br><br>^{13}C-NMR (126 MHz, CHLOROFORM-*d*) δ ppm 55.54, 55.90, 55.99, 96.26, 101.28, 107.80, 109.63, 111.17, 112.76, 120.13, 122.36, 128.17, 130.00, 139.08, 149.26, 150.91, 165.32, 165.76, 173.65, 194.35	356.1260	356.1265	Yellow	141–142	149-151	14.21
**52**	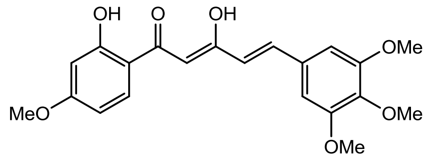	^{1}H-NMR (500 MHz, CHLOROFORM-*d*) δ ppm 3.82 (s, 3 H) 3.89 (s, 3 H) 3.90 (s, 6 H) 6.19 (s, 1 H) 6.40–6.49 (m, 3 H) 6.76 (s, 2 H) 7.51 (d, *J* = 15.73 Hz, 1 H) 7.58 (d, *J* = 9.03 Hz, 1 H) 12.68 (s, 1 H) 14.51 (s, 1 H)<br><br>^{13}C-NMR (126 MHz, CHLOROFORM-*d*) δ ppm 55.56, 56.17, 61.01, 96.66, 101.29, 105.07, 107.87, 112.73, 121.61, 130.05, 130.68, 139.01, 139.90, 153.47, 165.40, 165.87, 173.10, 194.51	386.1366	386.1367	Yellow	144–145	-	11.34

**53**	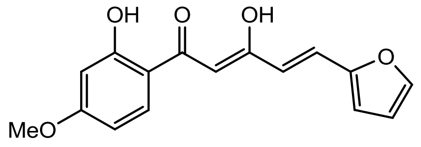	^{1}H-NMR (500 MHz, CHLOROFORM-*d*) δ ppm 3.84 (s, 3 H) 6.16 (s, 1 H) 6.43–6.50 (m, 4 H) 6.59 (d, *J* = 3.49 Hz, 1 H) 7.36 (d, *J* = 15.43 Hz, 1 H) 7.50 (d, *J* = 1.16 Hz, 1 H) 7.59 (d, *J* = 8.74 Hz, 1 H) 12.70 (s, 1 H) 14.46 (s, 1 H)<br><br>^{3}C-NMR (126 MHz, CHLOROFORM-*d*) δ ppm 55.56, 96.79, 101.27, 107.85, 112.51, 112.78, 114.27, 120.25, 125.49, 130.09, 144.59, 151.77, 165.38, 165.83, 172.95, 194.42	286.0841	286.0844	Yellow	136–137	-	17.88
**54**	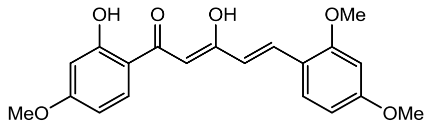	^{1}H-NMR (500 MHz, CHLOROFORM-*d*) δ ppm 3.84 (s, 3 H) 3.85 (s, 3 H) 3.90 (s, 3 H) 6.15 (s, 1 H) 6.41–6.48 (m, 3 H) 6.53 (d, *J* = 8.45 Hz, 1 H) 6.59 (d, *J* = 15.73 Hz, 1 H) 7.47 (d, *J* = 8.44 Hz, 1 H) 7.60 (d, *J* = 9.61 Hz, 1 H) 7.85 (d, *J* = 15.73 Hz, 1 H) 12.78 (s, 1 H) 14.66 (s, 1 H)<br><br>^{13}C-NMR (126 MHz, CHLOROFORM-*d*) δ ppm 55.51, 55.52, 55.53, 95.90, 98.48, 101.26, 105.43, 107.68, 112.85, 117.33, 120.33, 129.97, 130.18, 134.82, 159.82, 162.58, 165.21, 165.57, 174.85, 194.13	356.1260	356.1264	Yellow	154–155	-	12.38
**55**	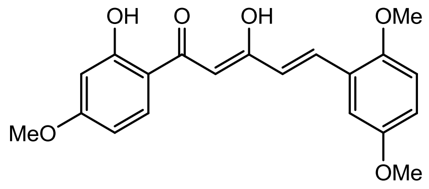	^{1}H-NMR (500 MHz, CHLOROFORM-*d*) δ ppm 3.81 (s, 3 H) 3.84 (s, 3 H) 3.87 (s, 3 H) 6.19 (s, 1 H) 6.39–6.48 (m, 2 H) 6.65 (d, *J* = 15.73 Hz, 1 H) 6.82–6.93 (m, 2 H) 7.07 (br. s., 1 H) 7.61 (d, *J* = 8.74 Hz, 1 H) 7.89 (d, *J* = 16.02 Hz, 1 H) 12.72 (s, 1 H) 14.54 (s, 1 H)<br><br>^{13}C-NMR (126 MHz, CHLOROFORM-*d*) δ ppm 55.55, 55.82, 56.12, 96.64, 101.27, 107.82, 112.44, 112.81, 113.14, 116.67, 123.13, 124.69, 130.09, 134.38, 152.83, 153.58, 165.35, 165.78, 173.83, 194.51	356.1260	356.1264	Yellow	152–153	-	17.59

**56**	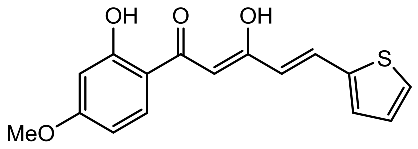	^{1}H-NMR (500 MHz, CHLOROFORM-*d*) δ ppm 3.83 (s, 3 H) 6.14 (s, 1 H) 6.36 (d, *J* = 15.43 Hz, 1 H) 6.41–6.47 (m, 2 H) 7.06 (dd, *J* = 4.95, 3.79 Hz, 1 H) 7.23 (d, *J* = 3.49 Hz, 1 H) 7.37 (d, *J* = 4.95 Hz, 1 H) 7.58 (d, *J* = 8.74 Hz, 1 H) 7.71 (d, *J* = 15.43 Hz, 1 H) 12.69 (s, 1 H) 14.50 (s, 1 H)<br><br>^{13}C-NMR (126 MHz, CHLOROFORM-*d*) δ ppm 55.56, 96.59, 101.29, 107.85, 112.76, 121.38, 128.13, 128.26, 130.09, 130.36, 131.72, 140.71, 165.39, 165.86, 172.82, 194.43	302.0613	302.0617	Yellow	108–110	-	38.33
**57**	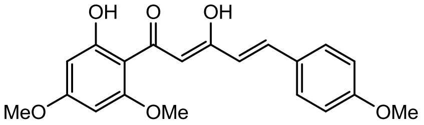	^{1}H-NMR (500 MHz, CHLOROFORM-*d*) δ ppm 3.81 (s, 3 H) 3.84 (s, 3 H) 3.89 (s, 3 H) 5.96 (d, *J* = 2.33 Hz, 1 H) 6.08 (d, *J* = 2.62 Hz, 1 H) 6.44 (d, *J* = 15.73 Hz, 1 H) 6.72 (s, 1 H) 6.91 (d, *J* = 8.74 Hz, 2 H) 7.48–7.53 (m, 3 H) 13.63 (s, 1 H) 14.83 (s, 1 H)<br><br>^{13}C-NMR (126 MHz, CHLOROFORM-*d*) δ ppm 55.39, 55.51, 55.76, 90.97, 91.32, 94.09, 102.49, 104.67, 114.35, 114.50, 128.16, 129.48, 130.14, 138.21, 161.82, 167.12, 173.95, 193.61	356.1260	356.1266	Yellow	122–124	-	24.17
**58**	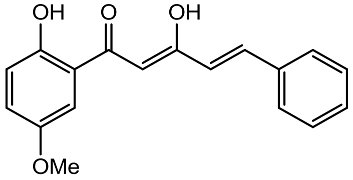	^{1}H-NMR (500 MHz, CHLOROFORM-*d*) δ ppm 3.82 (s, 3 H) 6.26 (s, 1 H) 6.60 (d, *J* = 15.73 Hz, 1 H) 6.93 (d, *J* = 9.03 Hz, 1 H) 7.09 (dd, *J* = 9.03, 2.91 Hz, 1 H) 7.13 (d, *J* = 2.91 Hz, 1 H) 7.35–7.44 (m, 3 H) 7.53–7.58 (m, 2 H) 7.66 (d, *J* = 16.02 Hz, 1 H) 11.81 (s, 1 H) 14.72 (s, 1 H)<br><br>^{13}C-NMR (126 MHz, CHLOROFORM-*d*) δ ppm 56.02, 97.04, 111.39, 118.66, 119.54, 122.11, 123.56, 128.03, 128.97, 130.12, 134.98, 139.97, 151.92, 156.98, 174.61, 195.55	296.1049	296.1053	Orange	90–92	-	56.27

**59**	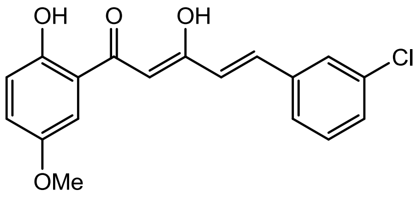	^{1}H-NMR (500 MHz, CHLOROFORM-*d*) δ ppm 3.83 (s, 3 H) 6.26 (s, 1 H) 6.58 (d, *J* = 15.73 Hz, 1 H) 6.93 (d, *J* = 8.74 Hz, 1 H) 7.07–7.13 (m, 2 H) 7.30–7.42 (m, 3 H) 7.51–7.60 (m, 2 H) 11.76 (s, 1 H) 14.61 (s, 1 H)<br><br>^{13}C-NMR (126 MHz, CHLOROFORM-*d*) δ ppm 55.98, 97.51, 111.25, 118.55, 119.60, 123.51, 123.85, 126.30, 127.50, 129.86, 130.18, 134.99, 136.83, 138.10, 151.94, 157.06, 173.71, 195.76	330.0659	330.0662	Orange	114–115	-	65.94
**60**	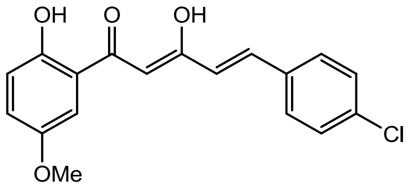	^{1}H-NMR (500 MHz, CHLOROFORM-*d*) δ ppm 3.80 (s, 3 H) 6.24 (s, 1 H) 6.55 (d, *J* = 15.73 Hz, 1 H) 6.93 (d, *J* = 9.03 Hz, 1 H) 7.06–7.12 (m, 2 H) 7.36 (d, *J* = 8.45 Hz, 2 H) 7.47 (d, *J* = 8.45 Hz, 2 H) 7.58 (d, *J* = 15.73 Hz, 1 H) 11.77 (s, 1 H) 14.66 (s, 1 H)<br><br>^{13}C-NMR (126 MHz, CHLOROFORM-*d*) δ ppm 56.00, 97.27, 111.37, 118.59, 119.57, 122.64, 123.66, 129.11, 129.23, 133.48, 135.95, 138.36, 151.92, 157.01, 174.05, 195.64	330.0659	330.0662	Orange	131–132	-	67.22
**61**	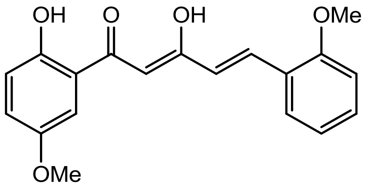	^{1}H-NMR (500 MHz, CHLOROFORM-*d*) δ ppm 3.82 (s, 3 H) 3.92 (s, 3 H) 6.25 (s, 1 H) 6.71 (d, *J* = 16.02 Hz, 1 H) 6.91–6.95 (m, 2 H) 6.99 (t, *J* = 7.57 Hz, 1 H) 7.08 (dd, *J* = 9.03, 2.91 Hz, 1 H) 7.14 (d, *J* = 3.20 Hz, 1 H) 7.32–7.39 (m, 1 H) 7.55 (dd, *J* = 7.57, 1.46 Hz, 1 H) 7.98 (d, *J* = 16.02 Hz, 1 H) 11.86 (s, 1 H) 14.79 (s, 1 H)<br><br>^{13}C-NMR (126 MHz, CHLOROFORM-*d*) δ ppm 55.56, 56.03, 96.77, 111.24, 111.52, 118.77, 119.45, 120.80, 122.61, 123.26, 123.93, 128.76, 131.42, 135.63, 151.88, 156.88, 158.40, 175.58, 195.29	326.1154	326.1157	Orange	134–135	-	48.12

**62**	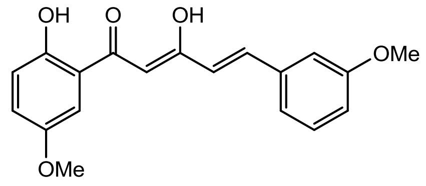	^{1}H-NMR (500 MHz, CHLOROFORM-*d*) δ ppm 3.82 (s, 3 H) 3.85 (s, 3 H) 6.25 (s, 1 H) 6.58 (d, *J* = 15.73 Hz, 1 H) 6.91–6.95 (m, 2 H) 7.06–7.10 (m, 2 H) 7.12 (d, *J* = 2.91 Hz, 1 H) 7.15 (d, *J* = 7.57 Hz, 1 H) 7.31 (t, *J* = 7.86 Hz, 1 H) 7.62 (d, *J* = 16.02 Hz, 1 H) 11.80 (s, 1 H) 14.70 (s, 1 H)<br><br>^{13}C-NMR (126 MHz, CHLOROFORM-*d*) δ ppm 55.33, 55.99, 97.06, 111.38, 113.01, 115.94, 118.64, 119.53, 120.69, 122.39, 123.58, 129.94, 136.34, 139.87, 151.92, 156.98, 159.98, 174.49, 195.55	326.1154	326.1157	Red	91–93	-	43.57
**63**	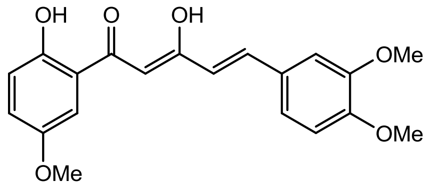	^{1}H-NMR (500 MHz, CHLOROFORM-*d*) δ ppm 3.82 (s, 3 H) 3.94 (s, 3 H) 3.95 (s, 3 H) 6.25 (s, 1 H) 6.48 (d, *J* = 15.73 Hz, 1 H) 6.90 (d, *J* = 8.15 Hz, 1 H) 6.93 (d, *J* = 9.03 Hz, 1 H) 7.05–7.18 (m, 4 H) 7.62 (d, *J* = 15.73 Hz, 1 H) 11.84 (s, 1 H) 14.82 (s, 1 H)<br><br>^{13}C-NMR (126 MHz, CHLOROFORM-*d*) δ ppm 50.73, 50.80, 50.83, 91.28, 104.50, 105.99, 106.24, 113.53, 114.29, 114.66, 117.41, 118.09, 122.80, 134.87, 144.10, 145.94, 146.69, 151.67, 169.99, 189.96	356.1260	356.1264	Orange	128–130	-	25.12
**64**	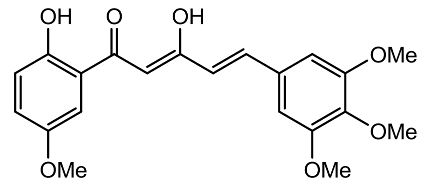	^{1}H-NMR (500 MHz, CHLOROFORM-*d*) δ ppm 3.81 (s, 3 H) 3.90 (s, 3 H) 3.91 (s, 6 H) 6.26 (s, 1 H) 6.50 (d, *J* = 15.73 Hz, 1 H) 6.78 (s, 2 H) 6.92 (d, *J* = 8.74 Hz, 1 H) 7.08 (dd, *J* = 9.03, 2.91 Hz, 1 H) 7.13 (d, *J* = 2.91 Hz, 1 H) 7.57 (d, *J* = 15.73 Hz, 1 H) 11.80 (s, 1 H) 14.74 (s, 1 H)<br><br>^{13}C-NMR (126 MHz, CHLOROFORM-*d*) δ ppm 56.03, 56.19, 61.03, 96.88, 105.23, 111.45, 118.67, 119.51, 121.32, 123.45, 130.48, 139.99, 140.14, 151.92, 153.50, 156.93, 174.62, 195.39	386.1366	386.1368	Orange	139–140	-	10.92

**65**	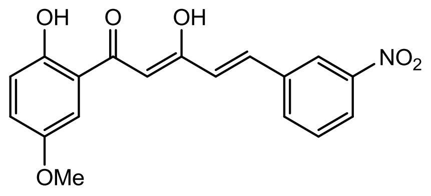	^{1}H-NMR (500 MHz, CHLOROFORM-*d*) δ ppm 3.84 (s, 3 H) 6.34 (s, 1 H) 6.74 (d, *J* = 15.73 Hz, 1 H) 6.96 (d, *J* = 8.74 Hz, 1 H) 7.09–7.17 (m, 2 H) 7.60 (t, *J* = 8.01 Hz, 1 H) 7.68 (d, *J* = 16.02 Hz, 1 H) 7.84 (d, *J* = 8.15 Hz, 1 H) 8.23 (d, *J* = 7.28 Hz, 1 H) 8.45 (s, 1 H) 11.73 (s, 1 H) 14.56 (br. s., 1 H)<br><br>^{13}C-NMR (126 MHz, CHLOROFORM-*d*) δ ppm 0.00, 56.04, 98.08, 111.17, 118.49, 119.71, 121.84, 124.14, 124.21, 125.23, 130.02, 133.83, 136.58, 136.81, 152.02, 157.19, 172.76, 196.05	341.0899	341.0899	Orange	180–181	-	65.33
**66**	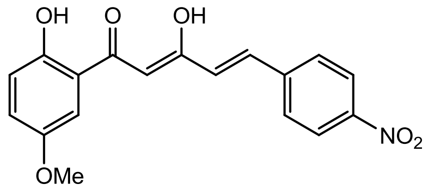	^{1}H-NMR (500 MHz, CHLOROFORM-*d*) δ ppm 3.83 (s, 3 H) 6.34 (s, 1 H) 6.73 (d, *J* = 15.73 Hz, 1 H) 6.96 (d, *J* = 9.32 Hz, 1 H) 7.08–7.17 (m, 2 H) 7.63–7.74 (m, 3 H) 8.27 (d, *J* = 7.57 Hz, 2 H) 11.70 (s, 1 H) 14.52 (br. s., 1 H)<br><br>^{13}C-NMR (126 MHz, CHLOROFORM-*d*) δ ppm 56.05, 98.41, 111.40, 118.52, 119.72, 124.10, 124.26, 126.42, 128.40, 136.56, 141.19, 152.00, 157.21, 172.52, 196.11	341.0899	341.0892	Red	202–203	-	61.10
**67**	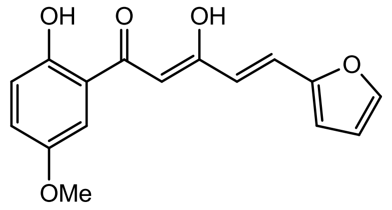	^{1}H-NMR (500 MHz, CHLOROFORM-*d*) δ ppm 3.81 (s, 3 H) 6.23 (s, 1 H) 6.47–6.54 (m, 2 H) 6.62 (d, *J* = 3.20 Hz, 1 H) 6.92 (d, *J* = 9.03 Hz, 1 H) 7.06–7.10 (m, 1 H) 7.11 (d, *J* = 2.91 Hz, 1 H) 7.40 (d, *J* = 15.43 Hz, 1 H) 7.51 (d, *J* = 1.46 Hz, 1 H) 11.82 (s, 1 H) 14.67 (s, 1 H)<br><br>^{13}C-NMR (126 MHz, CHLOROFORM-*d*) δ ppm 55.98, 97.01, 111.31, 112.62, 114.80, 118.69, 119.49, 119.96, 123.50, 126.27, 144.86, 151.66, 151.90, 156.94, 174.44, 195.27	286.0841	286.0845	Red	98–100	-	36.44

**68**	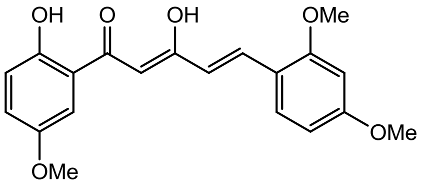	^{1}H-NMR (500 MHz, CHLOROFORM-*d*) δ ppm 3.84 (s, 3 H) 3.86 (s, 3 H) 3.91 (s, 3 H) 6.24 (s, 1 H) 6.40 (d, *J* = 2.18 Hz, 1 H) 6.44 (d, *J* = 15.73 Hz, 1 H) 6.50 (dd, *J* = 8.71, 2.18 Hz, 1 H) 6.89 (d, *J* = 9.03 Hz, 1 H) 7.07 (dd, *J* = 9.03, 2.93 Hz, 1 H) 7.11 (d, *J* = 2.93 Hz, 1 H) 7.46 (d, *J* = 8.71 Hz, 1 H) 7.61 (d, *J* = 15.73 Hz, 1 H) 11.62(s, 1 H) 14.71 (s, 1 H)<br><br>^{13}C-NMR (126 MHz, CHLOROFORM-*d*) δ ppm 55.54, 55.62, 56.20, 96.84, 98.99, 107.88, 112.01, 112.97, 114.21, 115.67, 124.77, 125.58, 131.74, 135.01, 152.13, 153.72, 162.67, 163.17, 175.21, 195.53	356.1260	356.1264	Orange	81–83	-	32.12
**69**	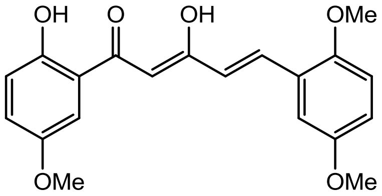	^{1}H-NMR (500 MHz, CHLOROFORM-*d*) δ ppm 3.81 (s, 3 H) 3.82 (s, 3 H) 3.87 (s, 3 H) 6.25 (s, 1 H) 6.68 (d, *J* = 16.02 Hz, 1 H) 6.84–6.87 (m, 1 H) 6.89–6.95 (m, 2 H) 7.05–7.11 (m, 2 H) 7.13 (d, *J* = 3.20 Hz, 1 H) 7.94 (d, *J* = 16.02 Hz, 1 H) 11.84 (s, 1 H) 14.76 (s, 1 H)<br><br>^{13}C-NMR (126 MHz, CHLOROFORM-*d*) δ ppm 55.81, 56.04, 56.10, 96.87, 111.50, 112.45, 113.22, 116.98, 118.74, 119.45, 122.83, 123.31, 124.48, 135.35, 151.88, 152.94, 153.59, 156.89, 175.35, 195.31	356.1260	356.1263	Orange	79–80	-	39.12

**70**	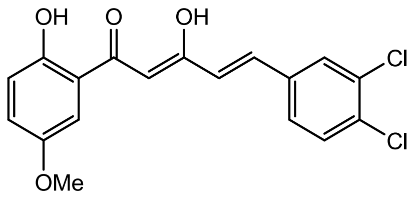	^{1}H-NMR (500 MHz, CHLOROFORM-*d*) δ ppm 3.82 (s, 3 H) 6.27 (s, 1 H) 6.57 (d, *J* = 15.73 Hz, 1 H) 6.94 (d, *J* = 8.74 Hz, 1 H) 7.11 (br. s., 2 H) 7.36 (d, *J* = 8.45 Hz, 1 H) 7.47 (d, *J* = 8.45 Hz, 1 H) 7.52 (d, *J* = 15.43 Hz, 1 H) 7.64 (br. s., 1 H) 11.74 (s, 1 H) 14.58 (br. s., 1 H)<br><br>^{13}C-NMR (126 MHz, CHLOROFORM-*d*) δ ppm 55.99, 97.65, 111.26, 118.53, 119.64, 123.93, 127.02, 129.28, 130.93, 133.32, 133.87, 135.07, 136.89, 151.96, 157.10, 173.32, 195.84	364.0269	364.0272	Brown	149–150	-	59.21
**71**	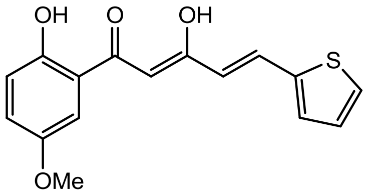	^{1}H-NMR (500 MHz, CHLOROFORM-*d*) δ ppm 3.82 (s, 3 H) 6.21 (s, 1 H) 6.39 (d, *J* = 15.43 Hz, 1 H) 6.92 (d, *J* = 8.74 Hz, 1 H) 7.06–7.10 (m, 2 H) 7.11 (d, *J* = 2.62 Hz, 1 H) 7.40 (d, *J* = 4.66 Hz, 1 H) 7.76 (d, *J* = 15.43 Hz, 1 H) 11.82 (s, 1 H) 14.71 (s, 1 H)<br><br>^{13}C-NMR (126 MHz, CHLOROFORM-*d*) δ ppm 56.00, 96.83, 111.25, 118.65, 119.52, 121.08, 123.60, 128.34, 128.53, 130.79, 132.62, 140.54, 151.92, 156.95, 174.33, 195.29	302.0613	302.0617	Red	91–93	-	64.13
**72**	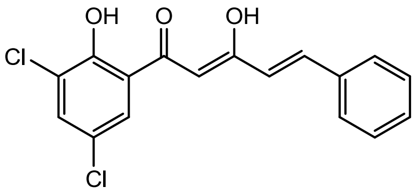	^{1}H-NMR (500 MHz, CHLOROFORM-*d*) δ ppm 6.23 (s, 1 H) 6.59 (d, *J* = 15.73 Hz, 1 H) 7.38–7.45 (m, 3 H) 7.52 (s, 1 H) 7.55 (br. s., 1 H) 7.57 (br. s., 2 H) 7.70 (d, *J* = 16.02 Hz, 1 H) 12.80 (s, 1 H) 14.40 (s, 1 H)<br><br>^{13}C-NMR (126 MHz, CHLOROFORM-*d*) δ ppm 96.74, 120.34, 121.49, 123.43, 124.13, 126.27, 128.21, 129.05, 130.55, 134.64, 134.99, 141.45, 156.89, 176.04, 193.86	334.0163	334.0169	Yellow	140–141	-	32.15

**73**	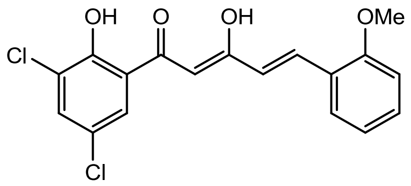	^{1}H-NMR (500 MHz, CHLOROFORM-*d*) δ ppm 3.94 (s, 3 H) 6.24 (s, 1 H) 6.74 (d, *J* = 15.73 Hz, 1 H) 6.95 (d, *J* = 8.45 Hz, 1 H) 7.00 (t, *J* = 7.57 Hz, 1 H) 7.38 (t, *J* = 7.86 Hz, 1 H) 7.51–7.57 (m, 2 H) 7.59 (d, *J* = 1.75 Hz, 1 H) 8.02 (d, *J* = 16.02 Hz, 1 H) 12.88 (s, 1 H) 14.49 (s, 1 H)<br><br>^{13}C-NMR (126 MHz, CHLOROFORM-*d*) δ ppm 55.58, 96.51, 111.29, 120.50, 120.88, 122.14, 123.34, 123.63, 124.05, 126.25, 129.20, 131.87, 134.79, 137.27, 156.85, 158.64, 177.07, 193.54	364.0269	364.0275	Yellow	169–170	-	25.65
**74**	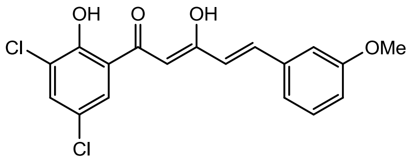	^{1}H-NMR (500 MHz, CHLOROFORM-*d*) δ ppm 3.86 (s, 3 H) 6.25 (s, 1 H) 6.59 (d, *J* = 15.73 Hz, 1 H) 6.96 (d, *J* = 7.86 Hz, 1 H) 7.08 (s, 1 H) 7.16 (d, *J* = 7.28 Hz, 1 H) 7.30–7.36 (m, 1 H) 7.52–7.60 (m, 2 H) 7.68 (d, *J* = 15.73 Hz, 1 H) 12.80 (s, 1 H) 14.40 (s, 1 H)<br><br>^{13}C-NMR (126 MHz, CHLOROFORM-*d*) δ ppm 55.36, 96.81, 113.03, 116.45, 120.36, 120.92, 121.80, 123.44, 124.14, 126.28, 130.04, 135.01, 136.01, 141.37, 156.90, 160.02, 175.94, 193.91	364.0269	364.0275	Yellow	149–150	-	24.16
**75**	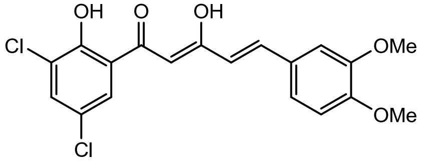	^{1}H-NMR (500 MHz, CHLOROFORM-*d*) δ ppm 3.94 (br. s., 6 H) 6.22 (br. s., 1 H) 6.47 (d, *J* = 15.43 Hz, 1 H) 6.90 (d, *J* = 8.15 Hz, 1 H) 7.08 (br. s., 1 H) 7.16 (d, *J* = 7.57 Hz, 1 H) 7.49–7.61 (m, 2 H) 7.66 (d, *J* = 14.85 Hz, 1 H) 12.87 (br. s., 1 H) 14.49 (br. s., 1 H)<br><br>^{13}C-NMR (126 MHz, CHLOROFORM-*d*) δ ppm 55.93, 56.05, 96.24, 109.66, 111.20, 119.21, 120.47, 123.10, 123.35, 124.08, 126.17, 127.68, 134.78, 141.57, 149.37, 151.54, 156.83, 176.60, 193.38	394.0375	394.0381	Orange	204–205	-	28.11

**76**	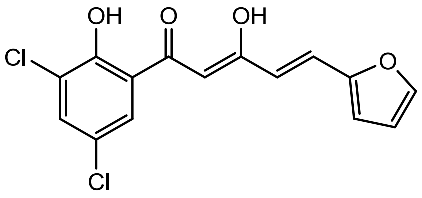	^{1}H-NMR (500 MHz, CHLOROFORM-*d*) δ ppm 6.22 (s, 1 H) 6.47–6.54 (m, 2 H) 6.68 (d, *J* = 3.49 Hz, 1 H) 7.46 (d, *J* = 15.43 Hz, 1 H) 7.50–7.56 (m, 2 H) 7.57 (d, *J* = 2.33 Hz, 1 H) 12.85 (s, 1 H) 14.38 (s, 1 H)<br><br>^{13}C-NMR (126 MHz, CHLOROFORM-*d*) δ ppm 96.75, 112.81, 115.74, 119.31, 120.44, 123.41, 124.08, 126.26, 127.51, 134.89, 145.35, 151.44, 156.87, 175.92, 193.55	323.9956	323.9960	Orange	113–114	-	15.19
**77**	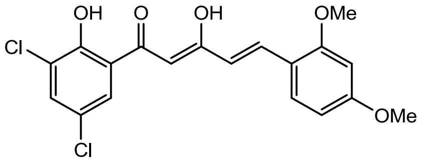	^{1}H-NMR (500 MHz, CHLOROFORM-*d*) δ ppm 3.87 (s, 3 H) 3.92 (s, 3 H) 6.19 (s, 1 H) 6.48 (d, *J* = 2.04 Hz, 1 H) 6.55 (dd, *J* = 8.44, 2.04 Hz, 1 H) 6.65 (d, *J* = 16.02 Hz, 1 H) 7.48 (d, *J* = 8.44 Hz, 1 H) 7.51 (d, *J* = 2.33 Hz, 1 H) 7.59 (d, *J* = 2.33 Hz, 1 H) 7.95 (d, *J* = 15.73 Hz, 1 H) 12.97 (s, 1 H) 14.60 (br. s., 1 H)<br><br>^{13}C-NMR (126 MHz, CHLOROFORM-*d*) δ ppm 55.53, 55.56, 95.96, 98.53, 105.64, 116.93, 119.48, 120.63, 123.24, 123.88, 126.16, 130.96, 134.54, 137.51, 156.78, 160.29, 163.22, 177.88, 195.44	394.0375	394.0379	Orange	153–154	-	18.21
**78**	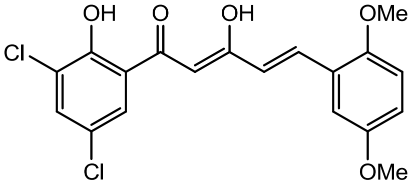	^{1}H-NMR (500 MHz, CHLOROFORM-*d*) δ ppm 3.82 (s, 3 H) 3.89 (s, 3 H) 6.23 (s, 1 H) 6.70 (d, *J* = 16.02 Hz, 1 H) 6.85–6.95 (m, 2 H) 7.06 (d, *J* = 2.91 Hz, 1 H) 7.52 (d, *J* = 2.33 Hz, 1 H) 7.58 (d, *J* = 2.04 Hz, 1 H) 7.98 (d, *J* = 16.02 Hz, 1 H) 12.87 (s, 1 H) 14.46 (br. s., 1 H)<br><br>^{13}C-NMR (126 MHz, CHLOROFORM-*d*) δ ppm 55.82, 56.10, 96.60, 112.49, 113.36, 117.60, 120.47, 122.29, 123.34, 124.05, 124.10, 126.24, 134.81, 136.94, 153.16, 153.59, 156.85, 176.84, 193.56	394.0375	394.0379	Orange	161–162	-	21.76

**79**	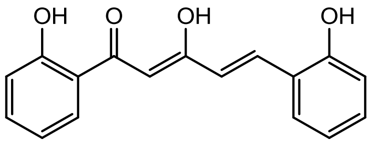	^{1}H-NMR (500 MHz, acetone) δ ppm 6.72 (s, 1 H) 6.91–7.04 (m, 5 H) 7.24–7.29 (m, 1 H) 7.51–7.55 (m, 1 H) 7.62–7.67 (m, 1 H) 7.94–7.99 (m, 1 H) 8.05 (d, *J* = 16.02 Hz, 1 H) 9.20 (s, 1 H) 12.15 (s, 1 H) 14.75 (br. s., 1 H)<br><br>^{13}C-NMR (126 MHz, acetone) δ ppm 96.91, 116.25, 118.28, 119.05, 119.14, 120.08, 121.99, 122.08, 128.63, 129.07, 131.47, 135.61, 135.88, 156.63, 162.46, 176.09, 196.00	282.0892	282.0893	Orange	163–164	-	46.36
**80**	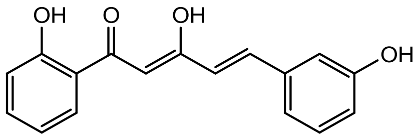	^{1}H-NMR (500 MHz, acetone) δ ppm 6.75 (s, 1 H) 6.87 (d, *J* = 15.73 Hz, 1 H) 6.91–6.94 (m, 1 H) 6.95–7.00 (m, 2 H) 7.14–7.20 (m, 2 H) 7.25–7.31 (m, 1 H) 7.51–7.56 (m, 1 H) 7.63 (d, *J* = 16.02 Hz, 1 H) 7.94 (dd, *J* = 8.30, 1.31 Hz, 1 H) 8.60 (s, 1 H) 12.09 (s, 1 H) 14.64 (br. s., 1 H)<br><br>^{13}C-NMR (126 MHz, acetone) δ ppm 97.25, 114.44, 117.40, 118.33, 119.00, 119.19, 119.65, 122.38, 129.09, 130.03, 136.06, 136.54, 139.81, 157.89, 162.50, 175.09, 196.25	282.0892	282.0894	Orange	149–150	-	44.51
**81**	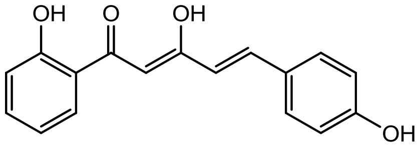	^{1}H-NMR (500 MHz, acetone) δ ppm 6.68 (s, 1 H) 6.74 (d, *J* = 15.73 Hz, 1 H) 6.89–6.99 (m, 4 H) 7.49–7.54 (m, 1 H) 7.59 (d, *J* = 8.74 Hz, 2 H) 7.66 (d, *J* = 16.02 Hz, 1 H) 7.92 (dd, *J* = 8.30, 1.31 Hz, 1 H) 9.16 (br. s., 1 H) 12.15 (br. s., 1 H) 14.79 (br. s., 1 H)<br><br>^{13}C-NMR (126 MHz, acetone) δ ppm 97.91, 101.57, 101.62, 121.25, 124.15, 124.26, 131.91, 134.16, 135.33, 140.98, 145.45, 165.11, 167.59, 181.43, 193.15	282.0892	282.0893	Orange	149–150	164-165	46.14

**82**	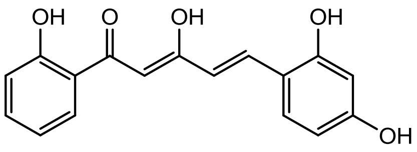	^{1}H-NMR (500 MHz, acetone) δ ppm 6.47 (d, J = 8.45 Hz, 1 H) 6.51 (s, 1 H) 6.62 (s, 1 H) 6.83 (d, J = 16.02 Hz, 1 H) 6.92–6.97 (m, 2 H) 7.47–7.53 (m, 2 H) 7.94 (d, J = 8.45 Hz, 1 H) 8.00 (d, J = 16.02 Hz, 1 H) 9.15 (br. s., 2 H) 12.23 (br. s., 1 H) 14.88 (br.s., 1 H)<br><br>^{13}C-NMR (126 MHz, acetone) δ ppm 95.94, 102.78, 108.38, 114.42, 118.21, 118.30, 119.03, 119.11, 128.88, 130.38, 135.53, 136.42, 158.57, 161.12, 162.36, 177.29, 195.23	298.0841	298.0845	Brown	90–92	-	38.11
**83**	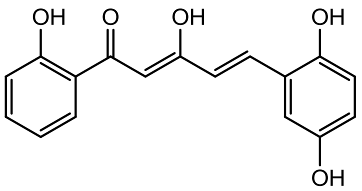	^{1}H-NMR (500 MHz, acetone) δ ppm 6.70 (s, 1 H) 6.78–6.82 (m, 1 H) 6.83–6.86 (m, 1 H) 6.91 (d, J = 16.02 Hz, 1 H) 6.94–6.98 (m, 2 H) 7.10 (d, J = 2.62 Hz, 1 H) 7.49–7.55 (m, 1 H) 7.95 (dd, J = 8.30, 1.31 Hz, 1 H) 7.98–8.05 (m, 2 H) 8.64 (br. s., 1 H) 12.16 (s, 1 H) 14.74 (br. s., 1 H)<br><br>^{13}C-NMR (126 MHz, acetone) δ ppm 96.90, 113.49, 117.05, 118.27, 119.05, 119.10, 119.13, 121.70, 122.40, 129.06, 135.65, 135.86, 150.07, 150.60, 162.45, 176.09, 195.93	298.0841	298.0845	Brown	173–174	-	36.12
**84**	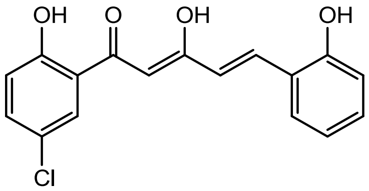	^{1}H-NMR (500 MHz, acetone) δ ppm 6.79 (s, 1 H) 6.93 (t, J = 7.57 Hz, 1 H) 6.99 (dd, J = 8.59, 3.35 Hz, 2 H) 7.04 (d, J = 16.02 Hz, 1 H) 7.27 (t, J = 7.72 Hz, 1 H) 7.51 (dd, J = 8.88, 2.18 Hz, 1 H) 7.64 (d, J = 7.57 Hz, 1 H) 7.97 (d, J = 2.62 Hz, 1 H) 8.07 (d, J = 16.02 Hz, 1 H) 9.25 (br. s., 1 H) 12.13 (s, 1 H) 14.68 (br. s., 1 H)<br><br>^{13}C-NMR (126 MHz, acetone) δ ppm 97.04, 116.27, 120.06, 120.09, 120.13, 121.84, 122.00, 123.45, 128.14, 128.73, 131.66, 135.34, 136.28, 156.79, 160.94, 177.03, 194.40	316.0502	316.0510	Orange	166–167	-	40.59

**85**	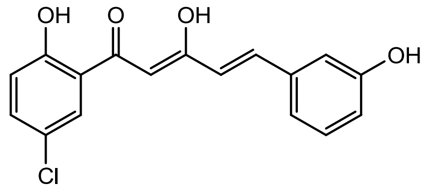	^{1}H-NMR (500 MHz, acetone) δ ppm 6.82 (s, 1 H) 6.87–6.95 (m, 2 H) 7.00 (d, *J* = 9.03 Hz, 1 H) 7.14–7.21 (m, 2 H) 7.25–7.32 (m, 1 H) 7.52 (dd, *J* = 8.88, 2.48 Hz, 1 H) 7.66 (d, *J* = 16.02 Hz, 1 H) 7.94 (d, *J* = 2.62 Hz, 1 H) 8.62 (s, 1 H) 12.06 (s, 1 H) 14.57 (br. s., 1 H)<br><br>^{13}C-NMR (126 MHz, acetone) δ ppm 97.36, 114.51, 117.56, 119.71, 120.00, 120.19, 122.21, 123.48, 128.15, 130.05, 135.51, 136.45, 140.45, 157.91, 160.97, 176.02, 194.70	316.0502	316.0504	Yellow	181–182	-	45.12
**86**	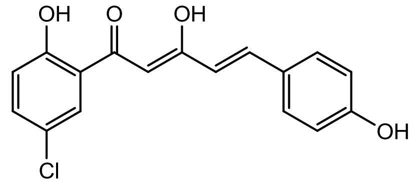	^{1}H-NMR (500 MHz, acetone) δ ppm 6.74 (s, 1 H) 6.77 (d, *J* = 16.02 Hz, 1 H) 6.93 (d, *J* = 8.74 Hz, 2 H) 6.98 (d, *J* = 8.74 Hz, 1 H) 7.50 (dd, *J* = 9.03, 2.62 Hz, 1 H) 7.60 (d, *J* = 8.74 Hz, 2 H) 7.70 (d, *J* = 16.02 Hz, 1 H) 7.92 (d, *J* = 2.62 Hz, 1 H) 9.04 (s, 1 H) 12.13 (s, 1 H) 14.73 (br. s., 1 H)<br><br>^{13}C-NMR (126 MHz, acetone) δ ppm 96.49, 116.03, 118.83, 120.13, 123.40, 126.68, 128.00, 130.27, 135.23, 140.88, 157.59, 160.00, 160.88, 177.14, 194.00	316.0502	316.0506	Yellow	183–184	-	42.67
**87**	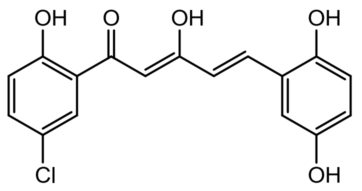	^{1}H-NMR (500 MHz, acetone) δ ppm 6.77 (s, 1 H) 6.78–6.87 (m, 2 H) 6.94 (d, *J* = 16.02 Hz, 1 H) 6.99 (d, *J* = 9.03 Hz, 1 H) 7.09 (d, *J* = 2.62 Hz, 1 H) 7.50 (dd, *J* = 8.88, 2.48 Hz, 1 H) 7.97 (d, *J* = 2.33 Hz, 1 H) 8.03 (d, *J* = 16.02 Hz, 1 H) 8.16 (br. s., 1 H) 8.82 (br. s., 1 H) 12.13 (br. s., 1 H) 14.64 (br.s., 1 H)<br><br>^{13}C-NMR (126 MHz, acetone) δ ppm 97.00, 113.53, 117.05, 119.35, 120.11, 121.48, 122.26, 123.45, 128.12, 128.14, 135.29, 136.42, 150.27, 150.61, 160.87, 177.12, 194.24	332.0452	332.0457	Orange	205–206	-	25.27

**88**	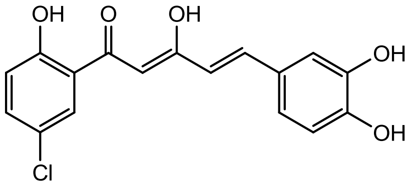	^{1}H-NMR (500 MHz, acetone) δ ppm 6.67–6.75 (m, 2 H) 6.91 (d, *J* = 8.15 Hz, 1 H) 6.98 (d, *J* = 9.03 Hz, 1 H) 7.11 (dd, *J* = 8.15, 2.04 Hz, 1 H) 7.22 (d, *J* = 2.04 Hz, 1 H) 7.50 (dd, *J* = 8.88, 2.48 Hz, 1 H) 7.63 (d, *J* = 15.73 Hz, 1 H) 7.92 (d, *J* = 2.33 Hz, 1 H) 8.41 (br. s., 2 H) 12.15 (br. s., 1 H) 14.72 (br. s., 1 H)<br><br>^{13}C-NMR (126 MHz, acetone) δ ppm 96.46, 114.42, 115.65, 118.89, 120.06, 120.12, 122.07, 123.39, 127.38, 128.00, 135.22, 141.26, 145.59, 148.22, 160.89, 177.12, 193.99	332.0452	332.0462	Orange	177–178	-	23.55
**89**	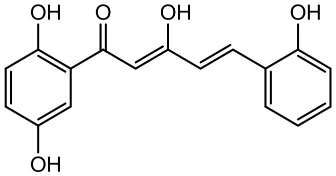	^{1}H-NMR (500 MHz, acetone) δ ppm 6.60 (s, 1 H) 6.83 (d, *J* = 9.03 Hz, 1 H) 6.92 (t, *J* = 7.57 Hz, 1 H) 6.97–7.03 (m, 2 H) 7.09 (dd, *J* = 8.88, 2.77 Hz, 1 H) 7.22–7.30 (m, 1 H) 7.34 (d, *J* = 2.62 Hz, 1 H) 7.65 (d, *J* = 7.57 Hz, 1 H) 8.04 (d, *J* = 16.02 Hz, 1 H) 8.09–8.33 (br. s., 1 H) 9.16 (br. s., 1 H) 11.55 (br. s., 1 H) 14.85 (br. s., 1 H)<br><br>^{13}C-NMR (126 MHz, acetone) δ ppm 96.99, 113.41, 116.23, 118.87, 118.93, 120.06, 122.02, 122.11, 124.33, 128.50, 131.43, 135.38, 149.55, 155.79, 156.63, 177.10, 195.61	298.0841	298.0849	Orange	196–197	-	29.44

**90**	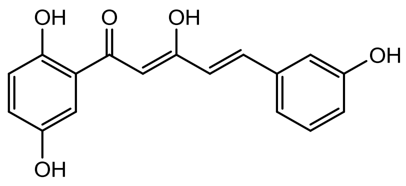	^{1}H-NMR (500 MHz, acetone) δ ppm 6.64 (s, 1 H) 6.83 (d, *J* = 8.74 Hz, 1 H) 6.87 (d, *J* = 16.02 Hz, 1 H) 6.91 (dd, *J* = 8.01, 2.18 Hz, 1 H) 7.09 (dd, *J* = 8.88, 2.77 Hz, 1 H) 7.14–7.19 (m, 2 H) 7.24–7.29 (m, 1 H) 7.31 (d, *J* = 2.91 Hz, 1 H) 7.61 (d, *J* = 16.02 Hz, 1 H) 8.39 (br. s., 1 H) 8.82 (br. s., 1 H) 11.49 (s, 1 H) 14.78 (br. s, 1 H)<br><br>^{13}C-NMR (126 MHz, acetone) δ ppm 97.39, 113.42, 114.42, 117.38, 118.90, 119.56, 122.42, 124.54, 130.00, 136.53, 139.68, 149.66, 155.74, 157.96, 175.08, 195.84	298.0841	298.0848	Brown	179–180	-	27.16
**91**	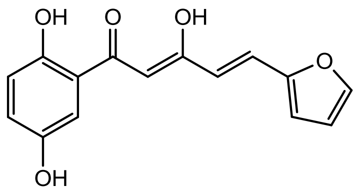	^{1}H-NMR (500 MHz, acetone) δ ppm 6.62–6.65 (m, 2 H) 6.67 (d, *J* = 15.73 Hz, 1 H) 6.83 (d, *J* = 8.74 Hz, 1 H) 6.88 (d, *J* = 3.20 Hz, 1 H) 7.10 (dd, *J* = 8.88, 2.77 Hz, 1 H) 7.34 (d, *J* = 2.91 Hz, 1 H) 7.49 (d, *J* = 15.73 Hz, 1 H) 7.76 (s, 1 H) 8.15 (s, 1 H) 11.51 (s, 1 H) 14.75 (br. s., 1 H)<br><br>^{13}C-NMR (126 MHz, acetone) δ ppm 97.35, 112.75, 113.50, 115.25, 118.87, 118.90, 119.82, 124.49, 126.08, 145.53, 149.57, 151.68, 155.85, 174.80, 195.68	272.0685	272.0690	Brown	142–143	-	27.35
**92**	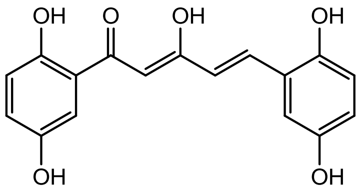	^{1}H-NMR (500 MHz, acetone) δ ppm 6.59 (s, 1 H) 6.76–6.86 (m, 3 H) 6.91 (d, *J* = 16.02 Hz, 1 H) 7.06–7.11 (m, 2 H) 7.33 (d, *J* = 2.91 Hz, 1 H) 7.97 (br. s., 1 H) 8.01 (d, *J* = 16.02 Hz, 1 H) 8.13 (br. s., 1 H) 8.58 (br. s., 1 H) 11.55 (s, 1 H) 14.85 (br. s., 1 H)<br><br>^{13}C-NMR (126 MHz, acetone) δ ppm 96.96, 113.39, 113.41, 117.03, 118.87, 118.94, 119.04, 121.73, 122.43, 124.31, 135.42, 149.53, 150.02, 150.60, 155.79, 181.81, 195.58	314.079	314.084	Orange	195–196	-	14.79

**93**	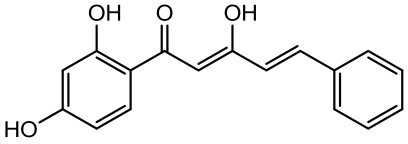	^{1}H-NMR (500 MHz, acetone) δ ppm 6.37 (d, J = 2.04 Hz, 1 H) 6.47 (dd, J = 8.88, 1.89 Hz, 1 H) 6.59 (s, 1 H) 6.89 (d, J = 16.02 Hz, 1 H) 7.40–7.48 (m, 3 H) 7.62 (d, J = 15.73 Hz, 1 H) 7.68 (d, J = 7.57 Hz, 2 H) 7.81 (d, J = 8.74 Hz, 1 H) 9.68 (br. s., 1 H) 12.50 (br. s., 1 H) 14.48 (br. s., 1 H)<br><br>^{13}C-NMR (126 MHz, acetone) δ ppm 98.85, 102.19, 108.38, 113.66, 117.21, 127.96, 133.10, 134.16, 135.05, 136.48, 136.54, 140.54, 143.67, 170.15, 170.71, 178.38, 200.26	282.0892	282.0894	Yellow	153–154	-	30.67
**94**	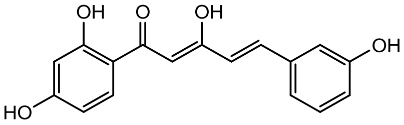	^{1}H-NMR (500 MHz, acetone) δ ppm 6.37 (d, J = 2.04 Hz, 1 H) 6.47 (dd, J = 8.74, 2.04 Hz, 1 H) 6.59 (s, 1 H) 6.82 (d, J = 16.02 Hz, 1 H) 6.90 (dd, J = 8.15, 2.33 Hz, 1 H) 7.11–7.17 (m, 2 H) 7.23–7.29 (m, 1 H) 7.55 (d, J = 16.02 Hz, 1 H) 7.82 (d, J = 8.74 Hz, 1 H) 8.61 (br. s., 1 H) 9.48 (br. s., 1 H) 12.50 (br. s., 1 H) 14.48 (br. s., 1 H)<br><br>^{13}C-NMR (126 MHz, acetone) δ ppm 96.90, 103.18, 108.38, 112.07, 114.28, 117.11, 119.45, 122.61, 129.98, 131.31, 136.71, 138.63, 157.87, 164.80, 165.50, 173.25, 195.05	298.0841	298.0842	Yellow	224–225	-	31.12
**95**	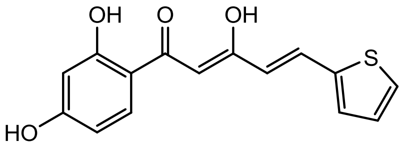	^{1}H-NMR (500 MHz, acetone) δ ppm 6.37 (d, J = 2.33 Hz, 1 H) 6.47 (dd, J = 8.88, 2.48 Hz, 1 H) 6.56–6.62 (m, 2 H) 7.13–7.17 (m, 1 H) 7.44 (d, J = 3.49 Hz, 1 H) 7.62 (d, J = 5.24 Hz, 1 H) 7.77 (d, J = 15.73 Hz, 1 H) 7.83 (d, J = 9.03 Hz, 1 H) 9.49 (br. s., 1 H) 12.51 (s, 1 H) 14.47 (br. s., 1 H)<br><br>^{13}C-NMR (126 MHz, acetone) δ ppm 96.75, 103.14, 108.35, 112.10, 121.36, 128.43, 128.66, 130.93, 131.35, 131.41, 140.59, 164.74, 165.48, 172.88, 194.88	288.0456	288.0458	Orange	167–168	-	40.85

**96**	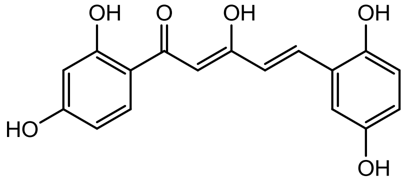	^{1}H-NMR (500 MHz, acetone) δ ppm 6.34–6.38 (m, 1 H) 6.46 (dd, *J* = 8.88, 2.18 Hz, 1 H) 6.53 (s, 1 H) 6.76–6.80 (m, 1 H) 6.81–6.88 (m, 2 H) 7.08 (d, *J* = 2.62 Hz, 1 H) 7.83 (d, *J* = 8.74 Hz, 1 H) 7.93 (d, *J* = 16.02 Hz, 1 H) 8.62 (br. s., 3 H) 12.56 (br. s., 1 H) 14.47 (br. s., 1 H)<br><br>^{13}C-NMR (126 MHz, acetone) δ ppm 96.49, 103.16, 108.31, 112.08, 113.36, 117.00, 118.71, 121.96, 122.58, 131.26, 134.45, 149.86, 150.58, 164.65, 165.41, 174.22, 194.84	314.0790	314.0792	Orange	190–191	-	16.28
**97**	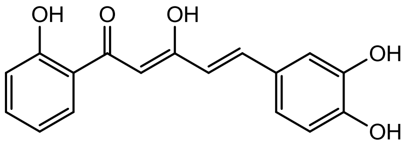	^{1}H-NMR (500 MHz, acetone) δ ppm 6.65–6.73 (m, 2 H) 6.88–7.00 (m, 3 H) 7.11 (d, *J* = 8.2 Hz, 1 H) 7.23 (s, 1 H) 7.52 (t, *J* = 7.86 Hz, 1 H) 7.61 (d, *J* = 15.73 Hz, 1 H) 7.92 (d, *J* = 8.15 Hz, 1 H) 8.28 (br. s, 1 H) 8.61 (br. s, 1 H) 12.19 (s, 1 H) 14.81 (br. s., 1 H)<br><br>^{13}C-NMR (126 MHz, acetone) δ ppm 96.39, 114.34, 115.64, 118.28, 119.03, 119.07, 119.12, 121.92, 127.45, 128.94, 135.79, 140.57, 145.57, 148.04, 162.41, 176.16, 195.62, 205.44,	298.0841	298.0843	Orange	155–156	NR	21.33

Notes: MP = Melting point; NR = Not reported.

Table S2. NO Suppression activity and cytotoxicity of compounds **1–97** on RAW 264.7 Cells.

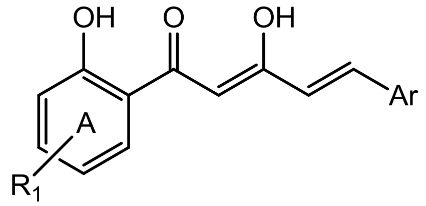

Compounds	R1 (Ring A)	Ar (Ring B)	NO inhibition at 50 µM (%) ± S.E.M	NO inhibition IC50 (µM) ± S.E.M	Cytotoxicity IC50 (µM) ± S.E.M
**Curcumin**	-	-	99.33 ± 0.20	14.69 ± 0.240	28.81 ± 0.770
**1**	H	phenyl	94.74 ± 1.20	22.59 ± 0.515	56.20 ± 1.134
**2**	H	2-chlorophenyl	91.00 ± 2.86	27.32 ± 0.234	>100
**3**	H	3-chlorophenyl	92.95 ± 3.43	26.66 ± 0.732	>100
**4**	H	3-bromophenyl	88.41 ± 3.21	29.40 ± 0.746	>100
**5**	H	2-methoxyphenyl	91.84 ± 1.49	25.67 ± 0.621	>100
**6**	H	3-methoxyphenyl	81.59 ± 3.97	31.59 ± 0.632	>100
**7**	H	4-methoxyphenyl	36.58 ± 2.38	ND	ND
**8**	H	3.4-dimethoxyphenyl	94.69 ± 1.70	24.57 ± 0.730	>100
**9**	H	3,4,5-trimethoxyphenyl	96.61 ± 1.43	16.63 ± 1.136	>100
**10**	H	3-nitrophenyl	39.87 ± 3.81	ND	ND
**11**	H	4-nitrophenyl	40.41 ± 7.10	ND	ND
**12**	H	Furan-2-yl	82.02 ± 3.18	48.95 ± 1.048	>100
**13**	H	2,4-dimethoxyphenyl	47.14 ± 2.96	ND	ND
**14**	H	2,5-dimethoxyphenyl	92.90 ± 0.18	28.38 ± 0.435	>100
**15**	H	3,4-dichlorophenyl	43.52 ± 2.72	ND	ND
**16**	H	Thiophene-2-yl	79.08 ± 4.58	42.64 ± 0.128	>100
**17**	H	5-chlorothiophene-2-yl	63.80 ± 2.57	36.42 ± 1.804	67.85 ± 2.867
**18**	H	5-methylthiophene-2yl	58.91 ± 5.76	39.59 ± 4.129	>100
**19**	H	naphthalen-1-yl	85.78 ± 3.93	22.96 ± 1.701	>100

Table S2. *Cont.*

Compounds	R1 (Ring A)	Ar (Ring B)	NO inhibition at 50 µM (%) ± S.E.M	NO inhibition IC50 (µM) ± S.E.M	Cytotoxicity IC50 (µM) ± S.E.M
**20**	H	naphthalen-2-yl	6.00 ± 1.19	ND	ND
**21**	5-Cl	phenyl	85.27 ± 6.76	47.63 ± 1.137	>100
**22**	5-Cl	2-chlorophenyl	82.49 ± 4.32	29.96 ± 2.100	>100
**23**	5-Cl	3-chlorophenyl	66.61 ± 4.11	36.16 ± 0.922	68.40 ± 2.188
**24**	5-Cl	2-methoxyphenyl	70.42 ± 1.32	36.02 ± 0.515	>100
**25**	5-Cl	3-methoxyphenyl	76.26 ± 6.48	17.37 ± 0.372	>100
**26**	5-Cl	4-methoxyphenyl	39.38 ± 3.95	ND	ND
**27**	5-Cl	3.4-dimethoxyphenyl	37.63 ± 11.67	ND	ND
**28**	5-Cl	3,4,5-trimethoxyphenyl	89.50 ± 5.87	13.64 ± 0.532	>100
**29**	5-Cl	3-nitrophenyl	12.69 ± 3.32	ND	ND
**30**	5-Cl	4-nitrophenyl	7.46 ± 2.89	ND	ND
**31**	5-Cl	Furan-2-yl	36.75 ± 7.40	ND	ND
**32**	5-Cl	2,4-dimethoxyphenyl	14.80 ± 3.65	ND	ND
**33**	5-Cl	2,5-dimethoxyphenyl	43.22 ± 1.78	ND	ND
**34**	5-Cl	3,4-dichlorophenyl	18.22 ± 3.63	ND	ND
**35**	5-Cl	Thiophene-2-yl	68.84 ± 4.21	32.17 ± 0.176	>100
**36**	5-Cl	5-chlorothiophene-2-yl	71.55 ± 3.20	25.32 ± 1.725	>100
**37**	5-Cl	5-methylthiophene-2yl	56.04 ± 2.62	62.44 ± 2.287	>100
**38**	5-Cl	5-methylfuran-2-yl	73.81 ± 1.14	25.49 ± 1.031	>100
**39**	5-Cl	naphthalen-1-yl	8.72 ± 3.92	ND	ND
**40**	5-Cl	naphthalen-2-yl	43.63 ± 0.41	ND	ND
**41**	5-Br	phenyl	79.90 ± 8.68	20.83 ± 0.540	>100
**42**	5-Br	2-chlorophenyl	33.69 ± 4.29	ND	ND
**43**	5-Br	3-chlorophenyl	81.18 ± 5.16	19.81 ± 0.907	>100
**44**	5-Br	3.4-dimethoxyphenyl	40.93 ± 5.54	ND	ND
**45**	4-OMe	phenyl	87.90 ± 5.13	29.54 ± 0.469	>100

Table S2. *Cont.*

Compounds	R1 (Ring A)	Ar (Ring B)	NO inhibition at 50 µM (%) ± S.E.M	NO inhibition IC50 (µM) ± S.E.M	Cytotoxicity IC50 (µM) ± S.E.M
**46**	4-OMe	2-chlorophenyl	76.18 ± 4.57	58.52 ± 2.452	>100
**47**	4-OMe	3-chlorophenyl	33.87 ± 4.71	ND	ND
**48**	4-OMe	2-methoxyphenyl	66.32 ± 6.75	39.04 ± 1.238	88.55 ± 2.544
**49**	4-OMe	3-methoxyphenyl	57.45 ± 6.45	45.75 ± 0.804	>100
**50**	4-OMe	4-methoxyphenyl	15.96 ± 6.58	ND	ND
**51**	4-OMe	3.4-dimethoxyphenyl	58.92 ± 2.76	21.70 ± 0.491	>100
**52**	4-OMe	3,4,5-trimethoxyphenyl	96.39 ± 0.37	29.31 ± 0.306	>100
**53**	4-OMe	Furan-2-yl	18.83 ± 6.68	ND	ND
**54**	4-OMe	2,4-dimethoxyphenyl	33.53 ± 4.21	ND	ND
**55**	4-OMe	2,5-dimethoxyphenyl	32.83 ± 4.76	ND	ND
**56**	4-OMe	Thiophene-2-yl	38.25 ± 5.12	ND	ND
**57**	4,6-OMe	4-methoxyphenyl	34.84 ± 10.51	ND	ND
**58**	5-OMe	phenyl	92.84 ± 1.80	25.92 ± 0.325	>100
**59**	5-OMe	2-chlorophenyl	89.14 ± 3.56	35.72 ± 0.789	>100
**60**	5-OMe	4-chlorophenyl	29.45 ± 4.32	ND	ND
**61**	5-OMe	2-methoxyphenyl	65.78 ± 2.37	27.41 ± 0.207	>100
**62**	5-OMe	3-methoxyphenyl	92.62 ± 1.18	35.06 ± 0.165	>100
**63**	5-OMe	3.4-dimethoxyphenyl	92.19 ± 0.77	19.79 ± 0.365	>100
**64**	5-OMe	3,4,5-trimethoxyphenyl	95.16 ± 1.09	18.39 ± 0.246	>100
**65**	5-OMe	3-nitrophenyl	1.55 ± 1.14	ND	ND
**66**	5-OMe	4-nitrophenyl	12.48 ± 1.92	ND	ND
**67**	5-OMe	Furan-2-yl	72.11 ± 3.07	71.50 ± 2.483	>100
**68**	5-OMe	2,4-dimethoxyphenyl	79.48 ± 4.71	33.44 ± 0.695	>100
**69**	5-OMe	2,5-dimethoxyphenyl	45.54 ± 3.25	ND	ND
**70**	5-OMe	3,4-dichlorophenyl	29.41 ± 5.89	ND	ND
**71**	5-OMe	Thiophene-2-yl	84.95 ± 2.66	35.63 ± 0.621	>100

Table S2. *Cont.*

Compounds	R1 (Ring A)	Ar (Ring B)	NO inhibition at 50 µM (%) ± S.E.M	NO inhibition IC50 (µM) ± S.E.M	Cytotoxicity IC50 (µM) ± S.E.M
**72**	3,5-Cl	phenyl	23.32 ± 5.00	ND	ND
**73**	3,5-Cl	2-methoxyphenyl	22.50 ± 4.81	ND	ND
**74**	3,5-Cl	3-methoxyphenyl	45.30 ± 7.85	ND	ND
**75**	3,5-Cl	3.4-dimethoxyphenyl	12.48 ± 0.74	ND	ND
**76**	3,5-Cl	Furan-2-yl	36.50 ± 2.34	ND	ND
**77**	3,5-Cl	2,4-dimethoxyphenyl	21.82 ± 10.07	ND	ND
**78**	3,5-Cl	2,5-dimethoxyphenyl	34.45 ± 4.73	ND	ND
**79**	H	2-hydroxyphenyl	95.46 ± 0.59	28.90 ± 1.542	72.63 ± 2.612
**80**	H	3-hydroxyphenyl	97.22 ± 0.69	30.81 ± 0.722	85.01 ± 1.442
**81**	H	4-hydroxyphenyl	91.45 ± 3.86	19.07 ± 0.644	94.13 ±0.917
**82**	H	2,4-dihydroxyphenyl	7.83 ± 2.03	ND	ND
**83**	H	2,5-dihydroxyphenyl	94.27 ± 1.83	16.74 ± 0.557	49.52 ± 1.661
**84**	5-Cl	2-hydroxyphenyl	95.20 ± 0.86	15.91 ± 0.947	40.87 ± 1.627
**85**	5-Cl	3-hydroxyphenyl	95.86 ± 0.45	26.93 ± 0.796	53.08 ± 0.973
**86**	5-Cl	4-hydroxyphenyl	90.29 ± 2.66	18.71 ± 0.786	89.12 ± 1.076
**87**	5-Cl	2,5-dihydroxyphenyl	96.47 ± 1.42	32.32 ± 0.865	66.02 ± 1.207
**88**	5-Cl	3,4-dihydroxyphenyl	99.67 ± 1.15	4.89 ± 0.312	56.37 ± 1.190
**89**	5-OH	2-hydroxyphenyl	83.61 ± 2.00	31.00 ± 1.980	67.30 ± 0.552
**90**	5-OH	3-hydroxyphenyl	90.94 ± 2.02	35.71 ± 0. 865	>100
**91**	5-OH	Furan-2-yl	83.56 ± 2.84	74.99 ± 4.900	>100
**92**	5-OH	2,5-dihydroxyphenyl	49.02 ± 12.49	ND	ND
**93**	4-OH	phenyl	94.71 ± 2.28	29.89 ± 0.444	59.46 ± 0.511
**94**	4-OH	3-hydroxyphenyl	95.38 ± 1.45	24.80 ± 0.355	70.13 ± 1.795
**95**	4-OH	Thiophene-2-yl	60.38 ± 2.04	44.84 ± 1.574	>100
**96**	4-OH	2,5-dihydroxyphenyl	4.07 ± 1.76	ND	ND
**97**	H	3,4-dihydroxyphenyl	98.89 ± 1.58	9.56 ± 0.468	>100

Figure S1. NMR spectra of compound **1**.

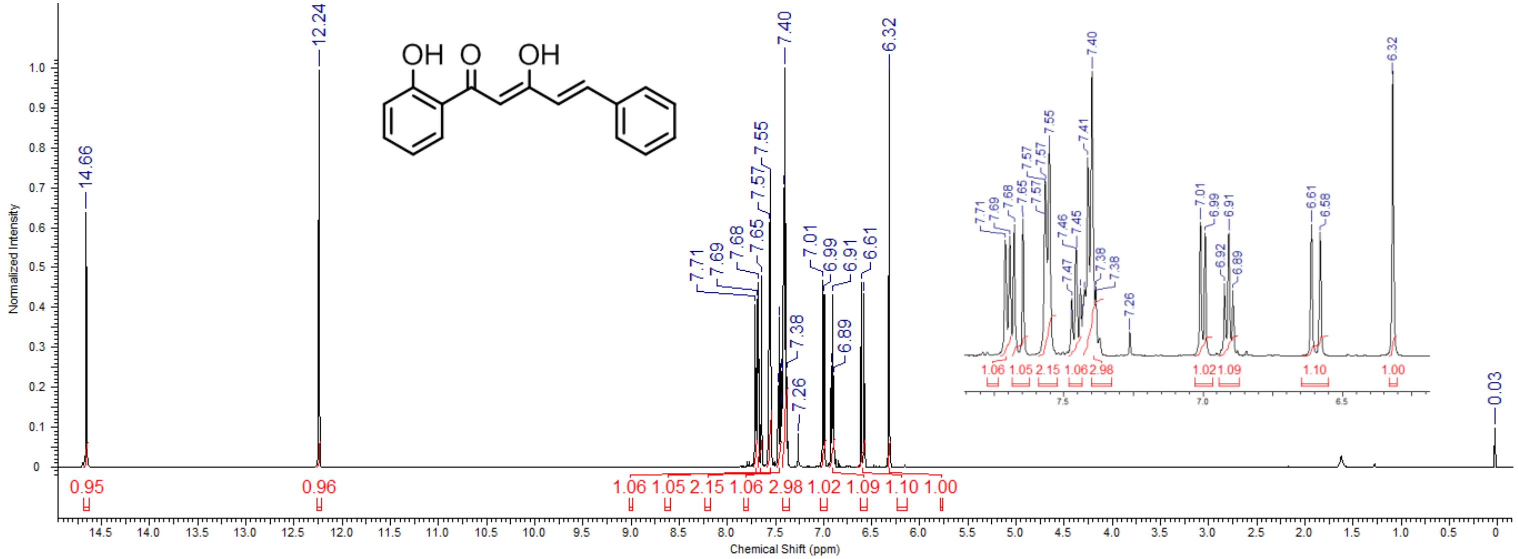

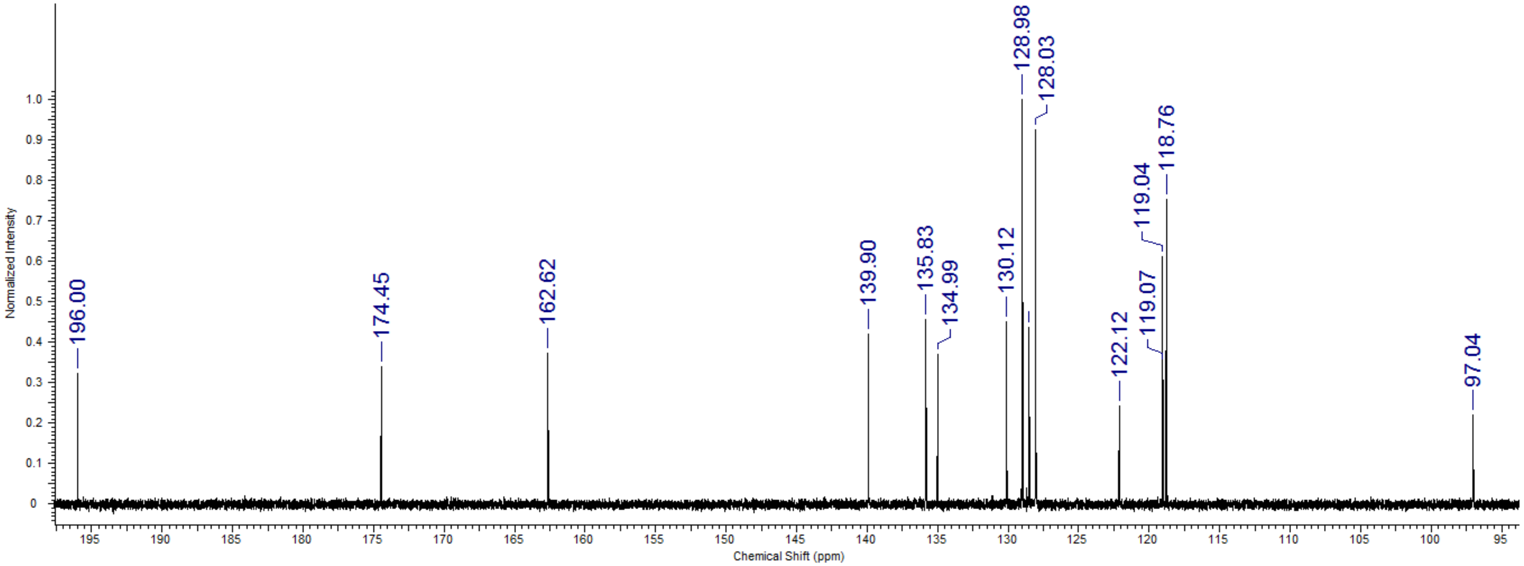

Figure S2. NMR spectra of compound **2**.

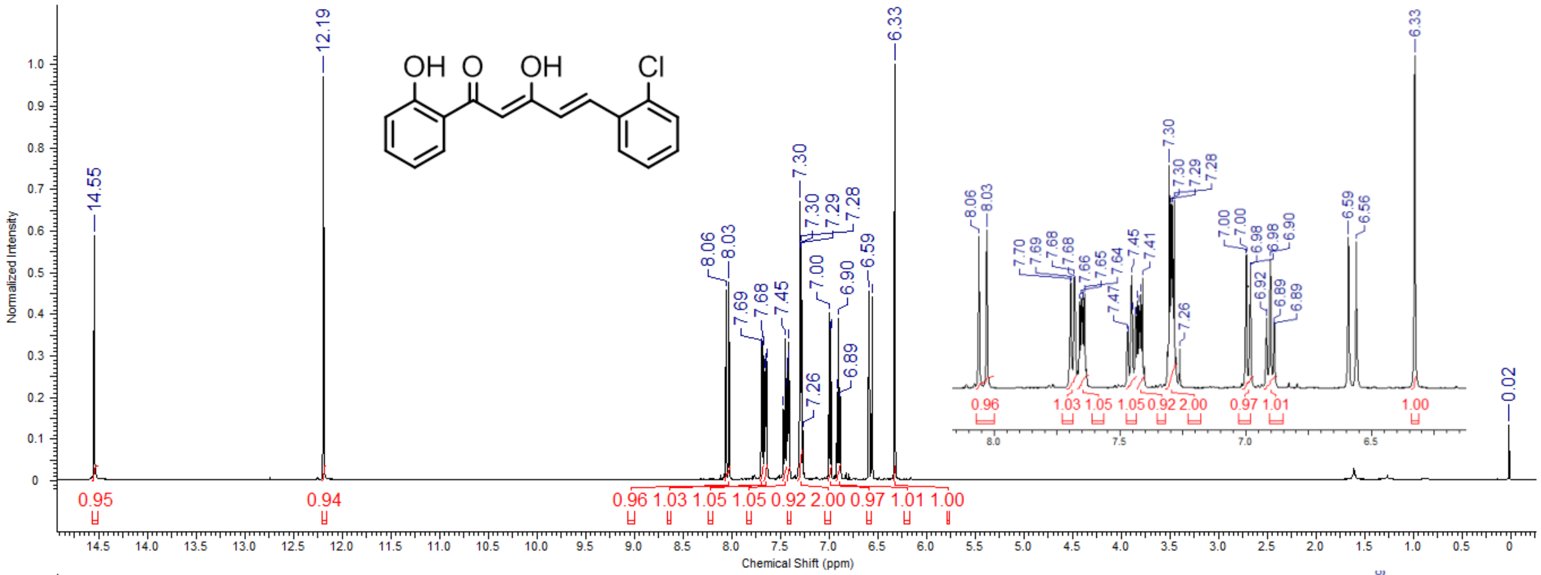

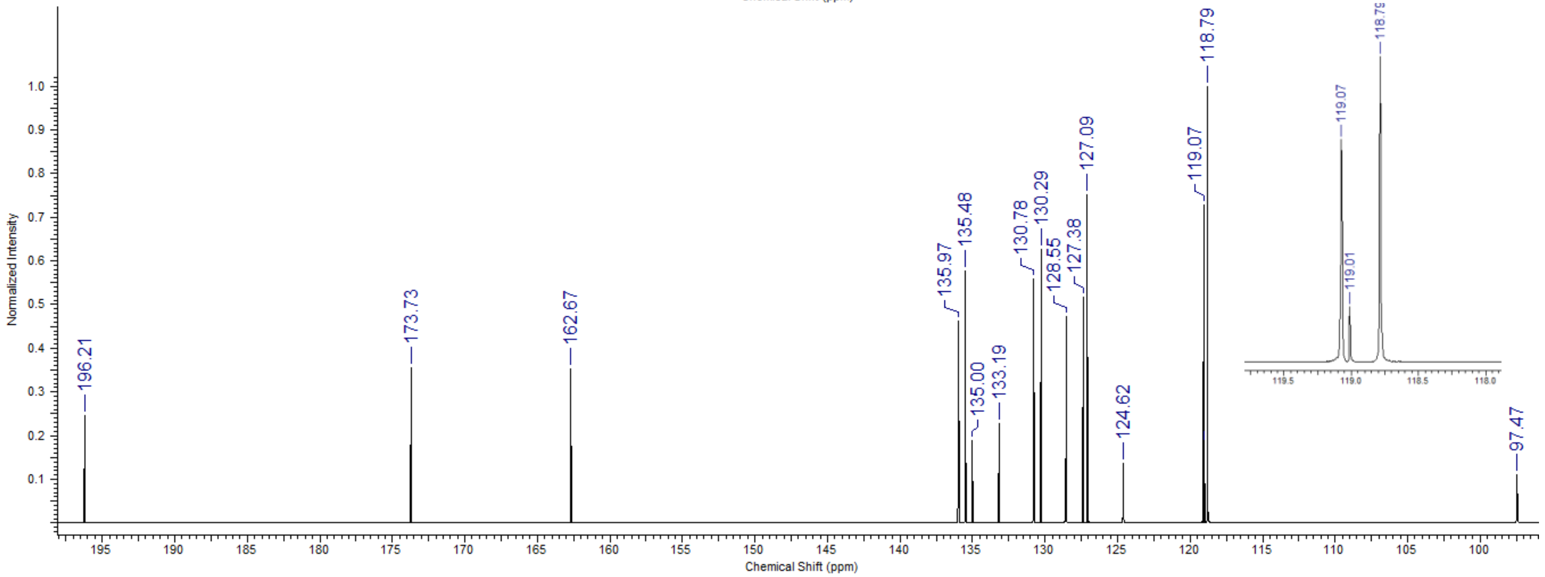

Figure S3. **N**MR spectra of compound **3**.

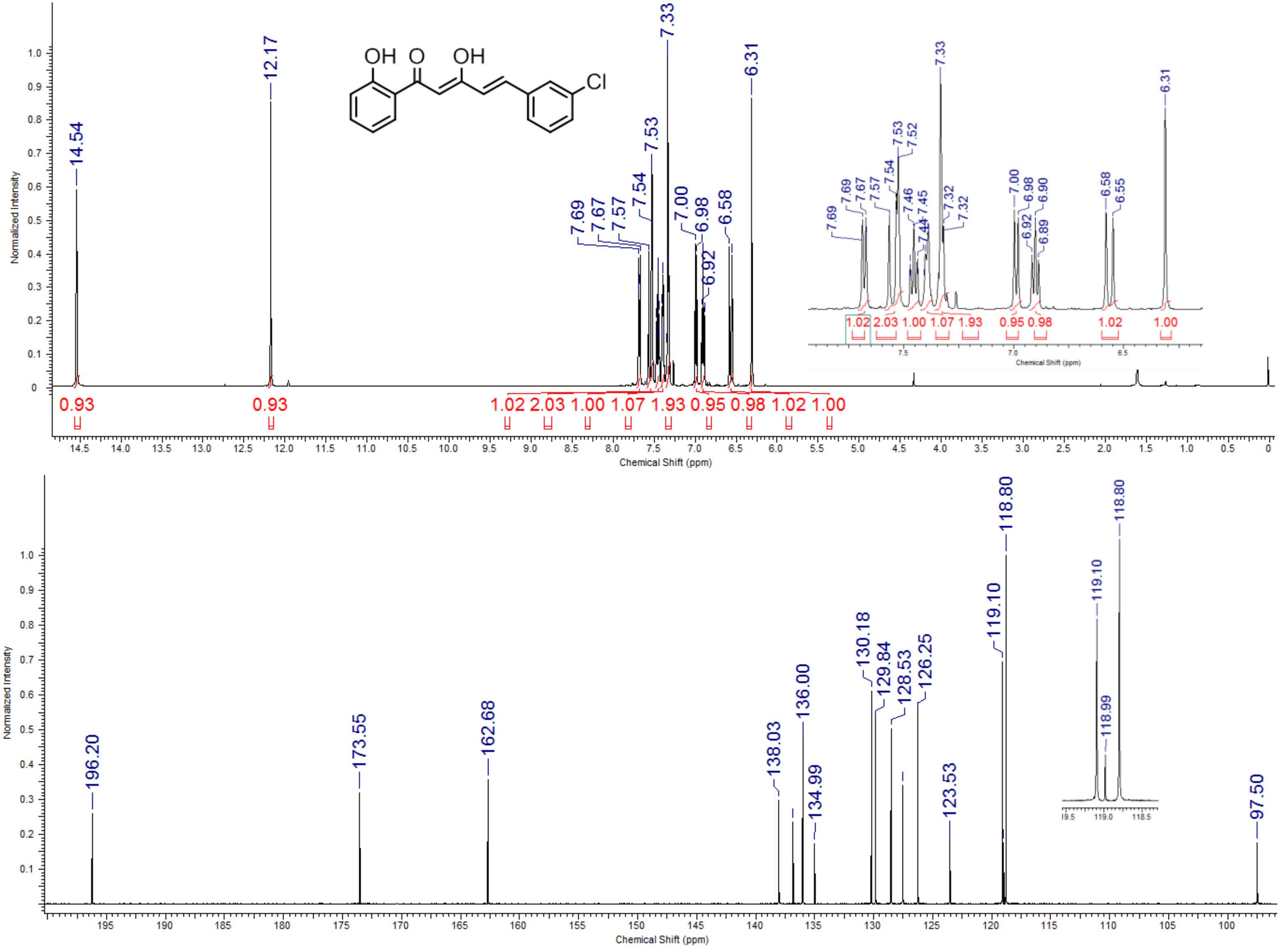

Figure S4. NMR spectra of compound **4**.

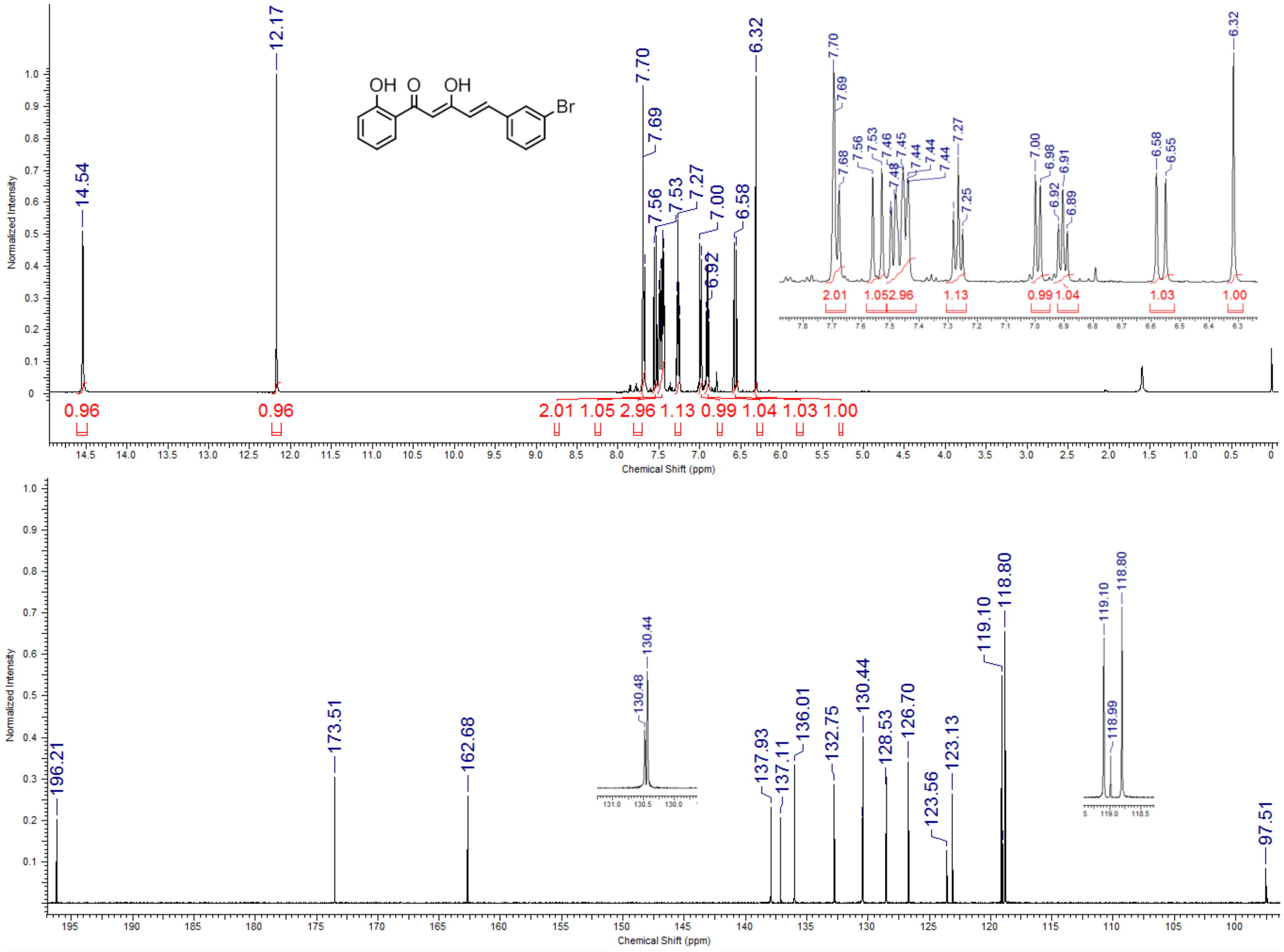

Figure S5. NMR spectra of compound **5**.

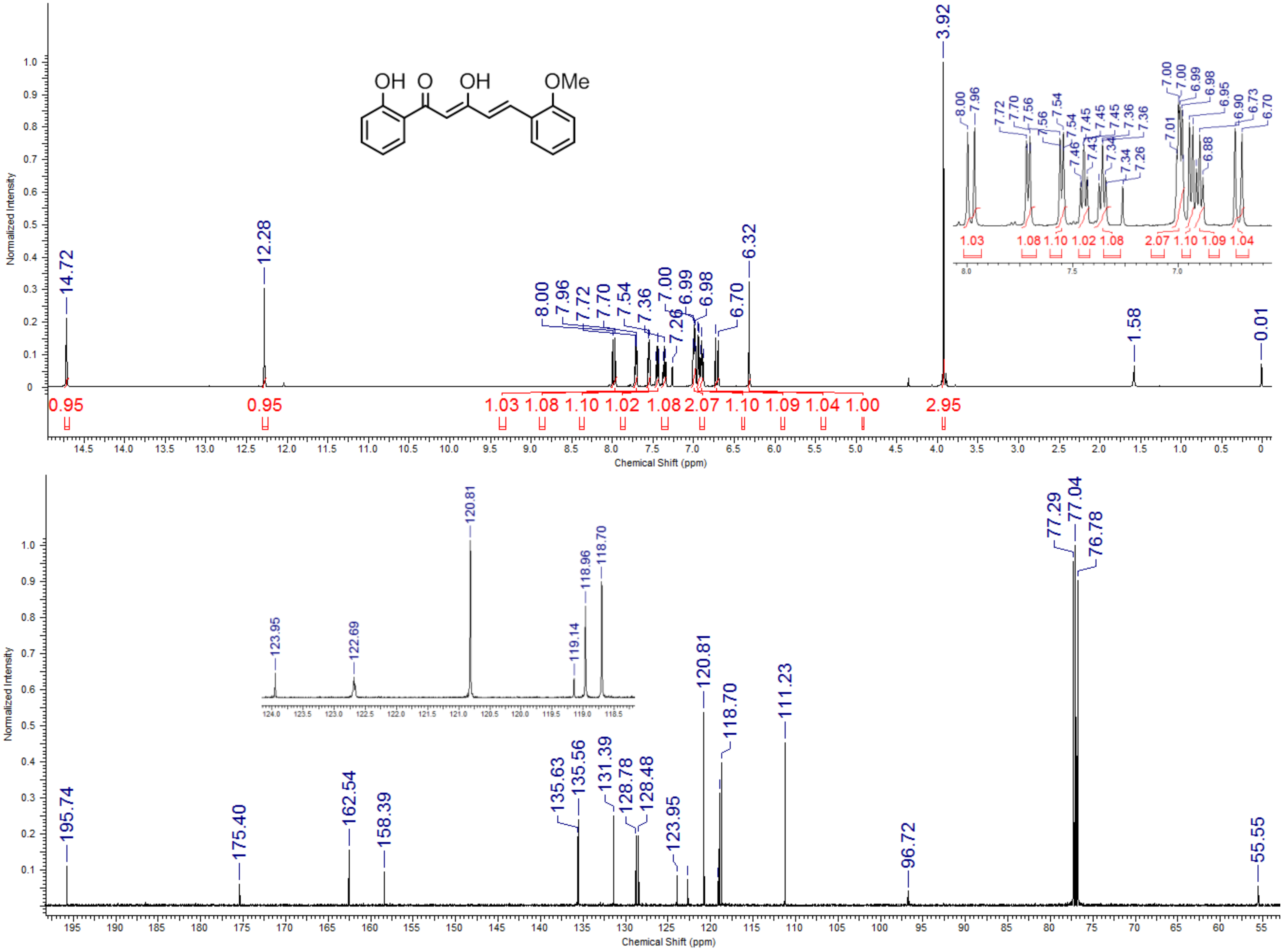

Figure S6. NMR spectra of compound **6**.

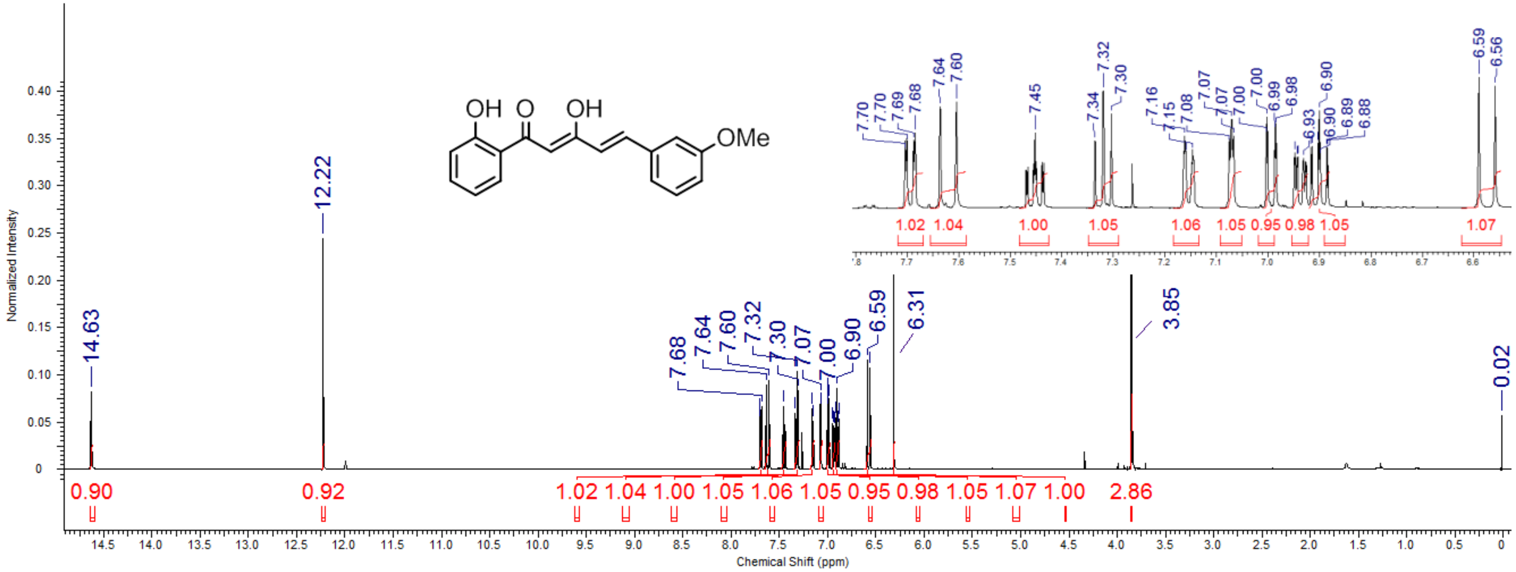

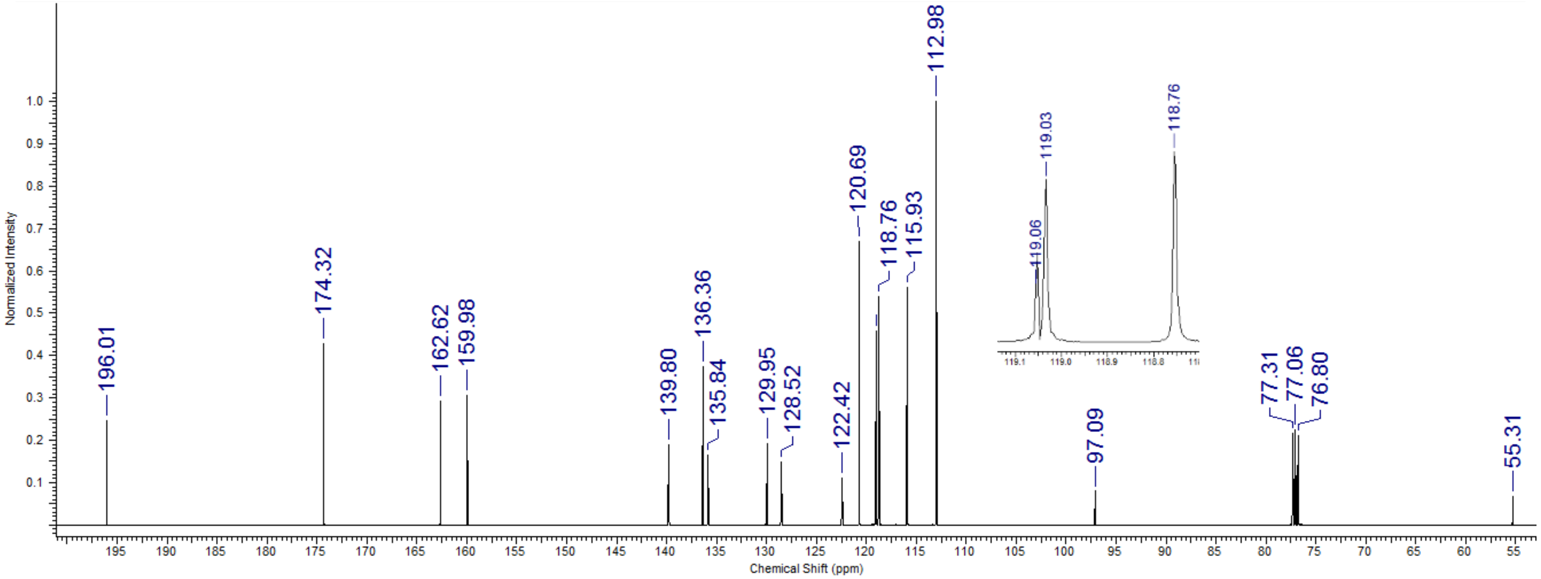

Figure S7. NMR spectra of compound **7**.

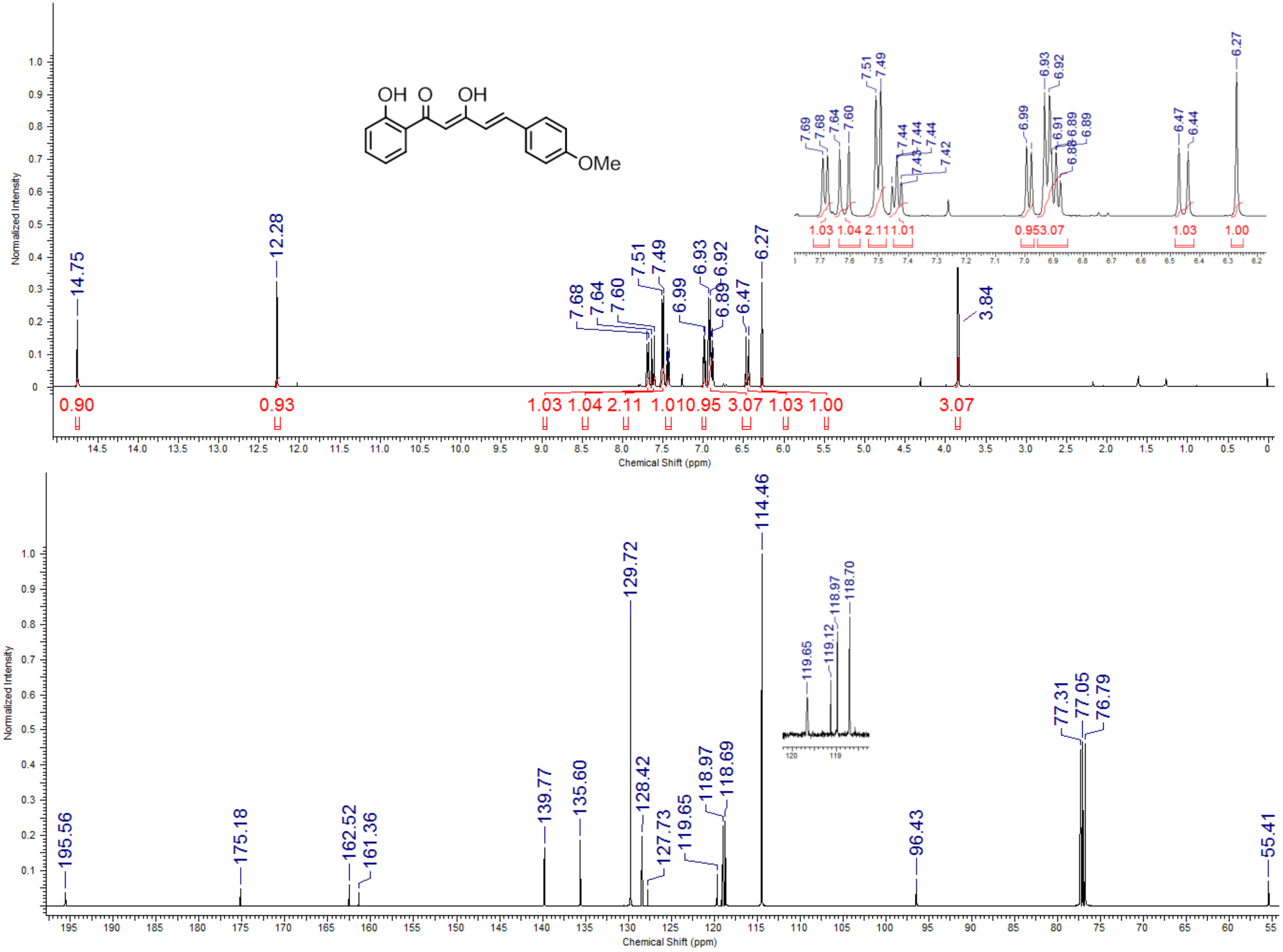

Figure S8. NMR spectra of compound **8**.

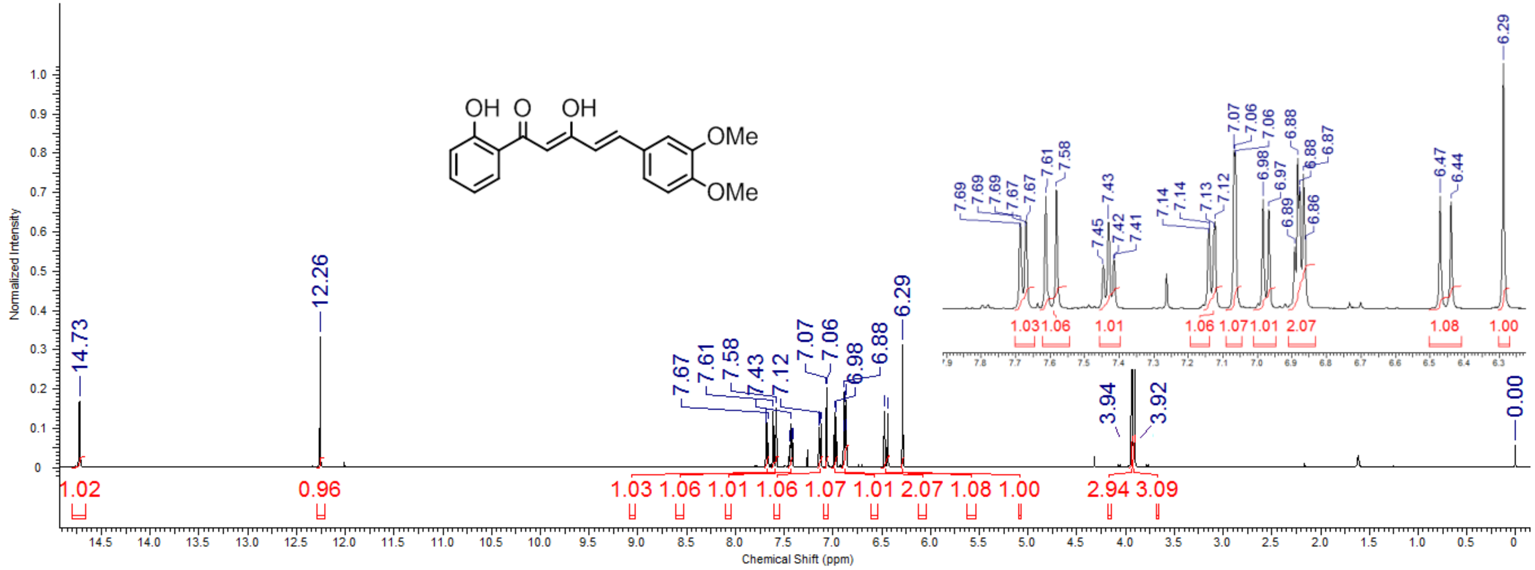

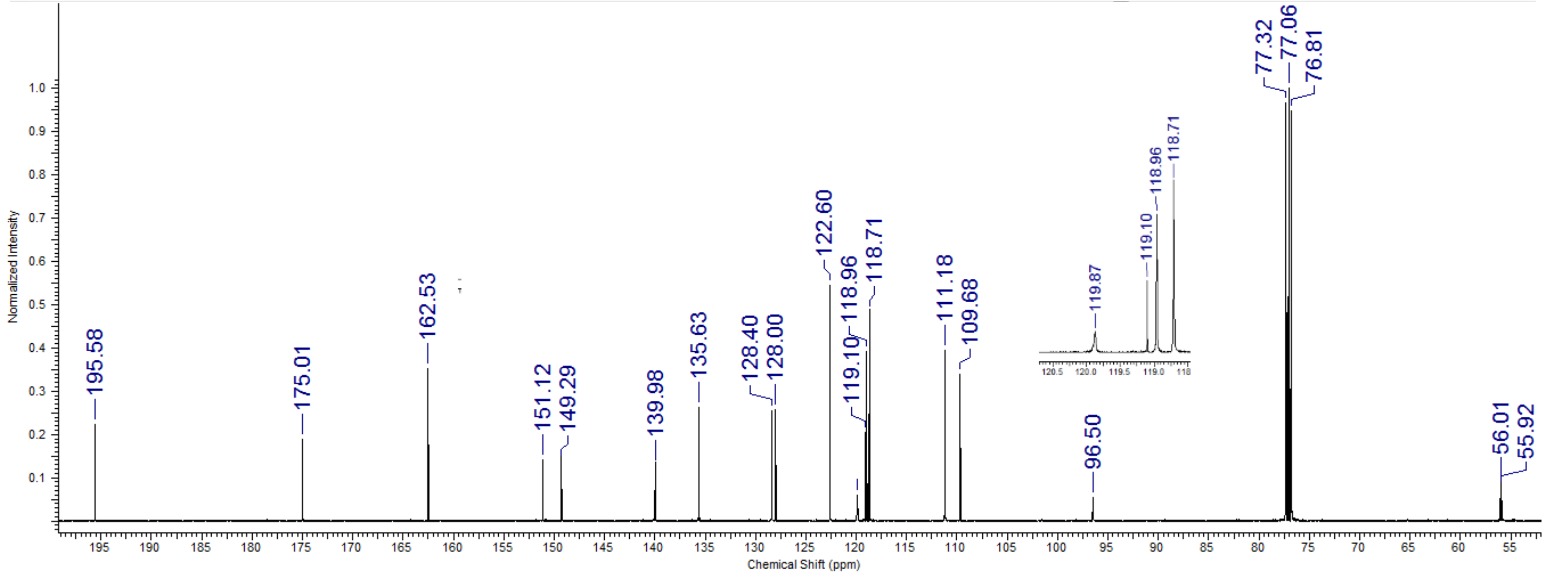

Figure S9. NMR spectra of compound **9**.

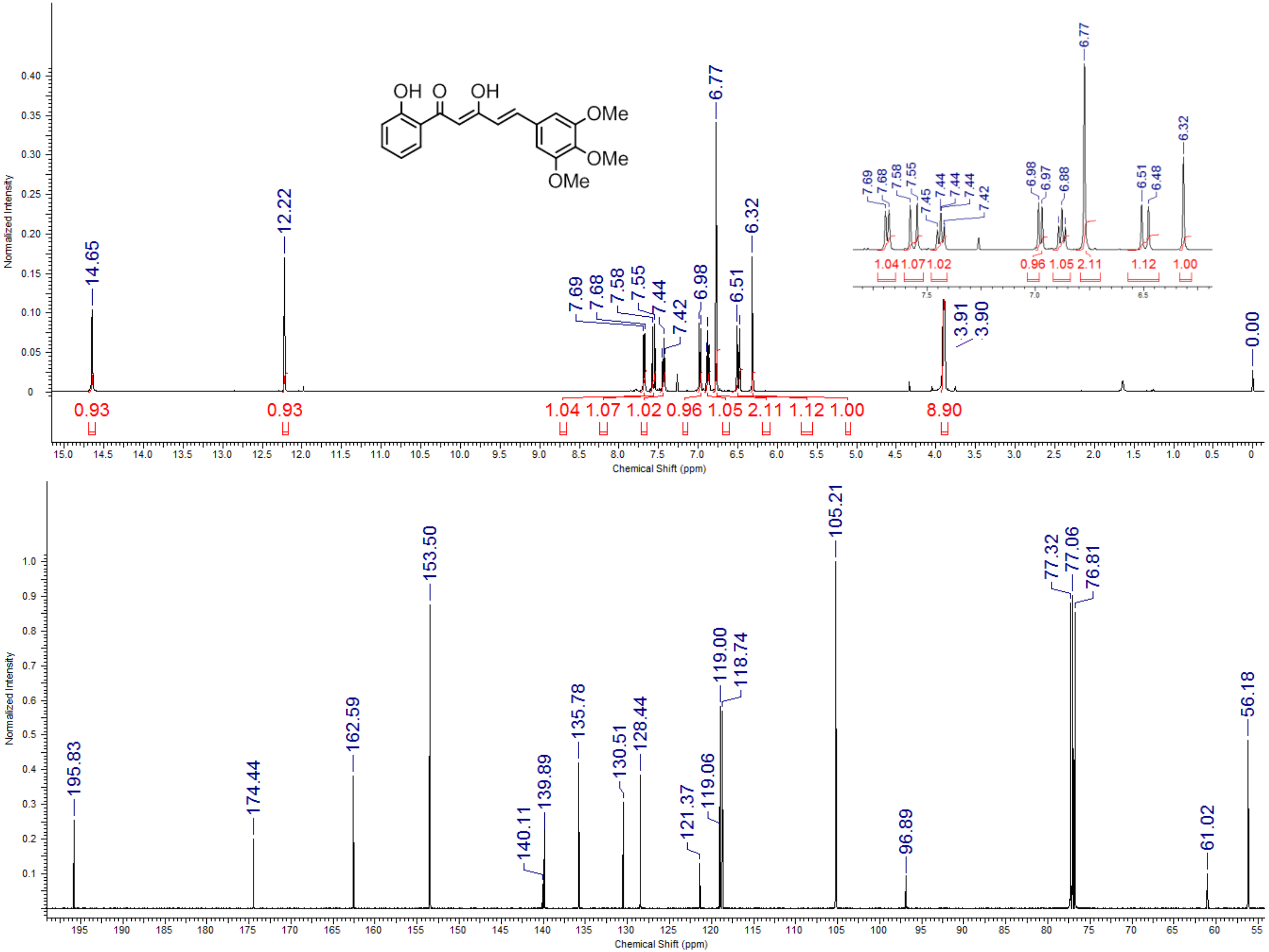

Figure S10. NMR spectra of compound **10**.

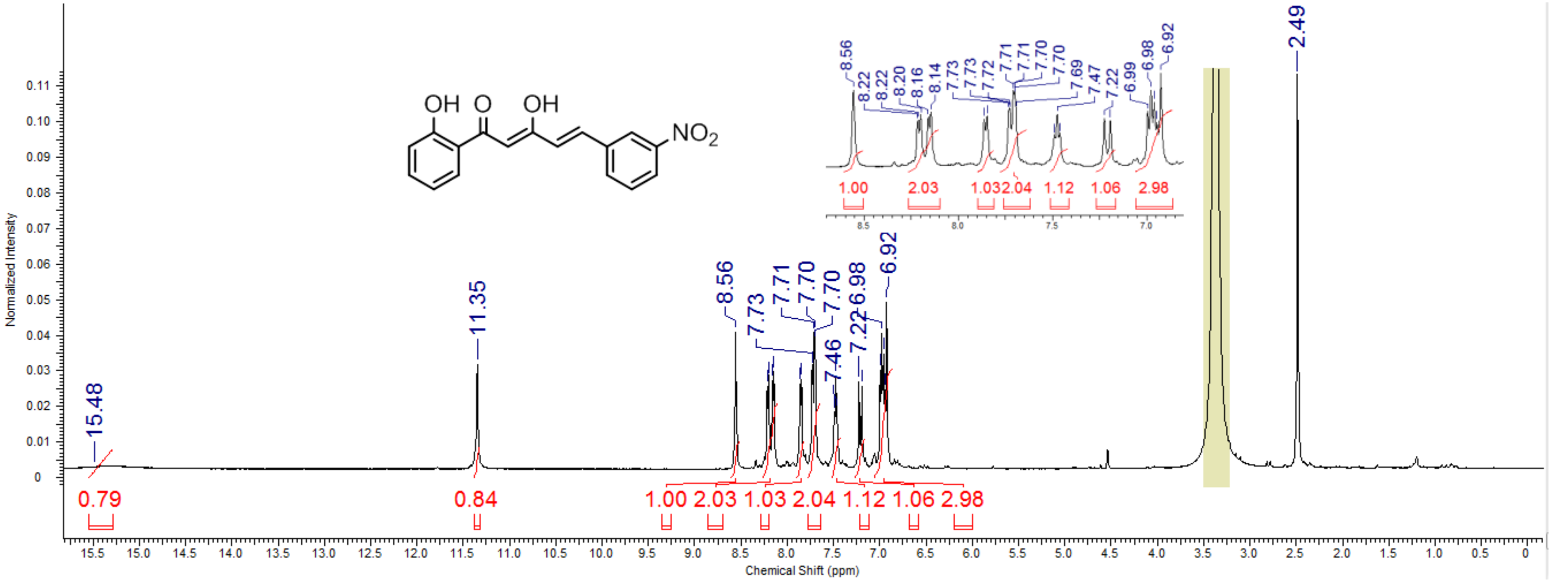

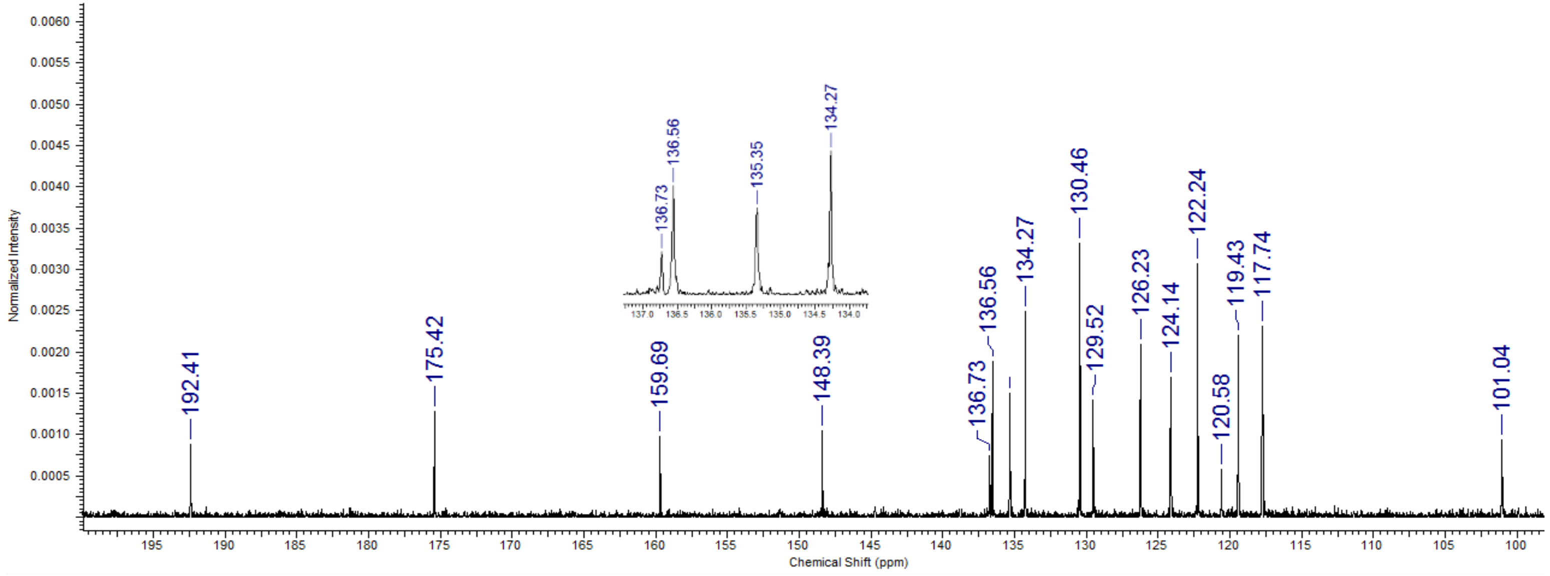

Figure S11. NMR spectra of compound **11**.

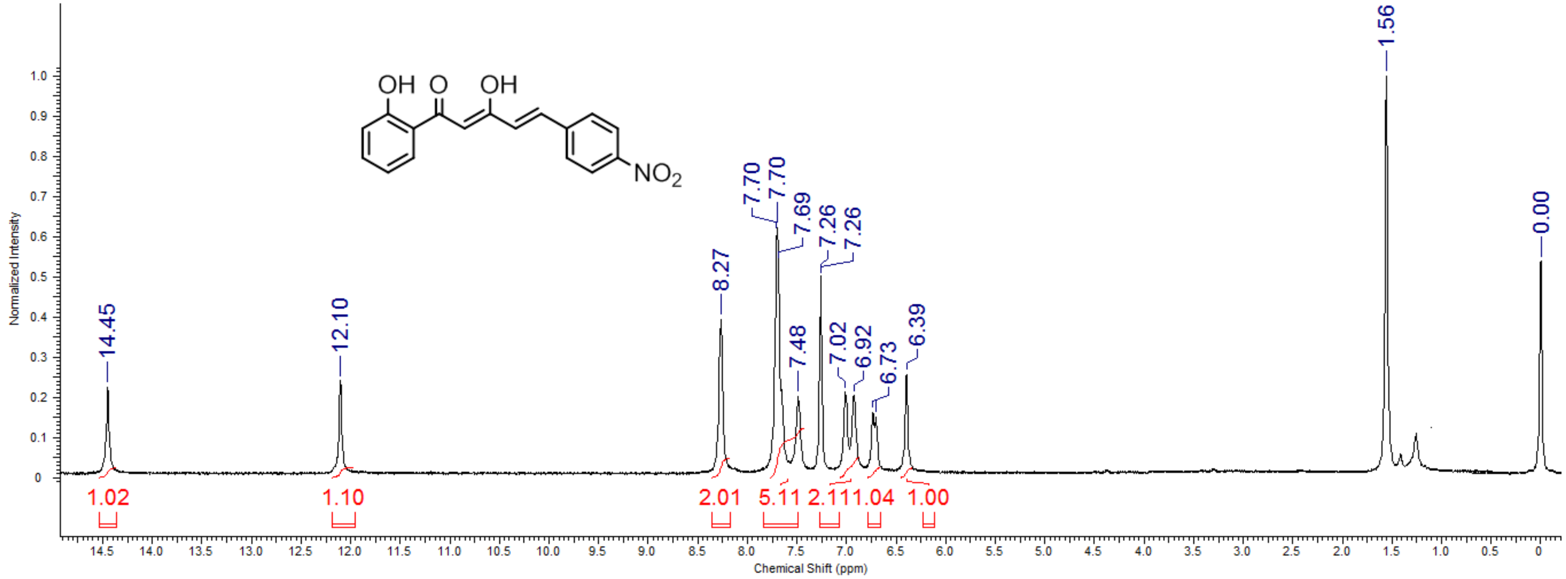

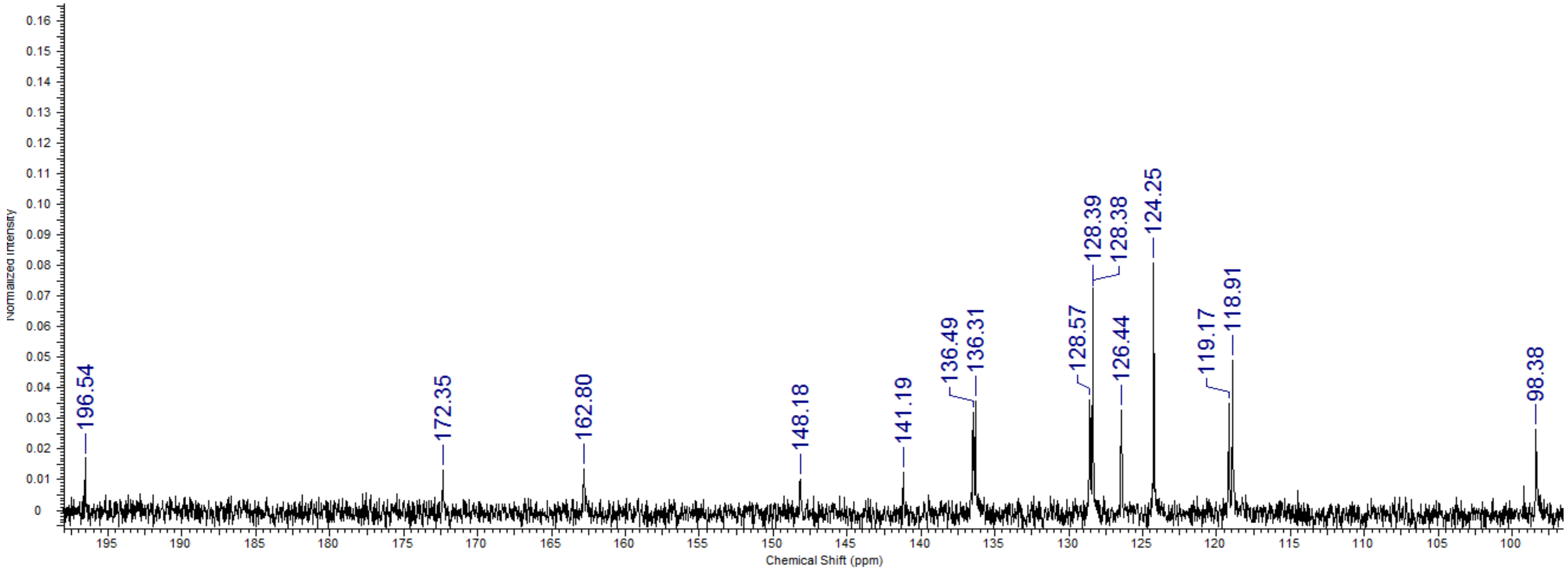

Figure S12. NMR spectra of compound **12**.

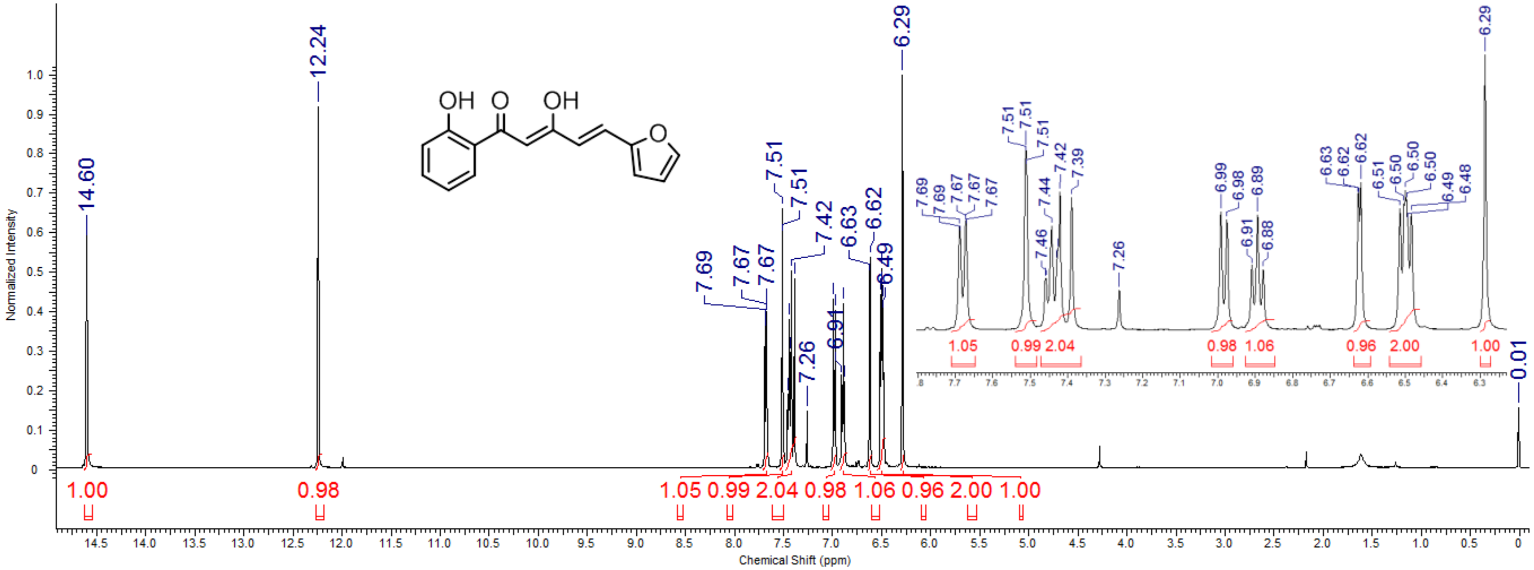

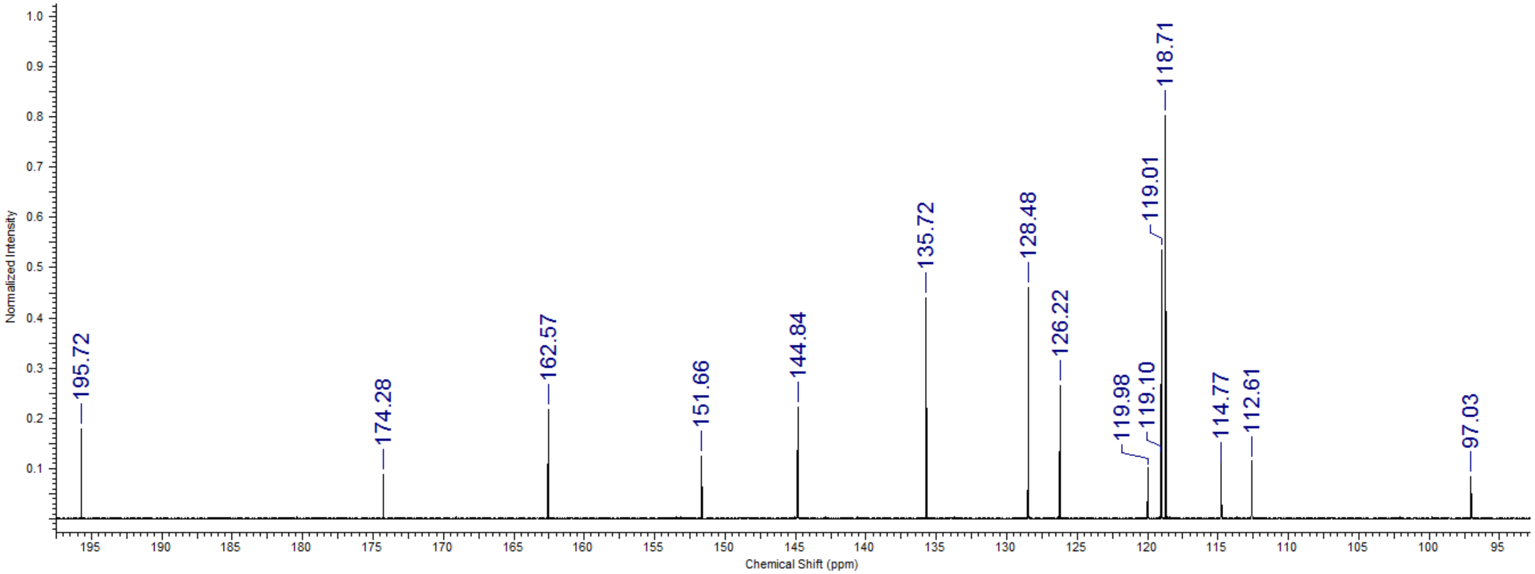

Figure S13. NMR spectra of compound **13**.

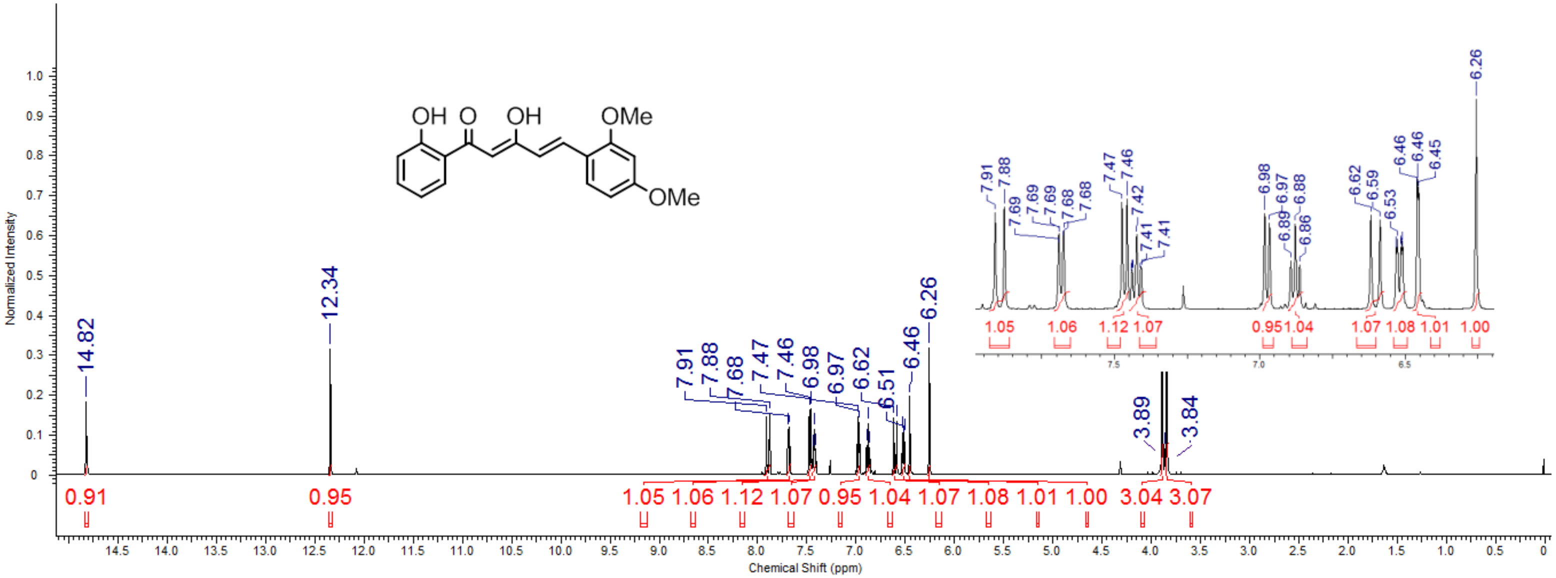

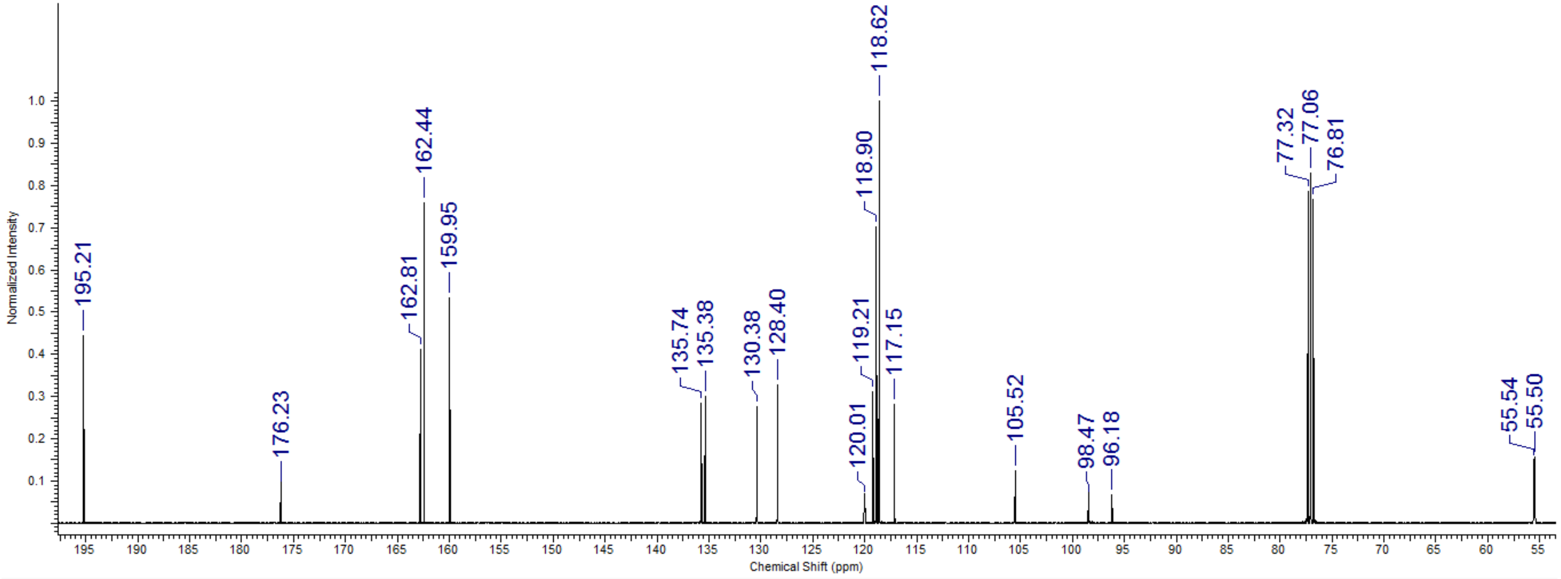

Figure S14. NMR spectra of compound **14**.

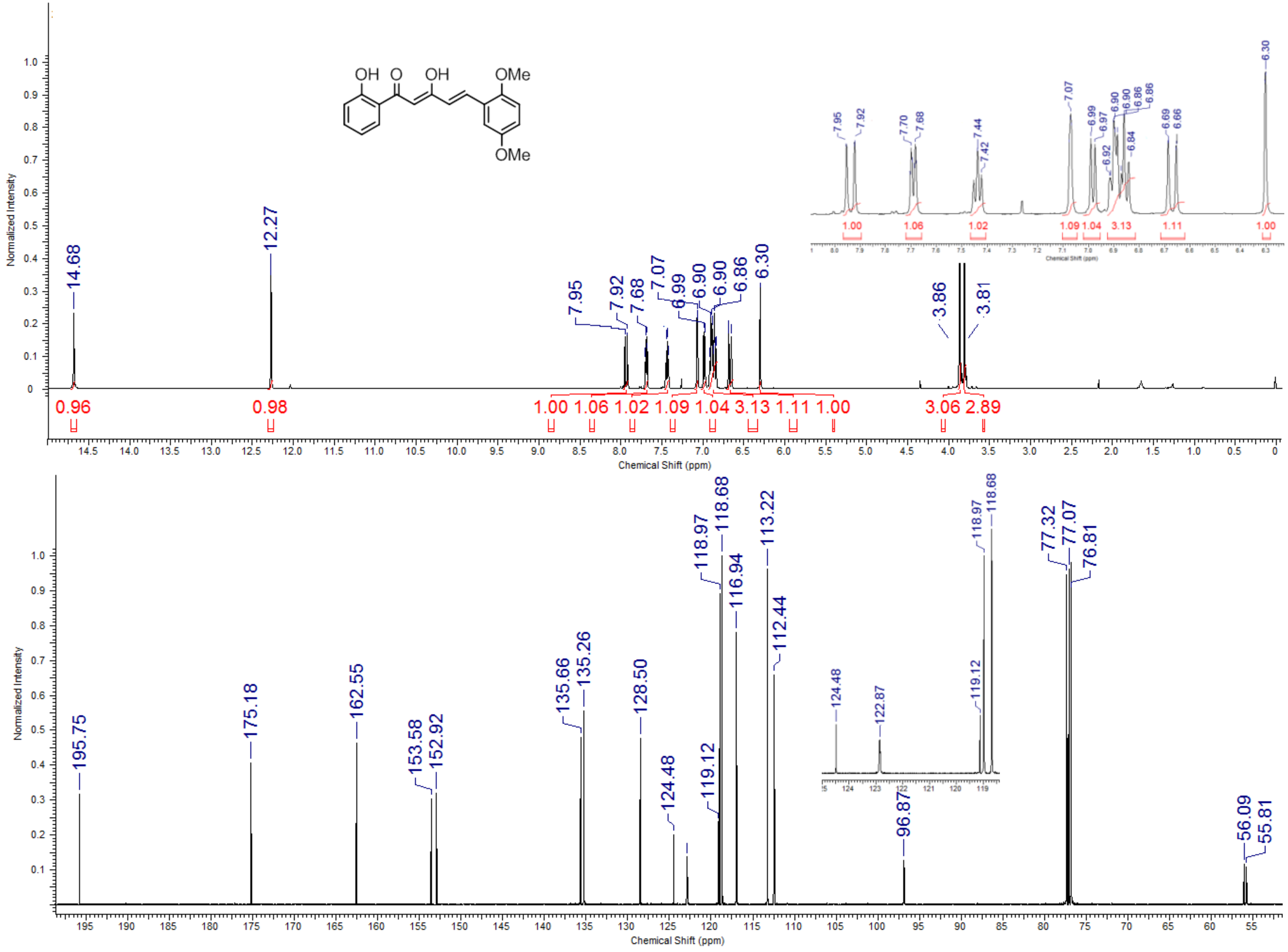

Figure S15. NMR spectra of compound **15**.

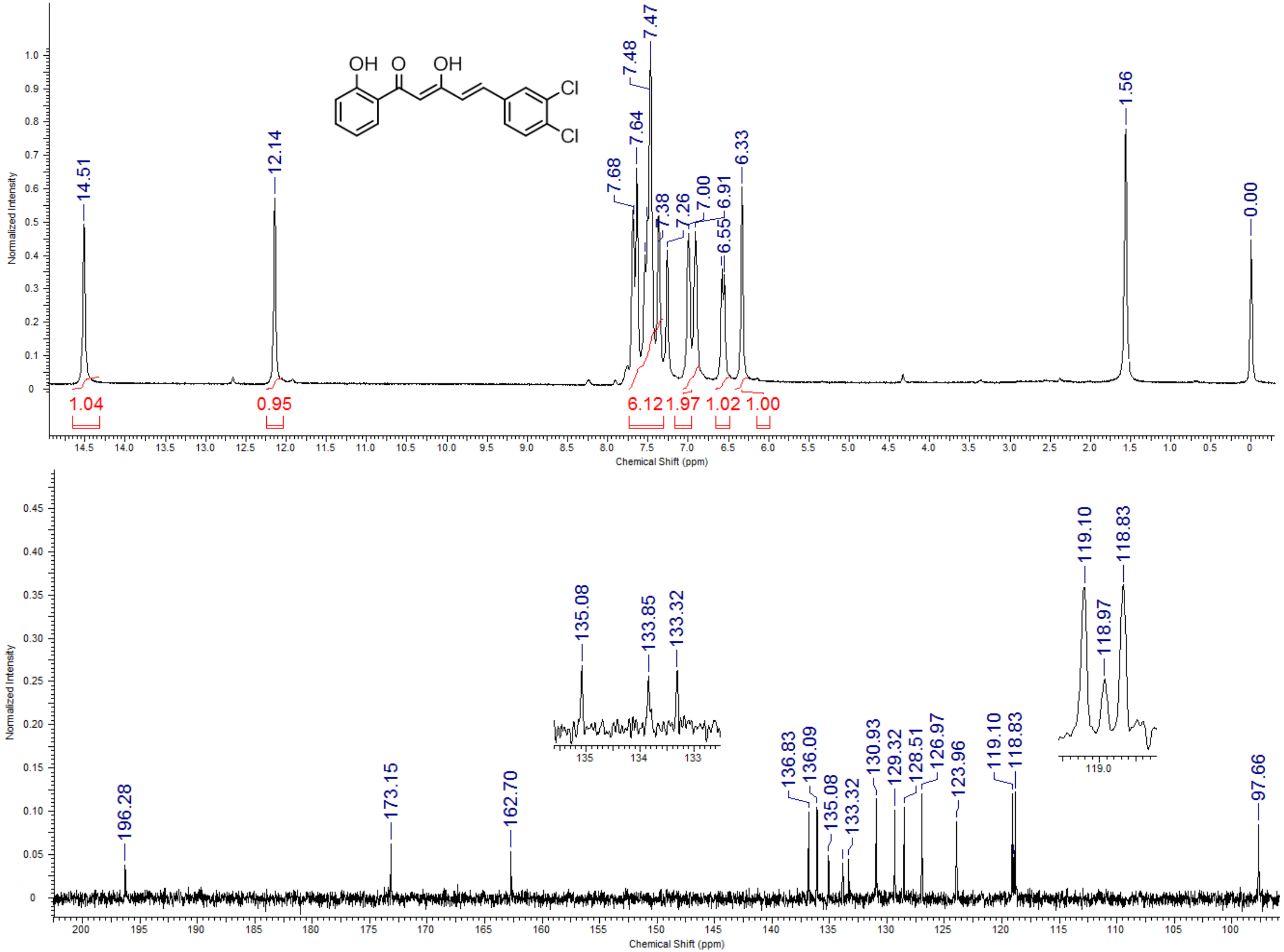

Figure S16. NMR spectra of compound **16.**

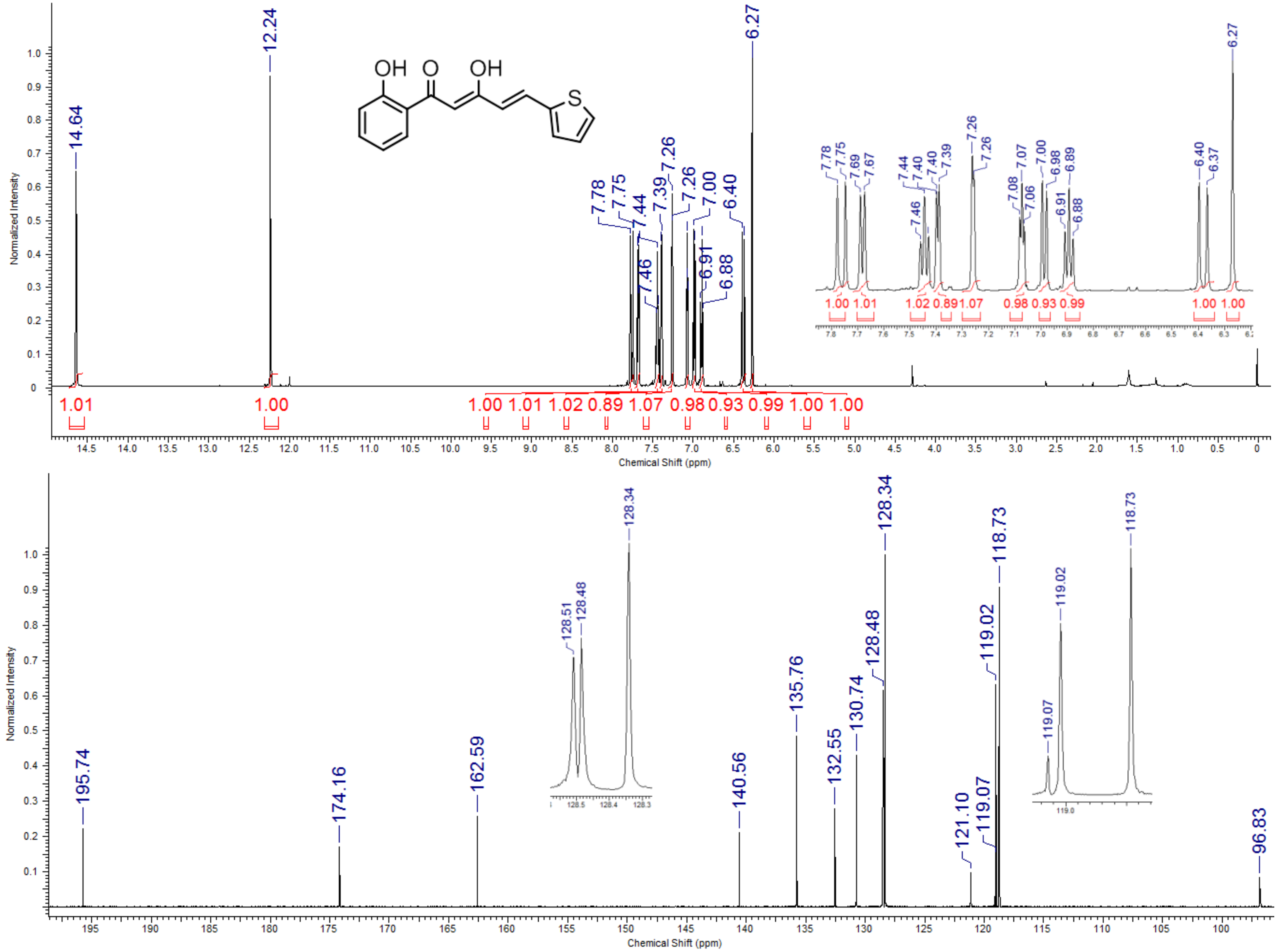

Figure S17. NMR spectra of compound **17**.

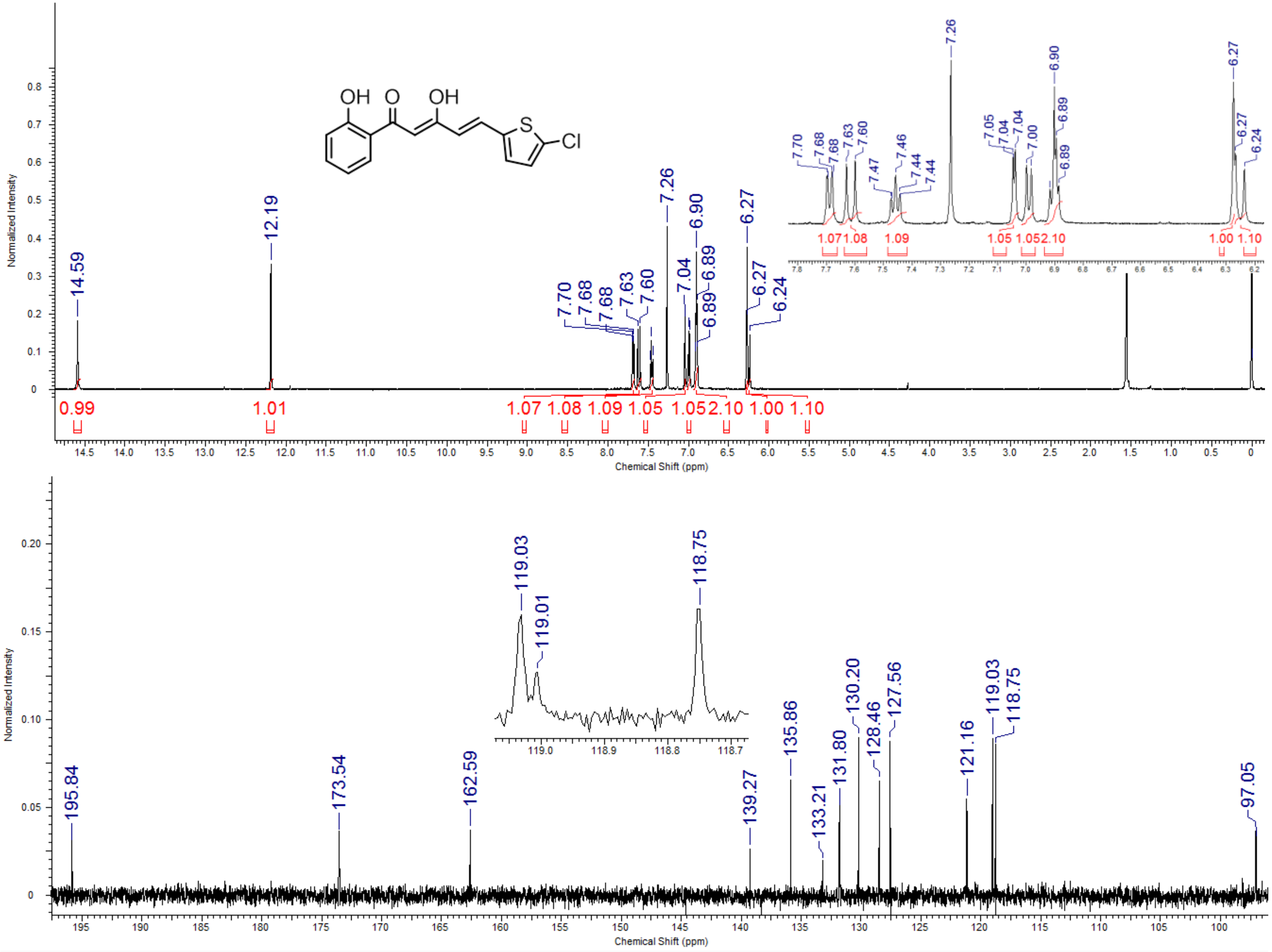

Figure S18. NMR spectra of compound **18**.

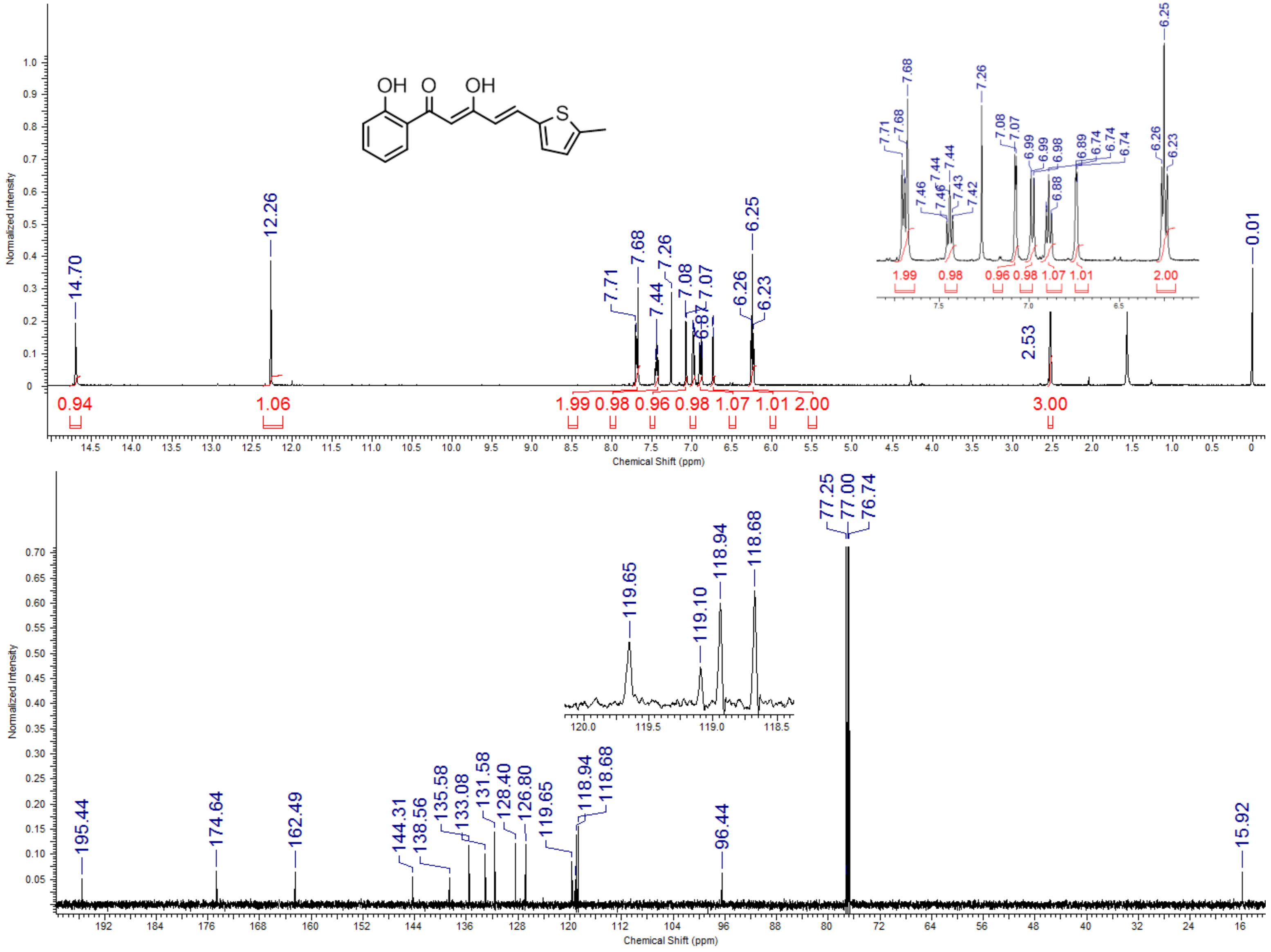

Figure S19. NMR spectra of compound **19**.

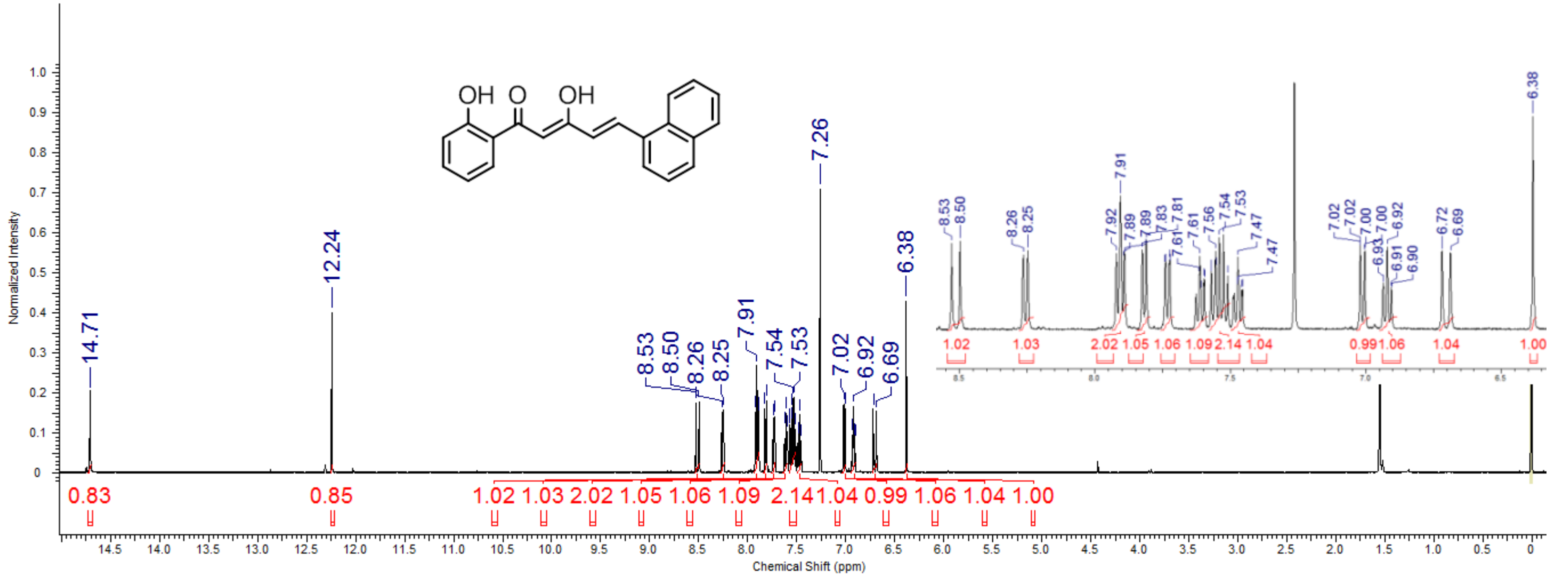

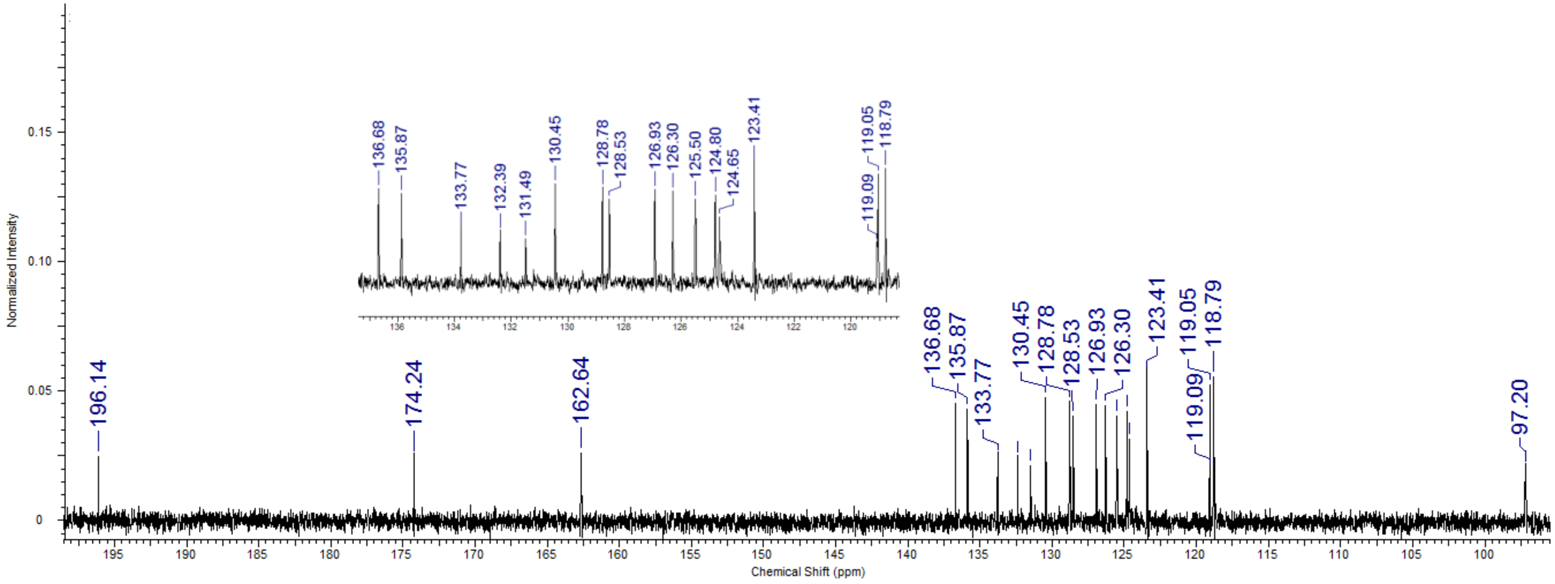

Figure S20. NMR spectra of compound **20**.

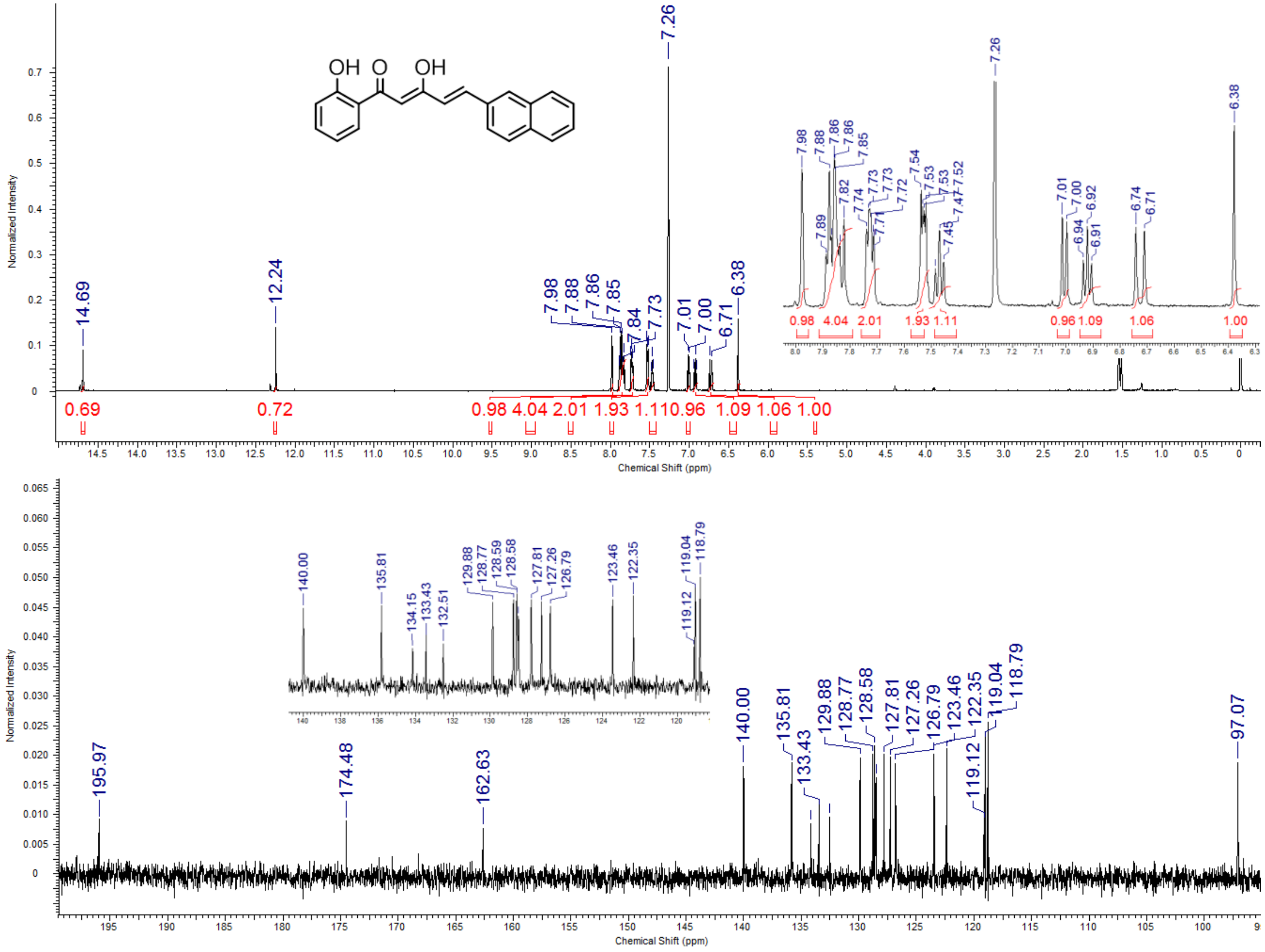

Figure S21. NMR spectra of compound **21**.

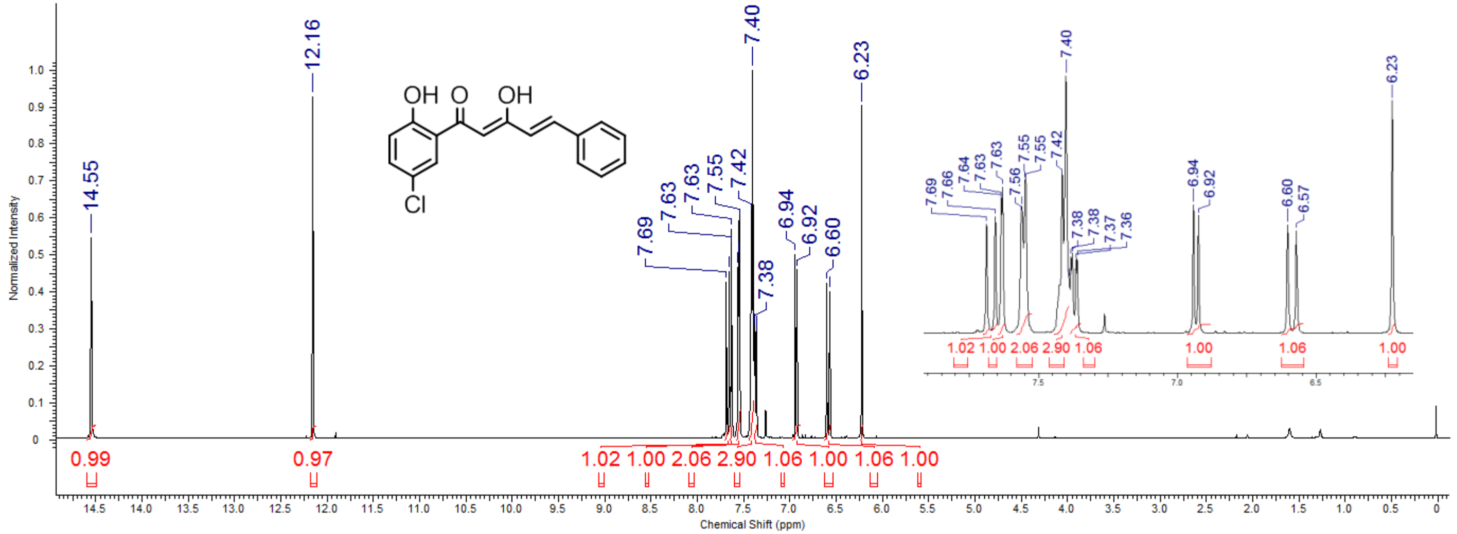

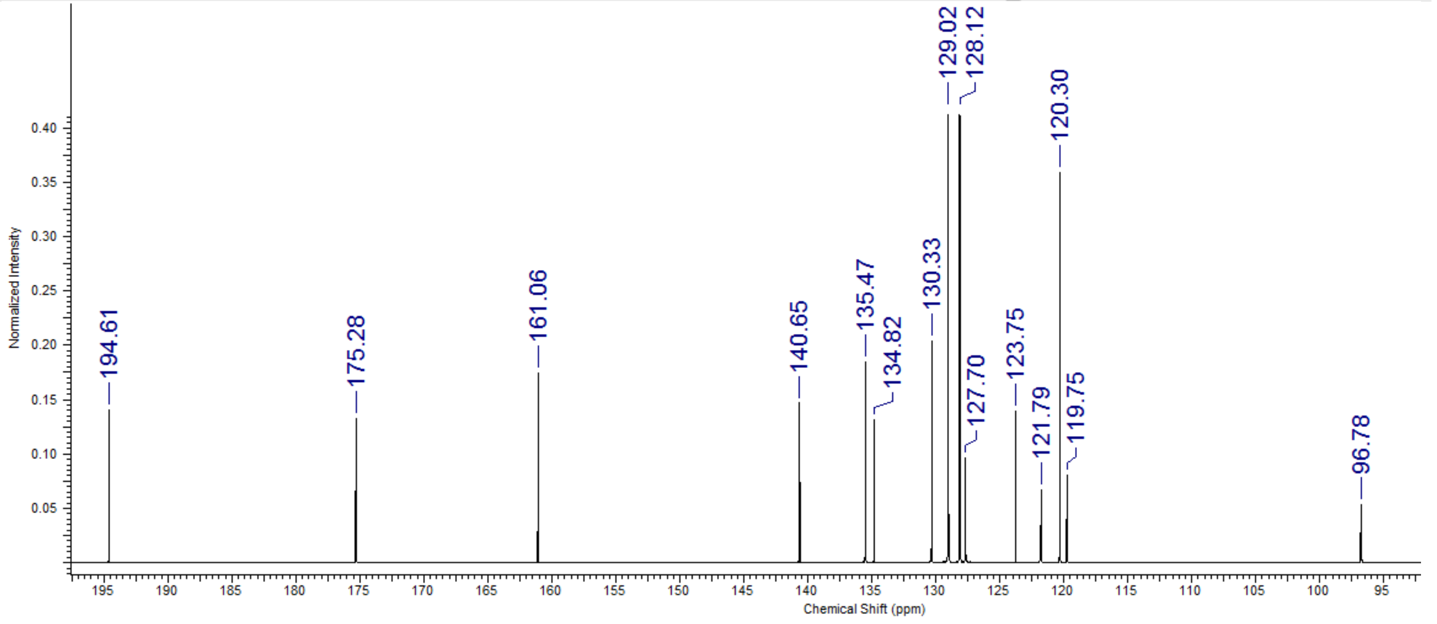

Figure S22. NMR spectra of compound **22**.

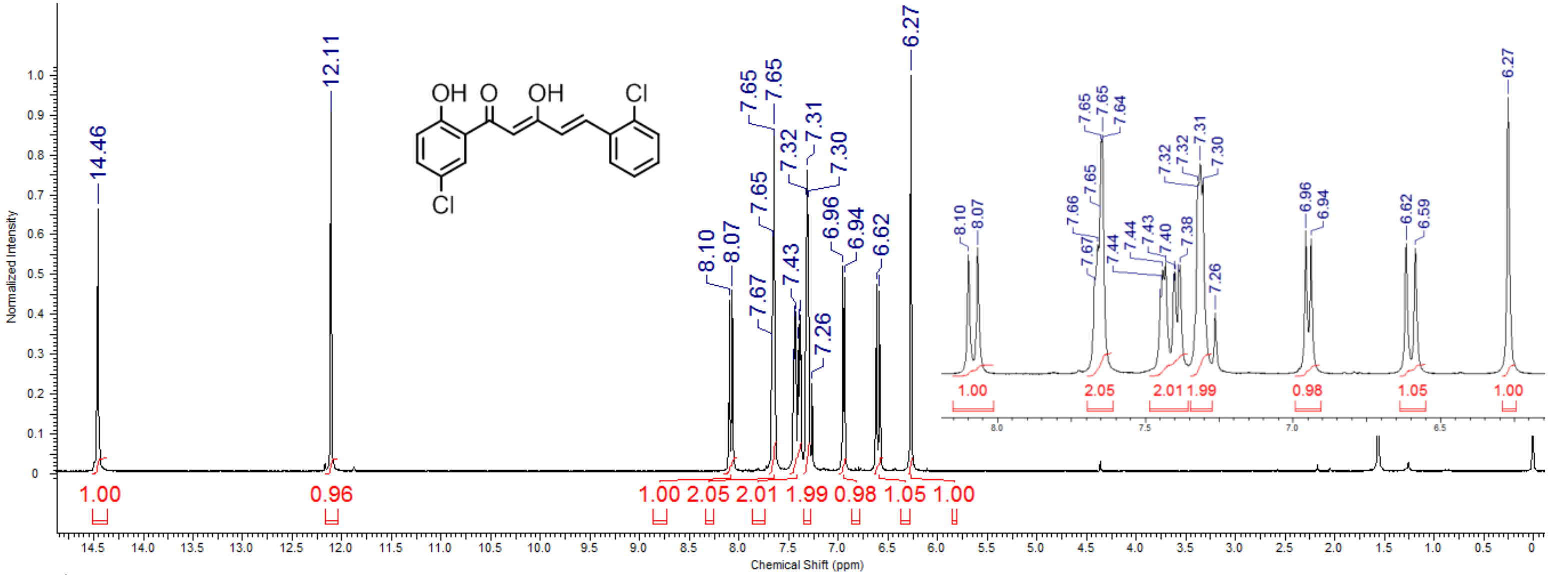

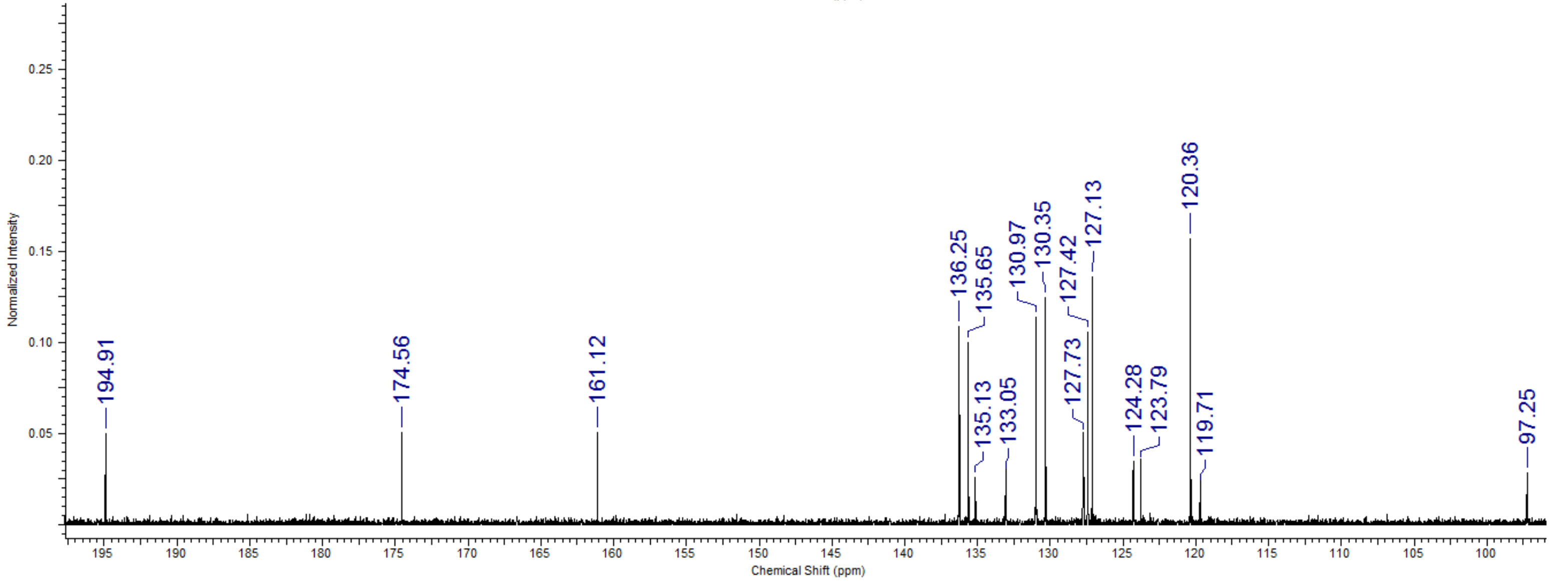

Figure S23. NMR spectra of compound **23**.

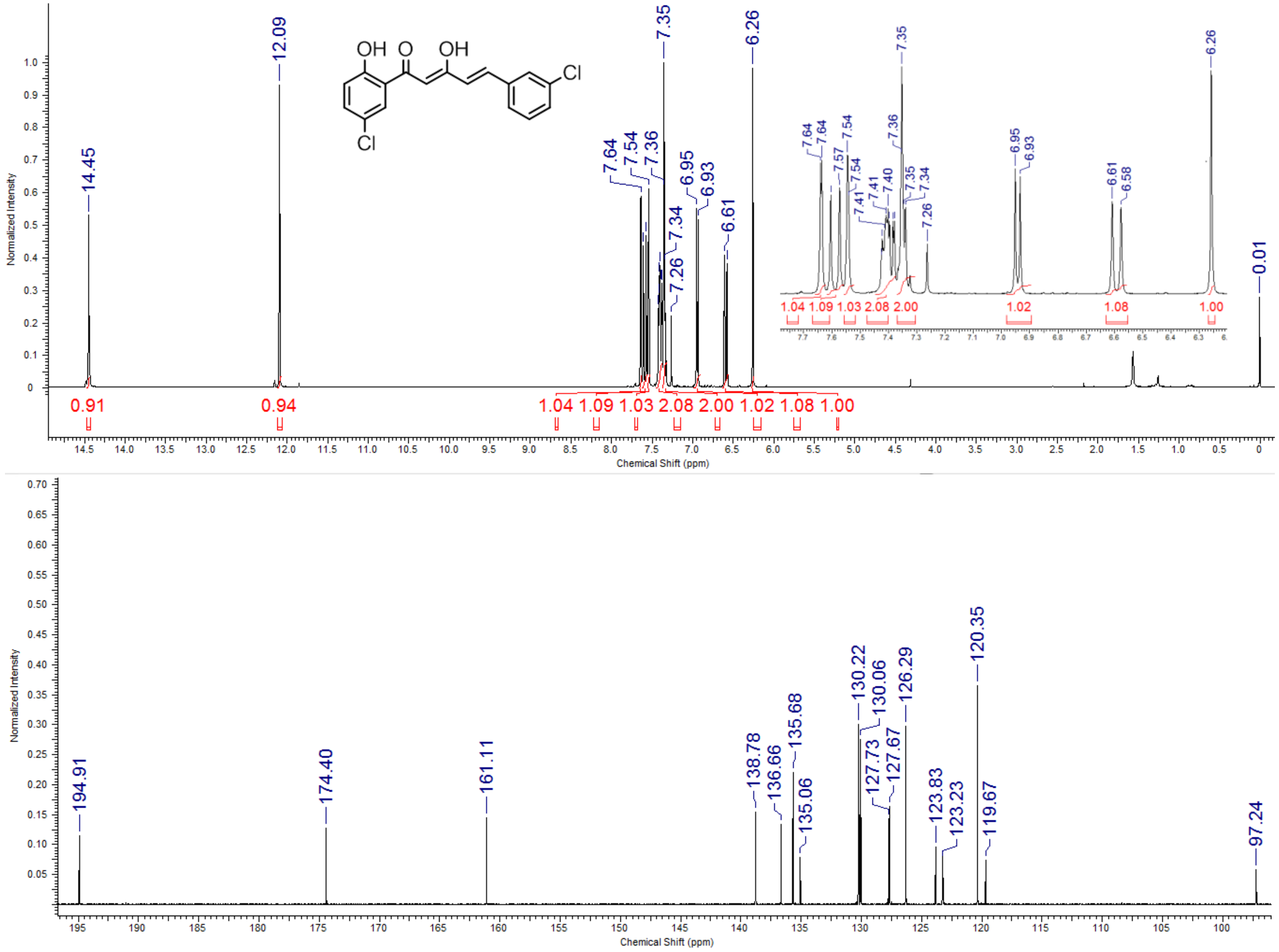

Figure S24. NMR spectra of compound **24**.

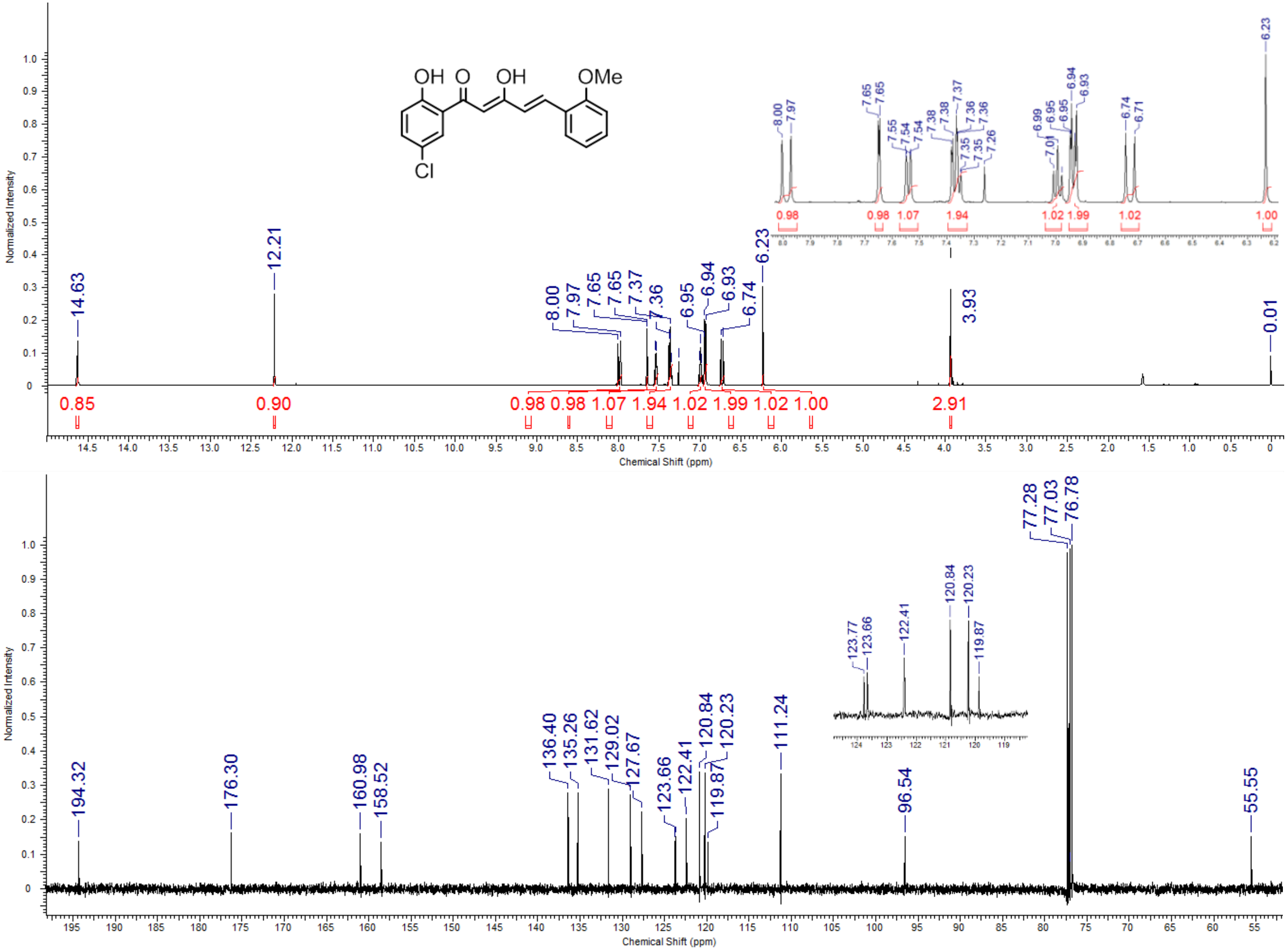

Figure S25. NMR spectra of compound **25**.

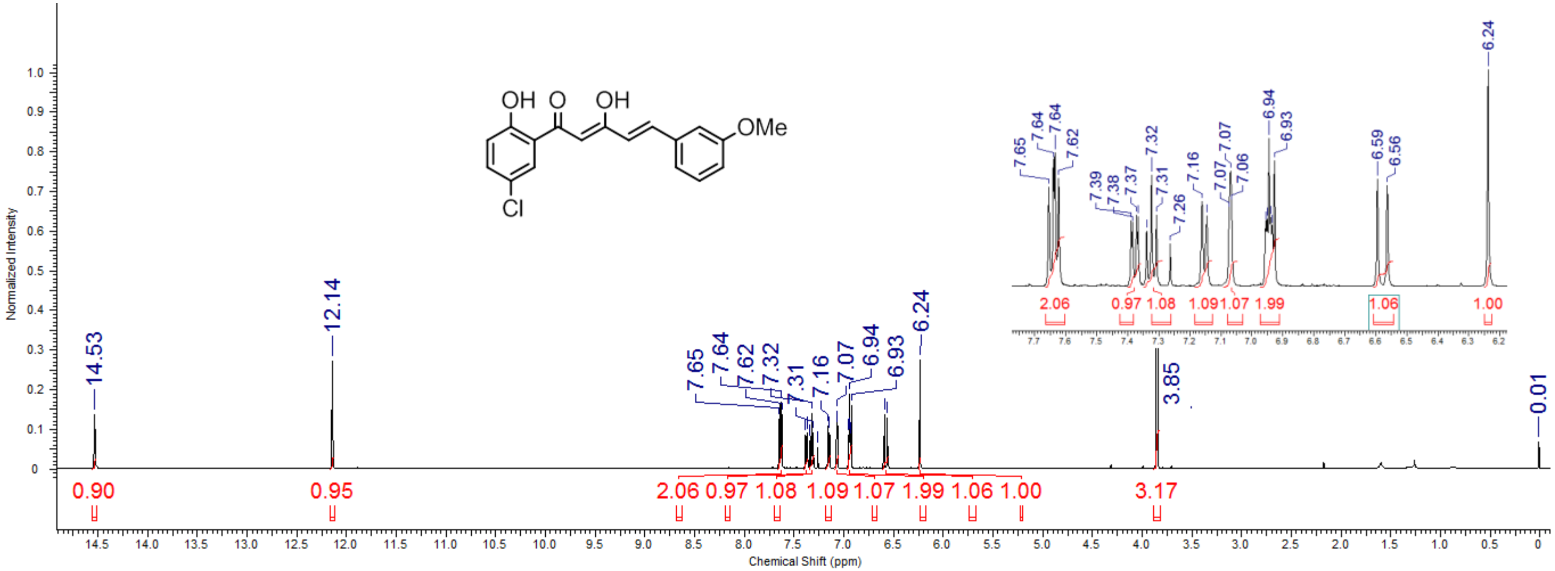

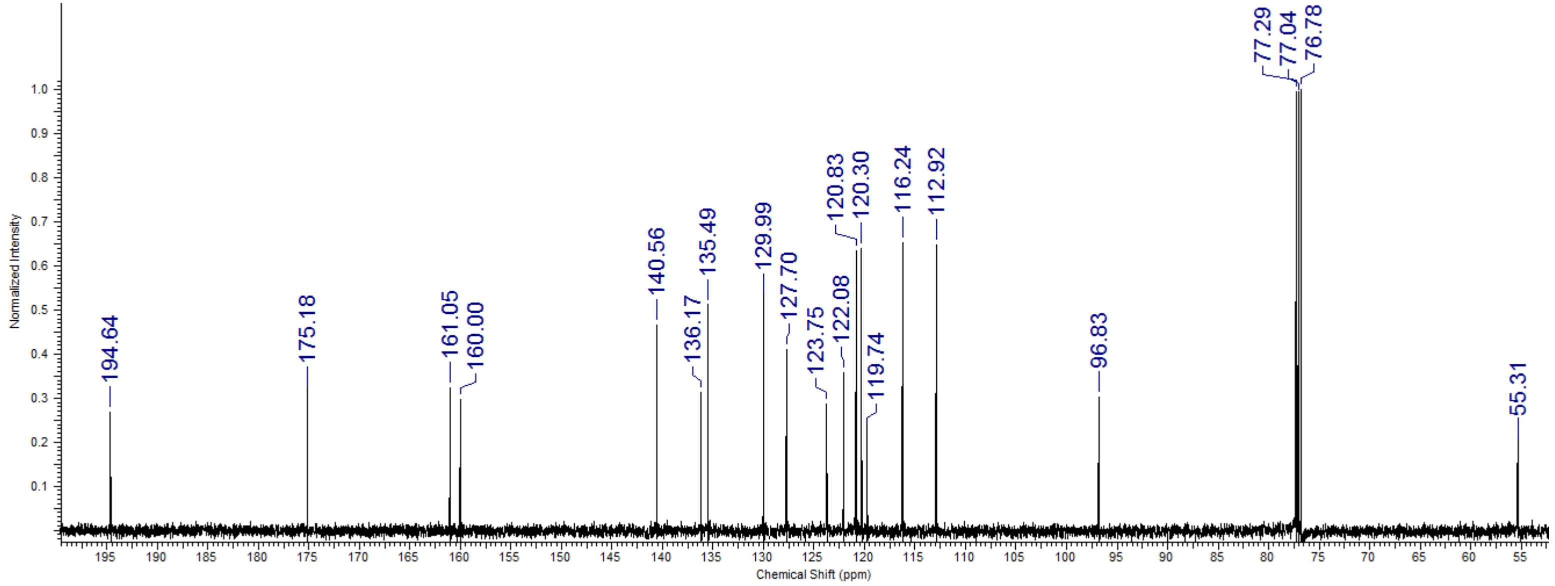

Figure S26. NMR spectra of compound **26**.

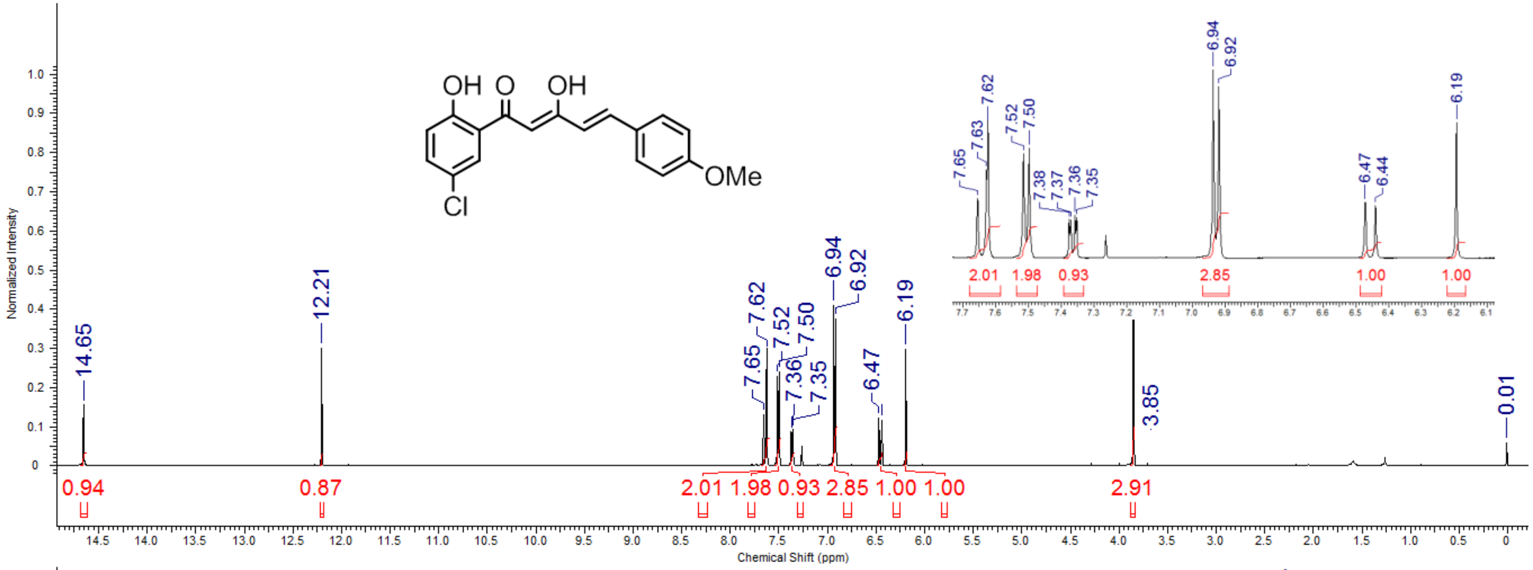

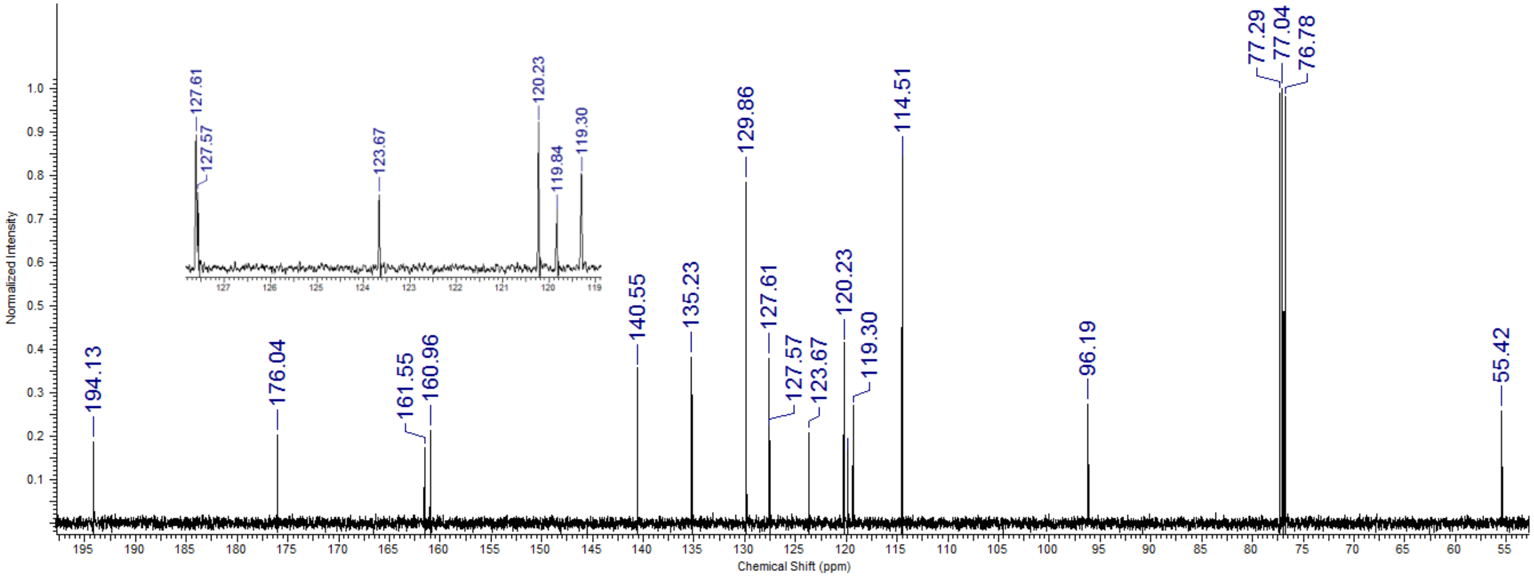

Figure S27. NMR spectra of compound **27**.

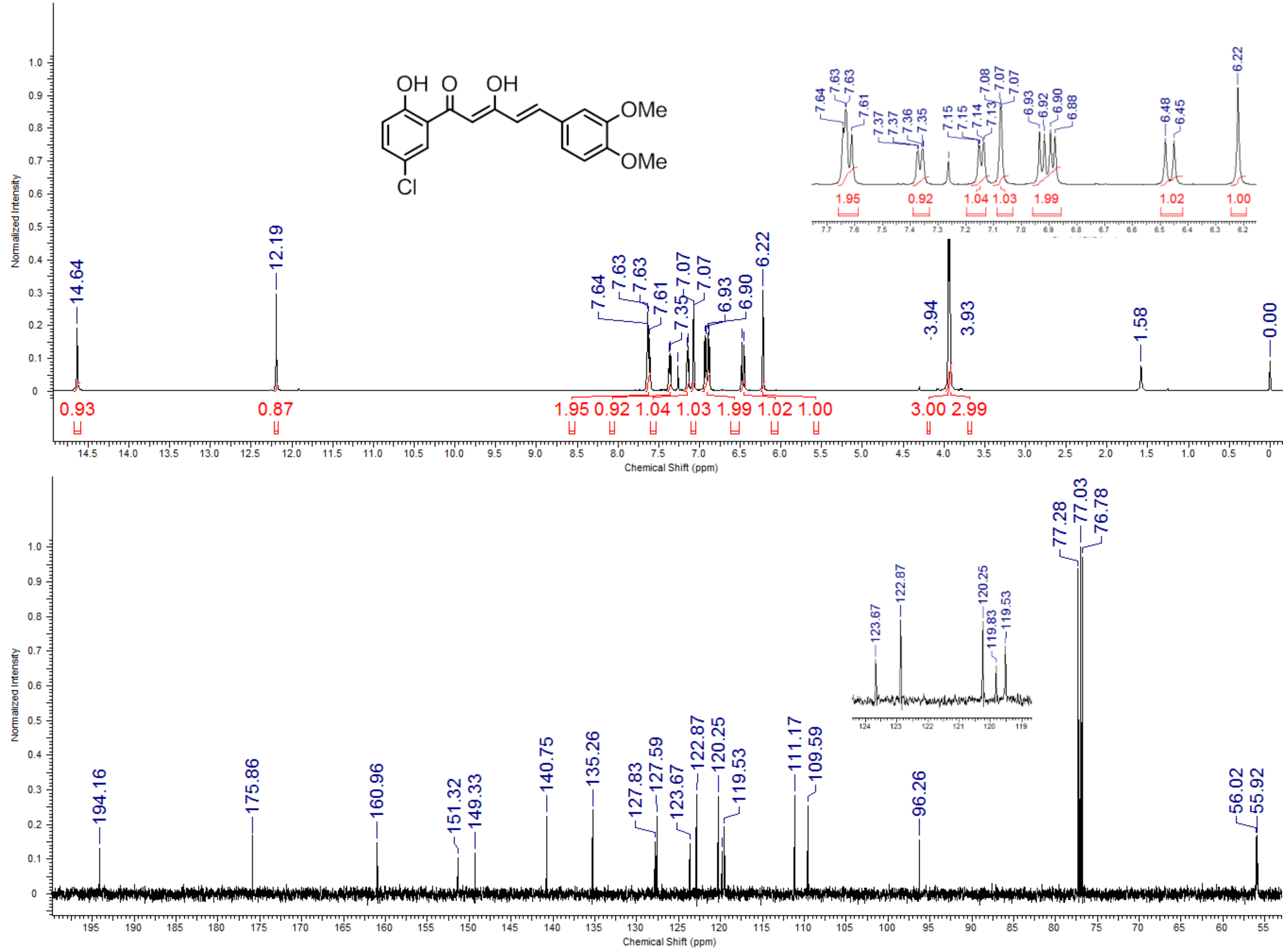

Figure S28. NMR spectra of compound **28**.

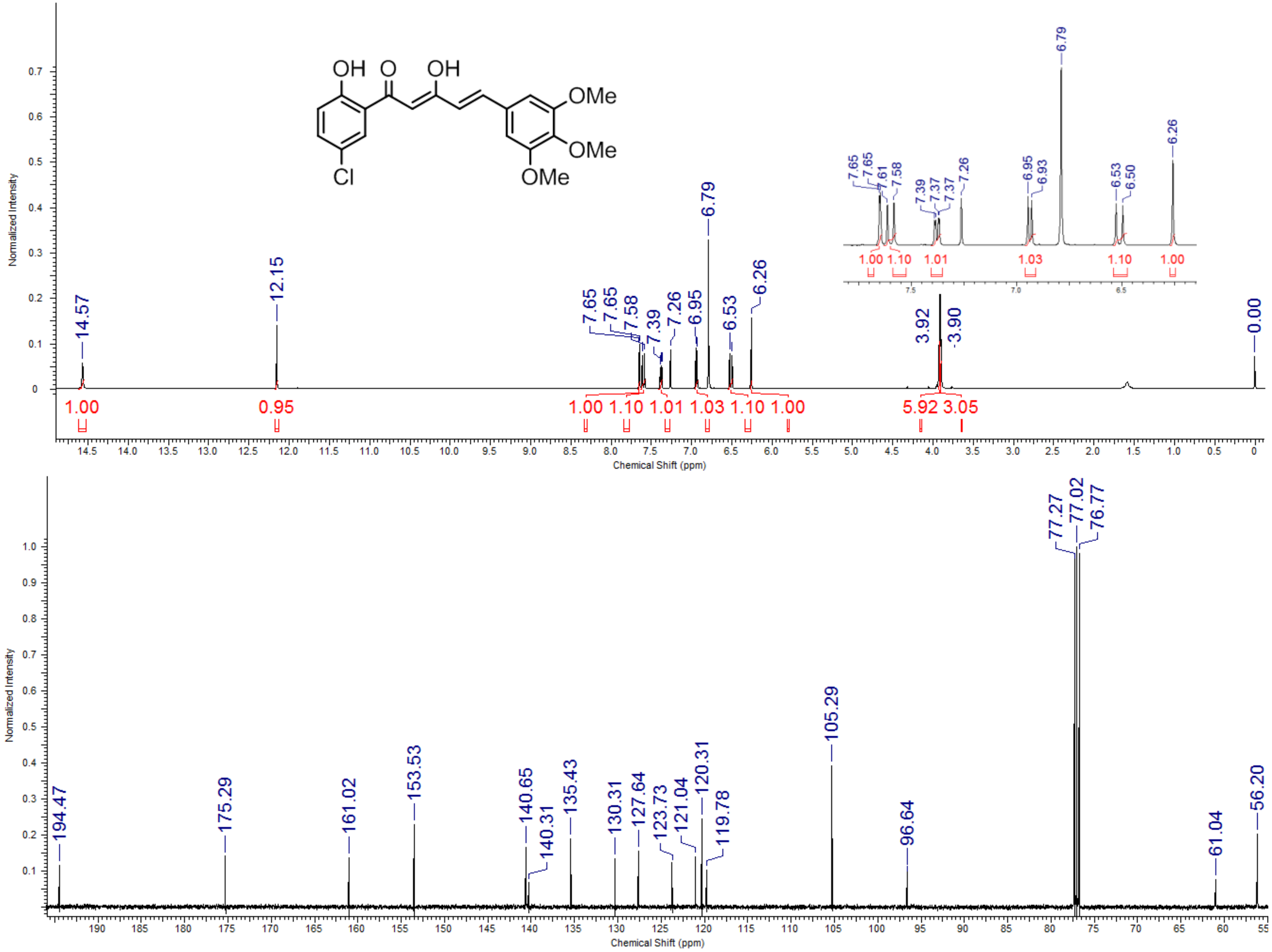

Figure S29. NMR spectra of compound **29**.

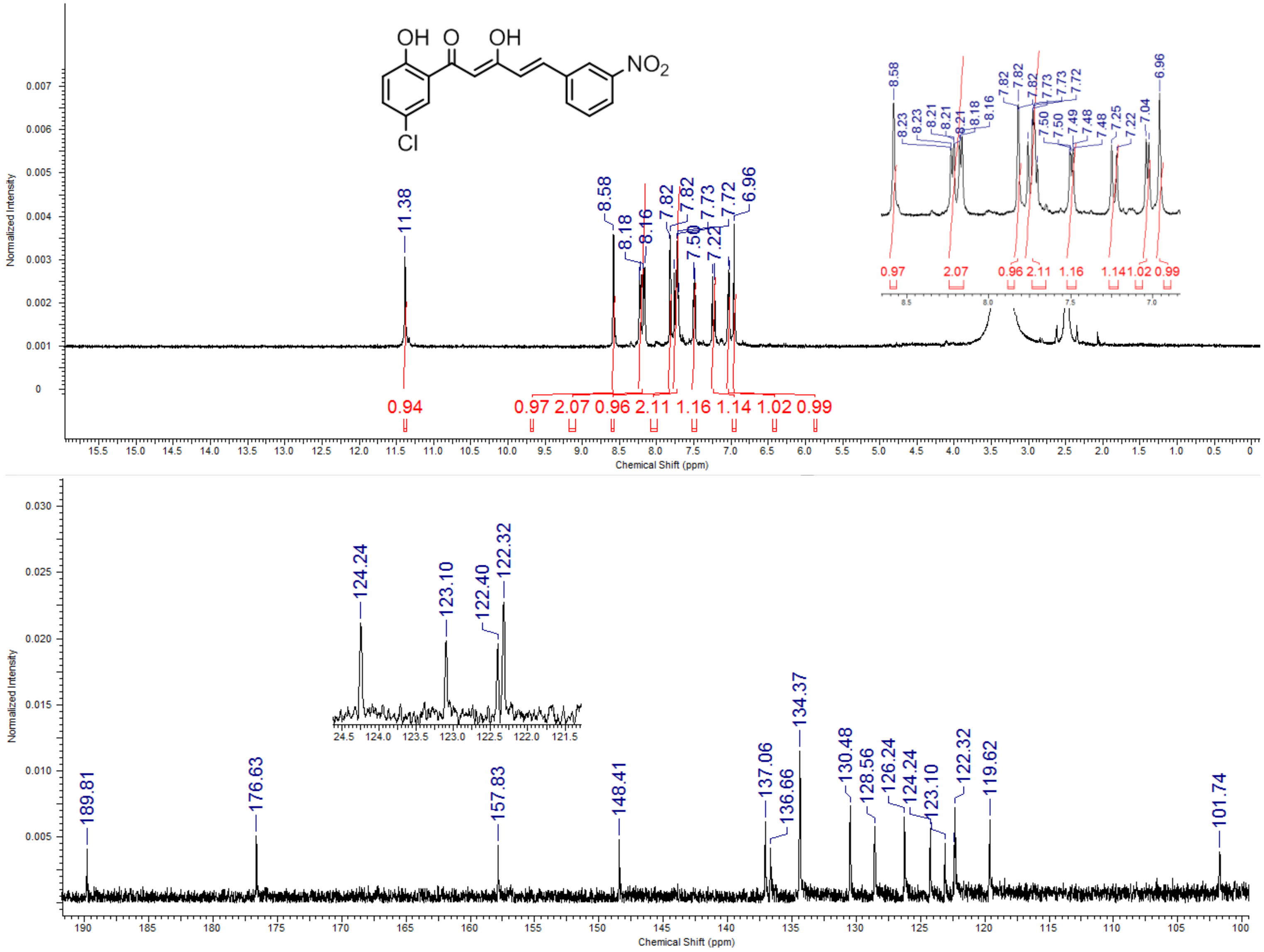

Figure S30. NMR spectra of compound **30**.

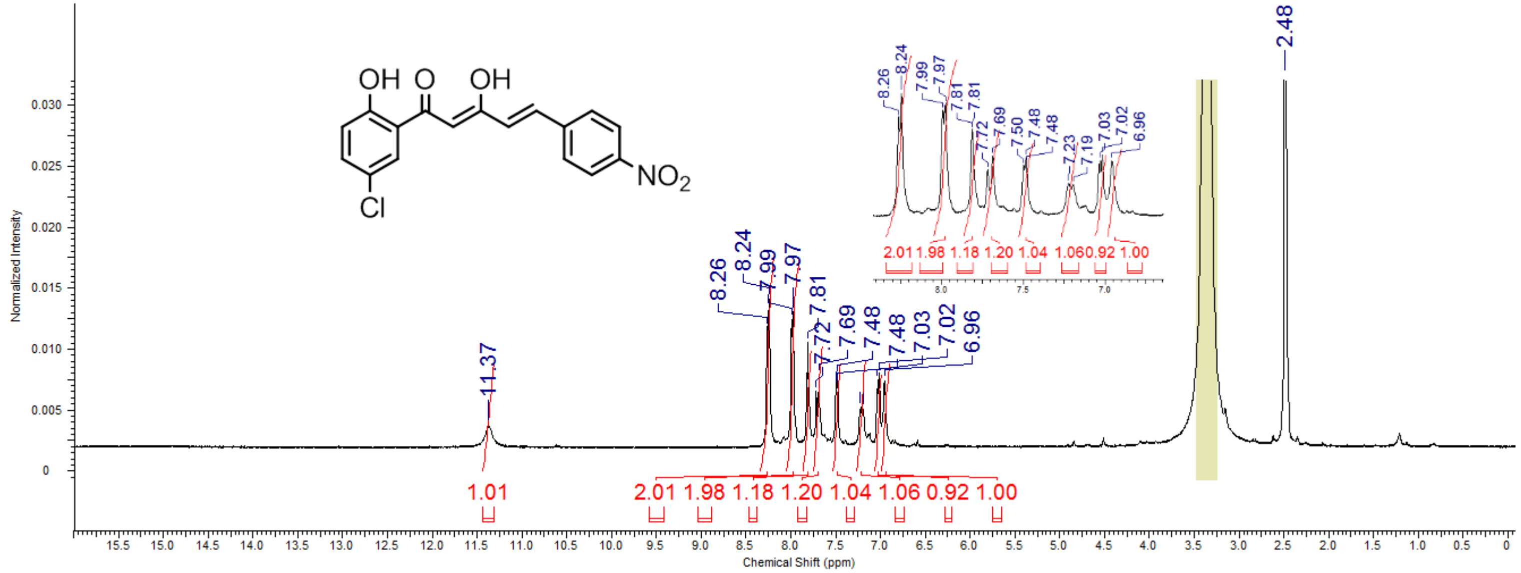

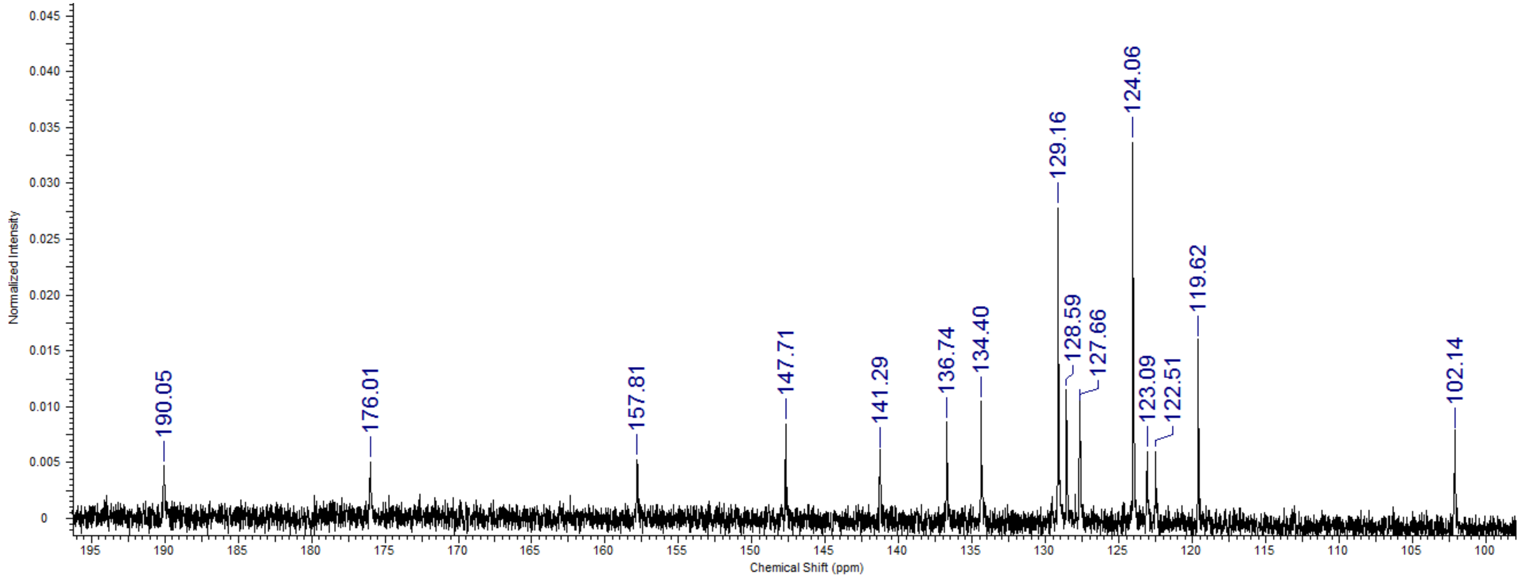

Figure S31. NMR spectra of compound **31**.

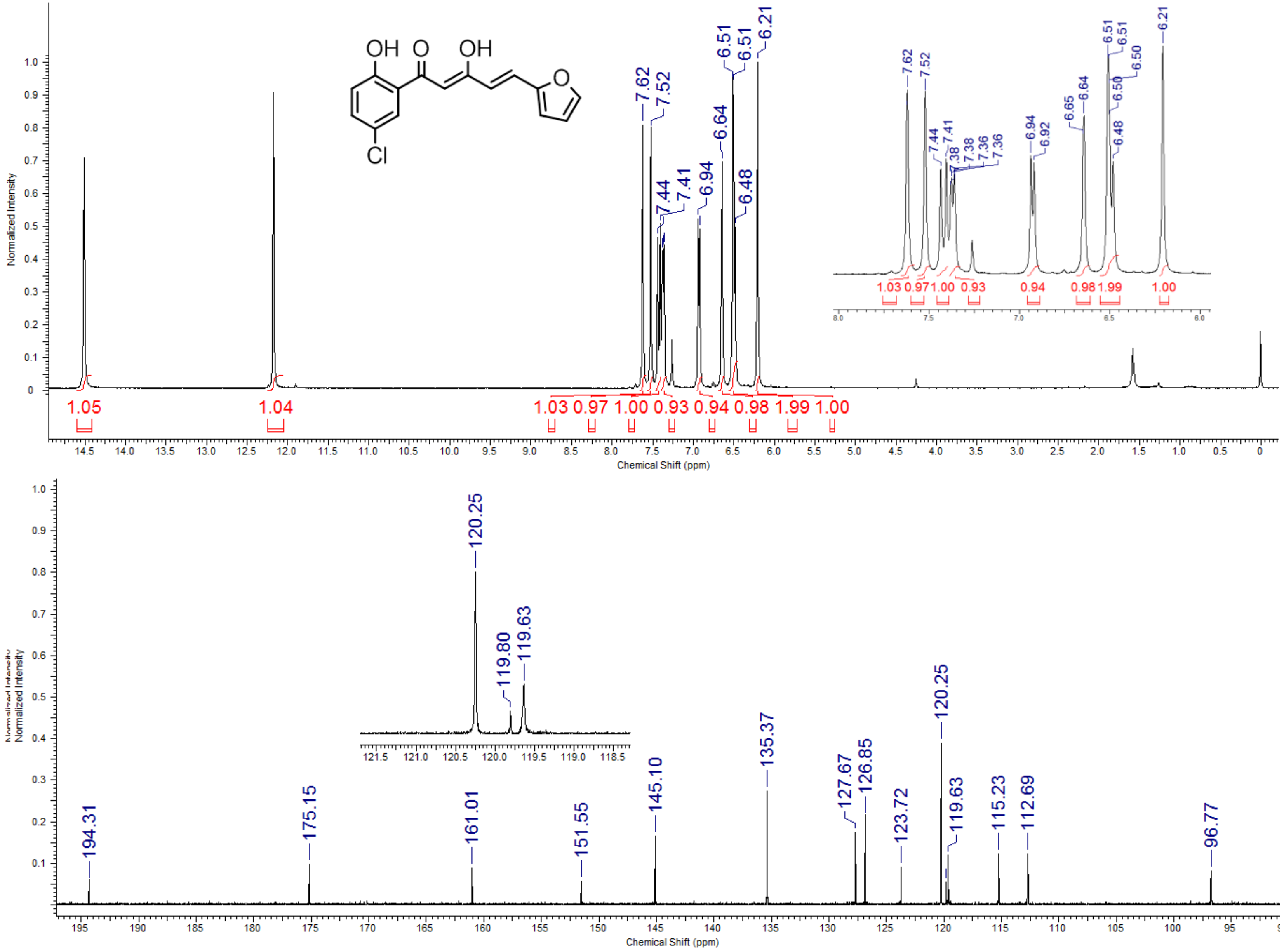

Figure S32. NMR spectra of compound **32**.

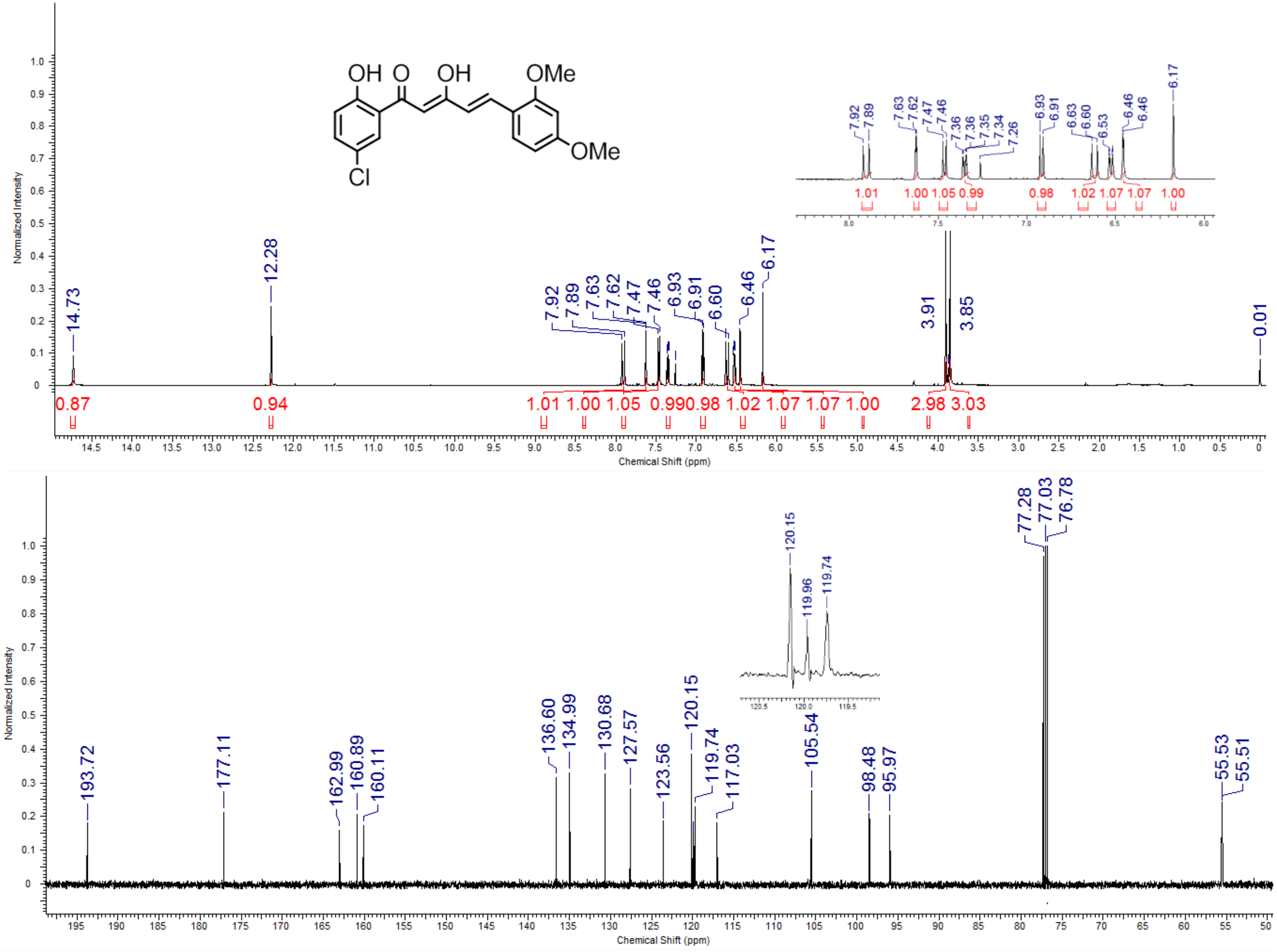

Figure S33. NMR spectra of compound **33**.

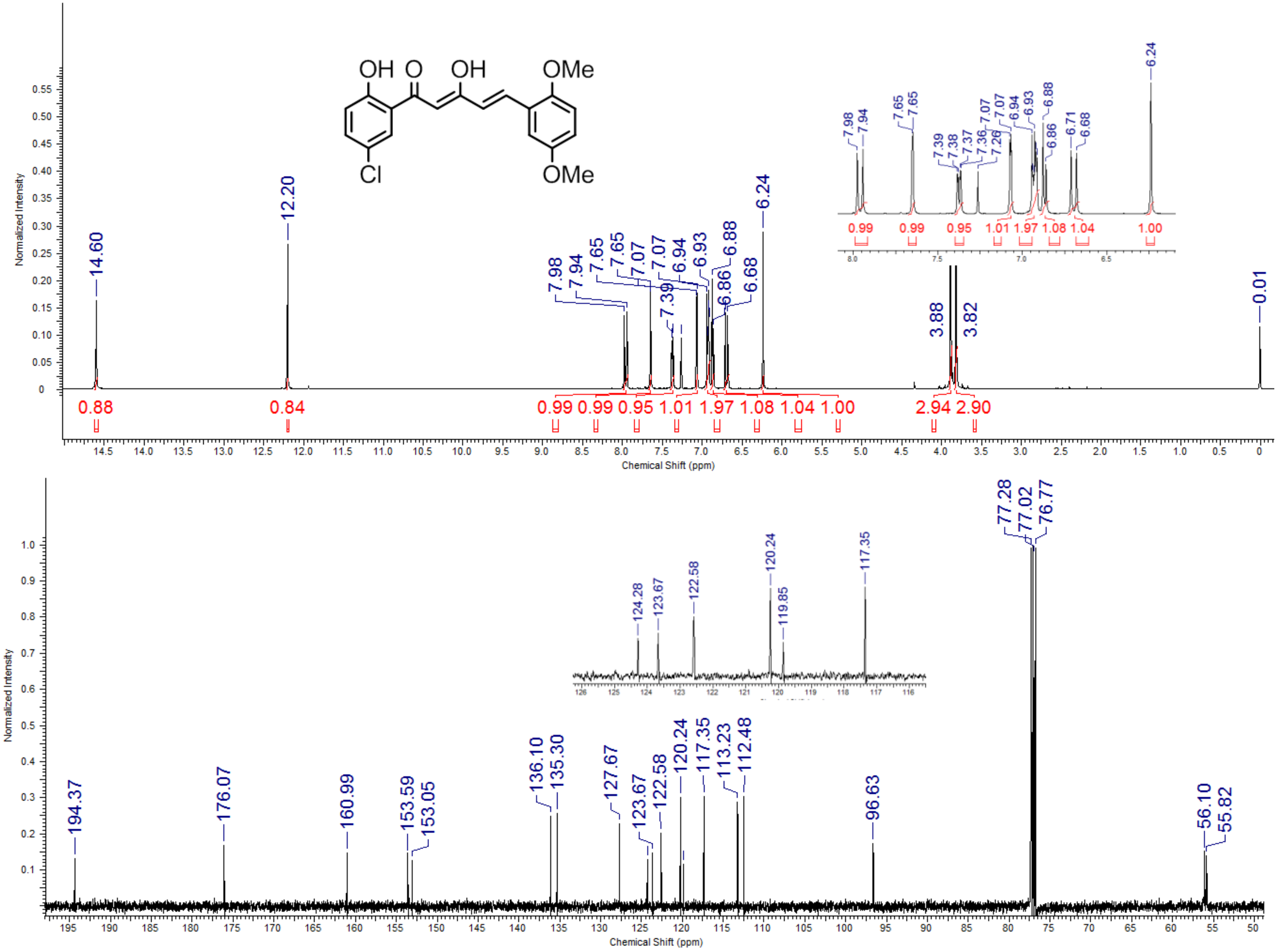

Figure S34. NMR spectra of compound **34**.

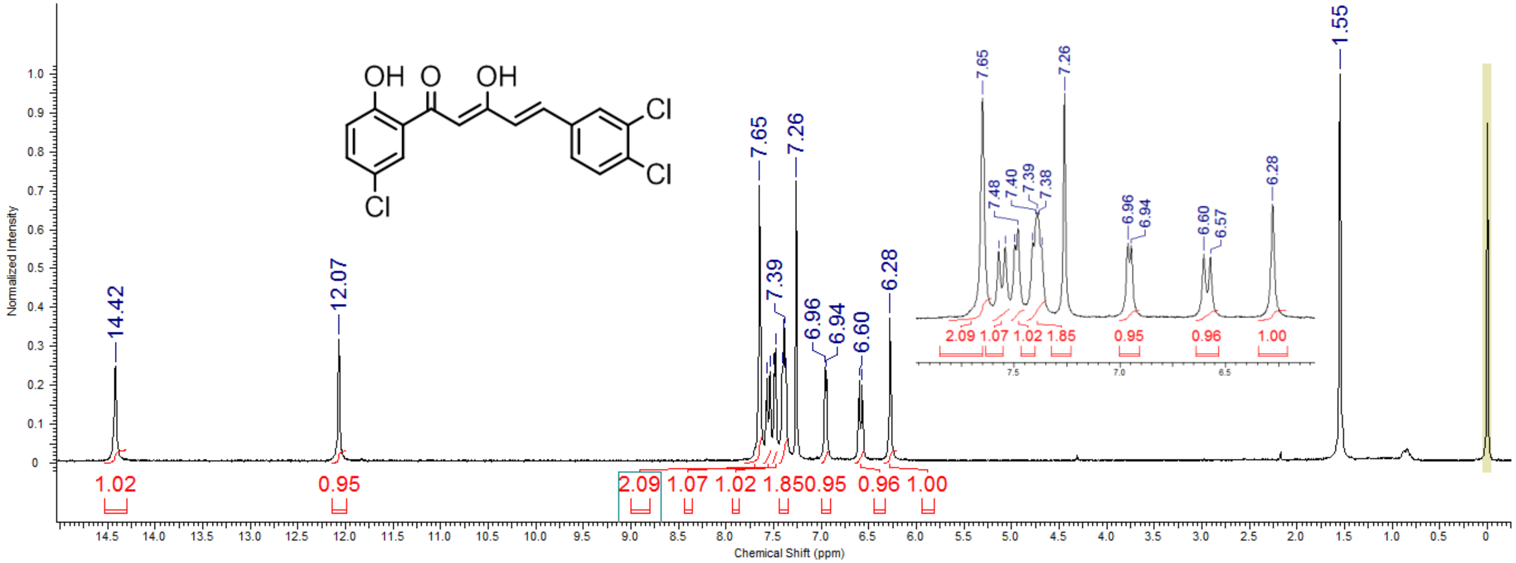

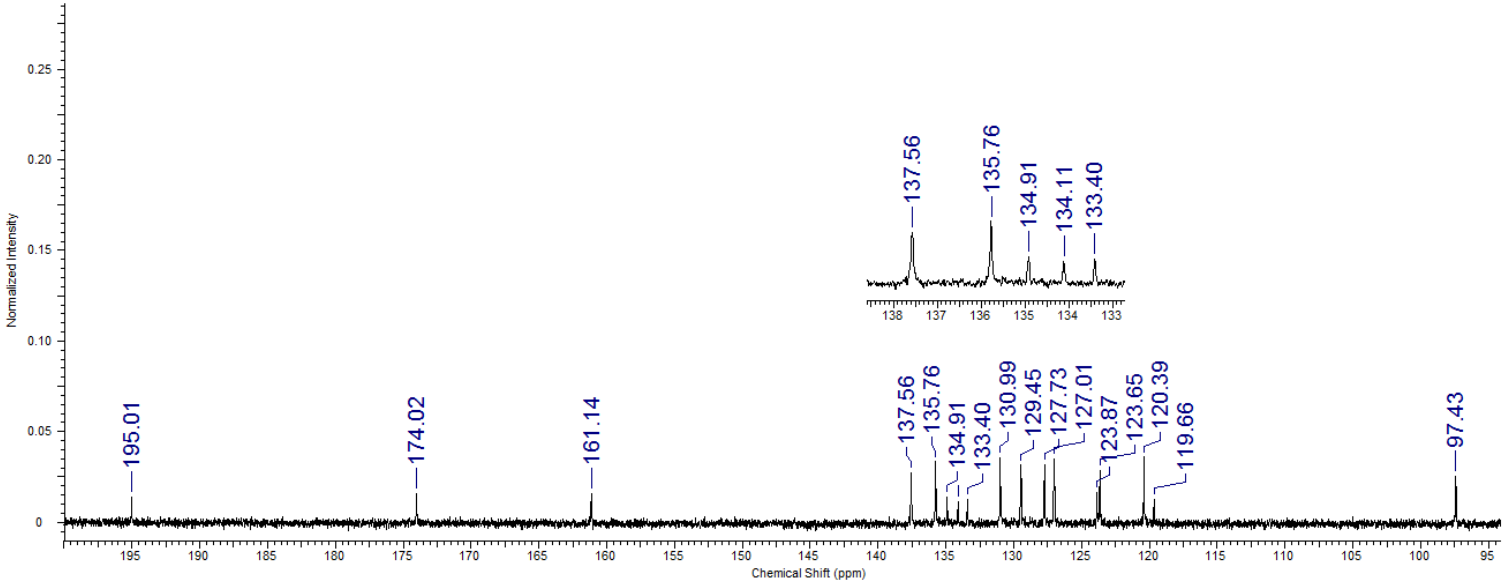

Figure S35. NMR spectra of compound **35**.

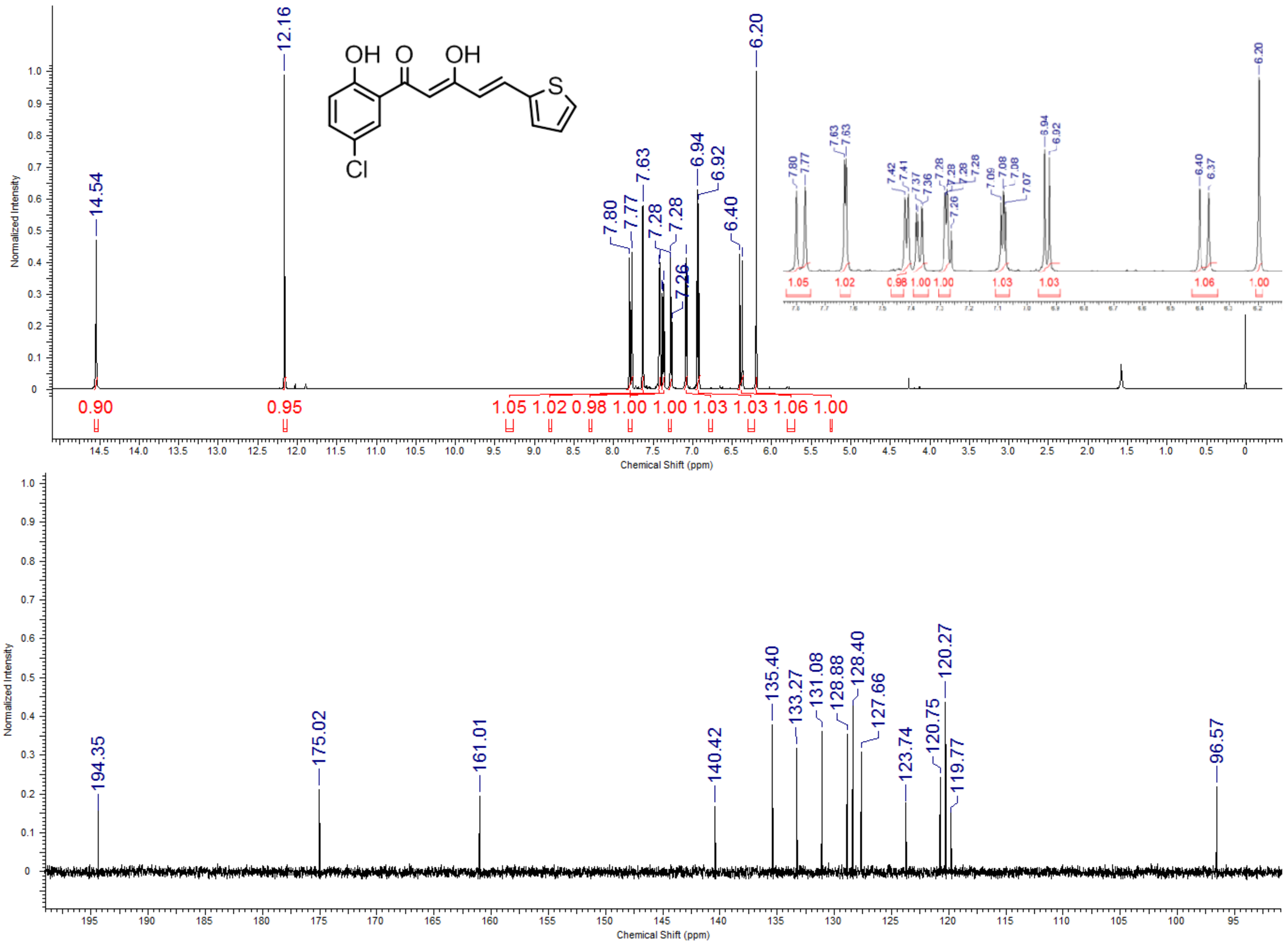

Figure S36. NMR spectra of compound **36**.

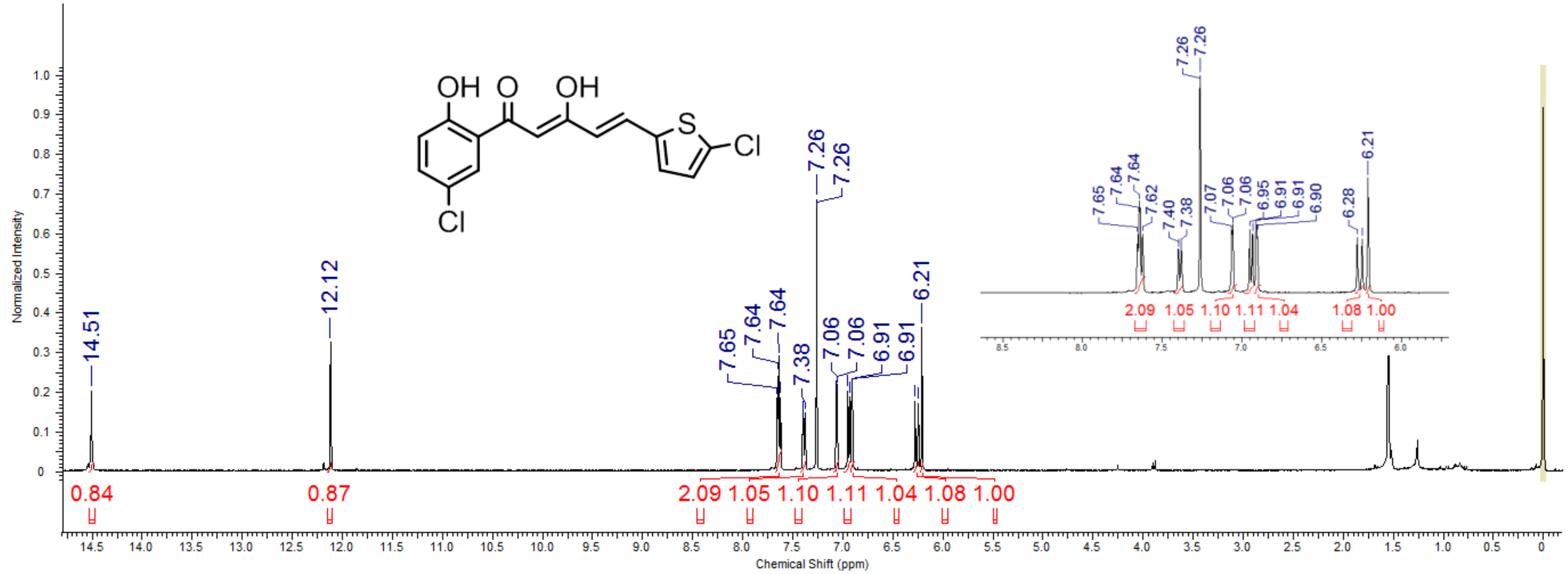

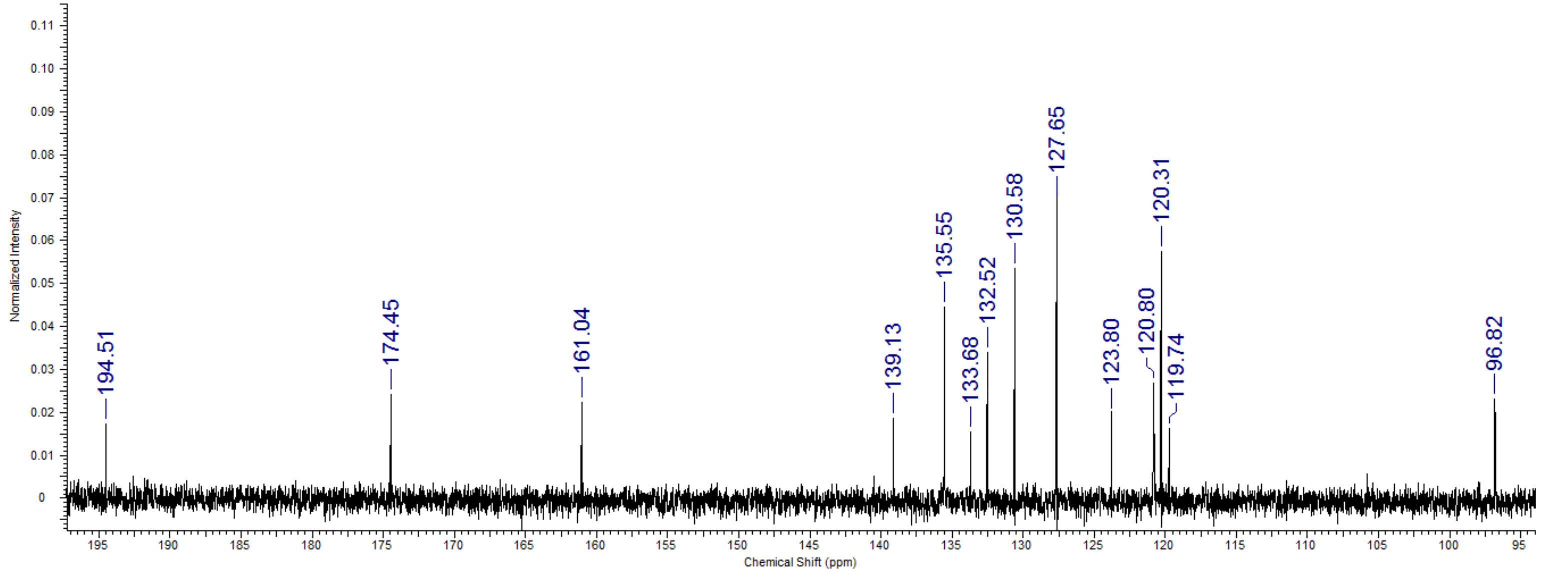

Figure S37. NMR spectra of compound **37**.

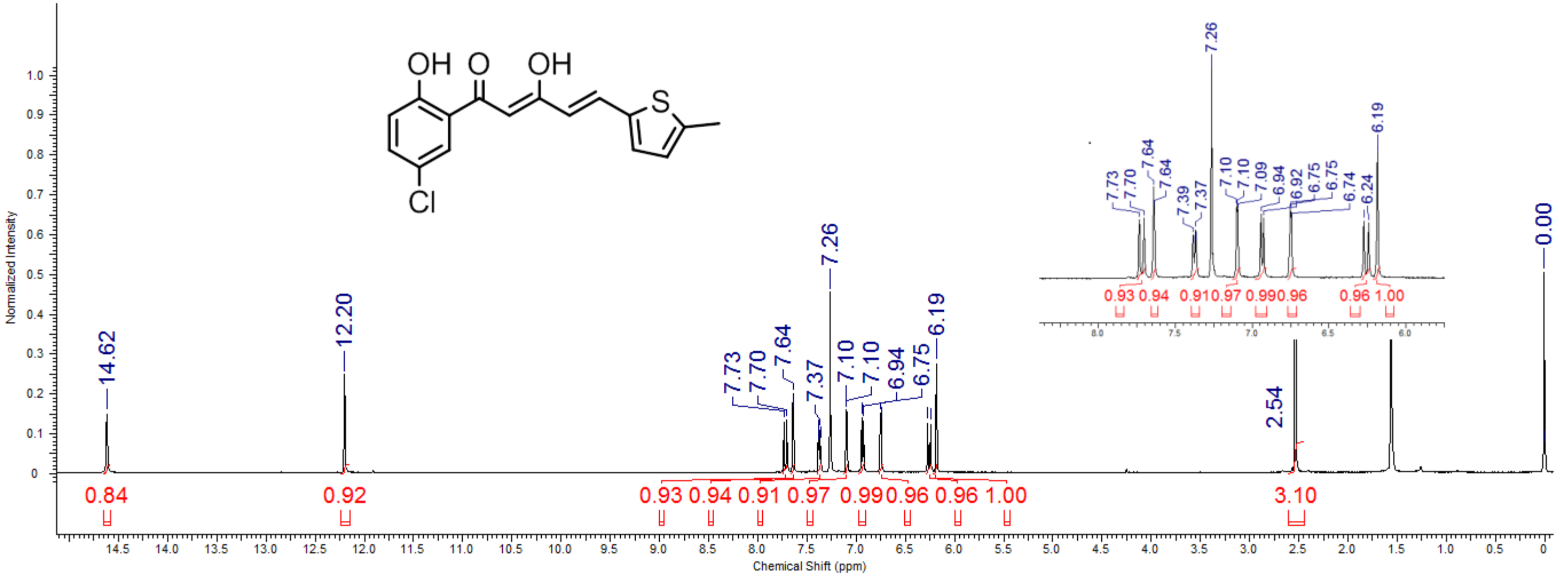

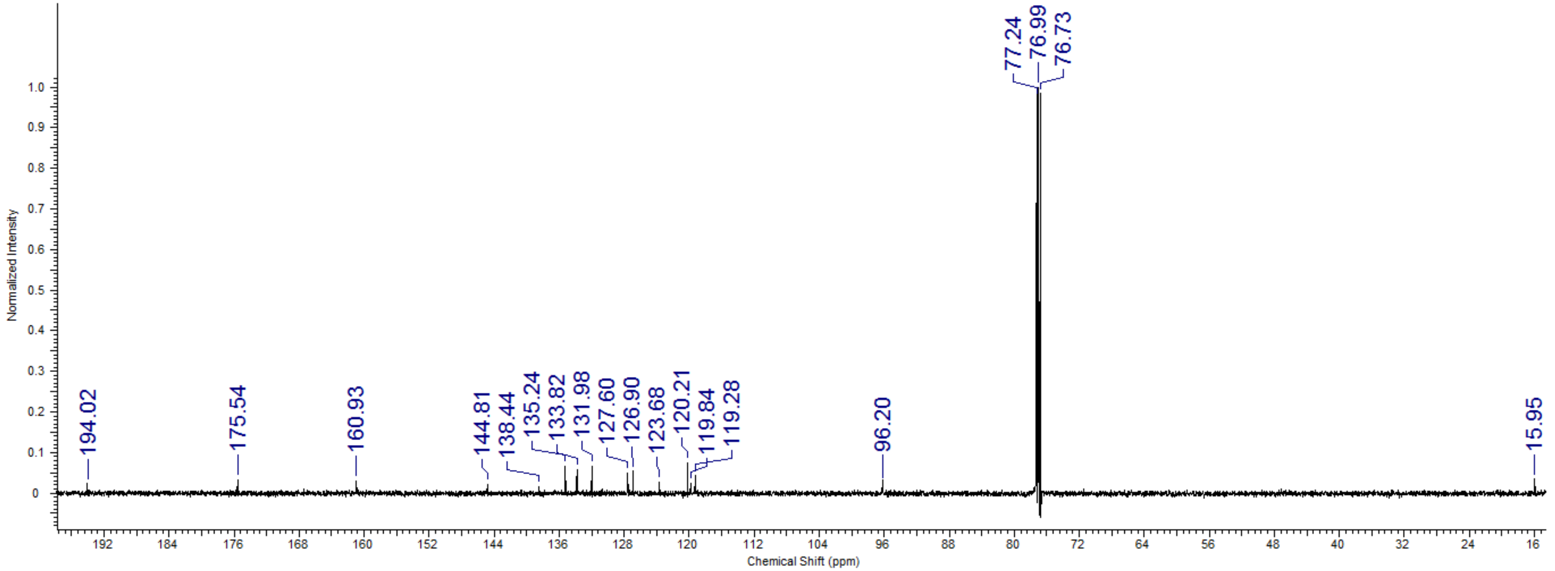

Figure S38. NMR spectra of compound **38**.

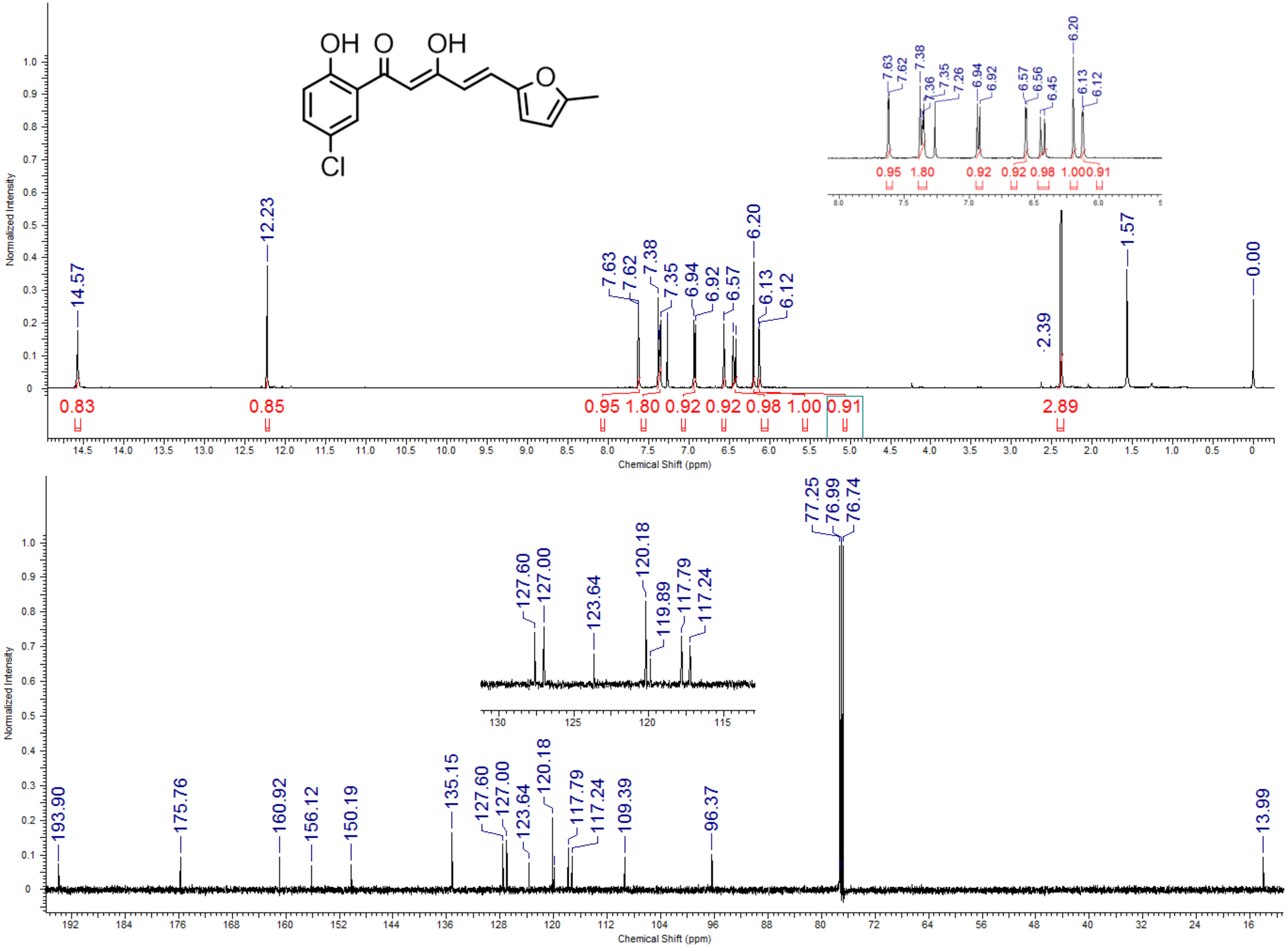

Figure S39. NMR spectra of compound **39**.

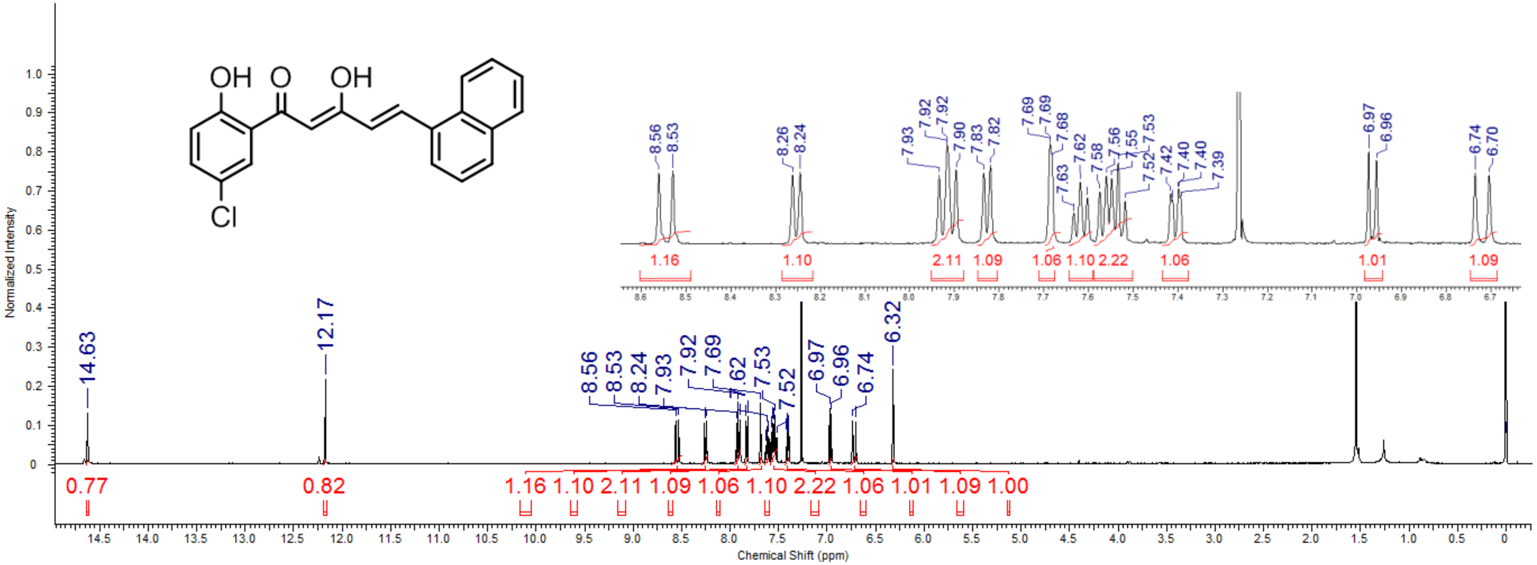

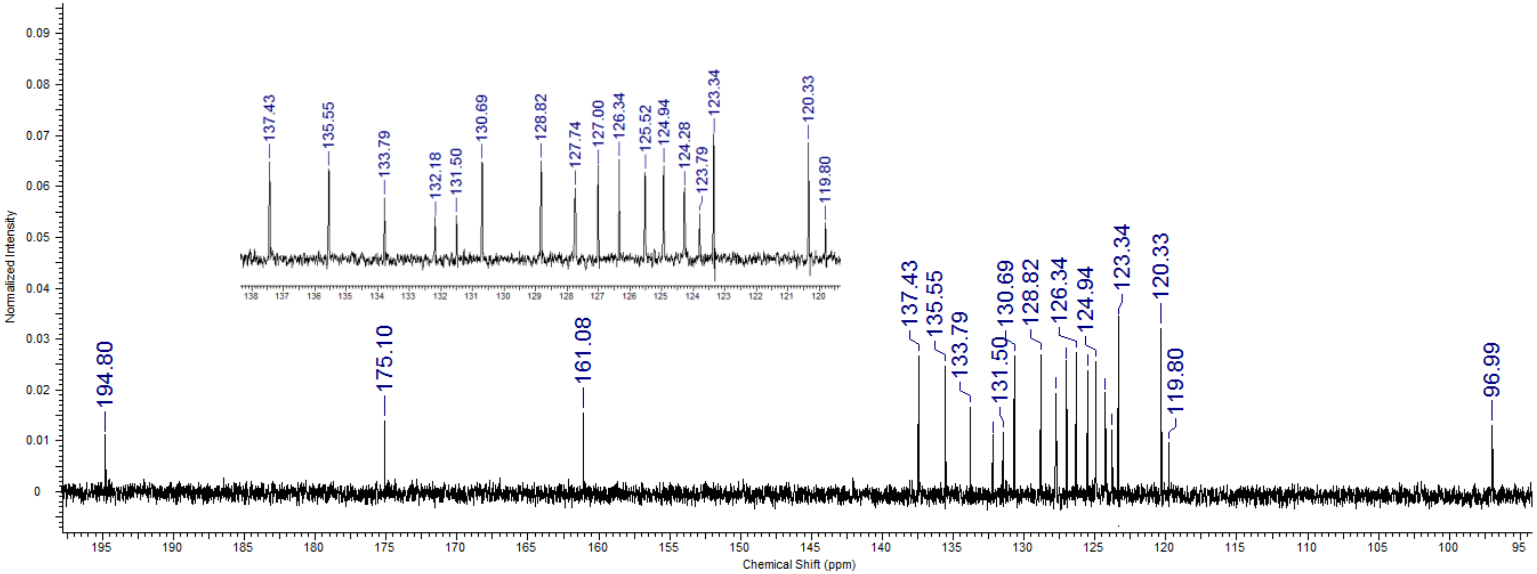

Figure S40. NMR spectra of compound **40**.

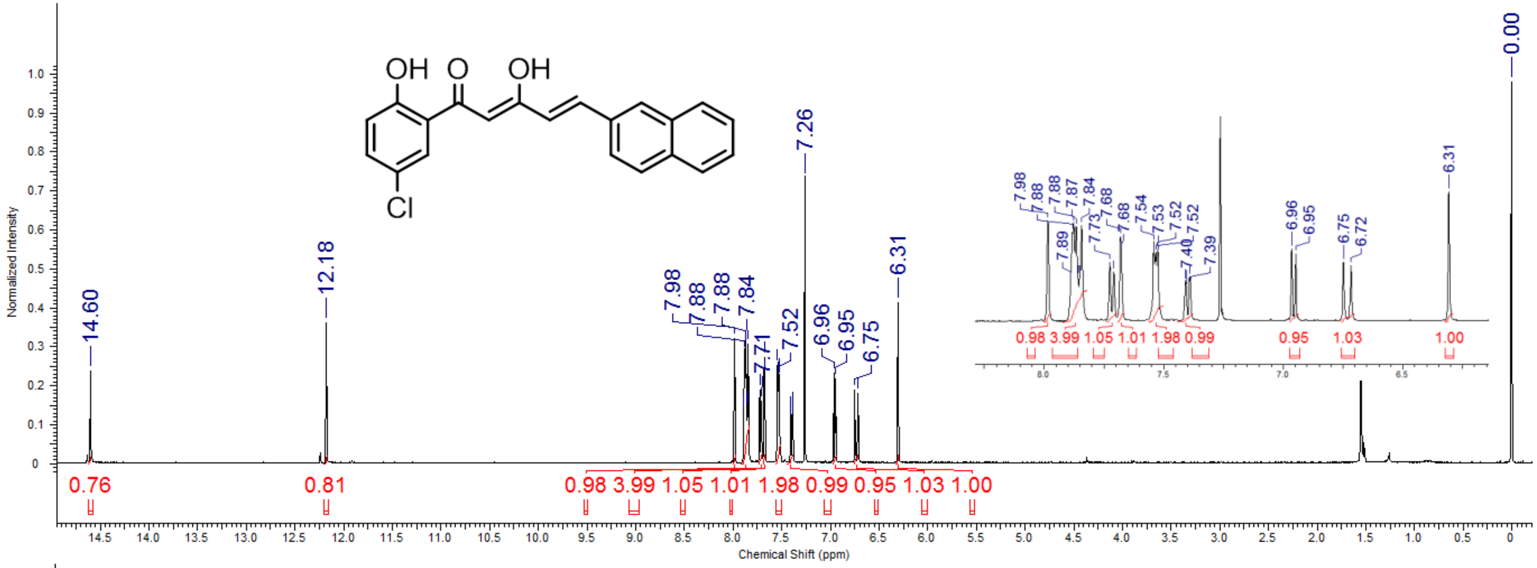

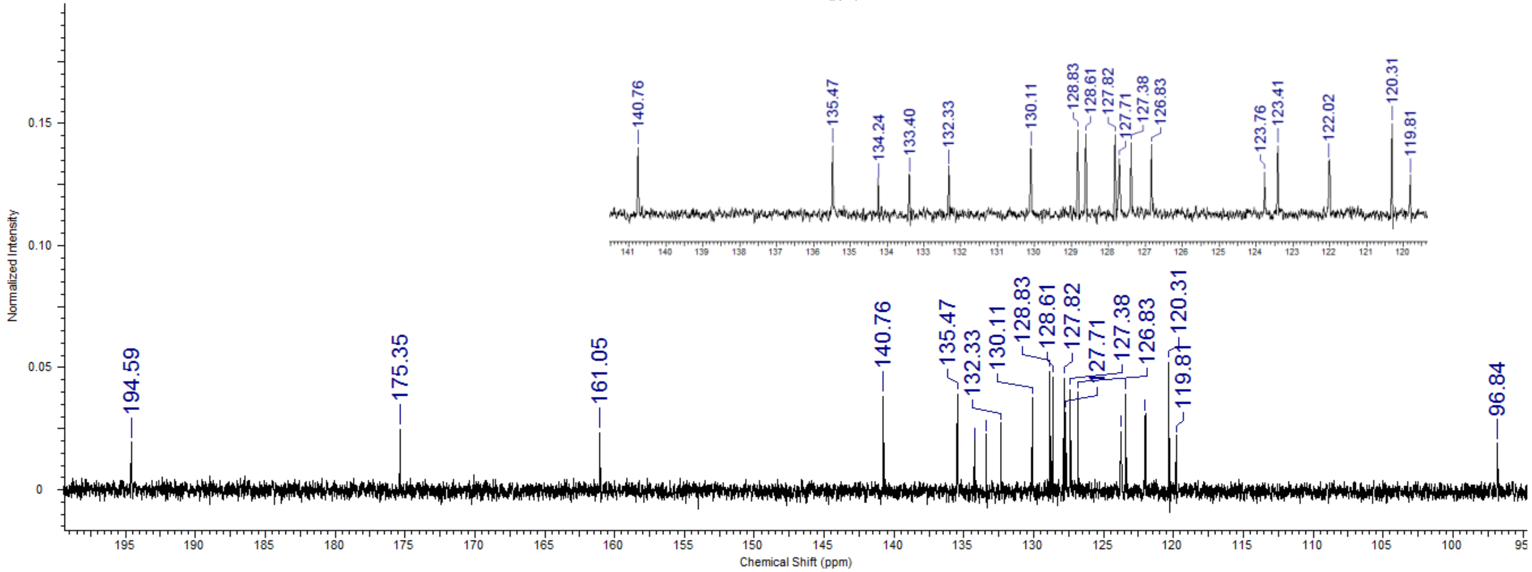

Figure S41. NMR spectra of compound **41**.

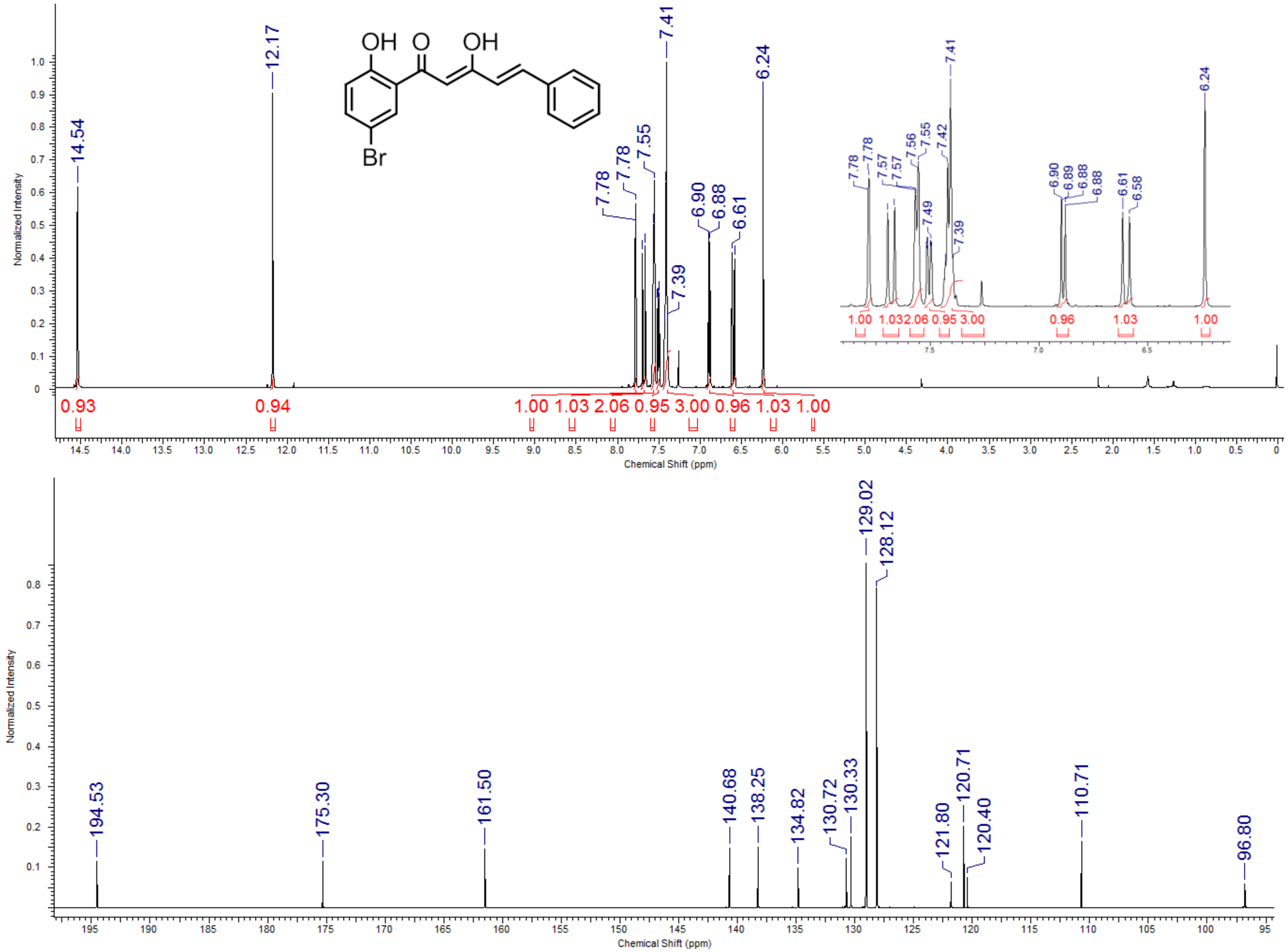

Figure S42. NMR spectra of compound **42**.

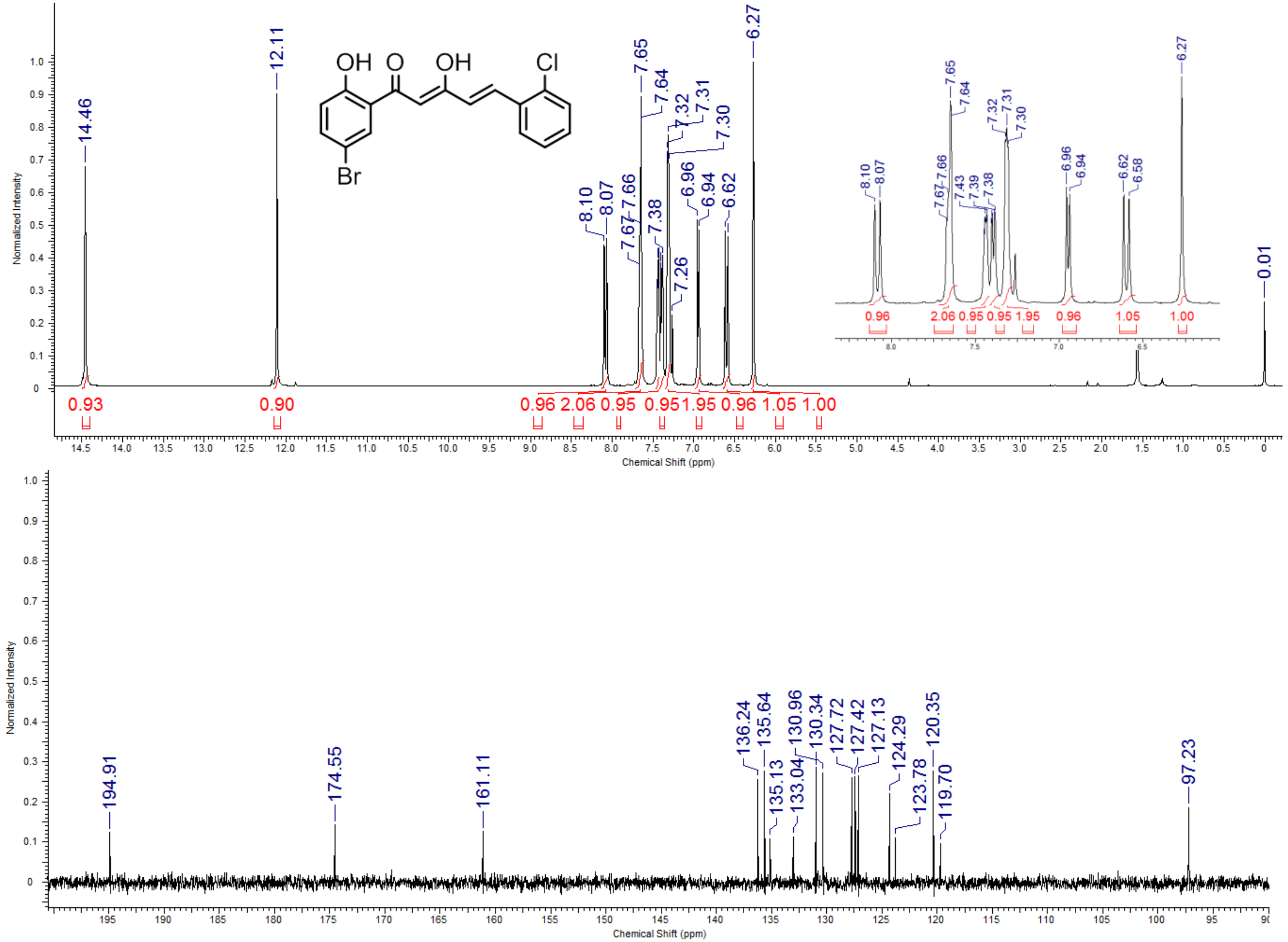

Figure S43. NMR spectra of compound **43**.

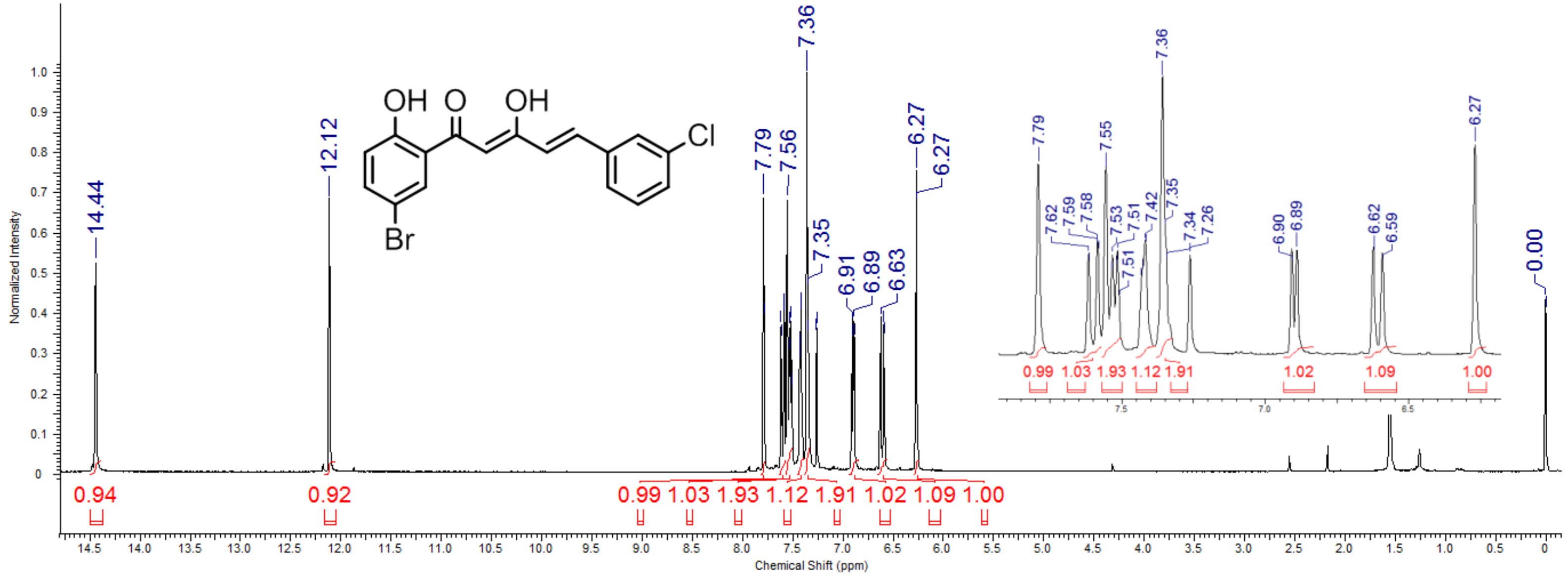

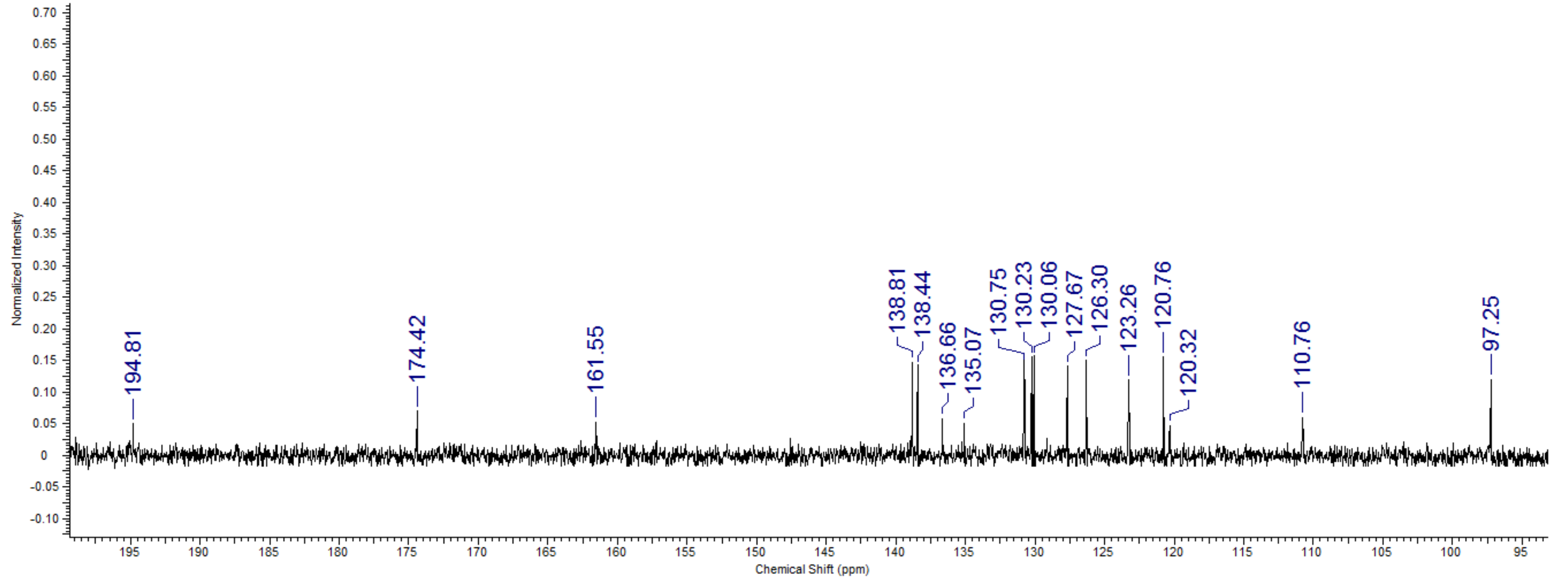

Figure S44. NMR spectra of compound **44**.

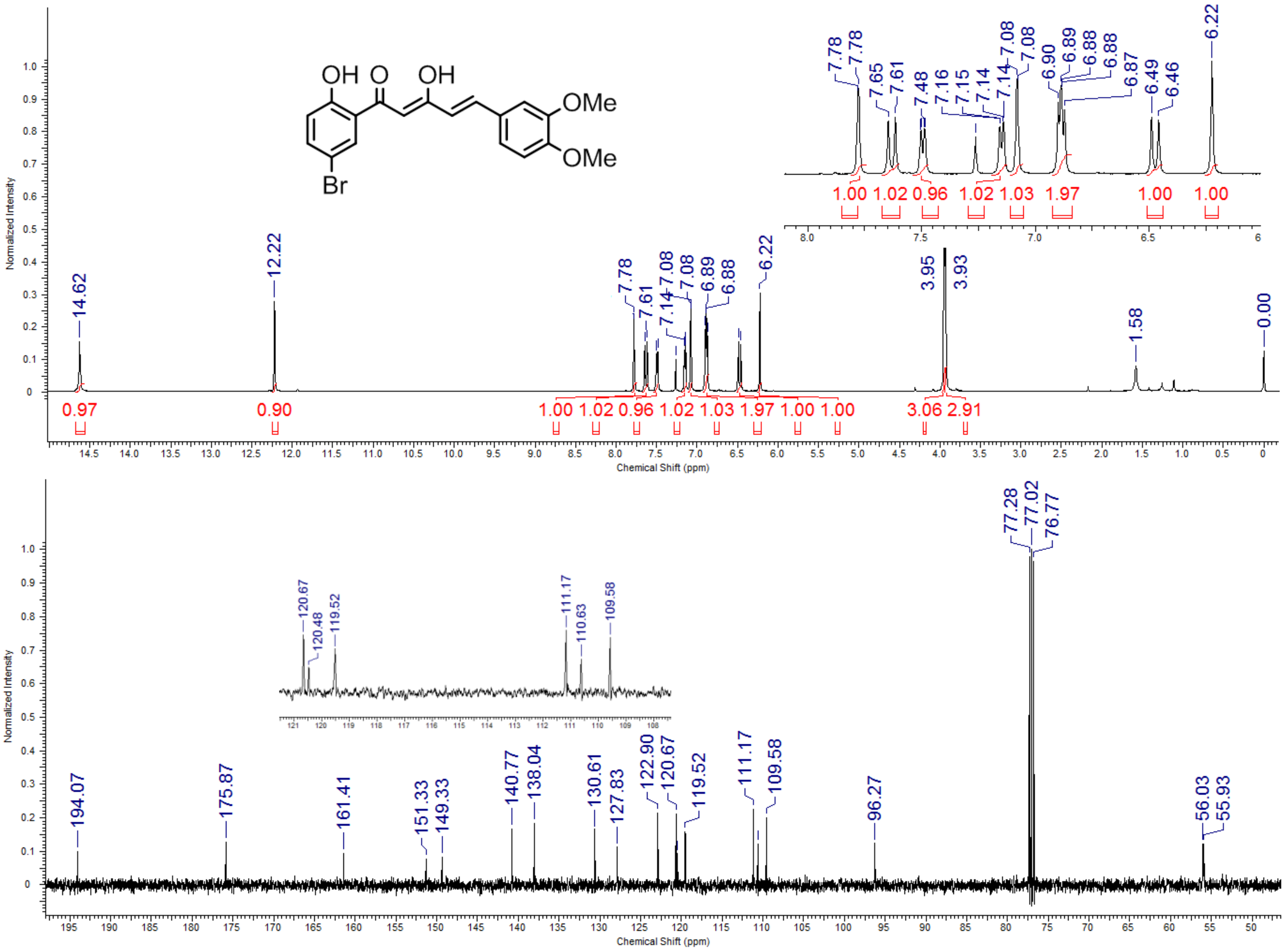

Figure S45. NMR spectra of compound **45**.

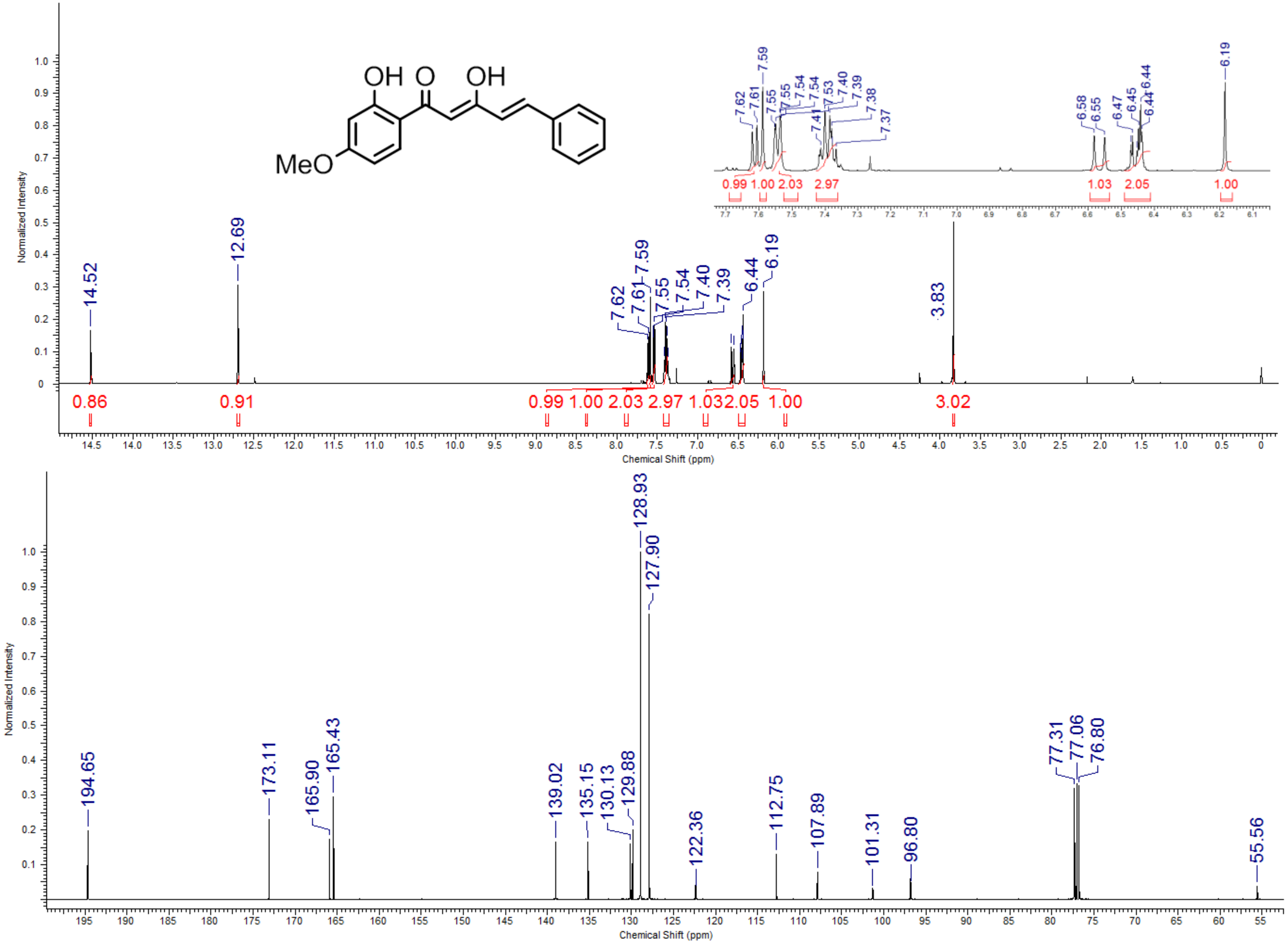

Figure S46. NMR spectra of compound **46**.

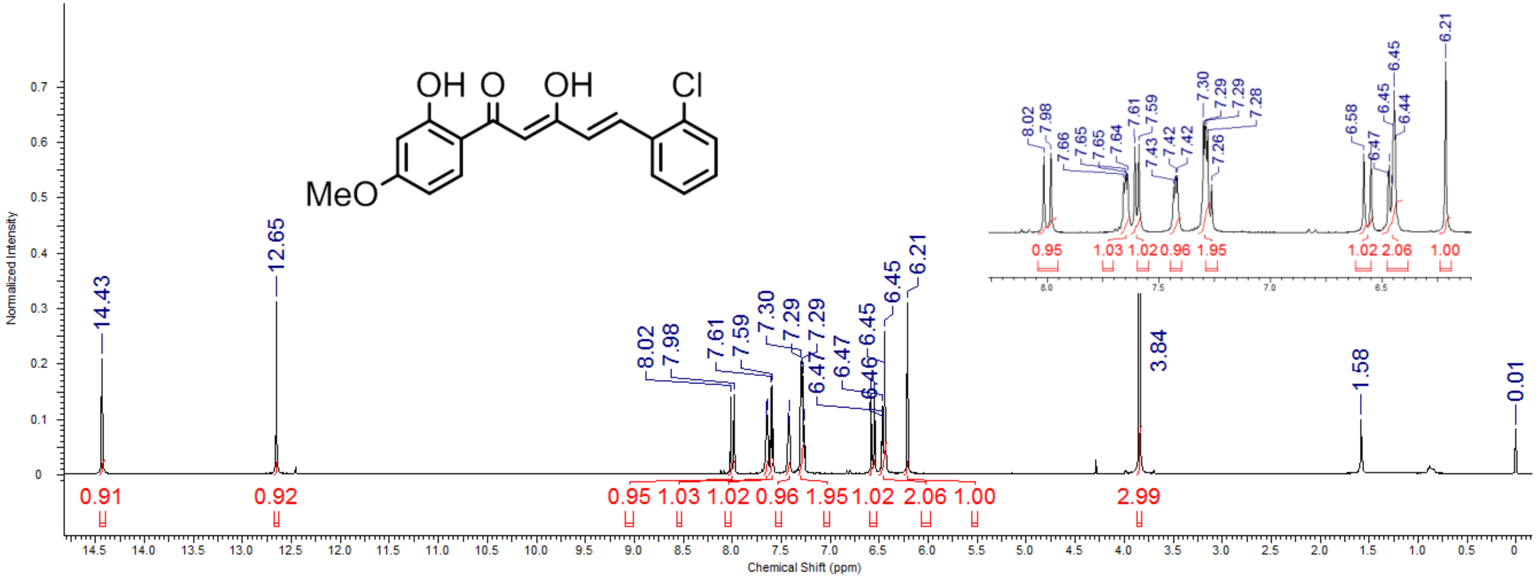

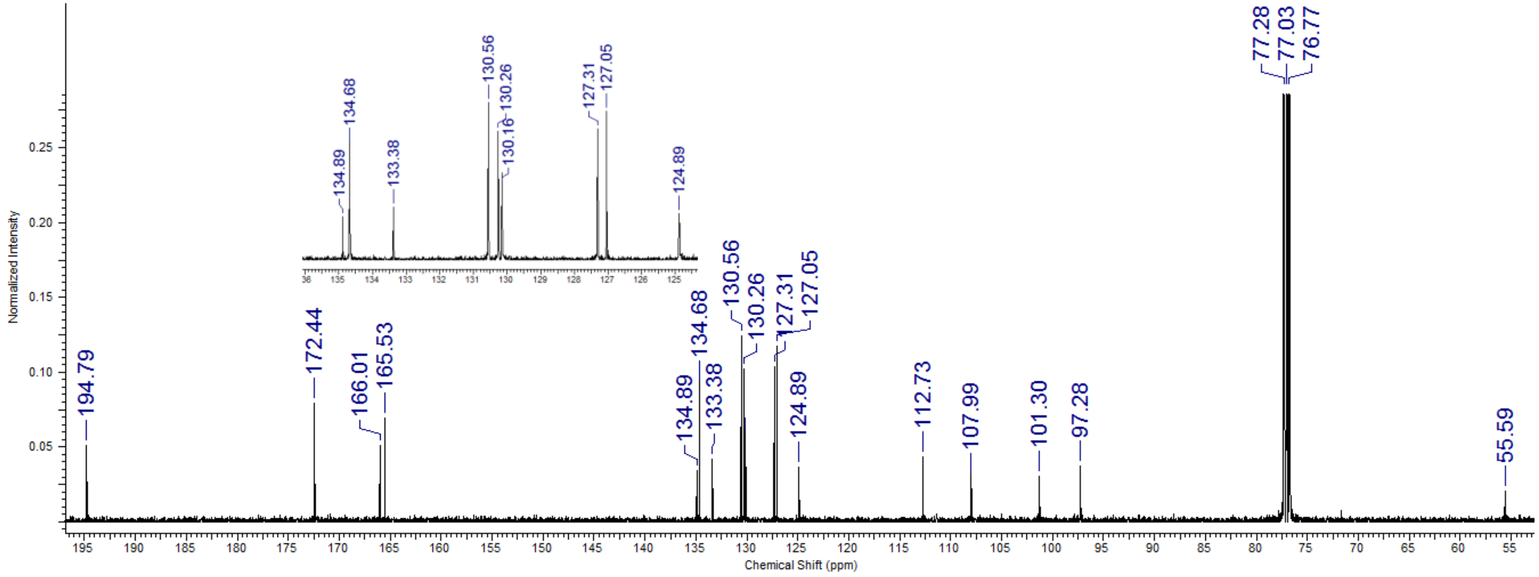

Figure S47. NMR spectra of compound **47**.

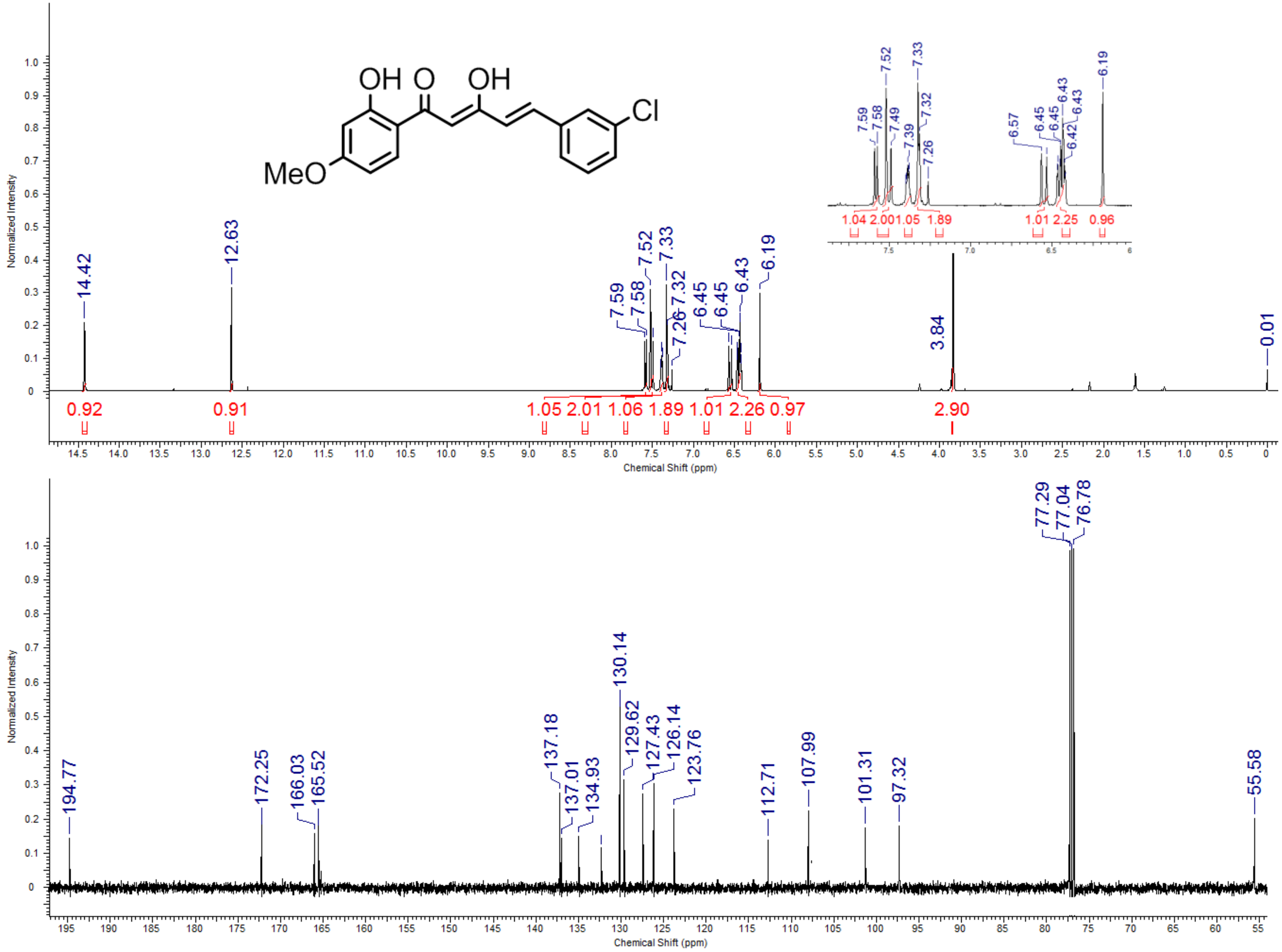

Figure S48. NMR spectra of compound **48**.

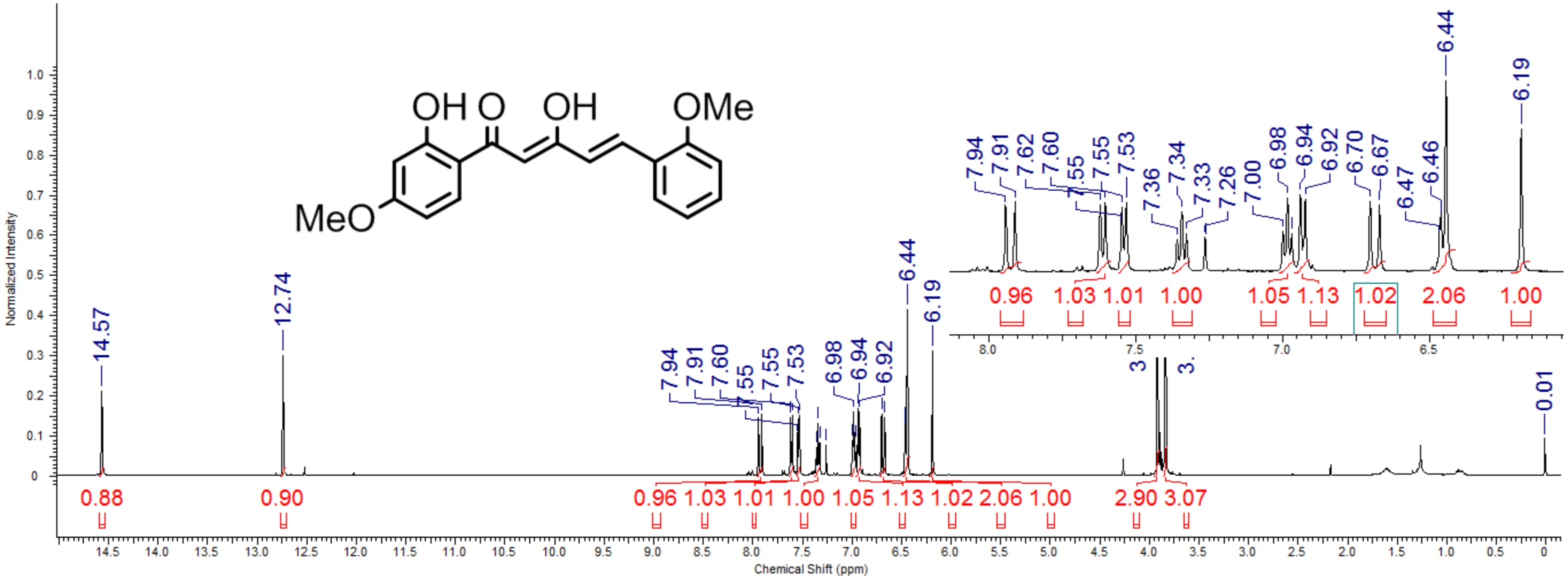

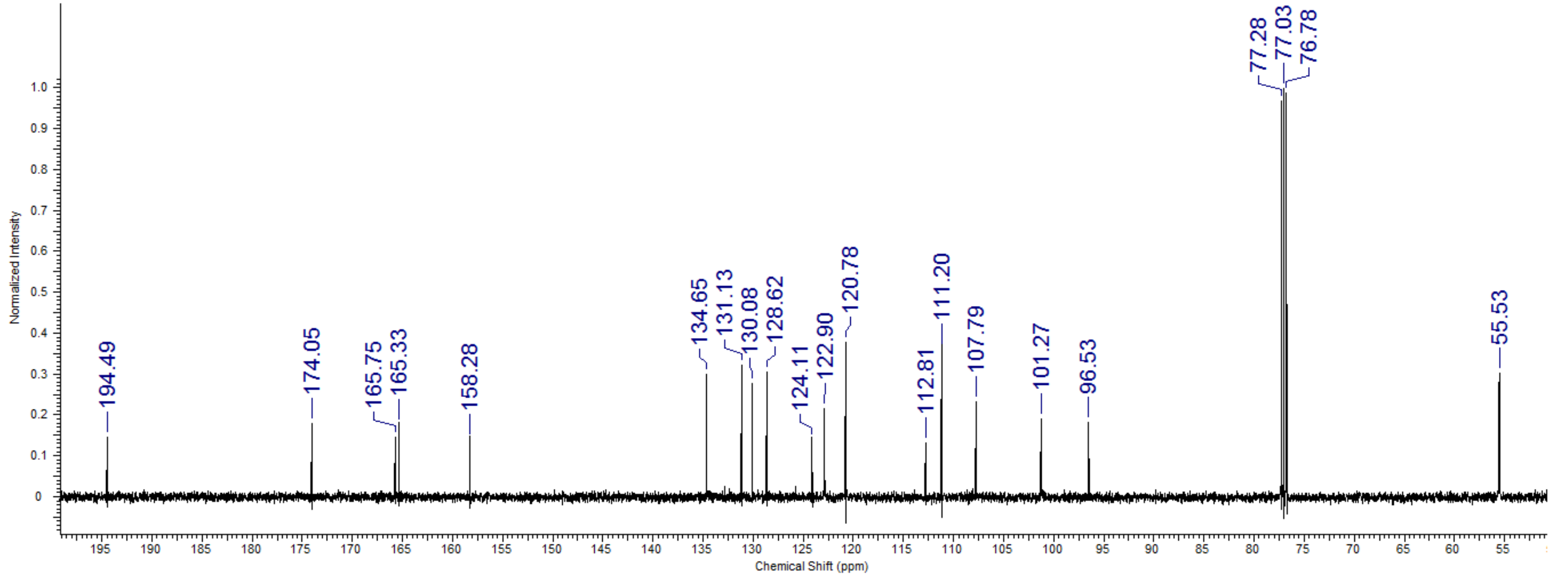

Figure S49. NMR spectra of compound **49**.

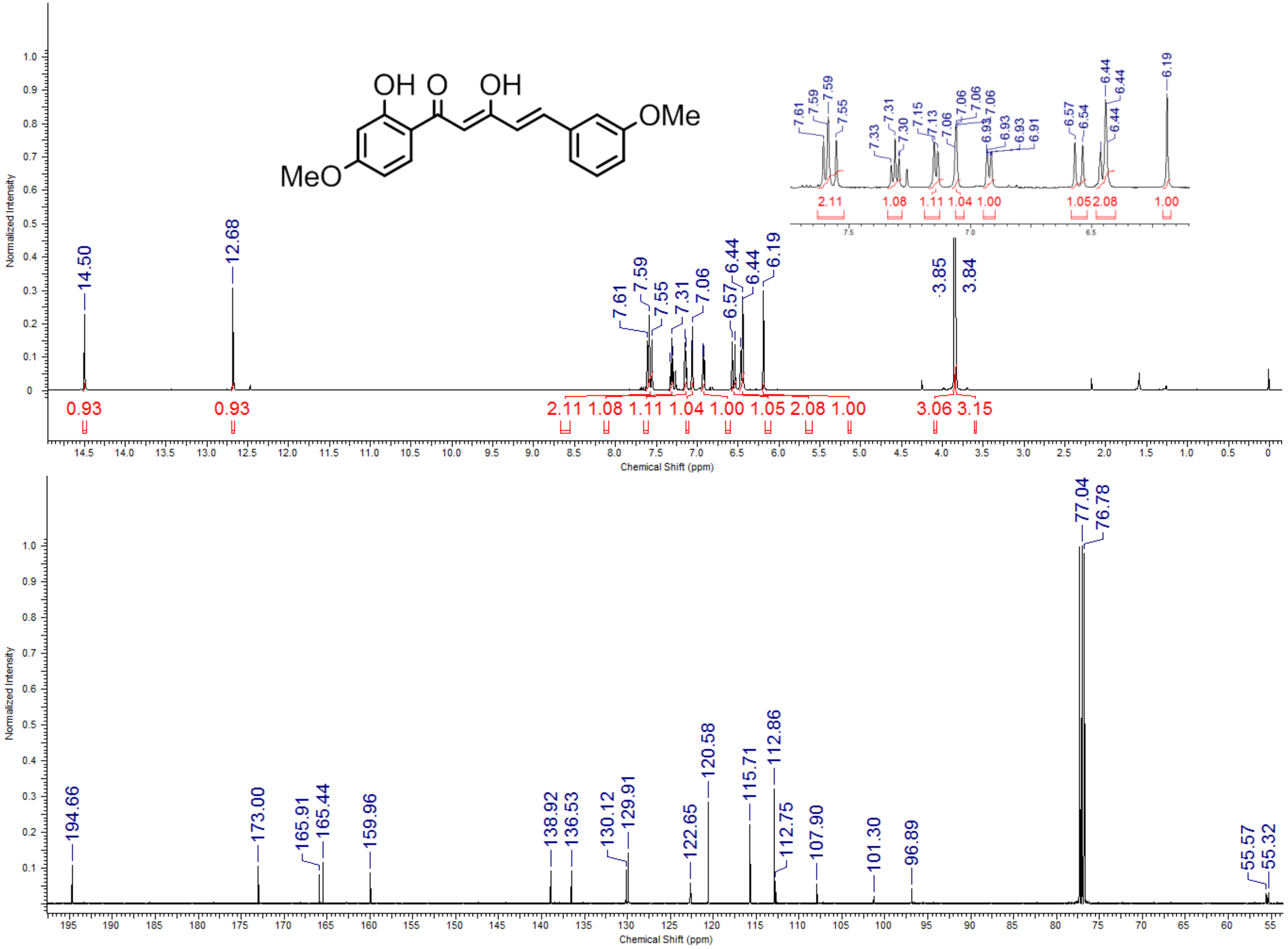

Figure S50. NMR spectra of compound **50**.

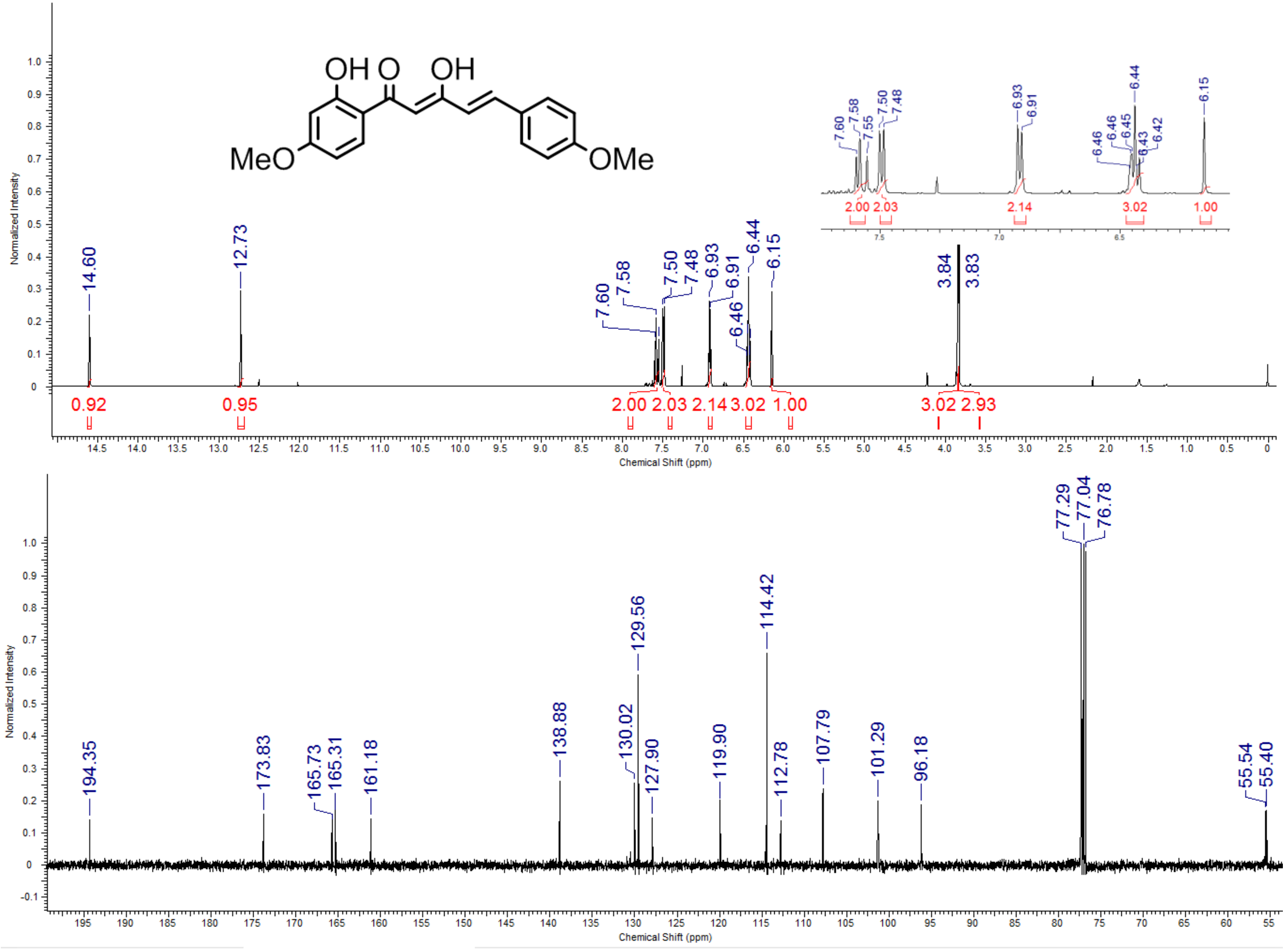

Figure S51. NMR spectra of compound **51**.

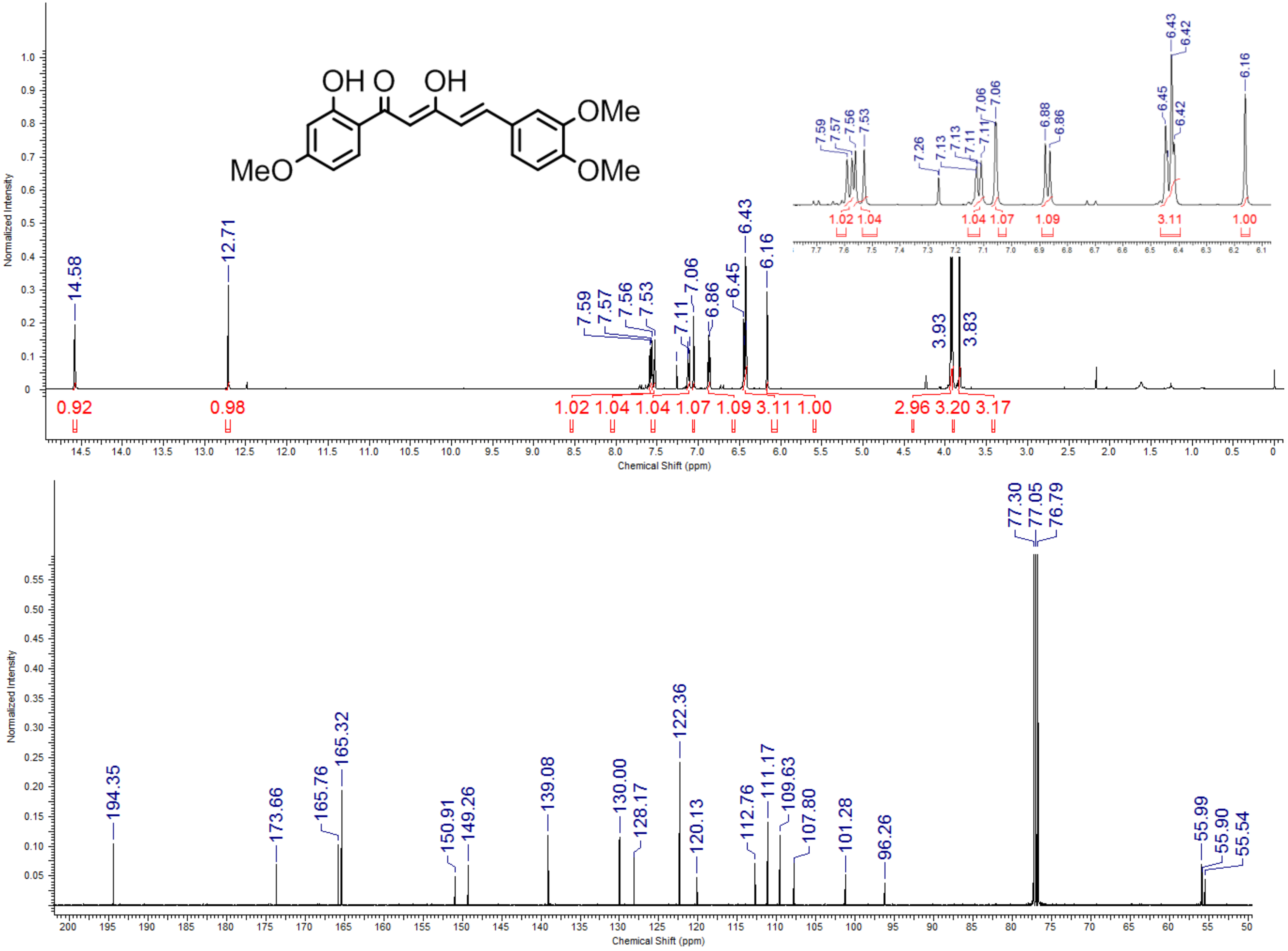

Figure S52. NMR spectra of compound **52**.

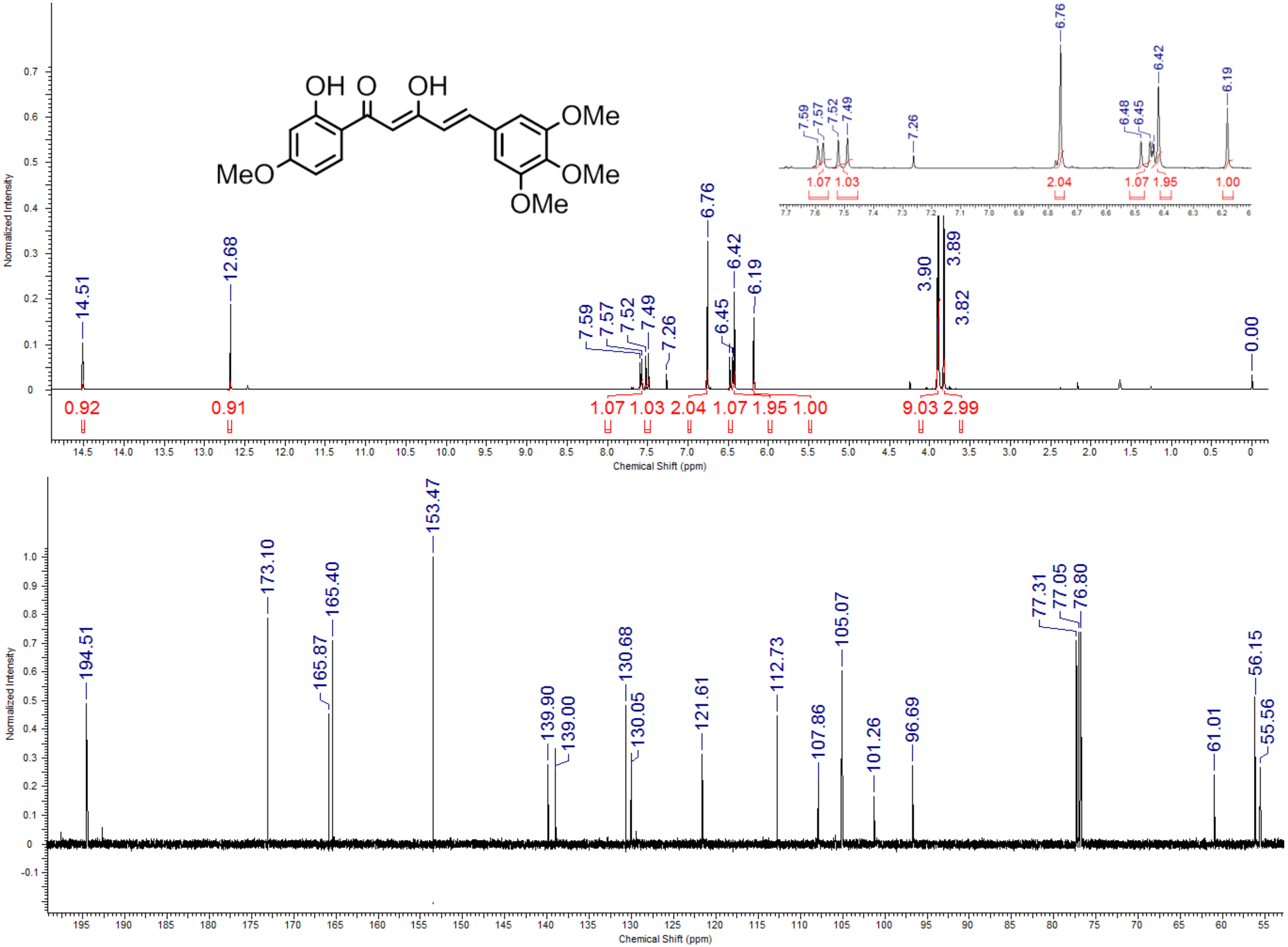

Figure S53. NMR spectra of compound **53**.

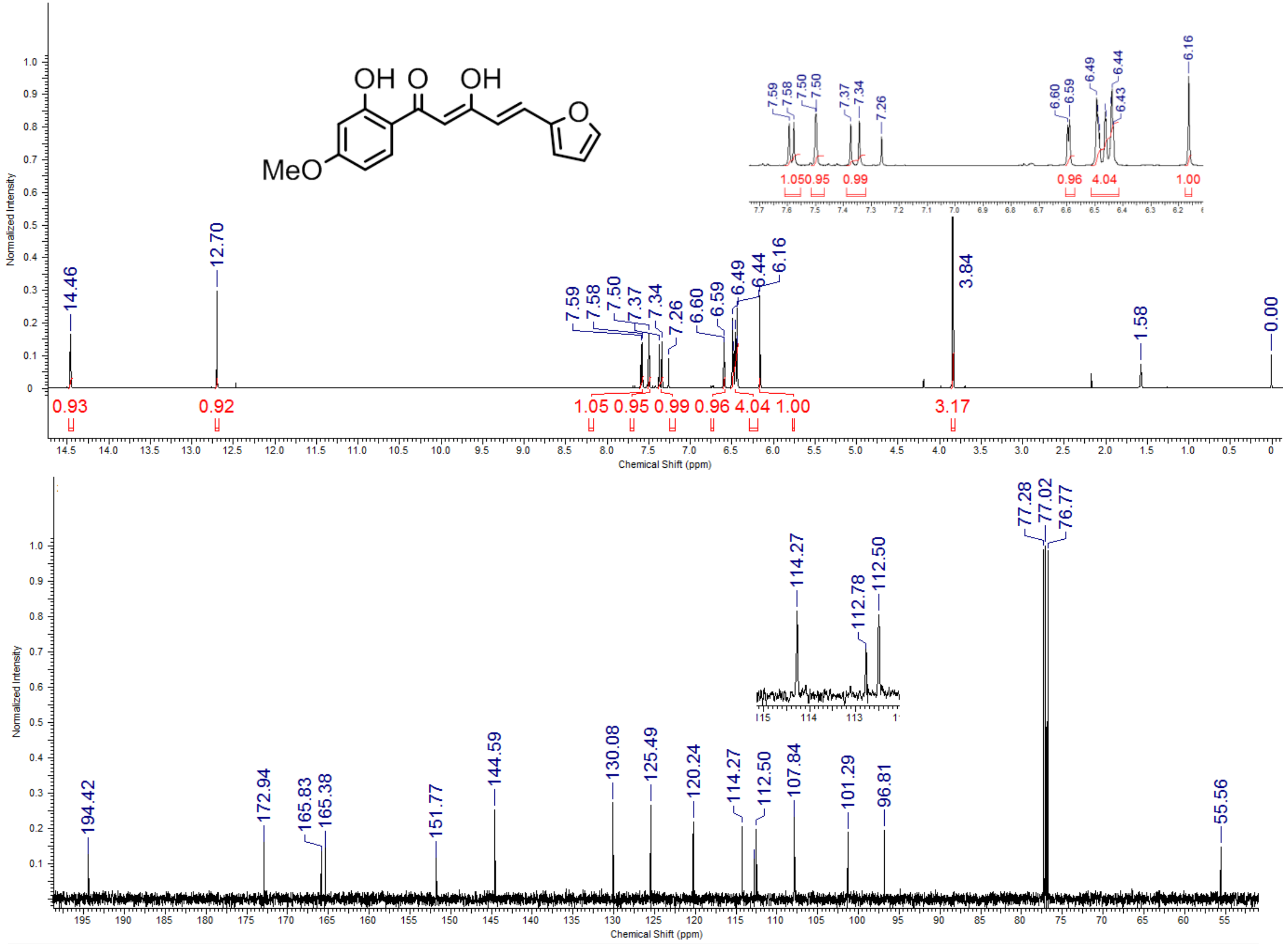

Figure S54. NMR spectra of compound **54**.

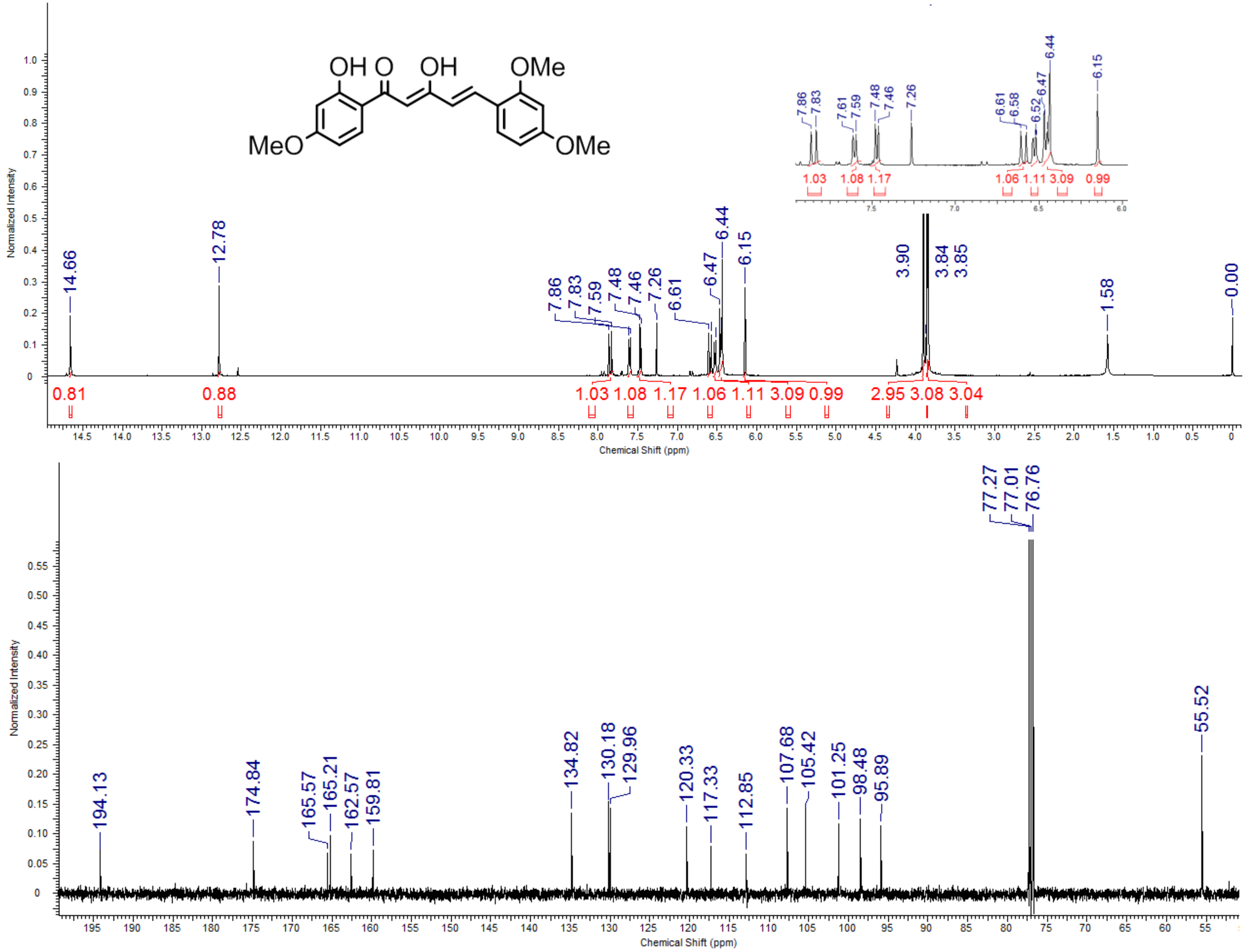

Figure S55. NMR spectra of compound **55**.

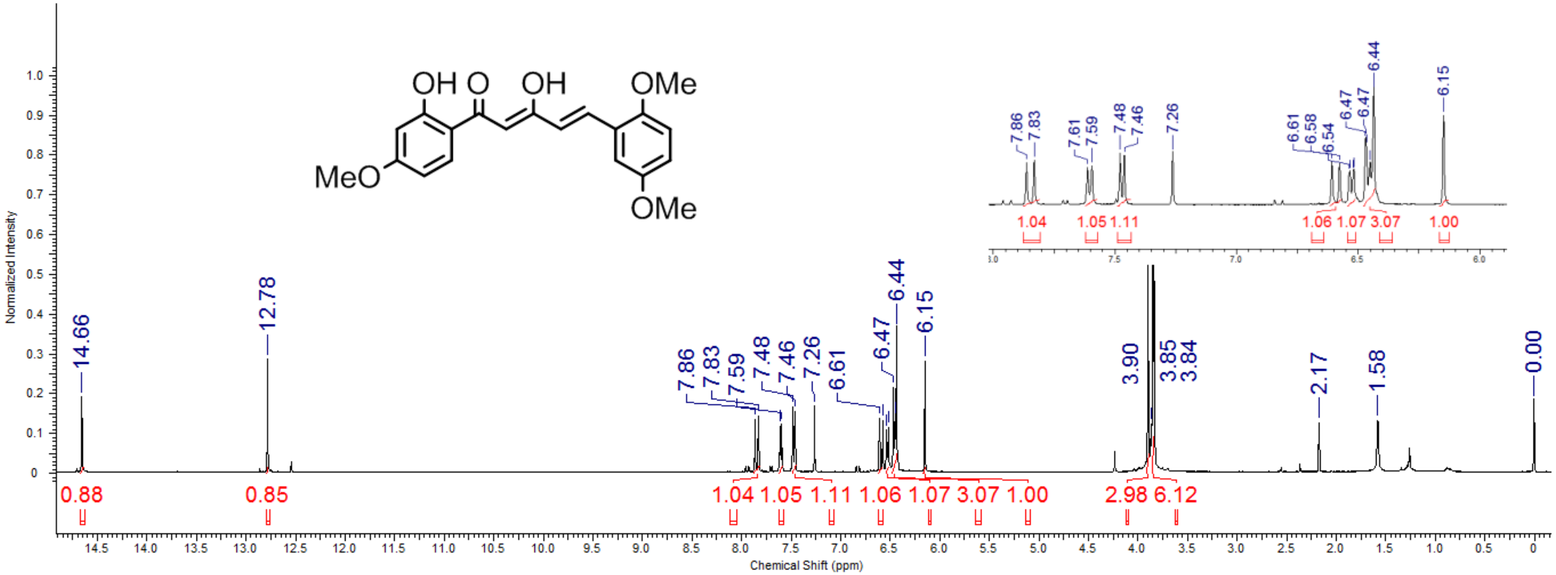

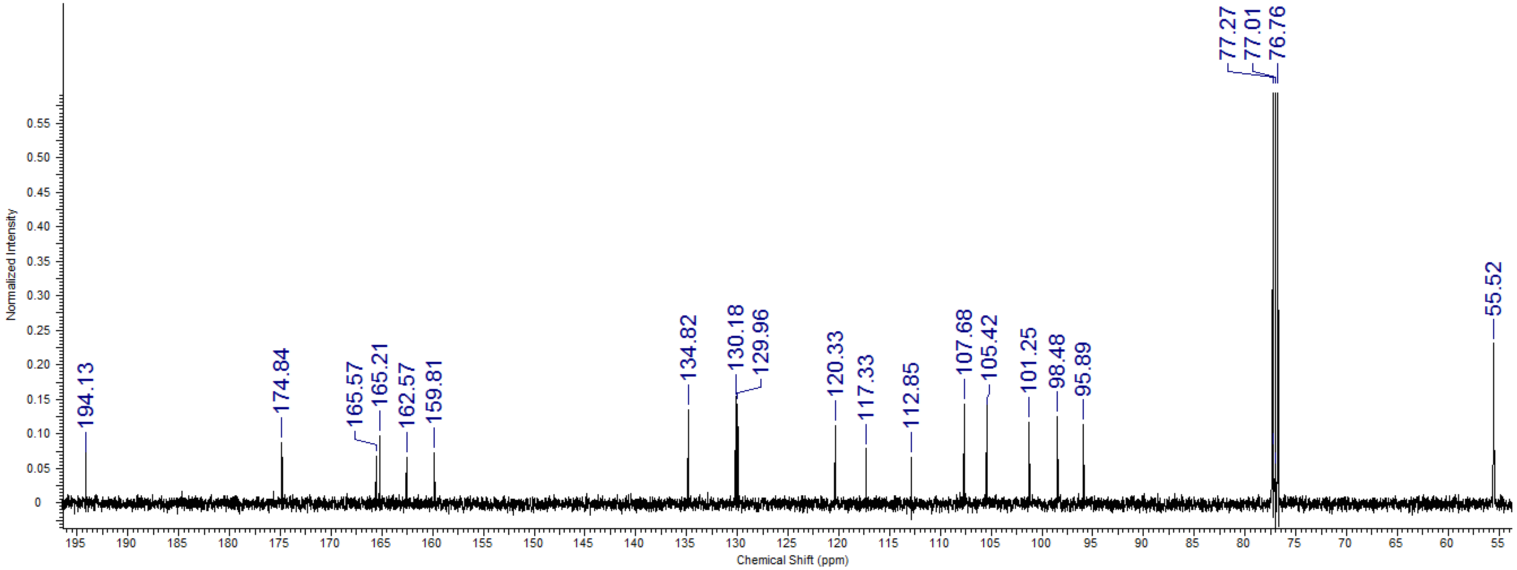

Figure S56. NMR spectra of compound **56**.

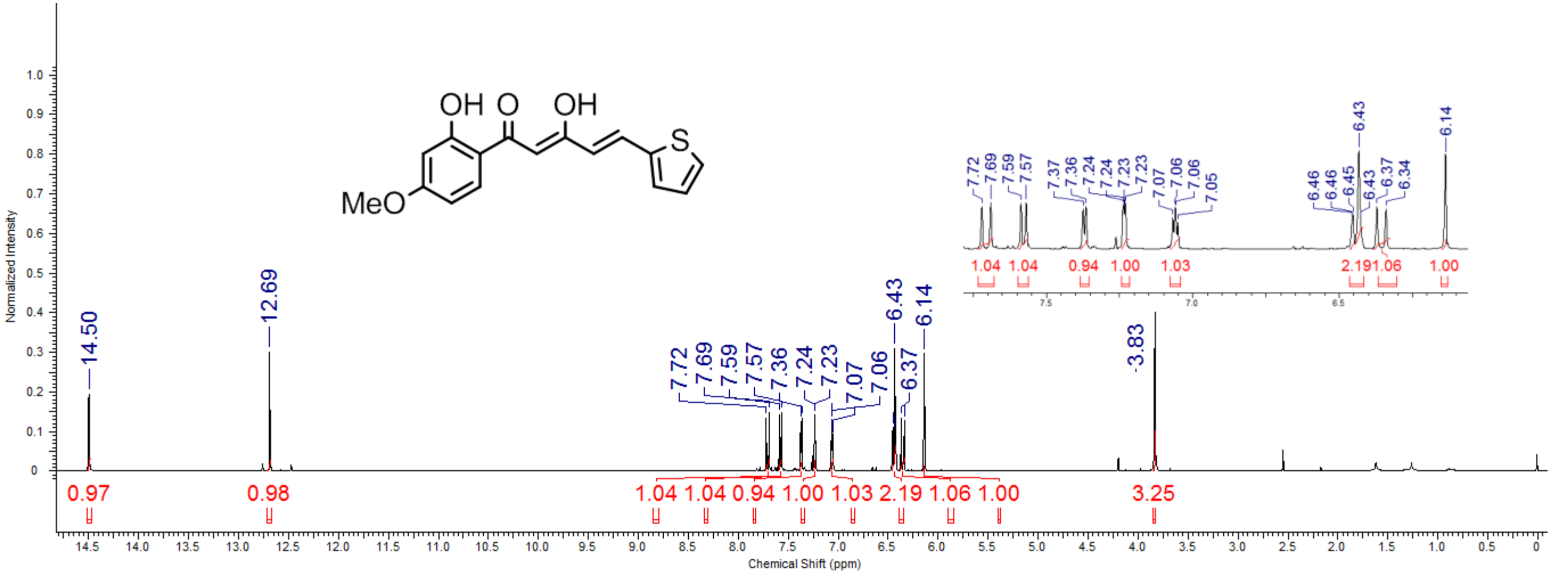

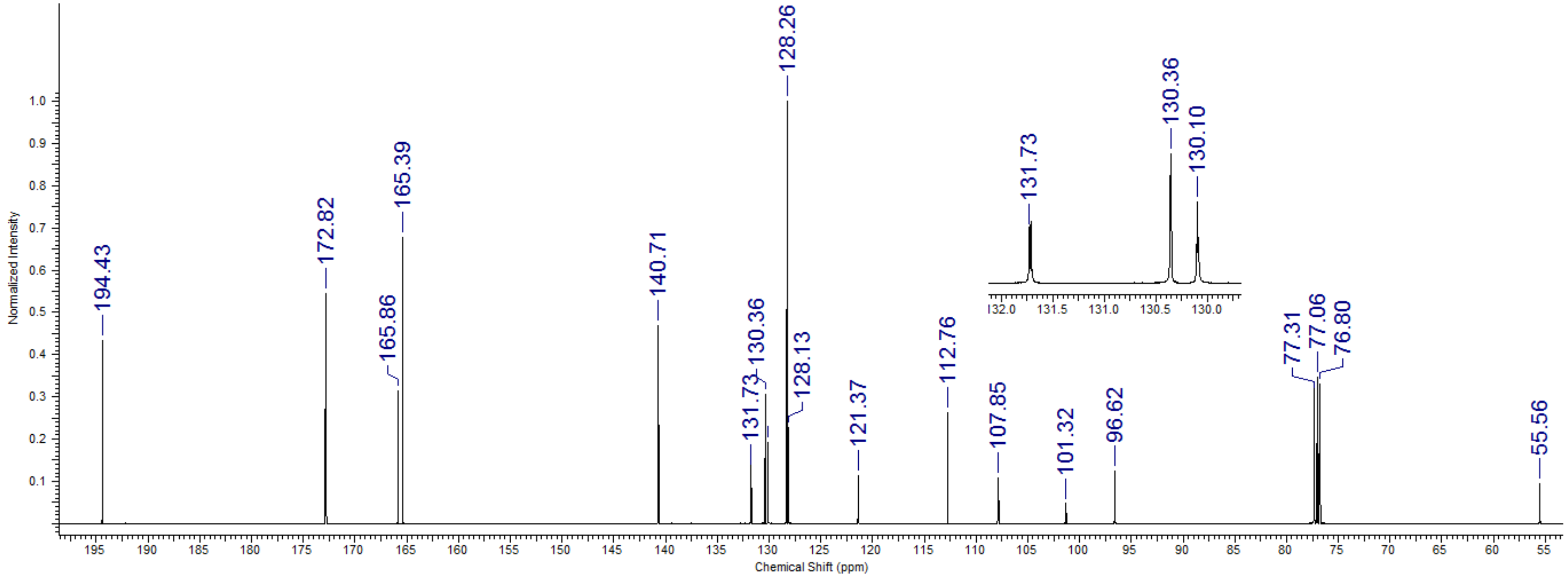

Figure S57. NMR spectra of compound **57**.

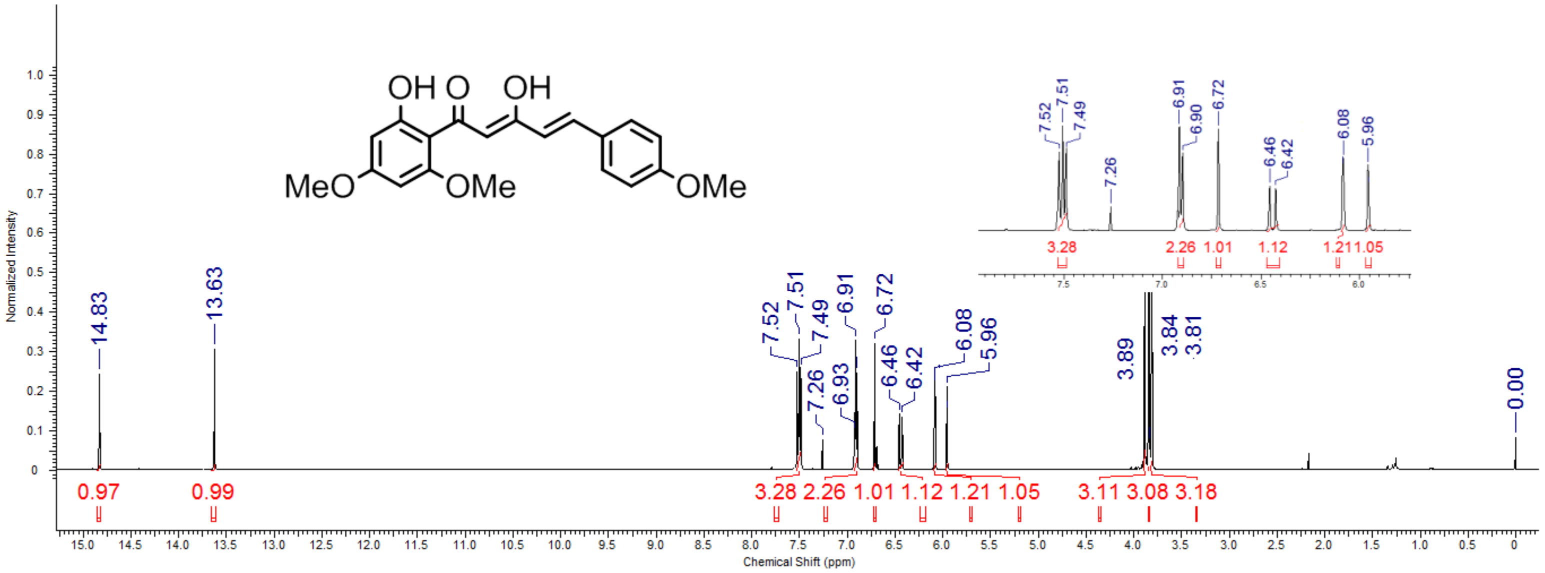

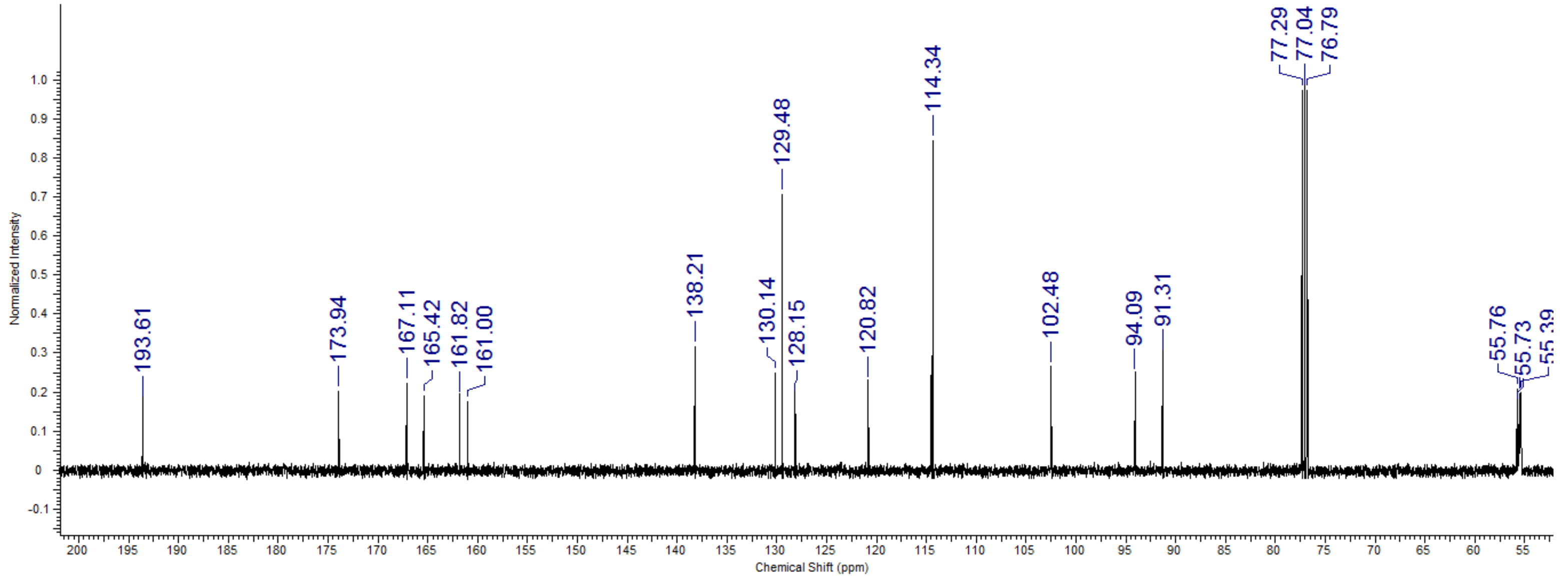

Figure S58. NMR spectra of compound **58**.

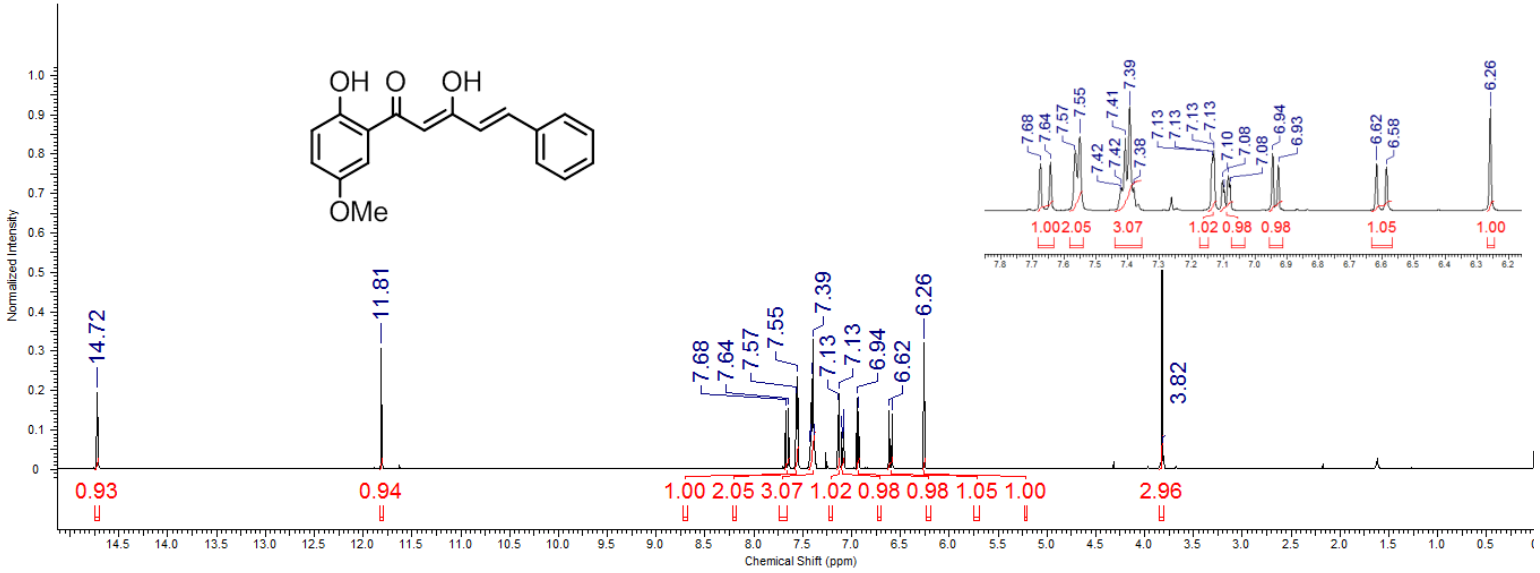

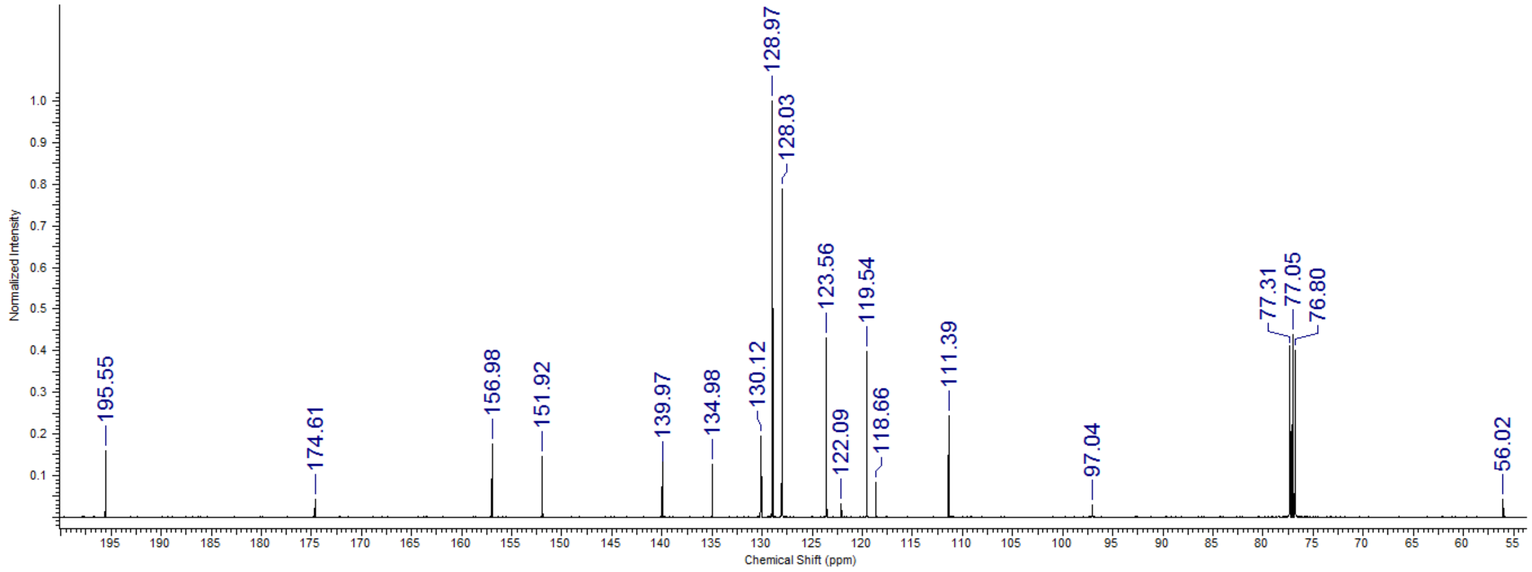

Figure S59. NMR spectra of compound **59**.

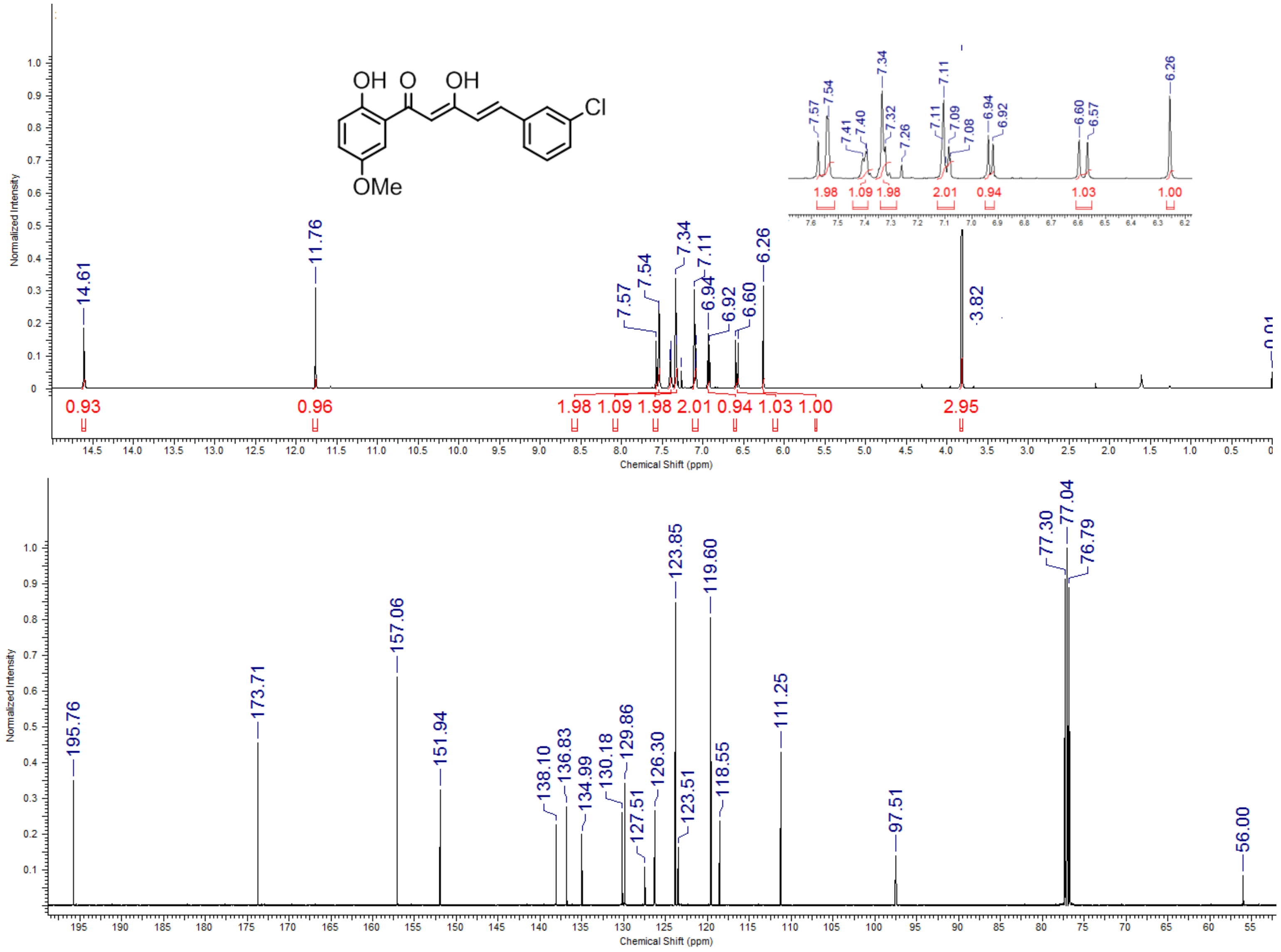

Figure S60. NMR spectra of compound **60**.

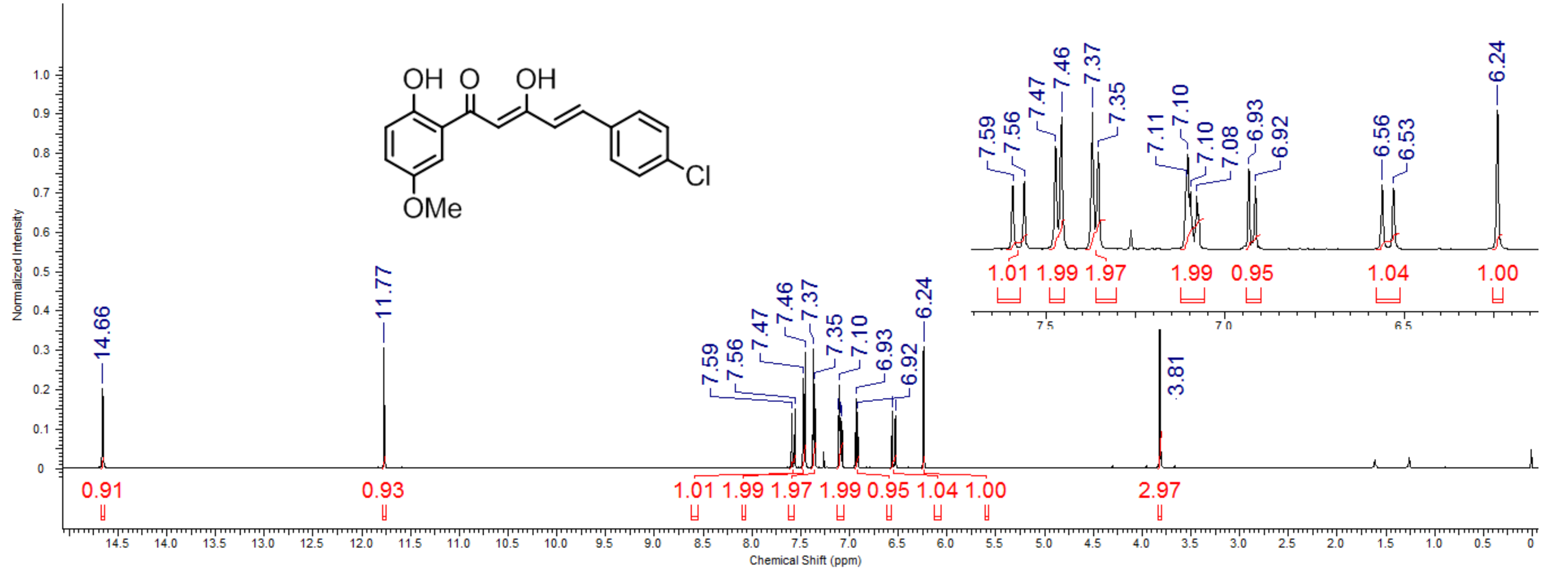

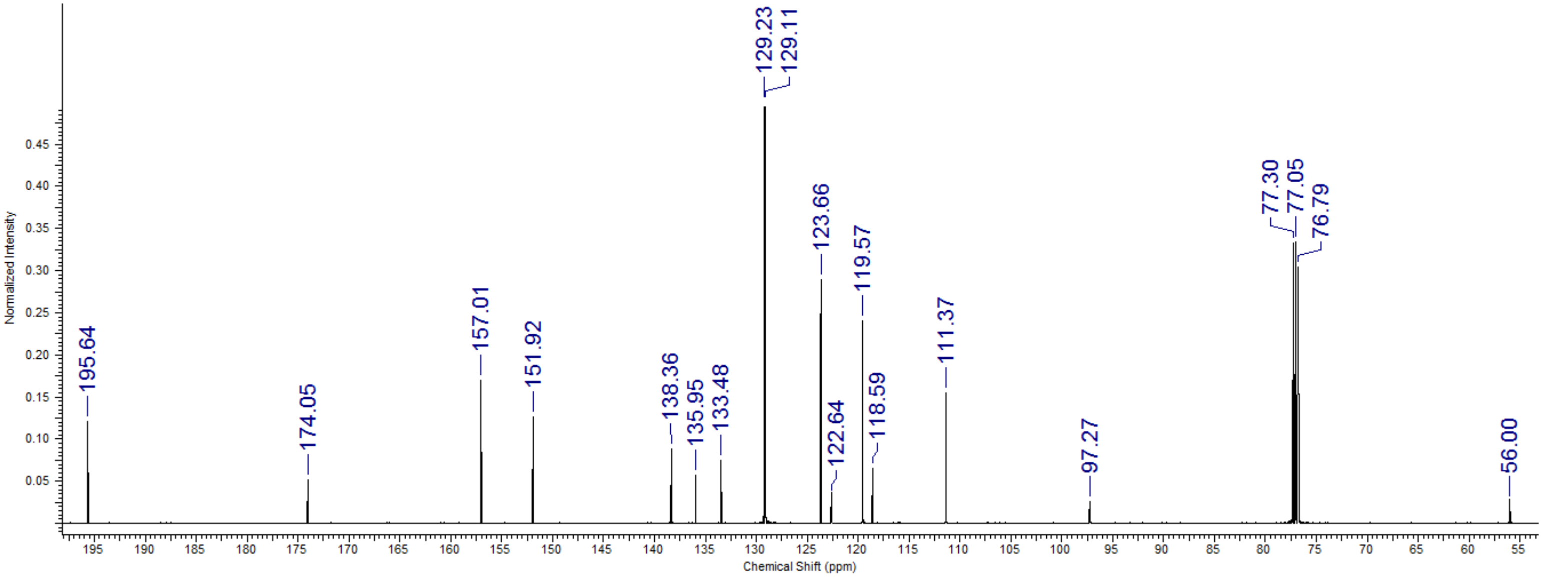

Figure S61. NMR spectra of compound **61**.

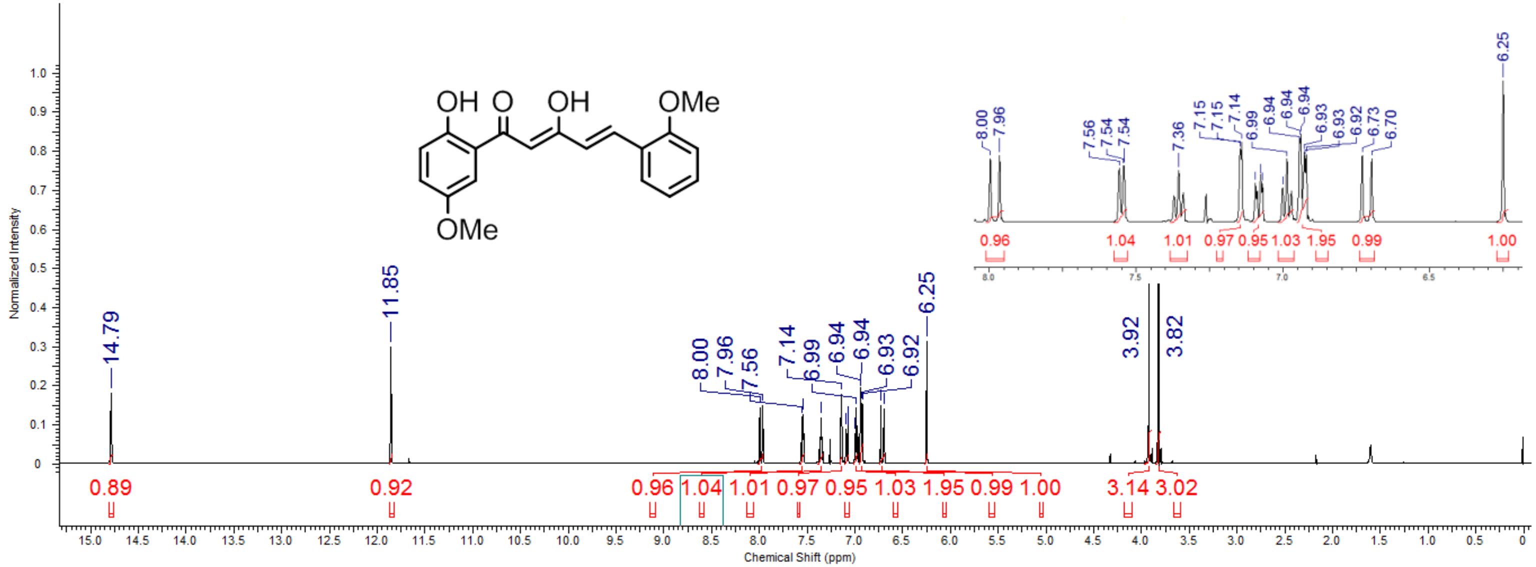

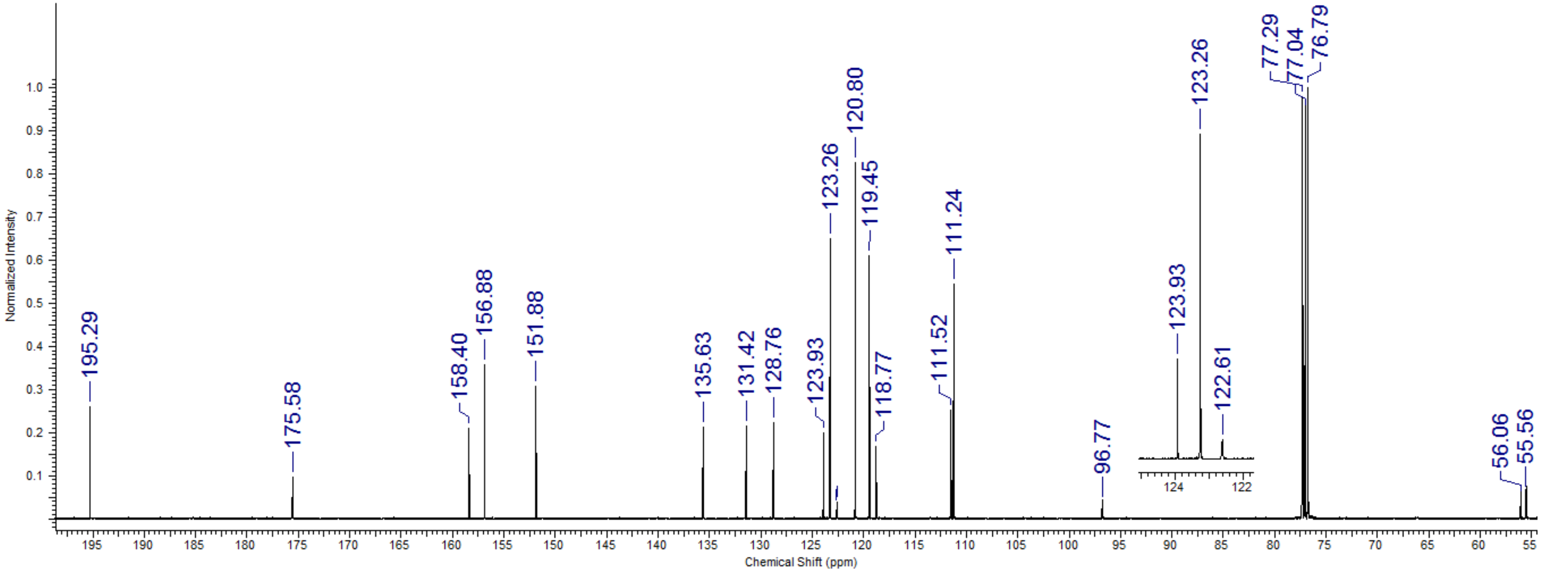

Figure S62. NMR spectra of compound **62**.

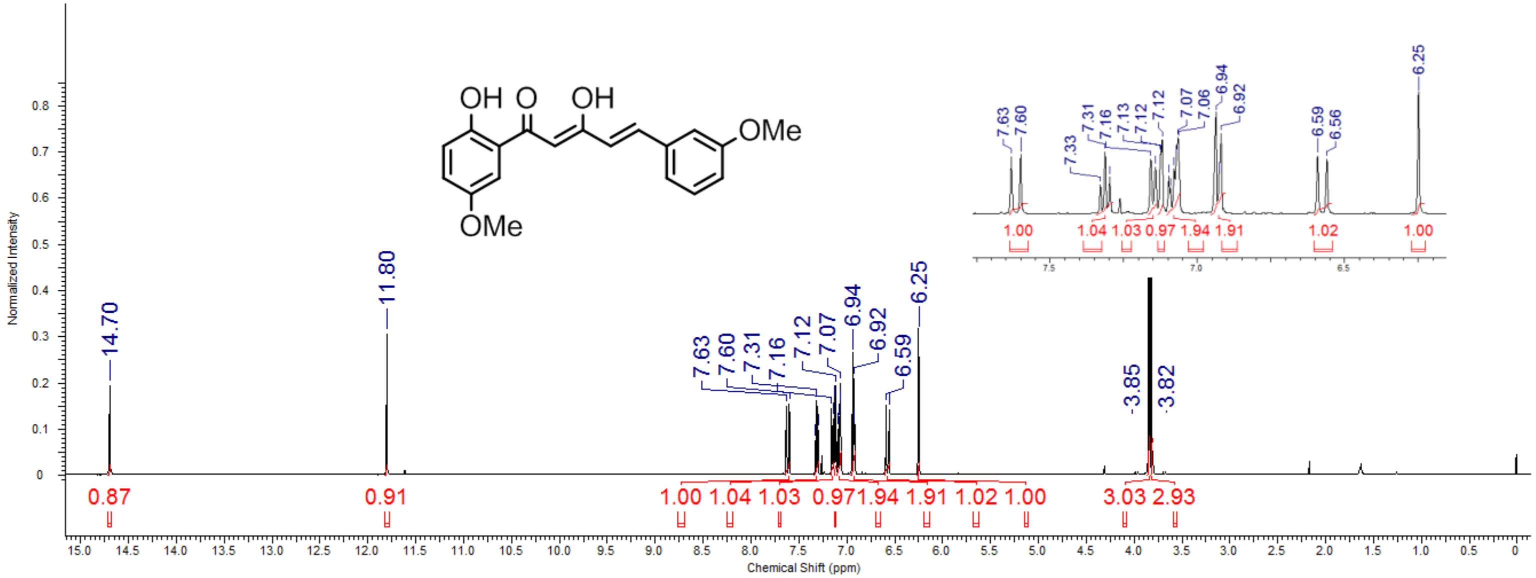

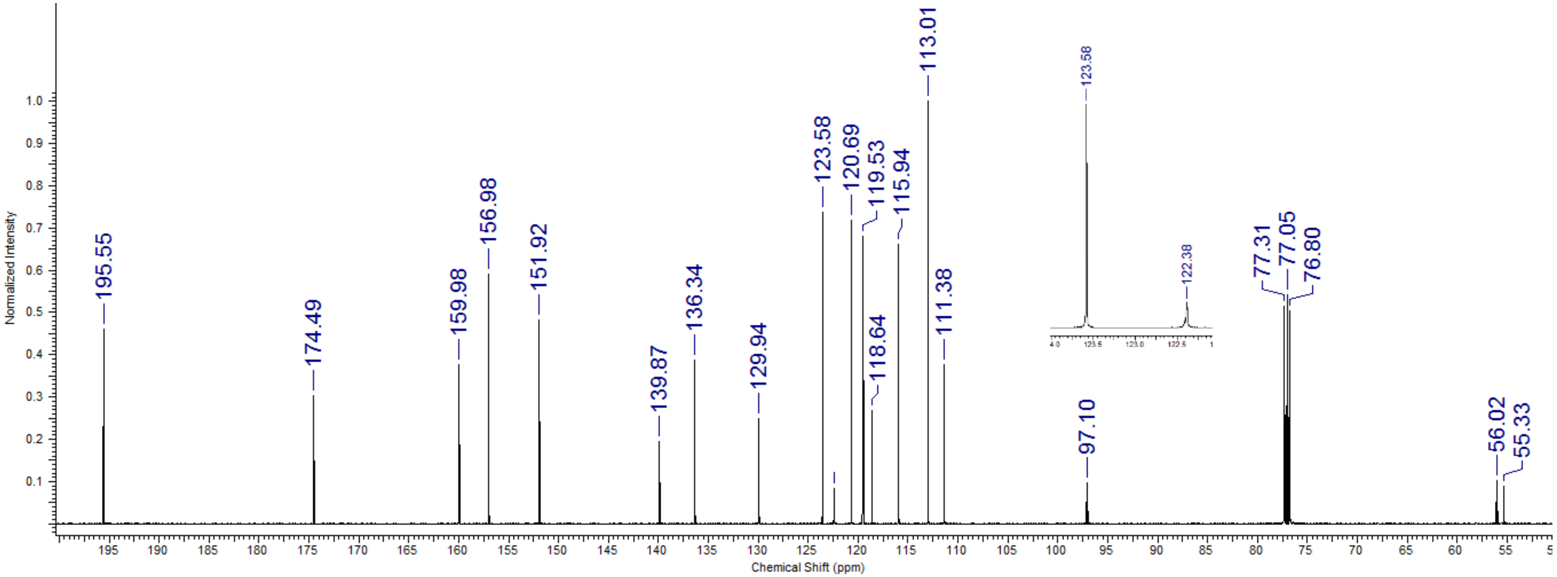

Figure S63. NMR spectra of compound **63**.

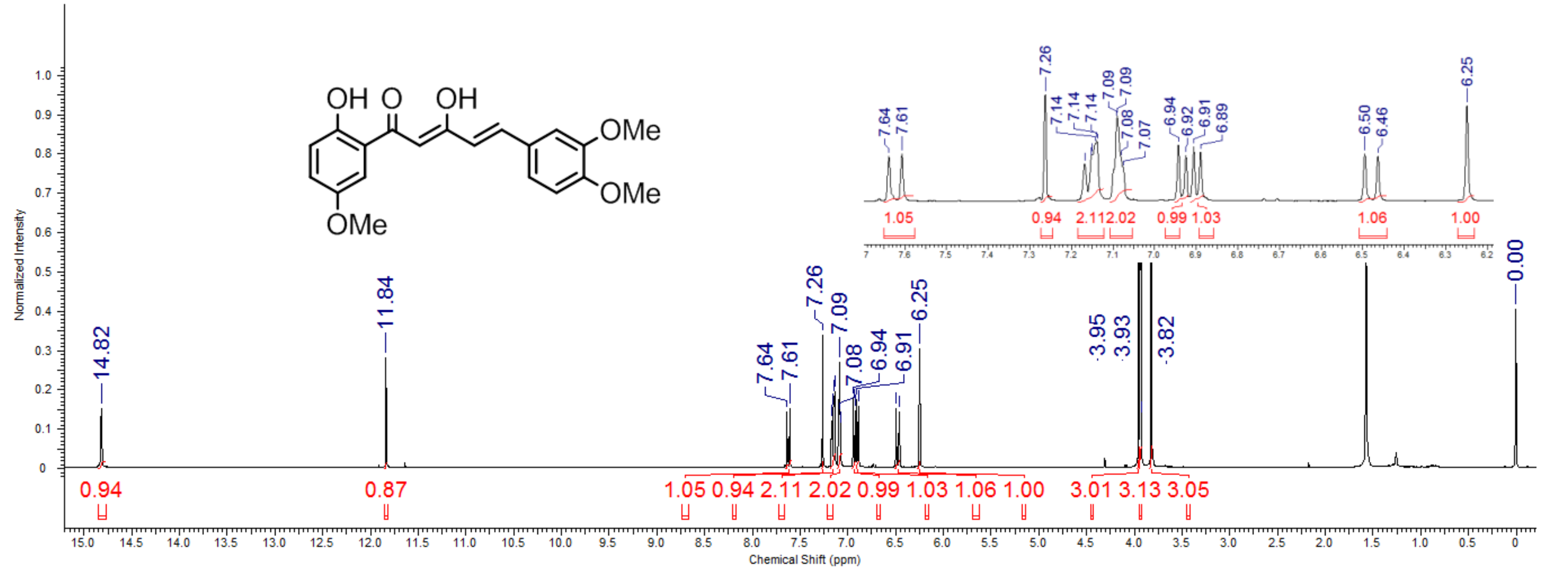

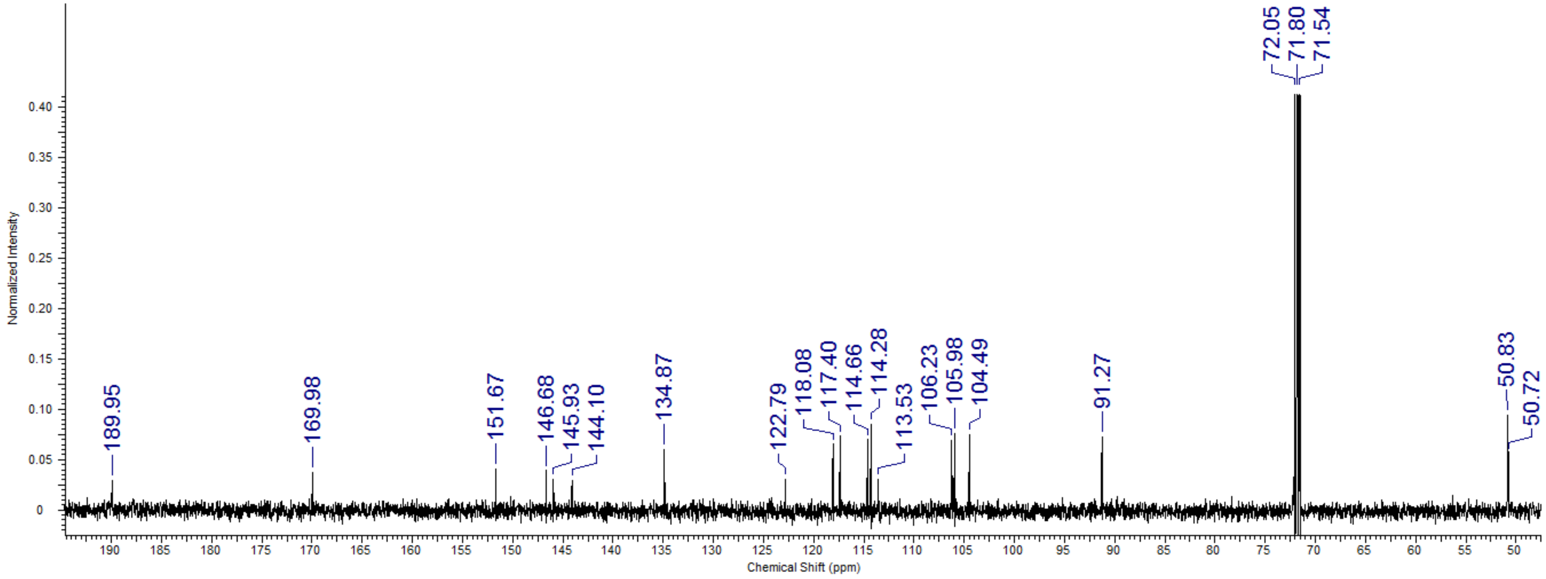

Figure S64. NMR spectra of compound **64**.

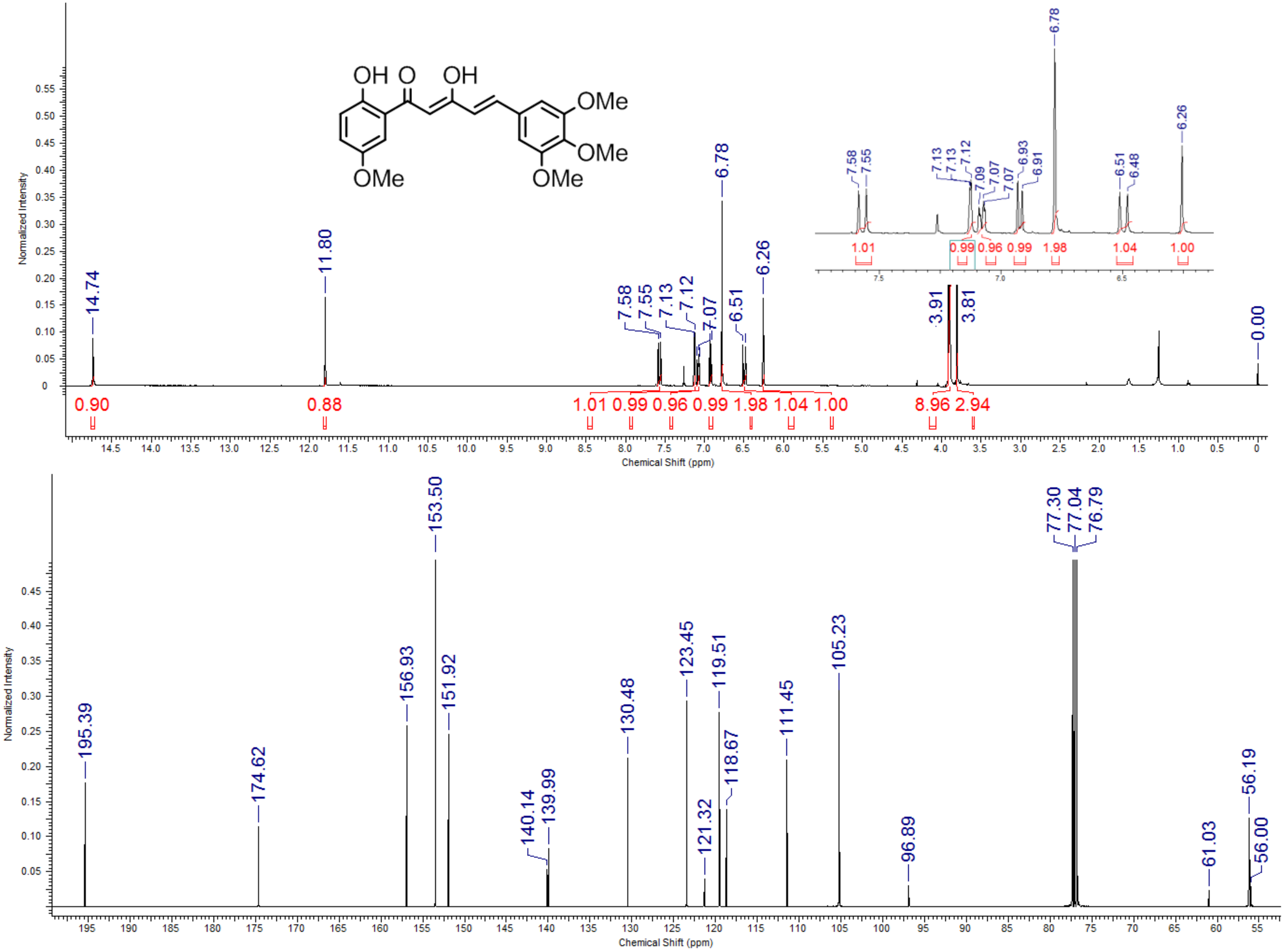

Figure S65. NMR spectra of compound **65**.

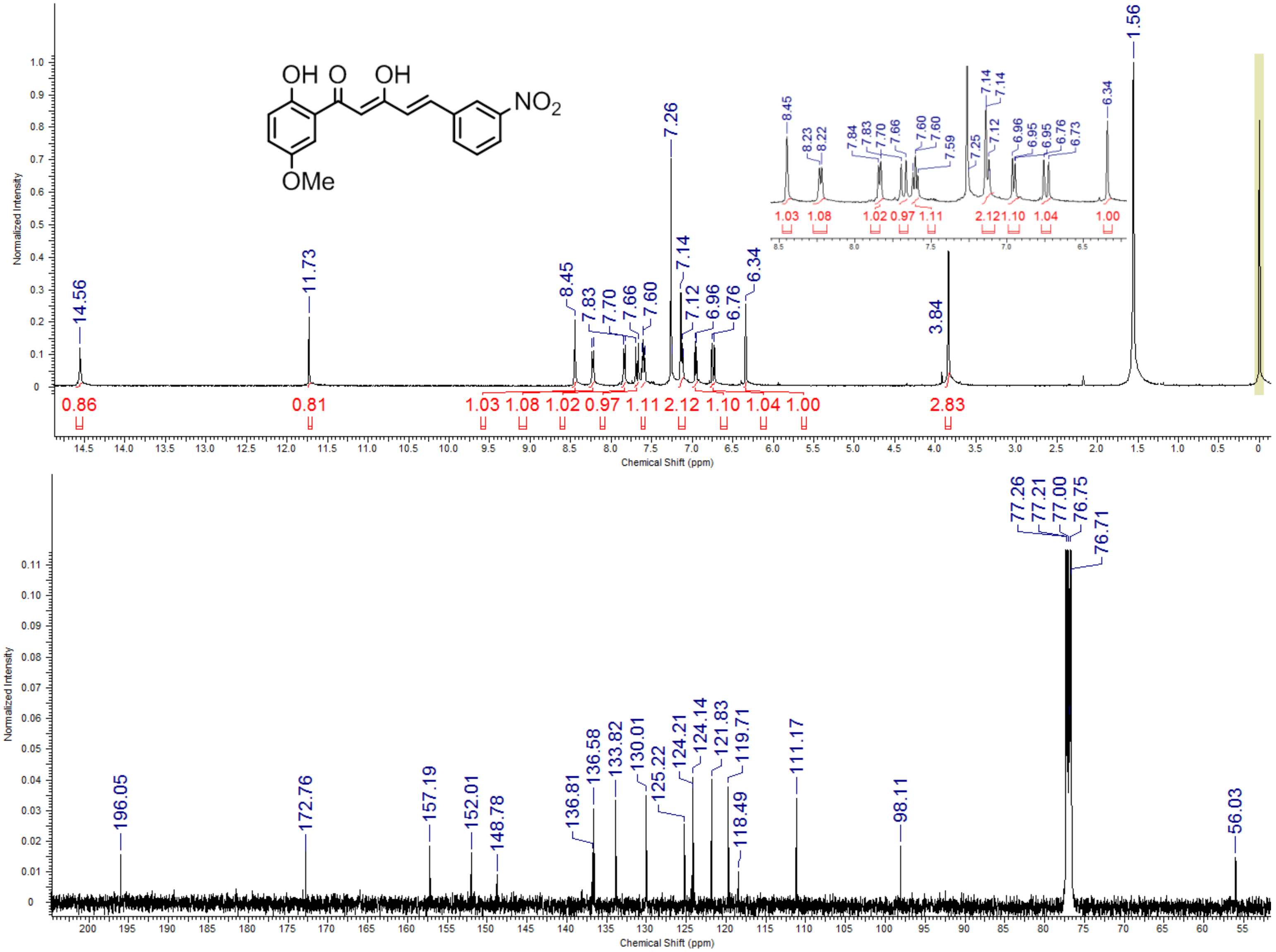

Figure S66. NMR spectra of compound **66**.

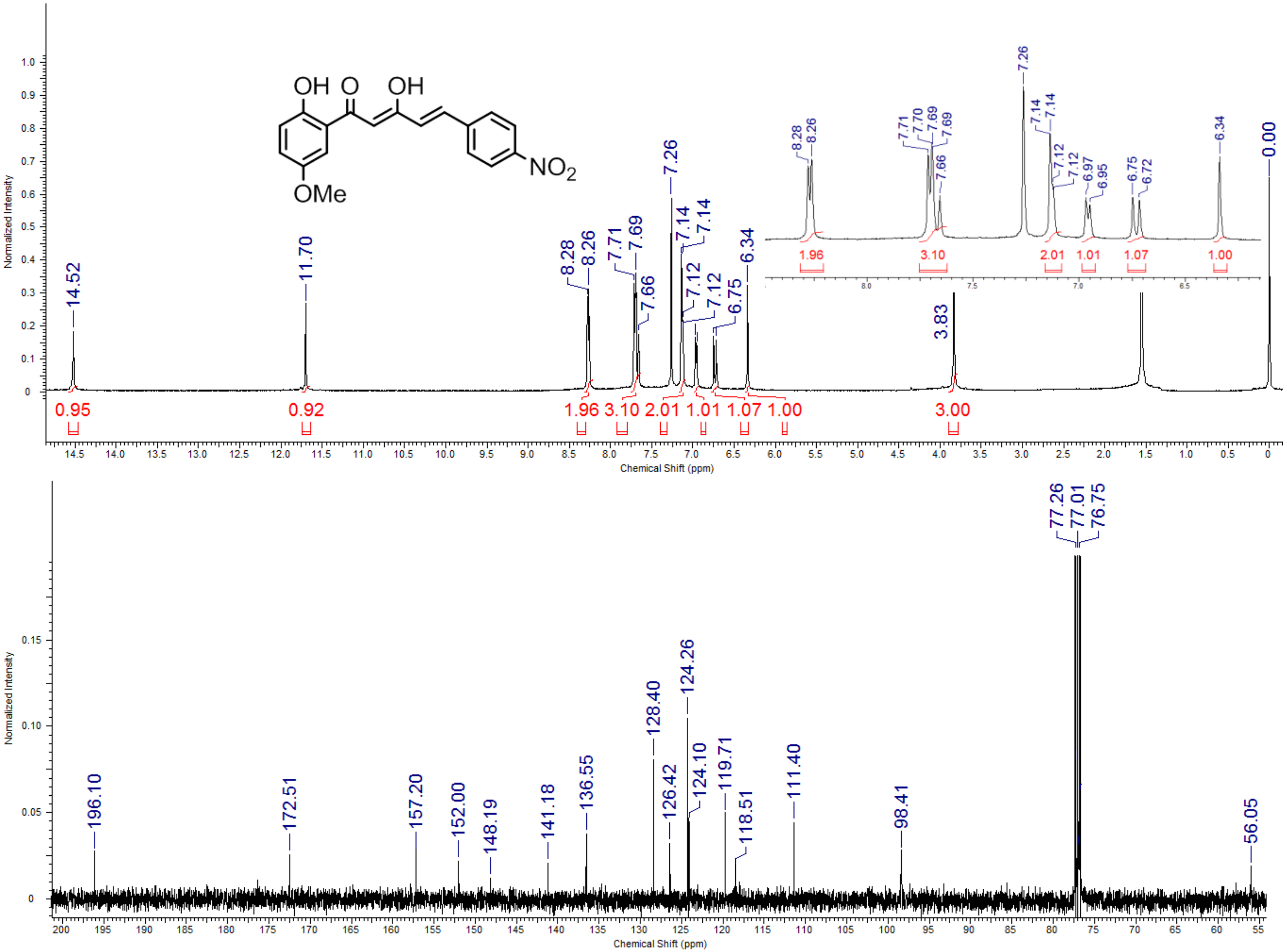

Figure S67. NMR spectra of compound **67**.

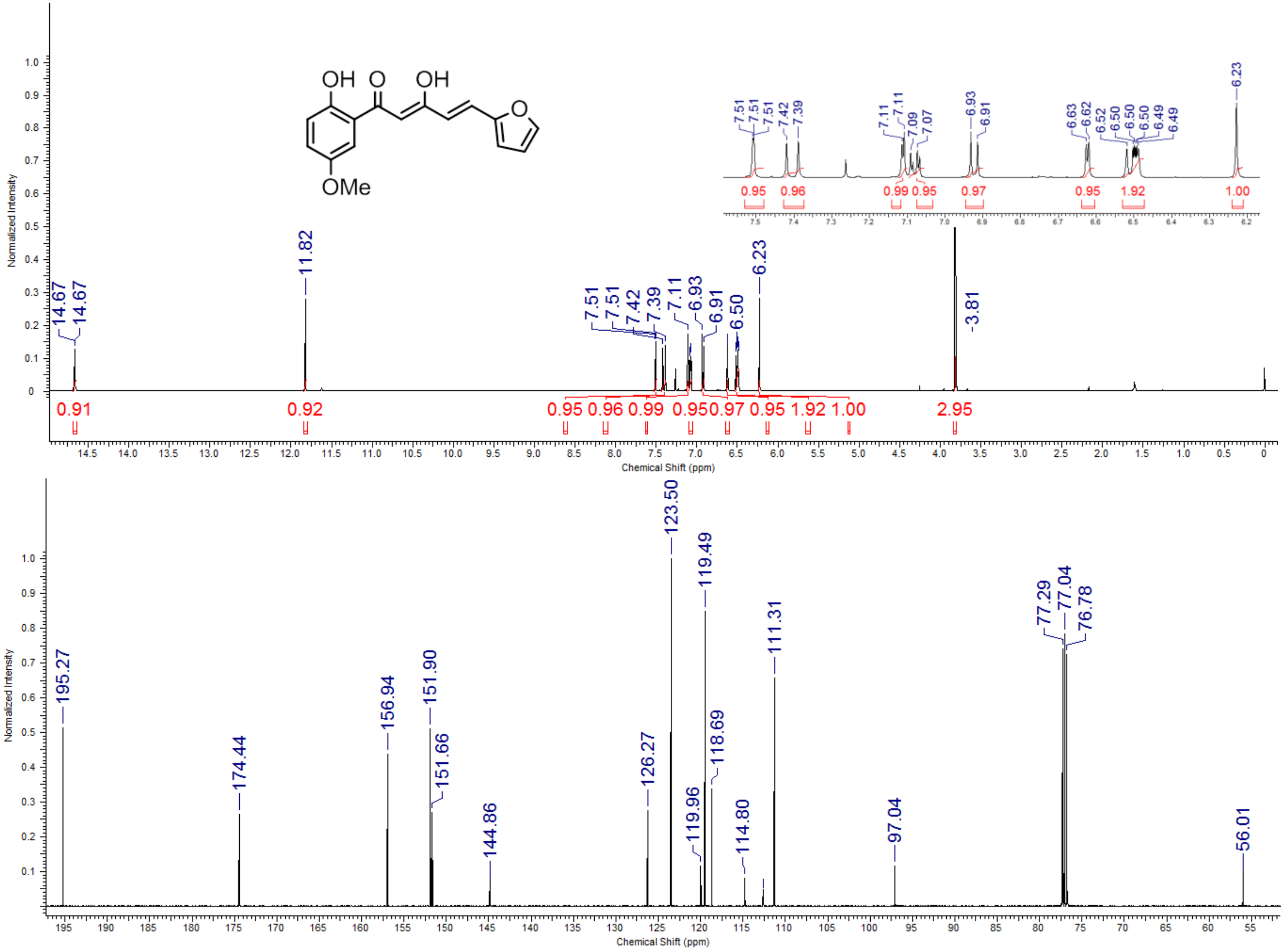

Figure S68. NMR spectra of compound **68**.

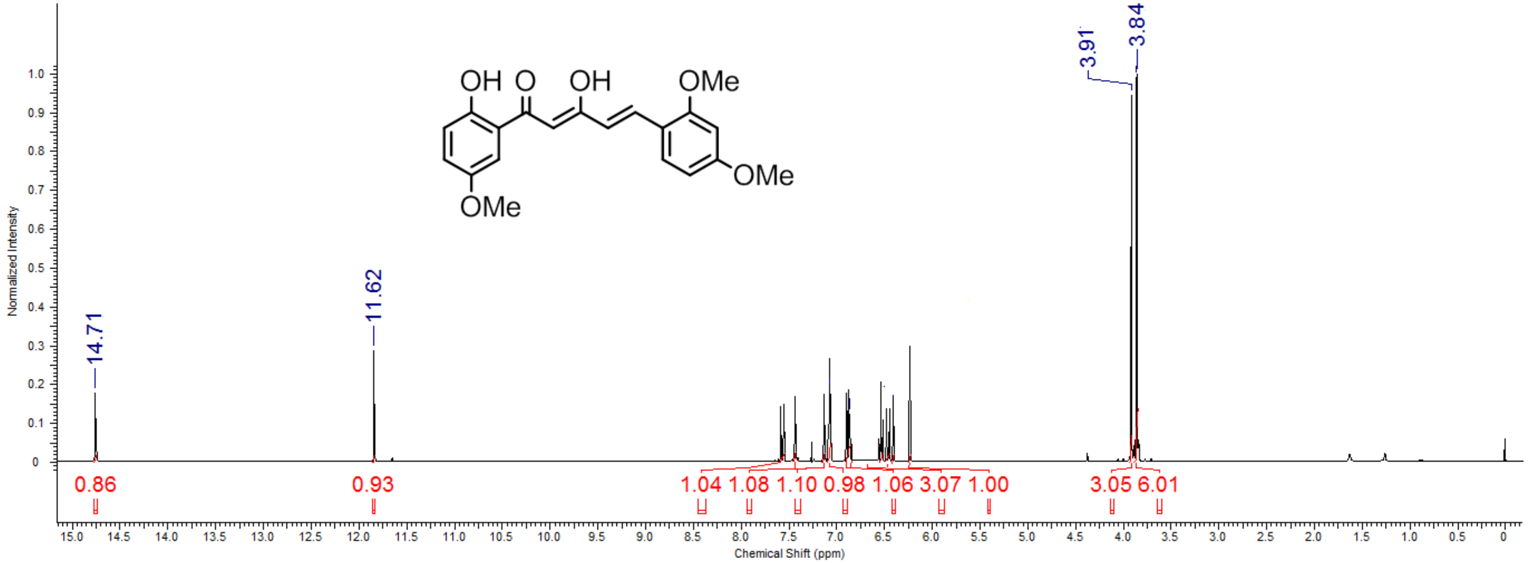

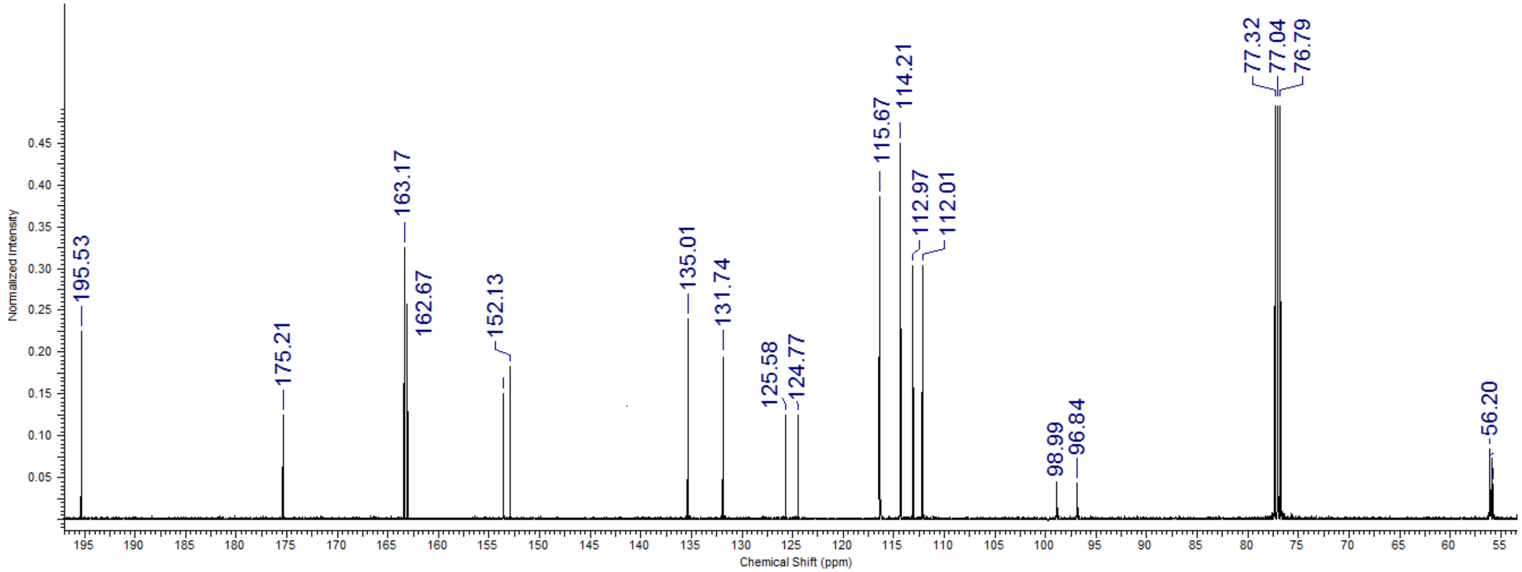

Figure S69. NMR spectra of compound **69**.

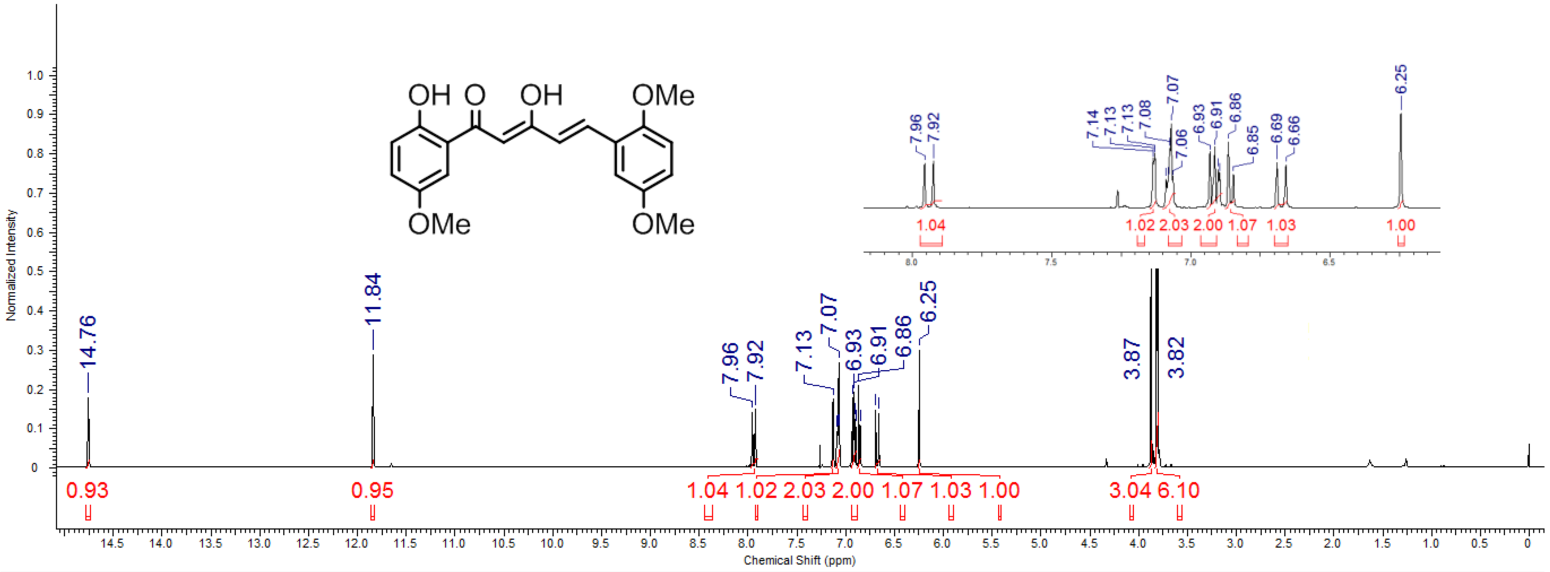

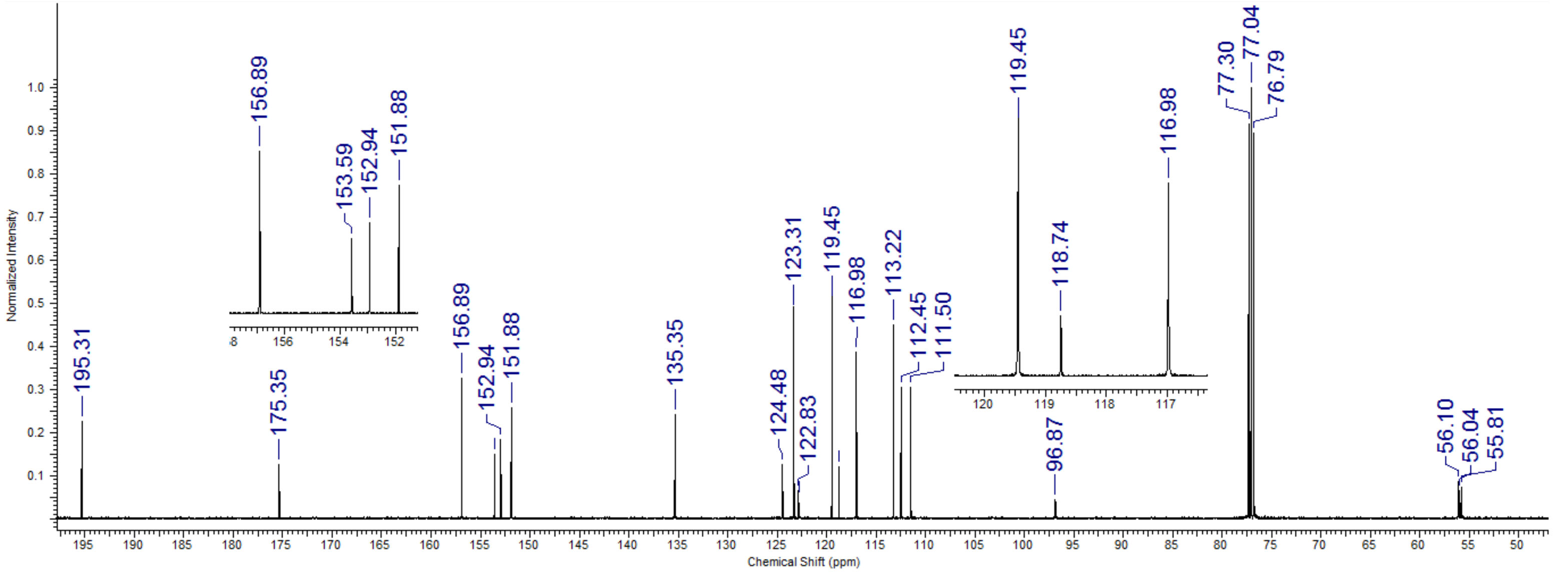

Figure S70. NMR spectra of compound **70**.

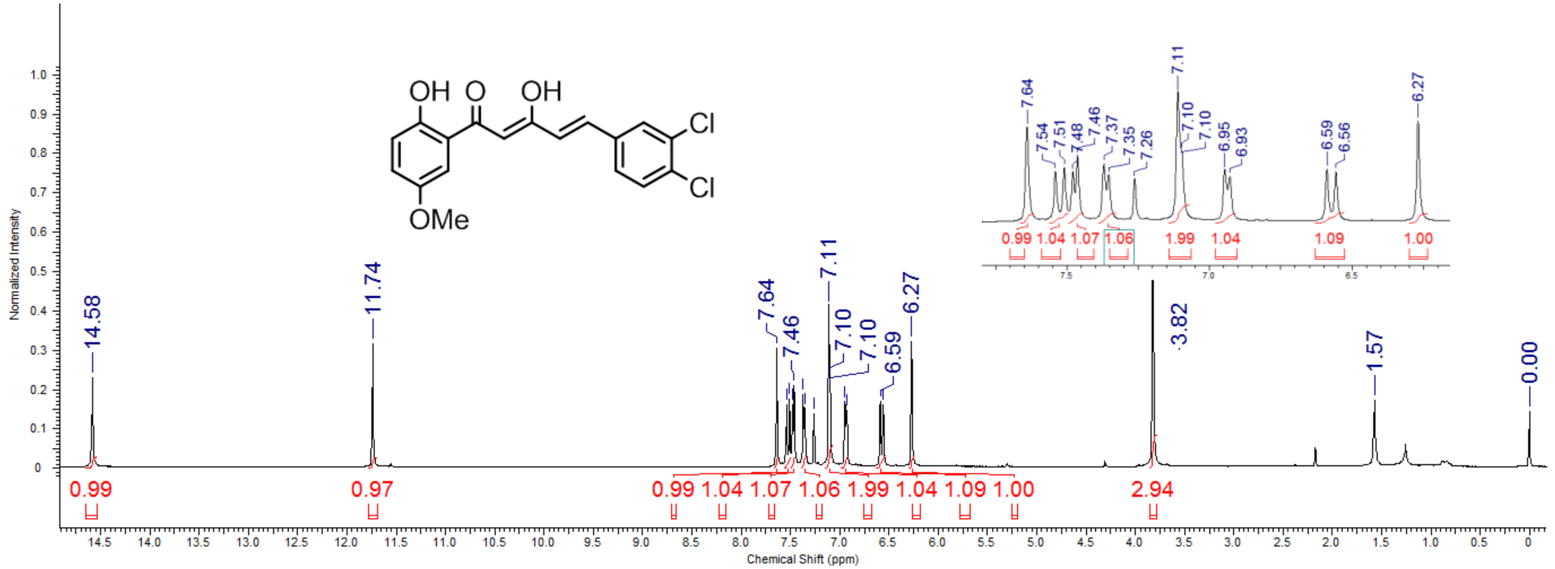

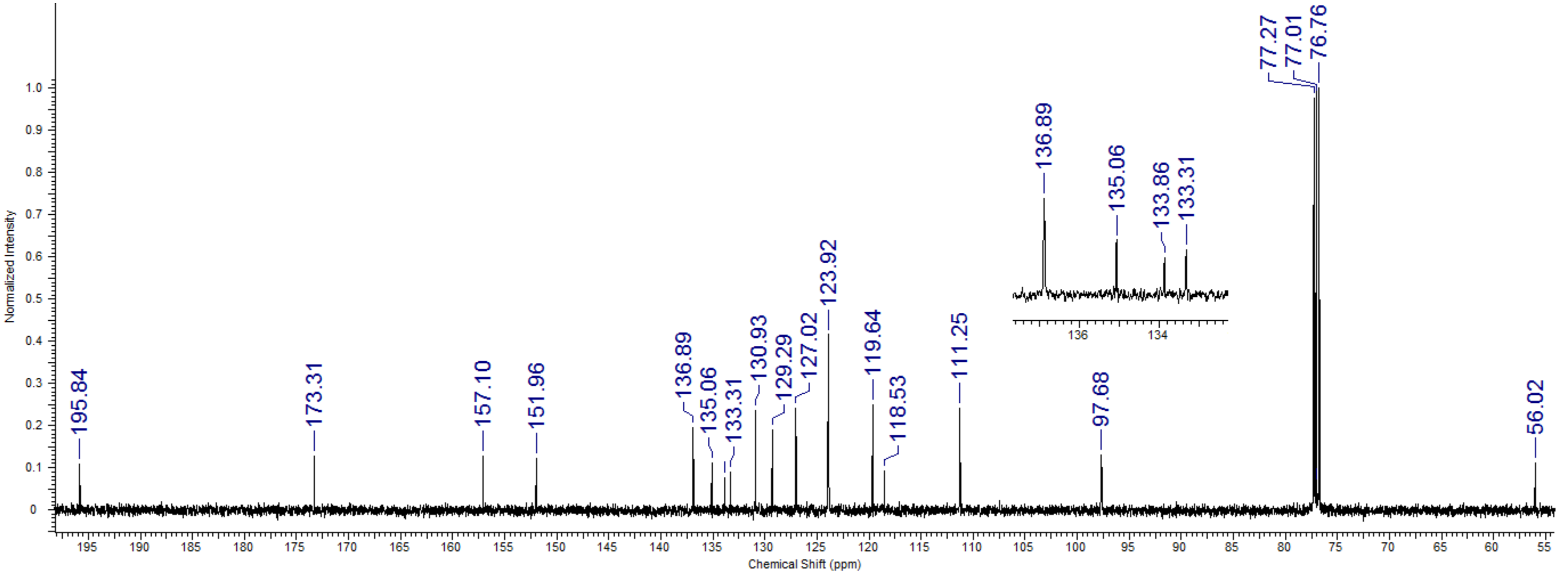

Figure S71. NMR spectra of compound **71**.

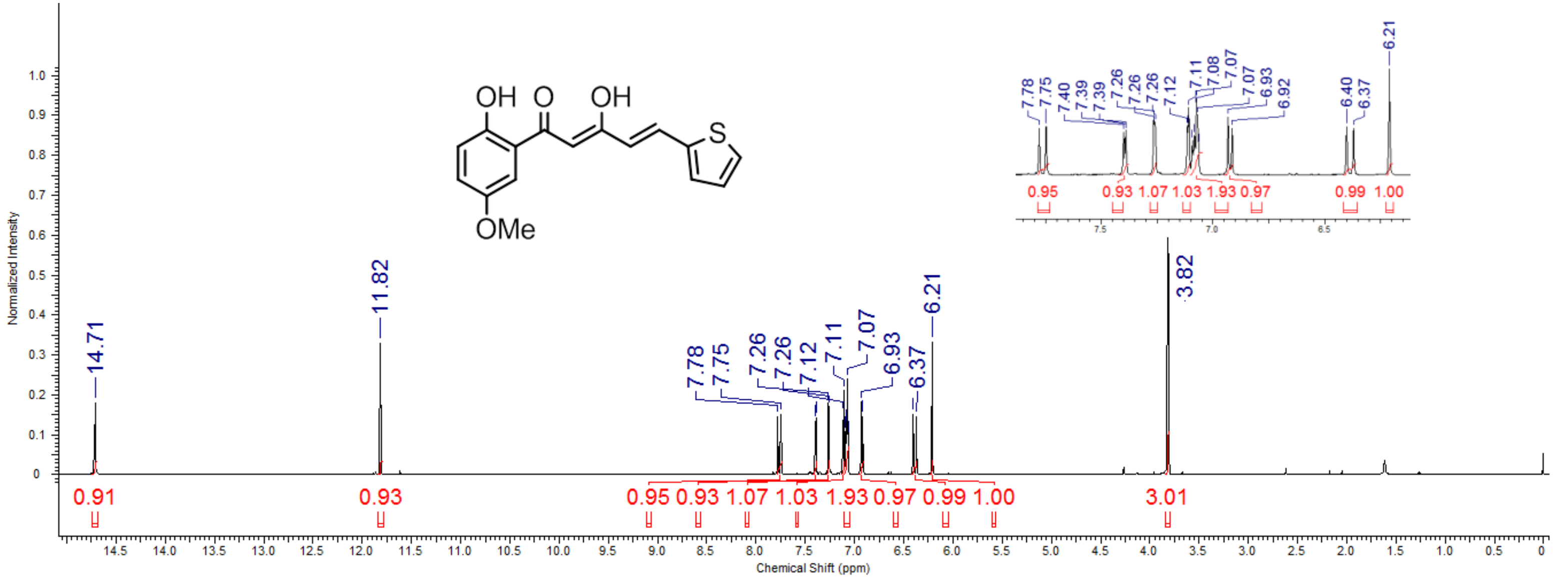

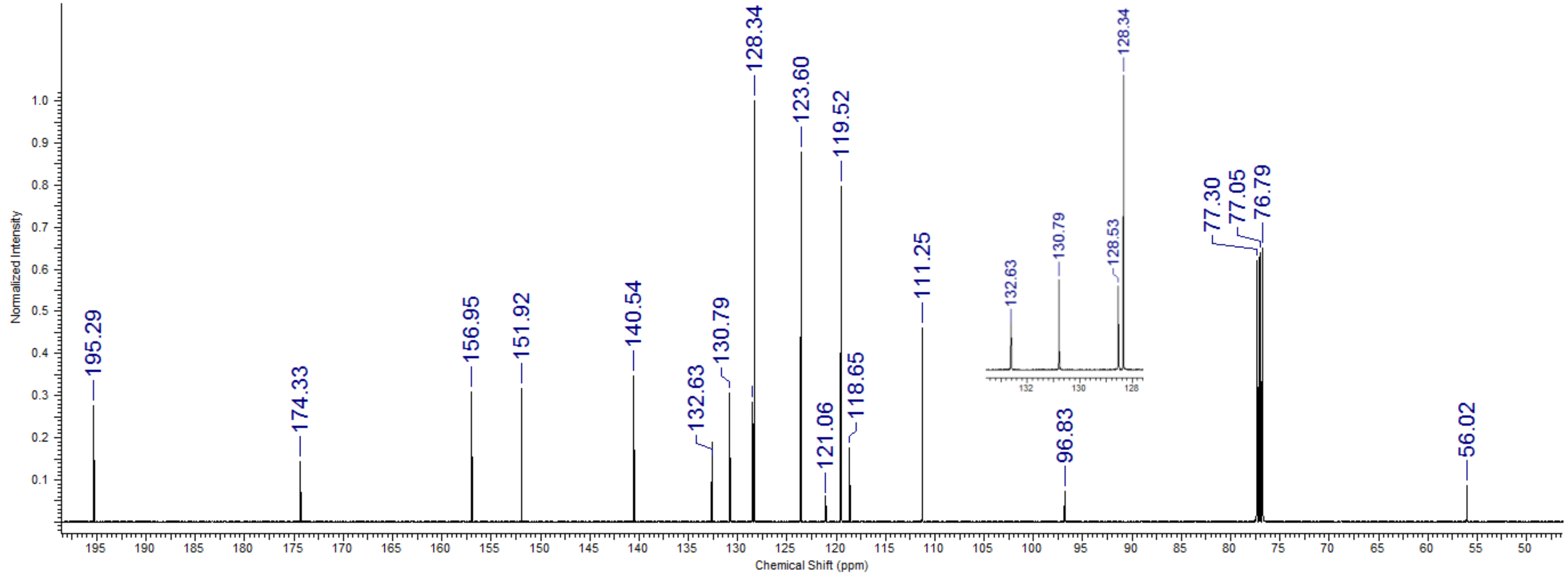

Figure S72. NMR spectra of compound **72**.

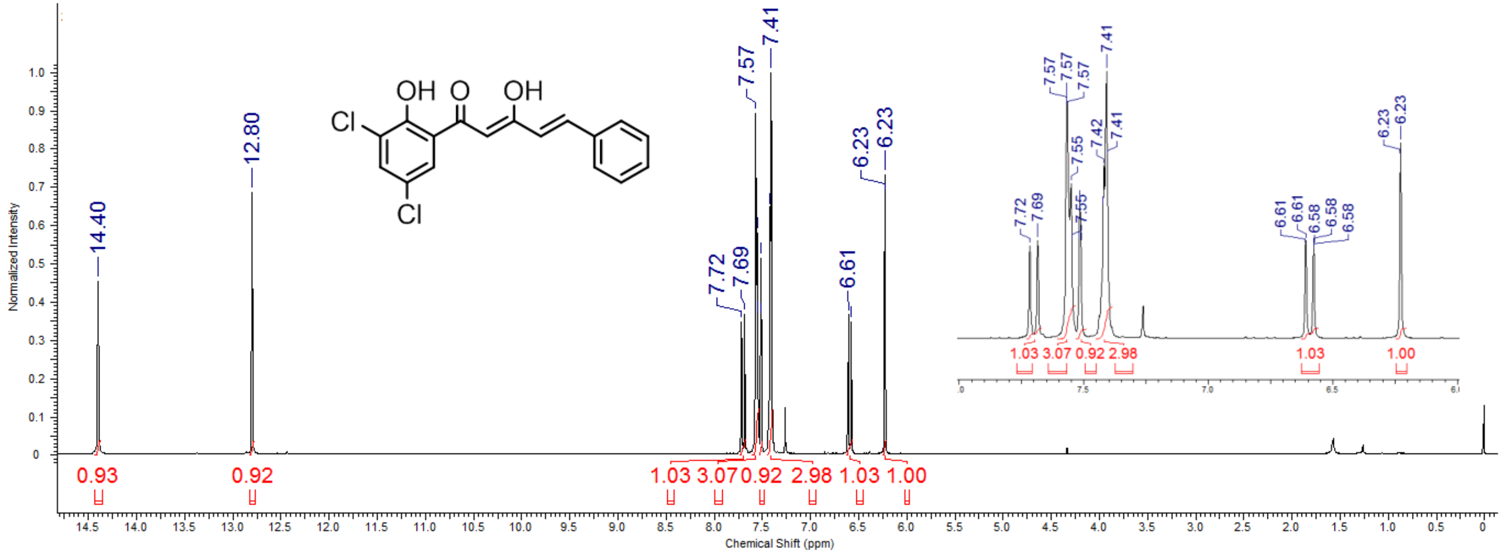

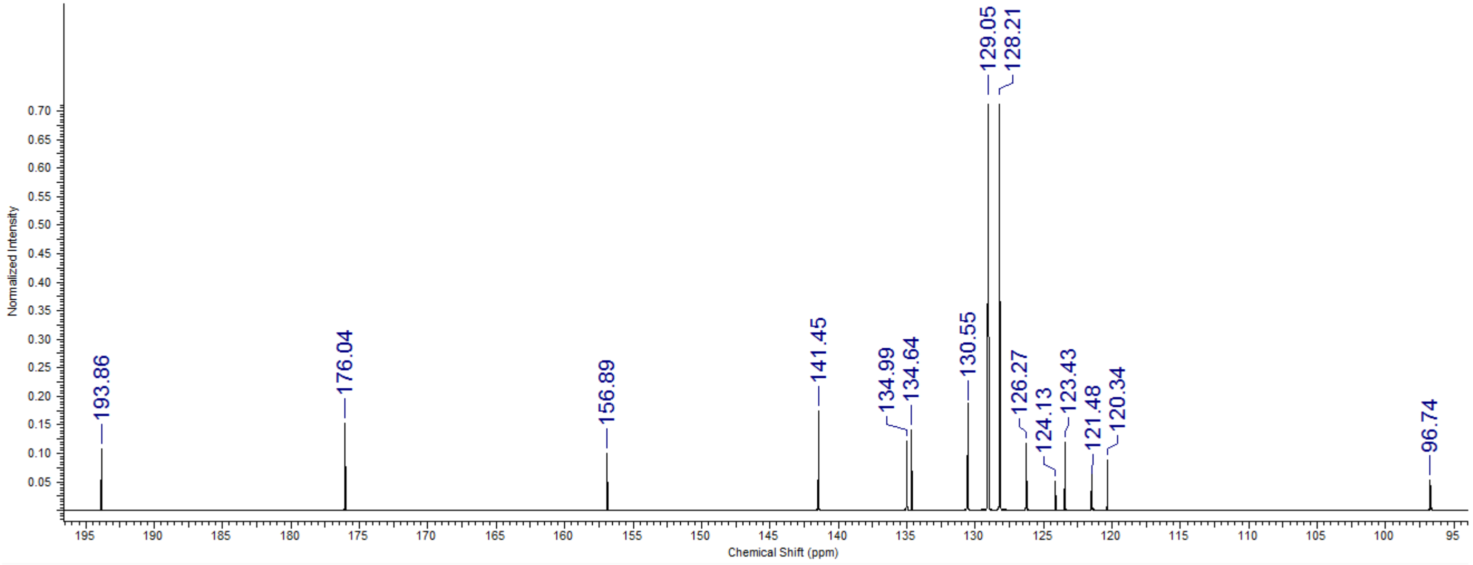

Figure S73. NMR spectra of compound **73**.

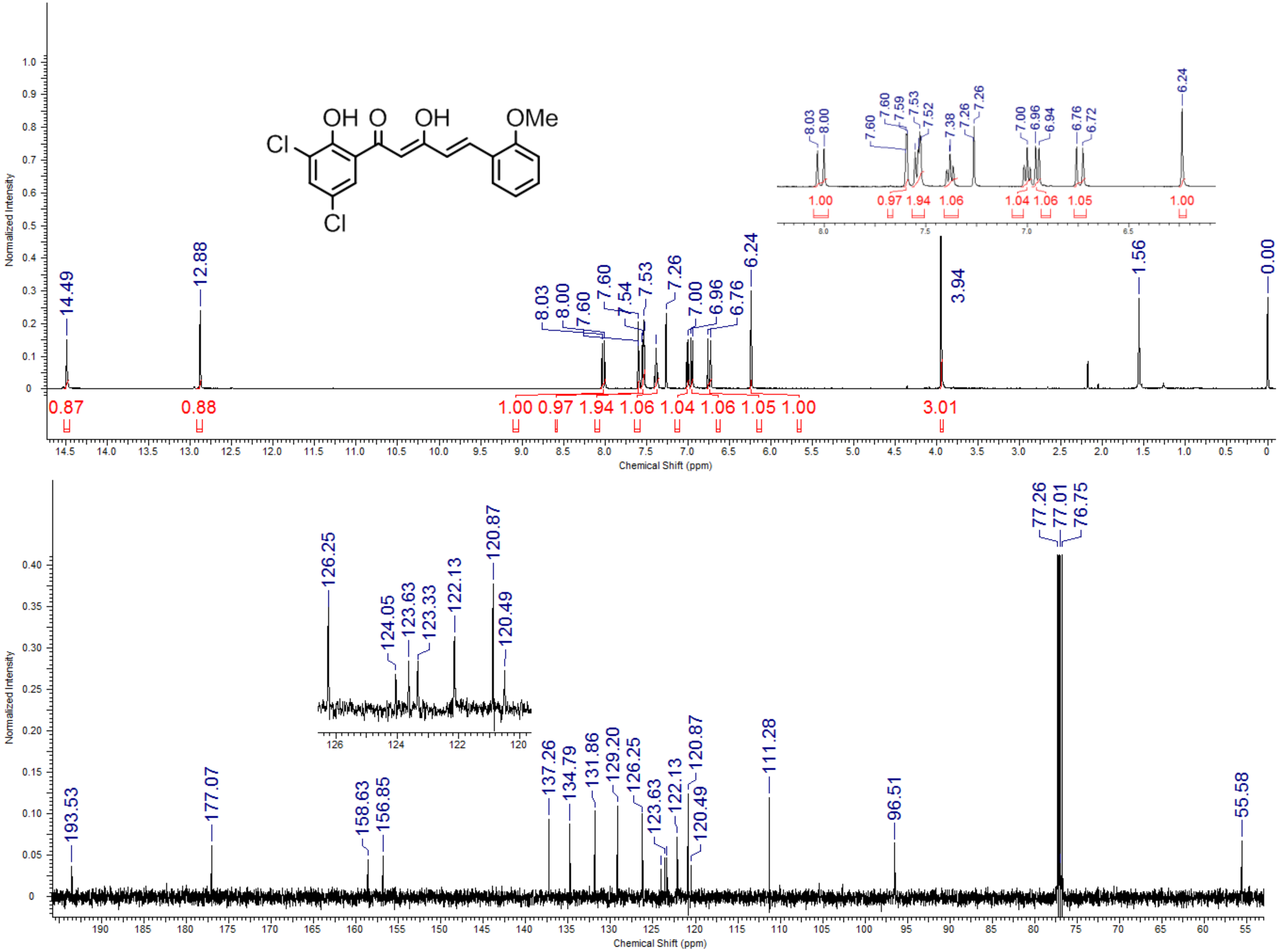

Figure S74. NMR spectra of compound **74**.

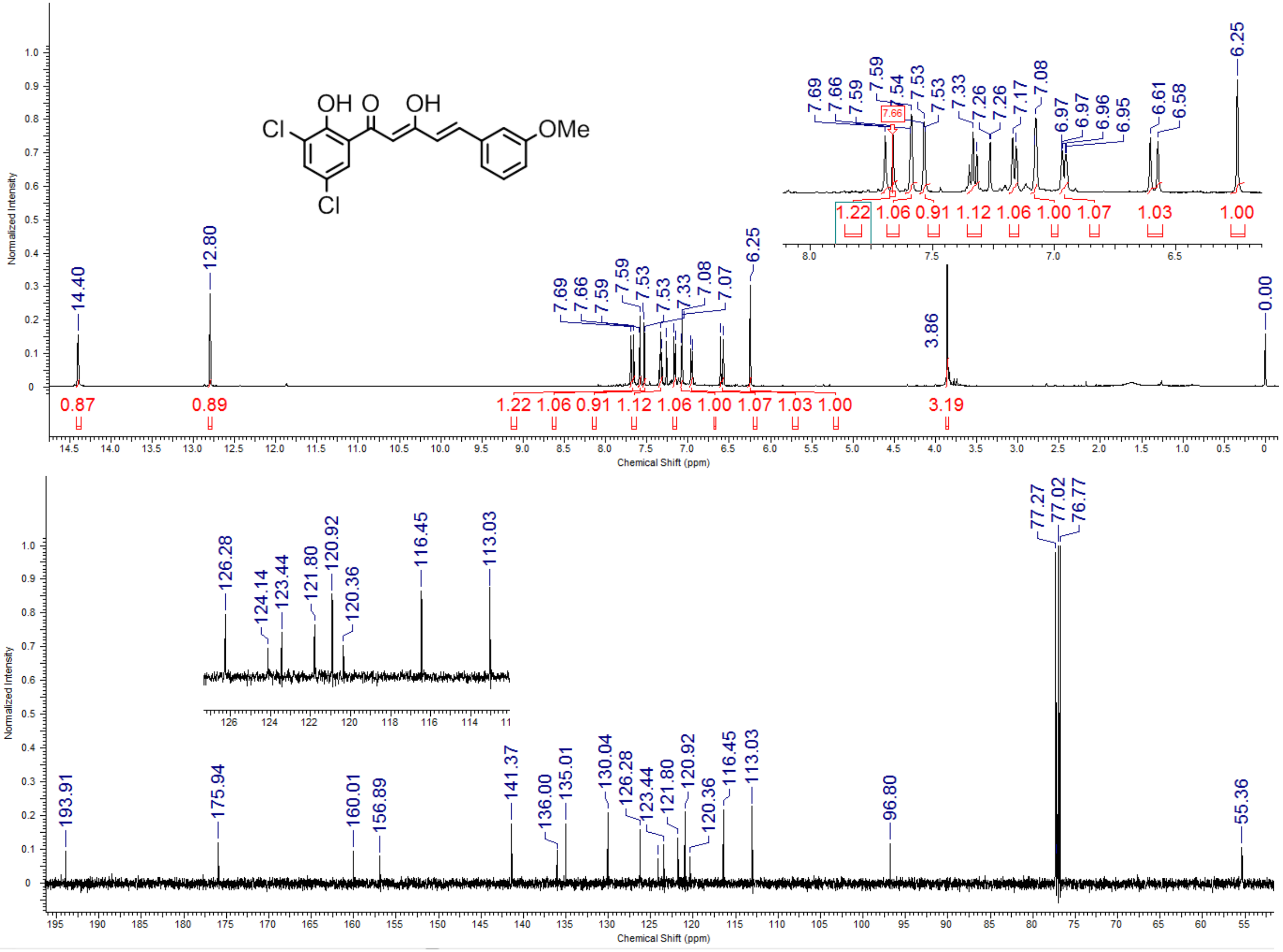

Figure S75. NMR spectra of compound **75**.

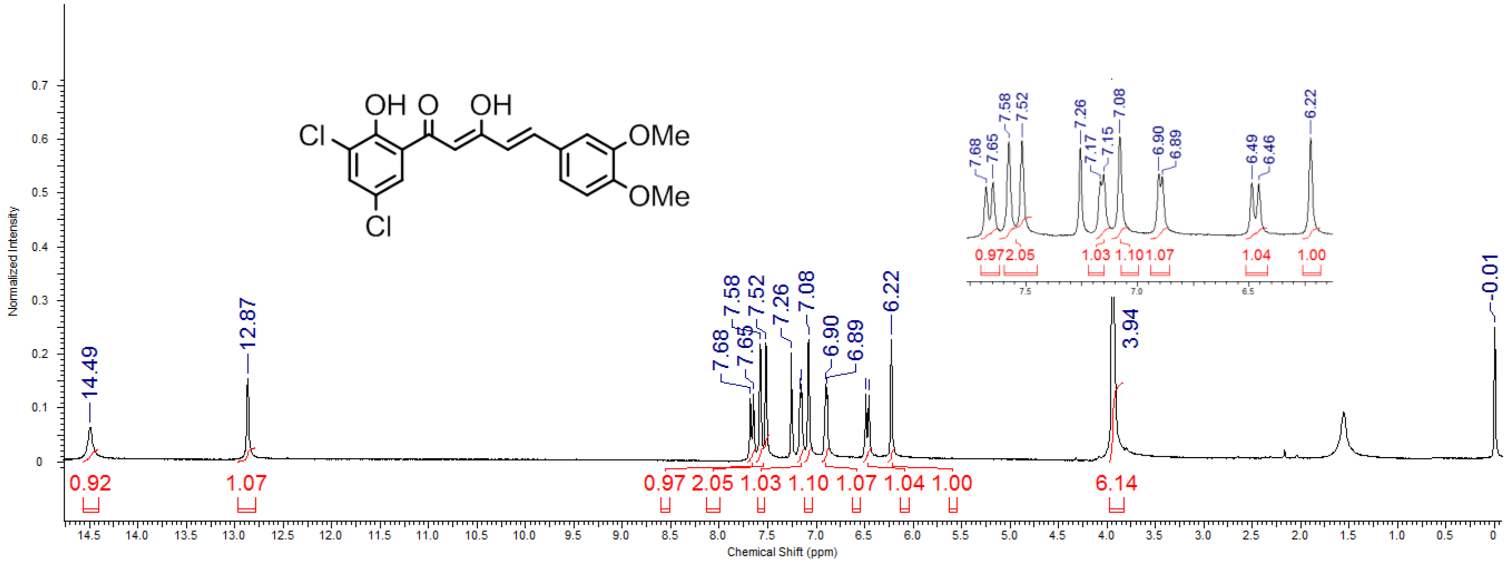

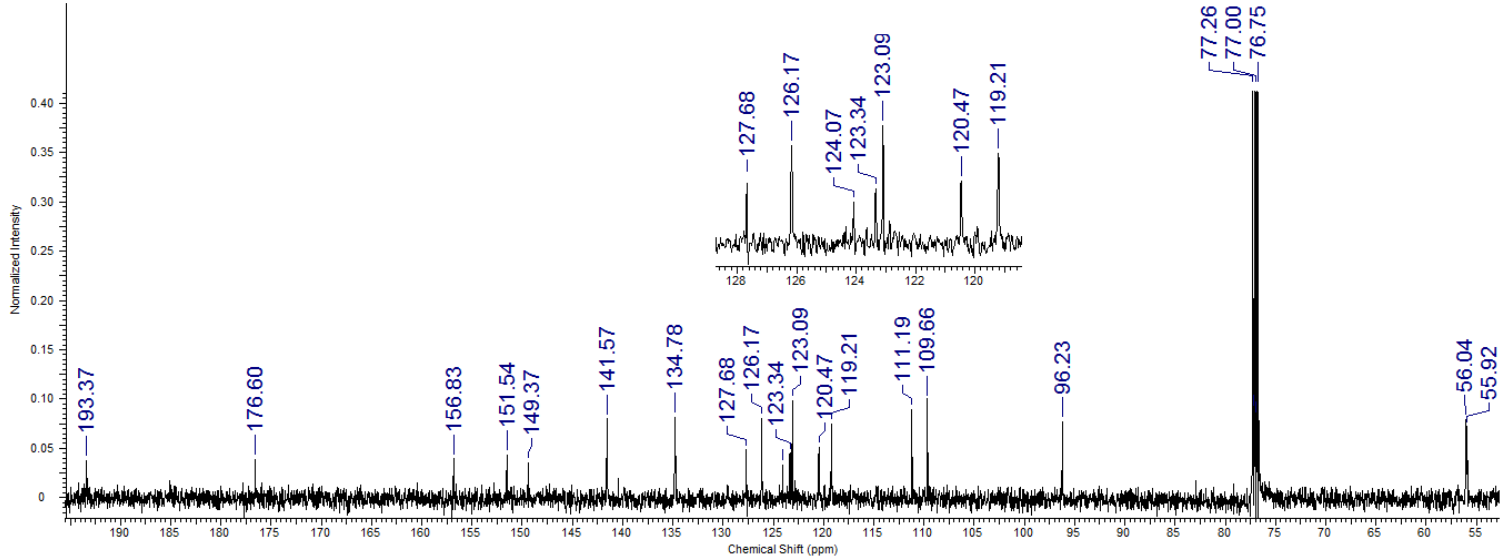

Figure S76. NMR spectra of compound **76**.

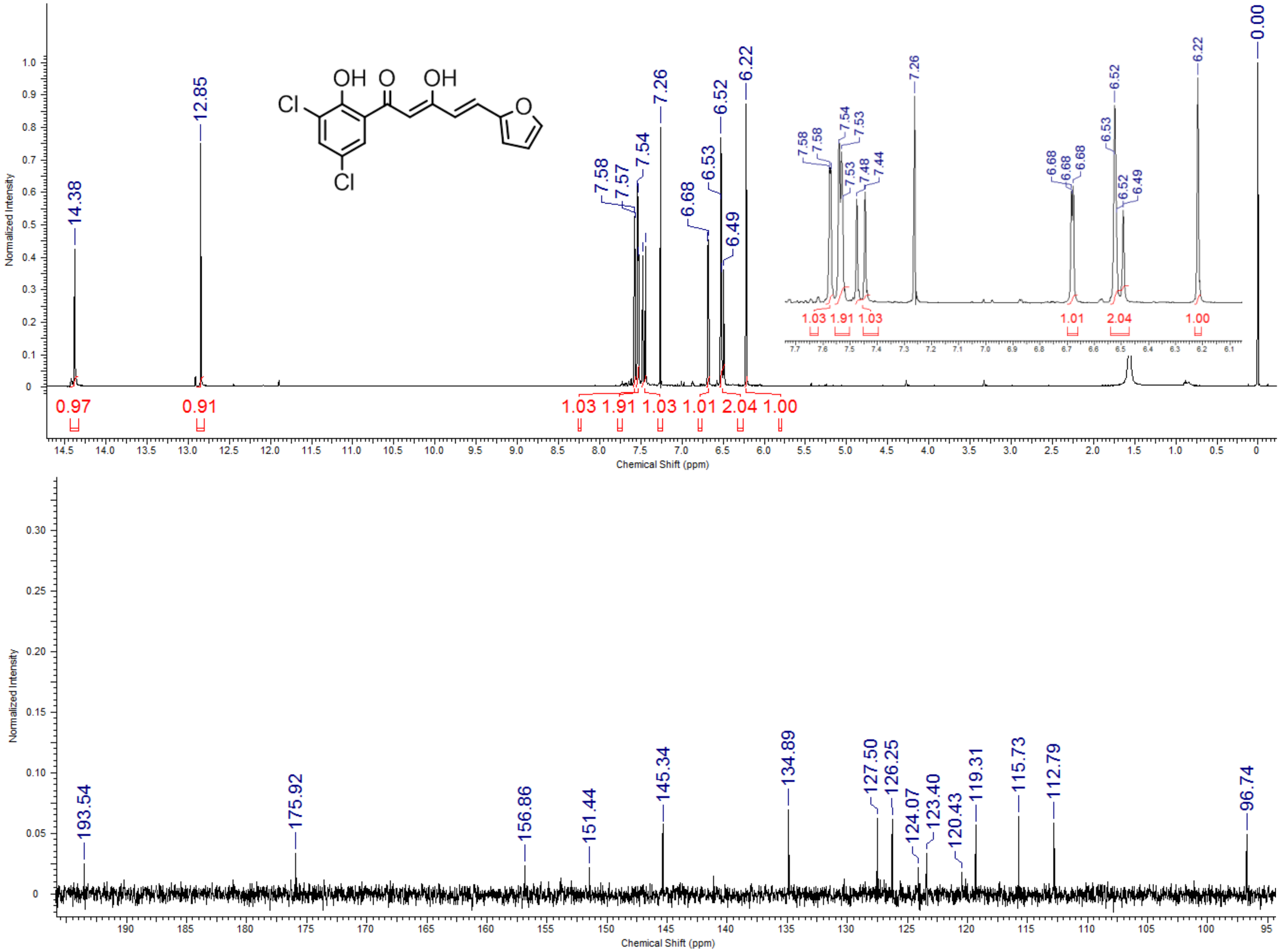

Figure S77. NMR spectra of compound **77**.

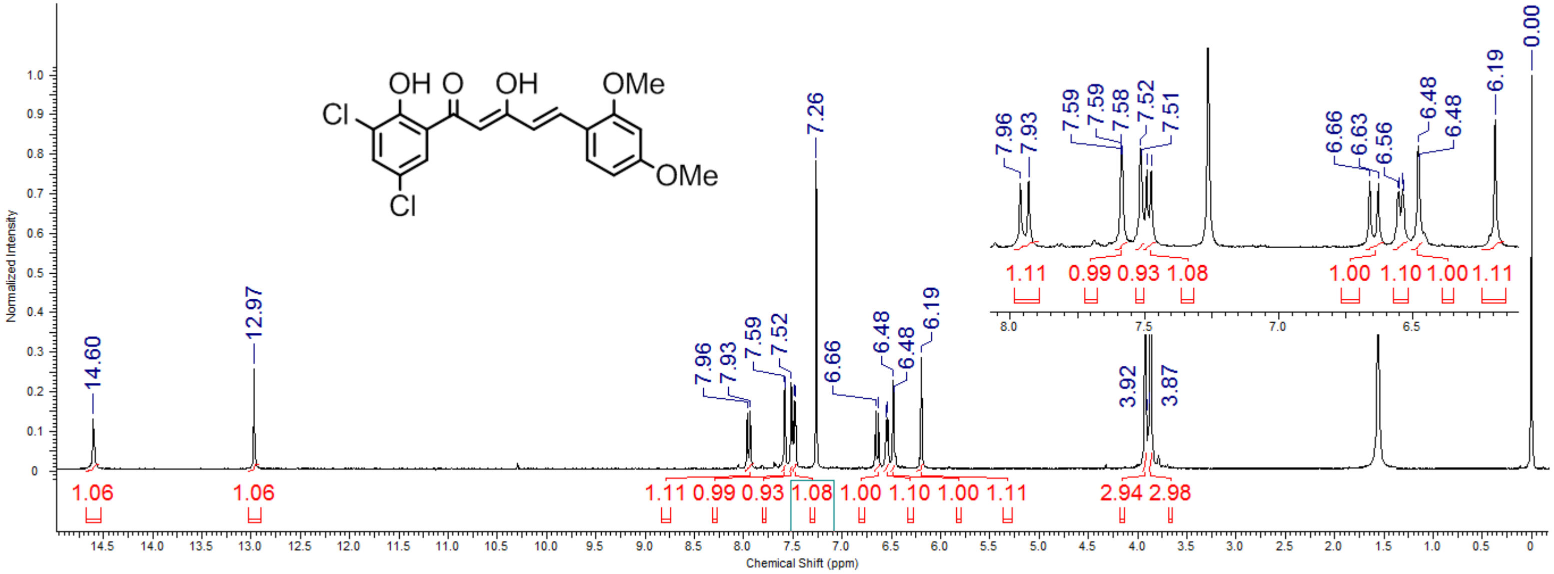

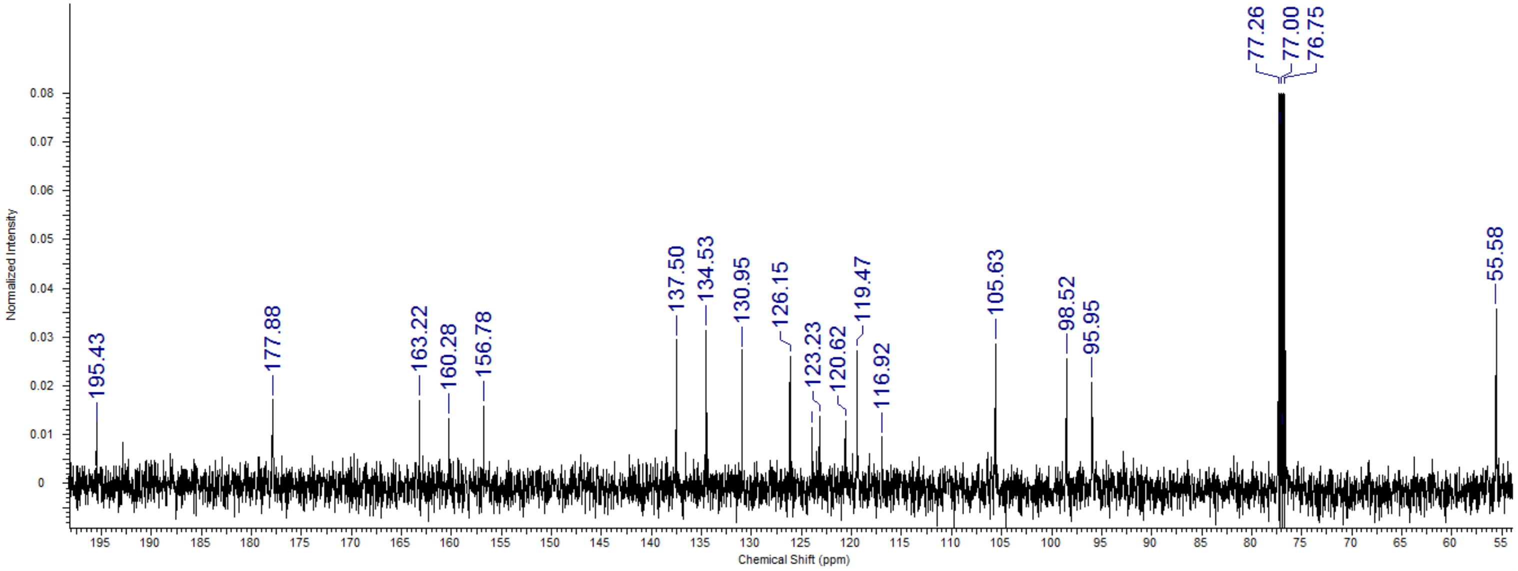

Figure S78. NMR spectra of compound **78**.

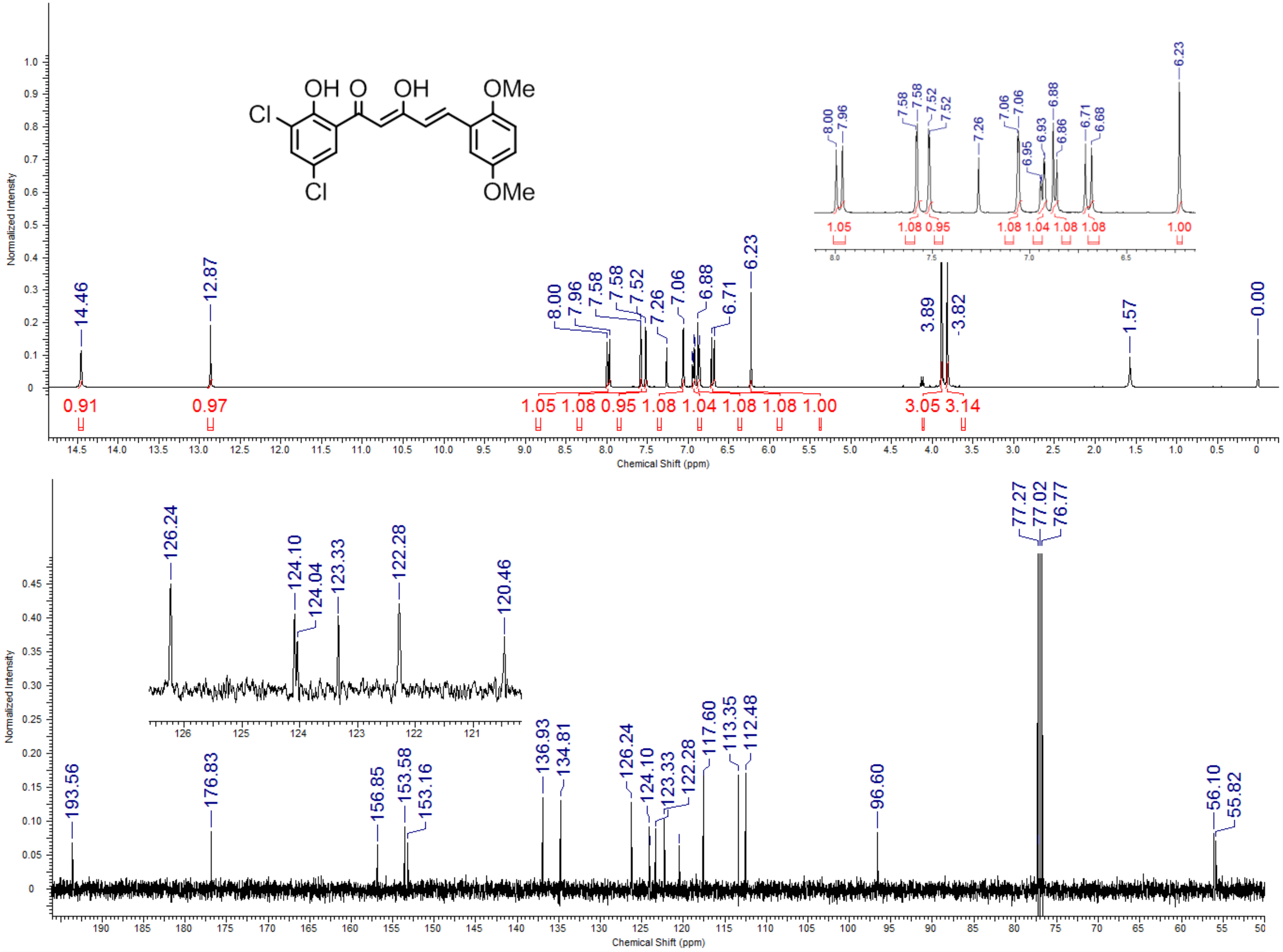

Figure S79. NMR spectra of compound **79**.

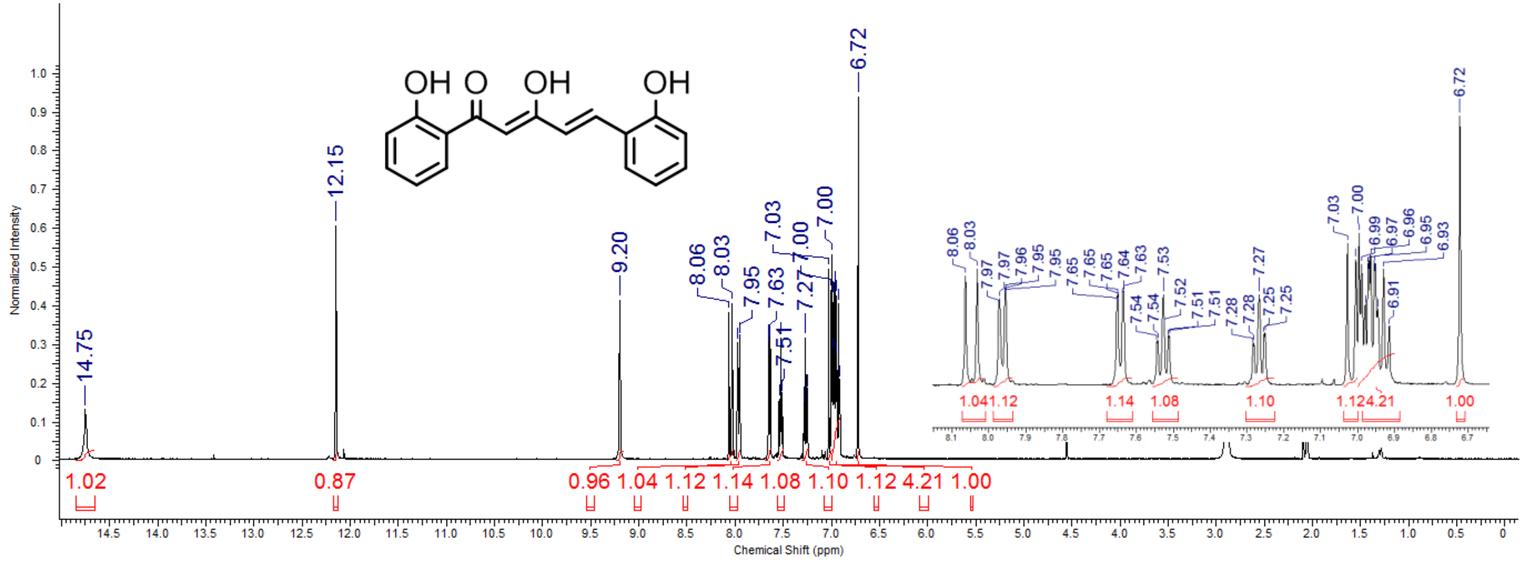

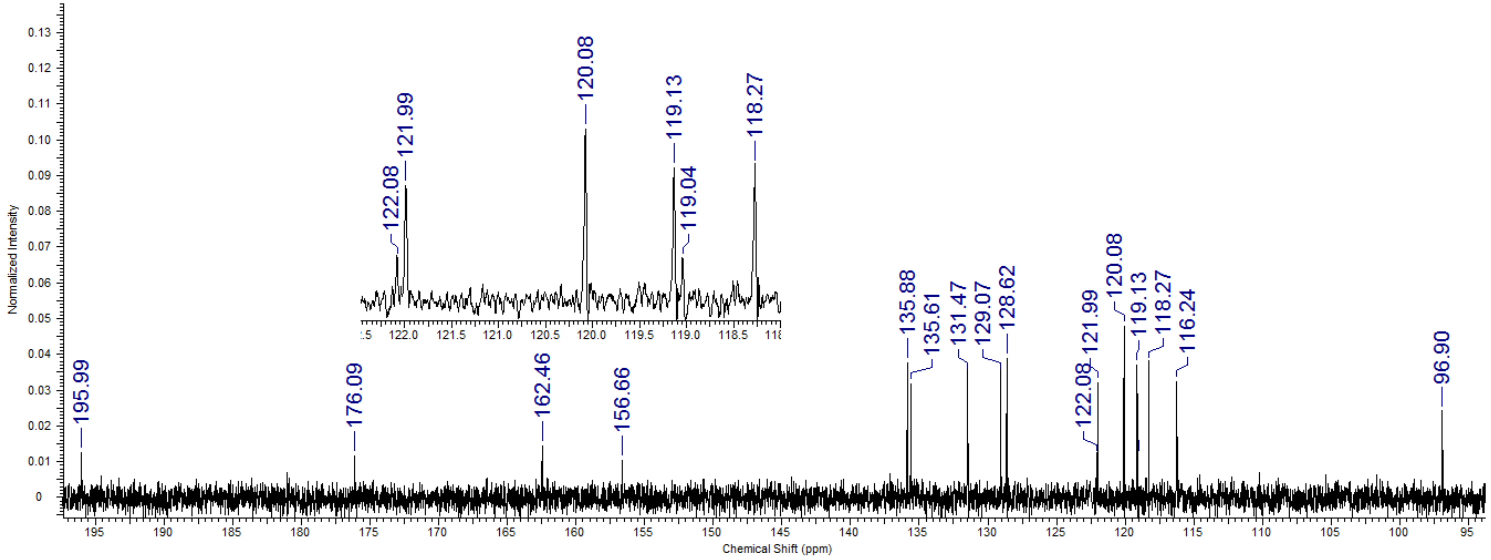

Figure S80. NMR spectra of compound **80**.

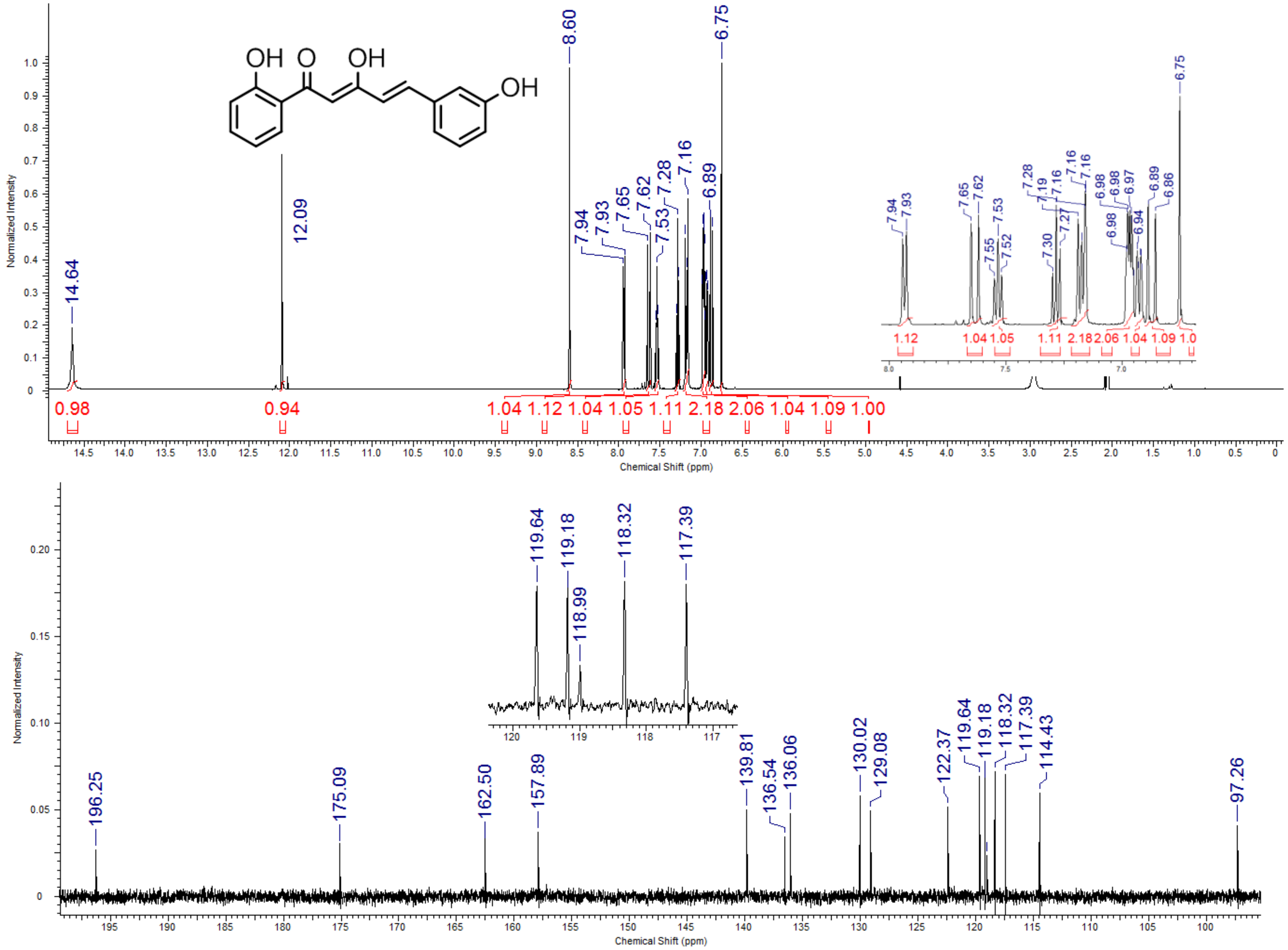

Figure S81. NMR spectra of compound **81**.

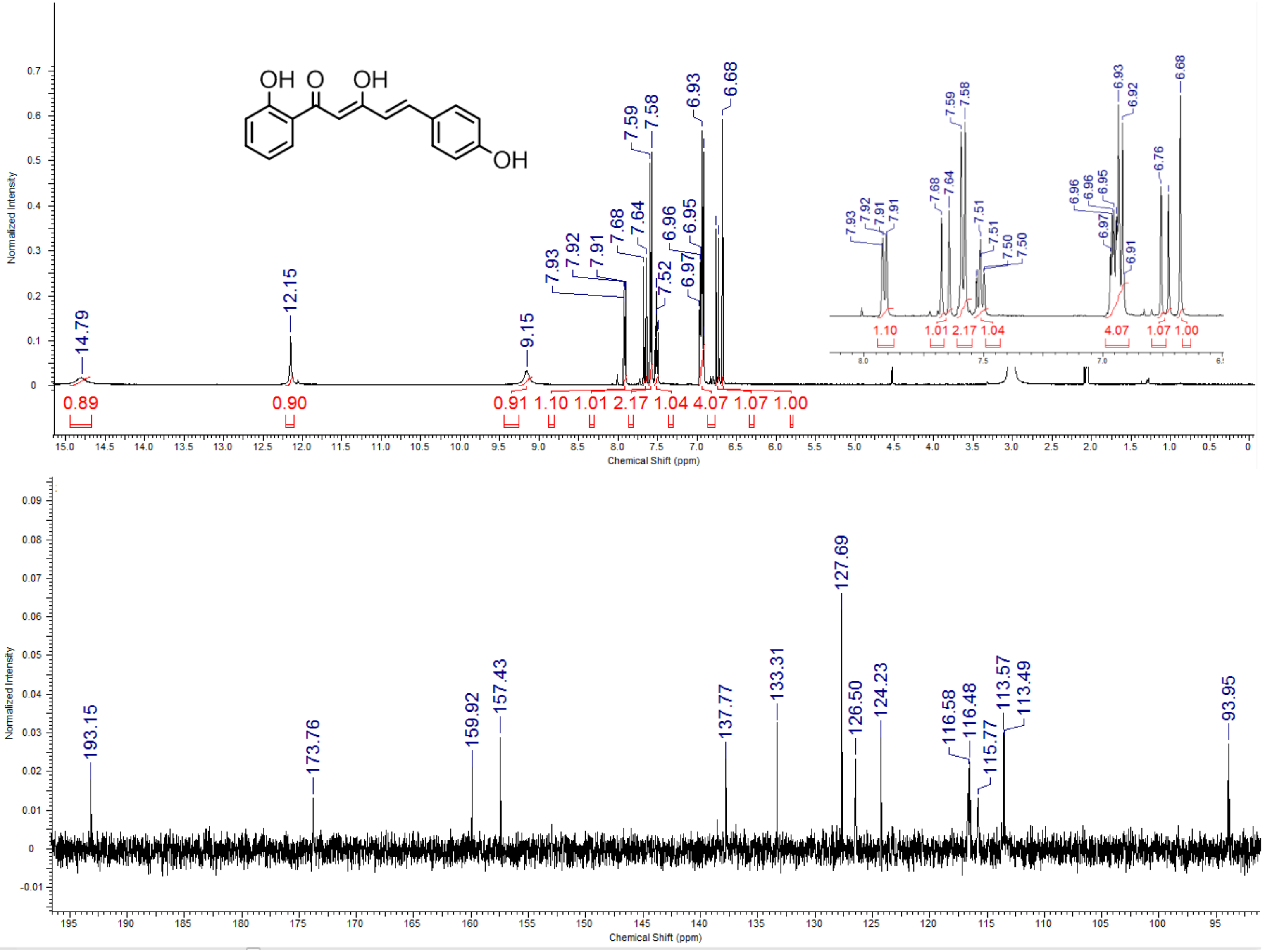

Figure S82. NMR spectra of compound **82**.

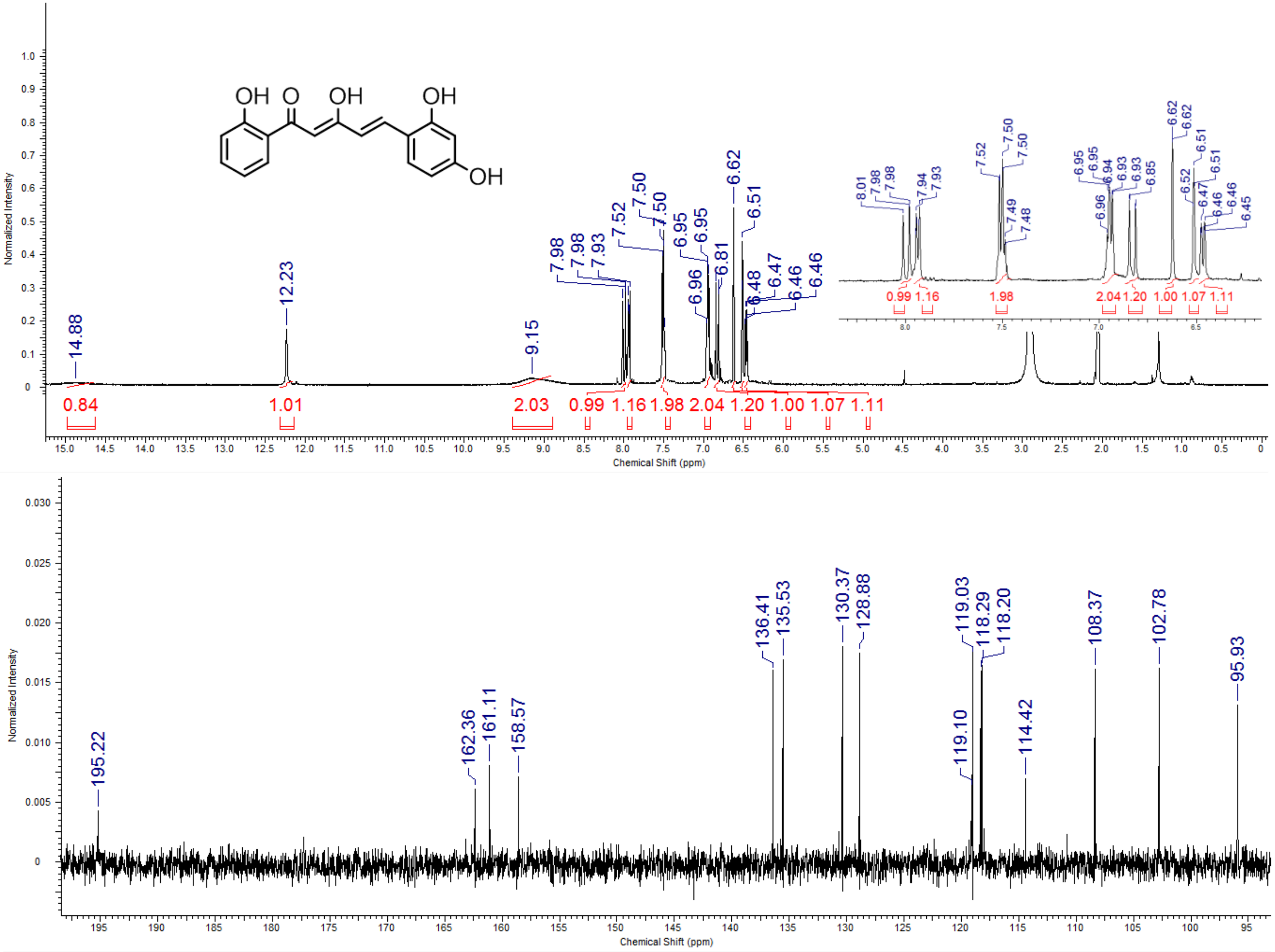

Figure S83. NMR spectra of compound **83**.

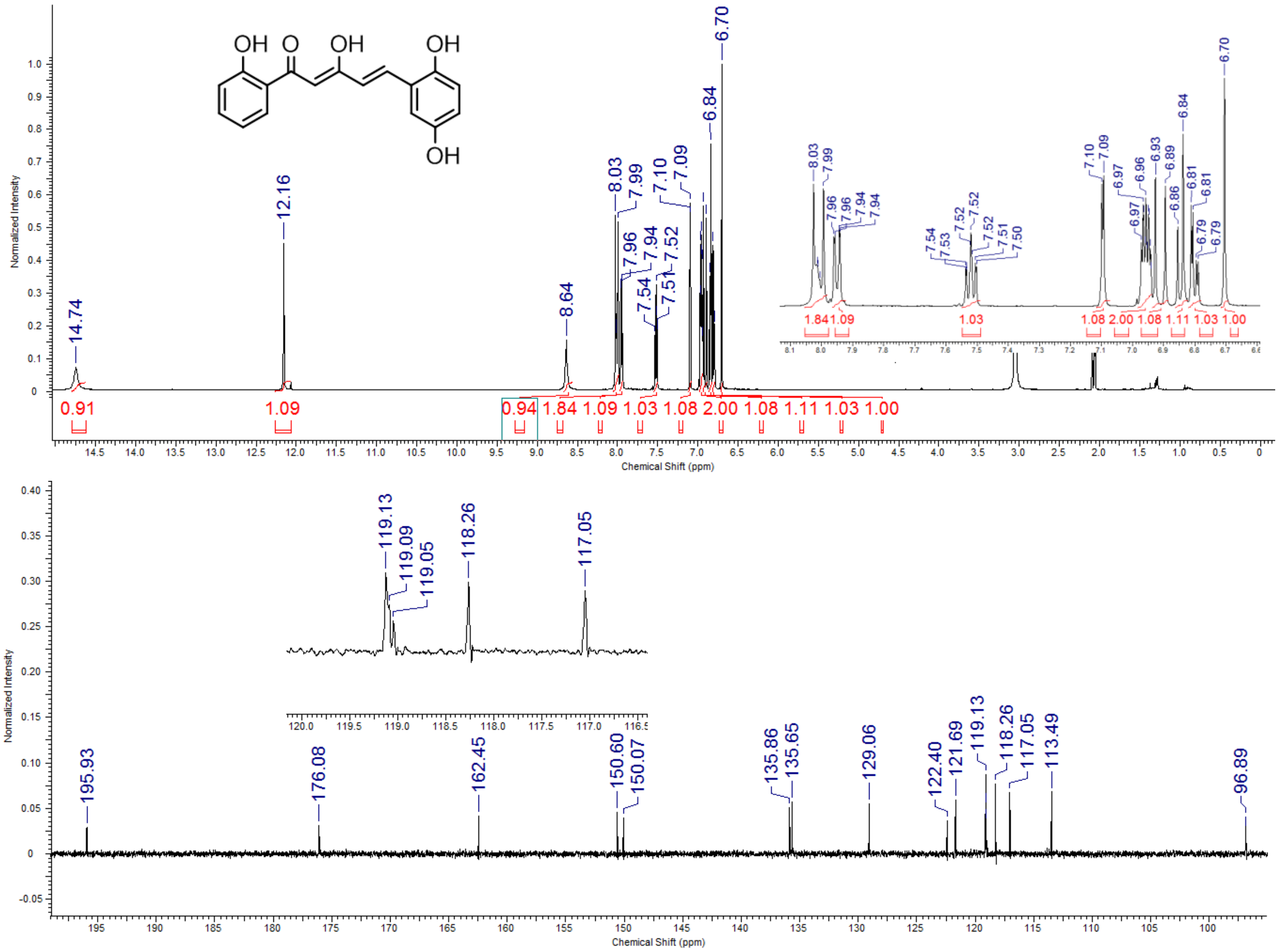

Figure S84. NMR spectra of compound **84**.

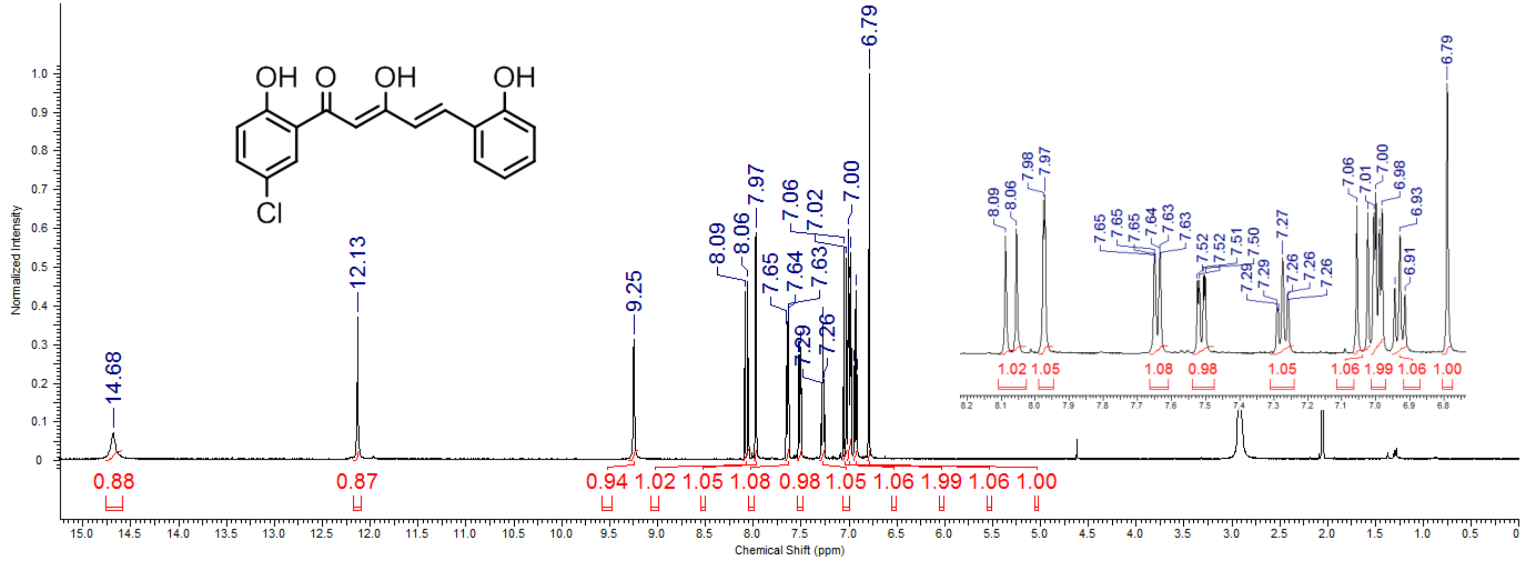

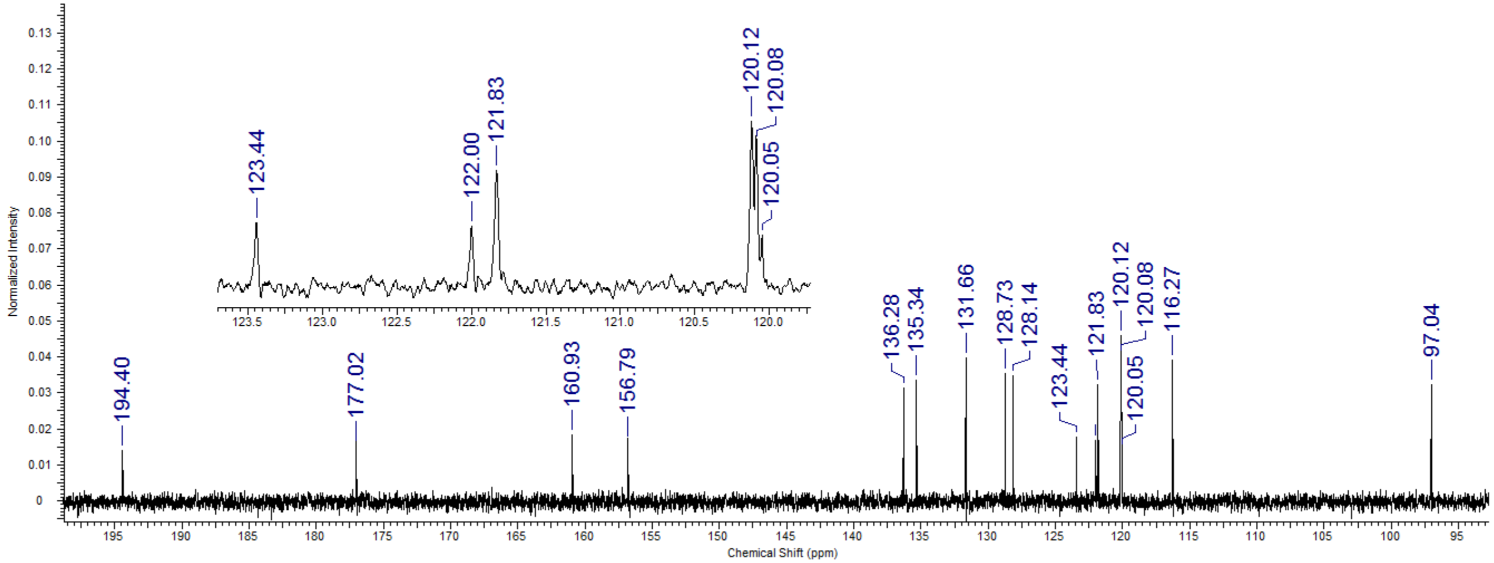

Figure S85. NMR spectra of compound **85**.

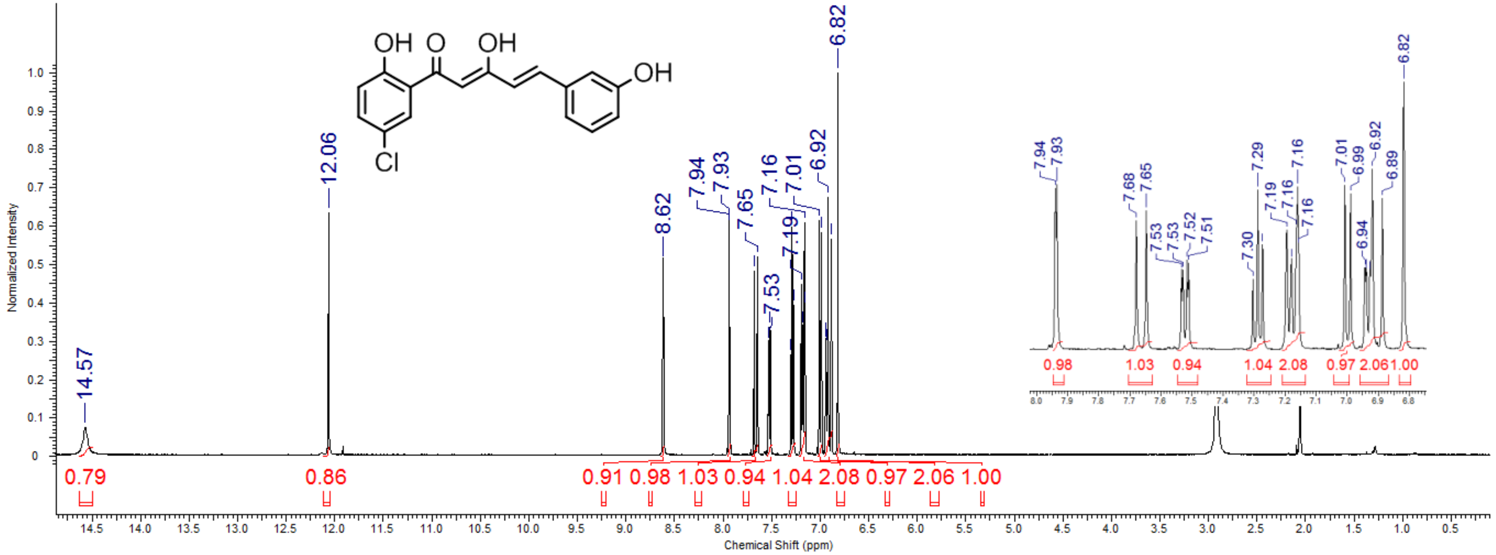

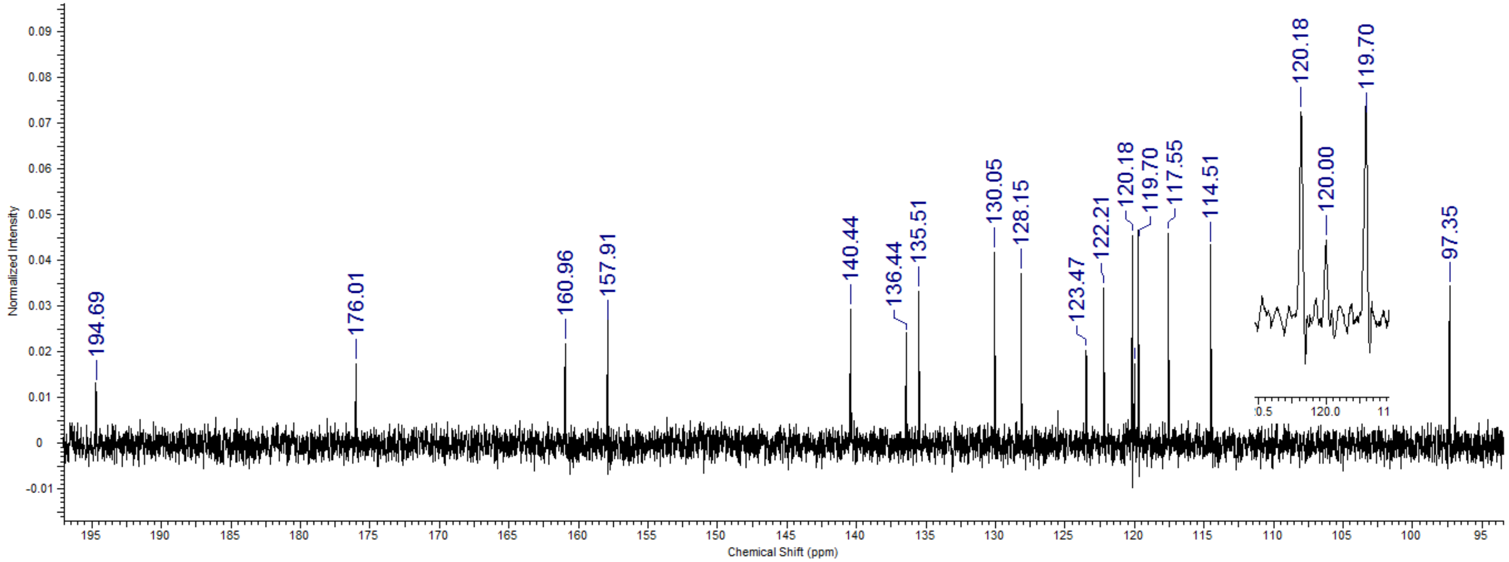

Figure S86. NMR spectra of compound **86**.

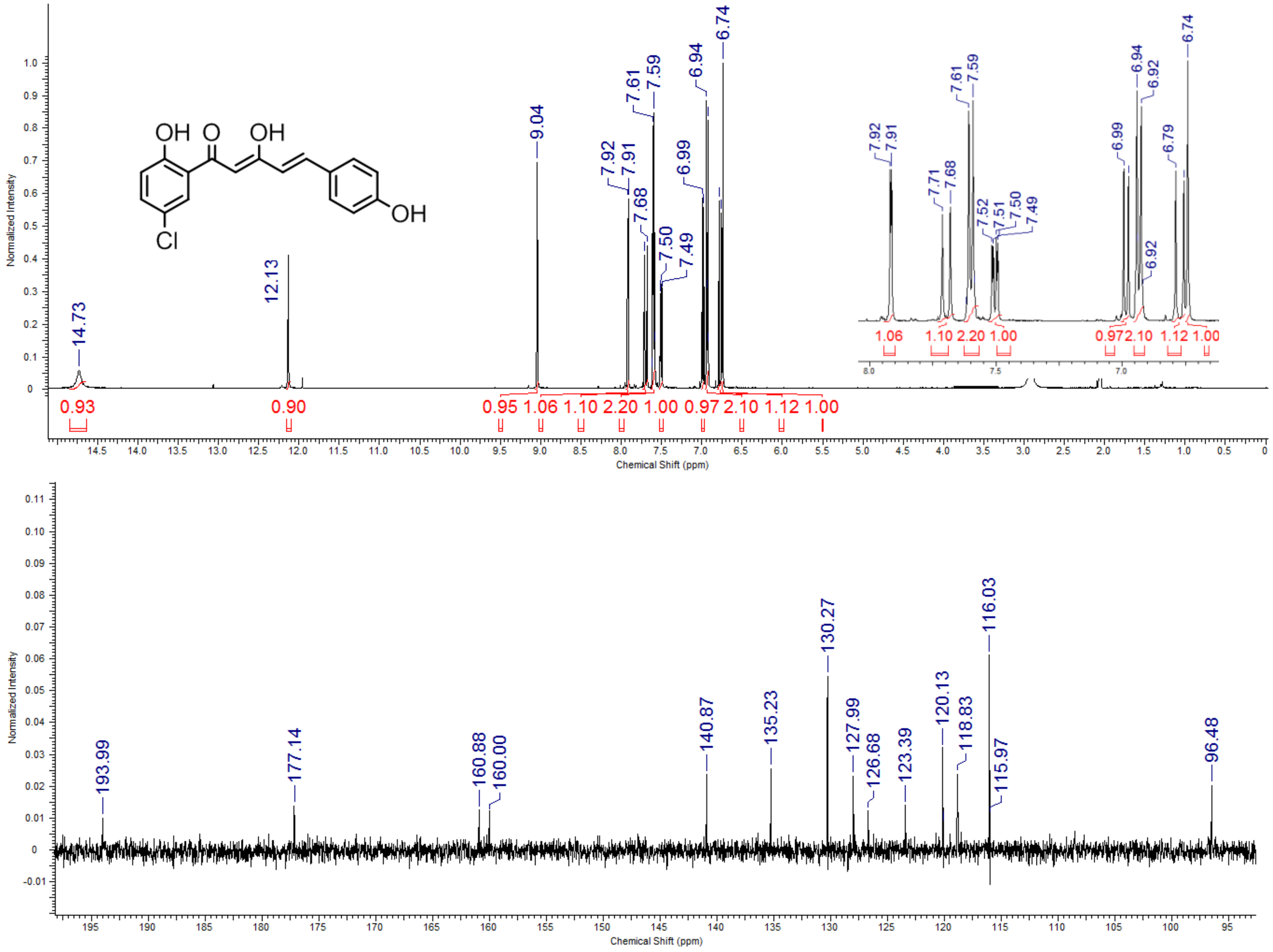

Figure S87. NMR spectra of compound **87**.

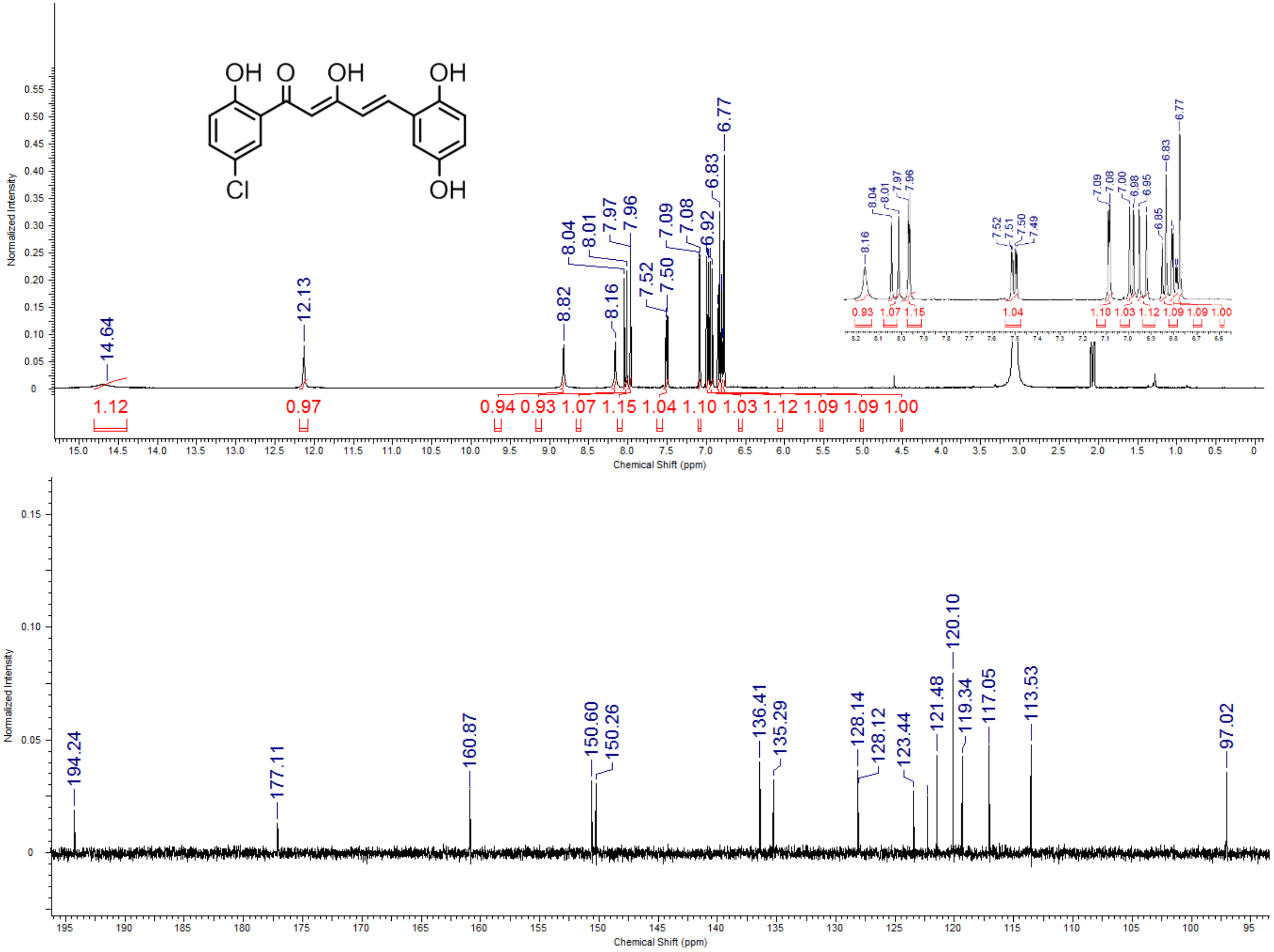

Figure S88. NMR spectra of compound **88**.

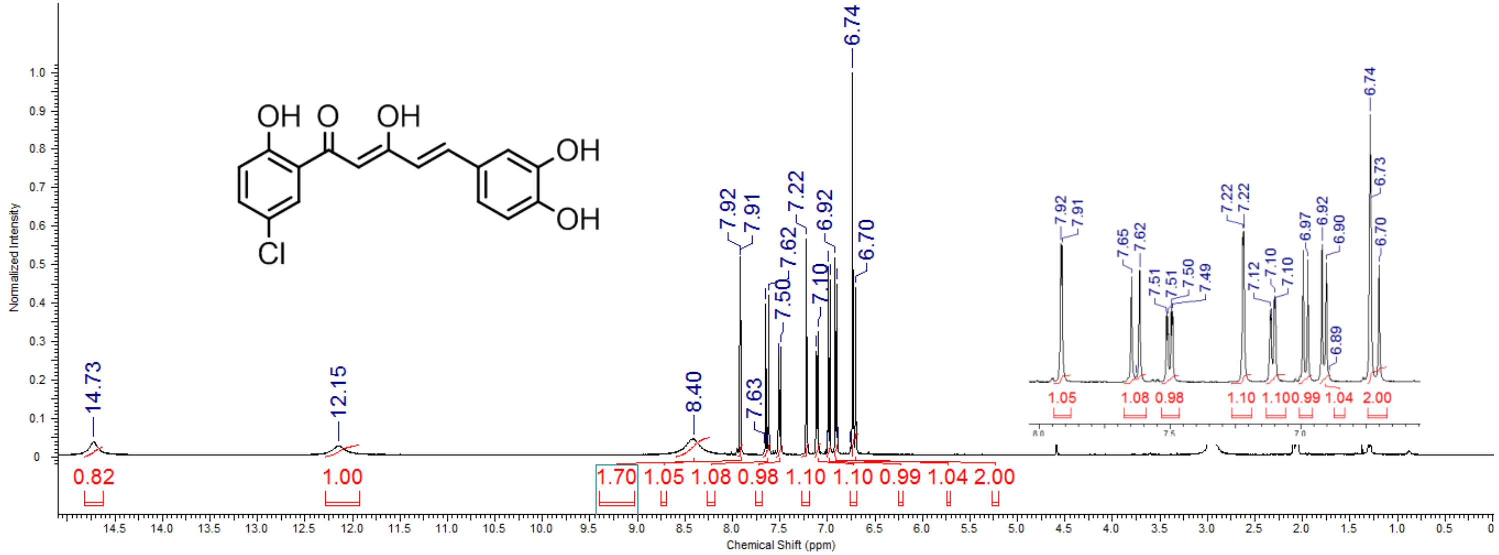

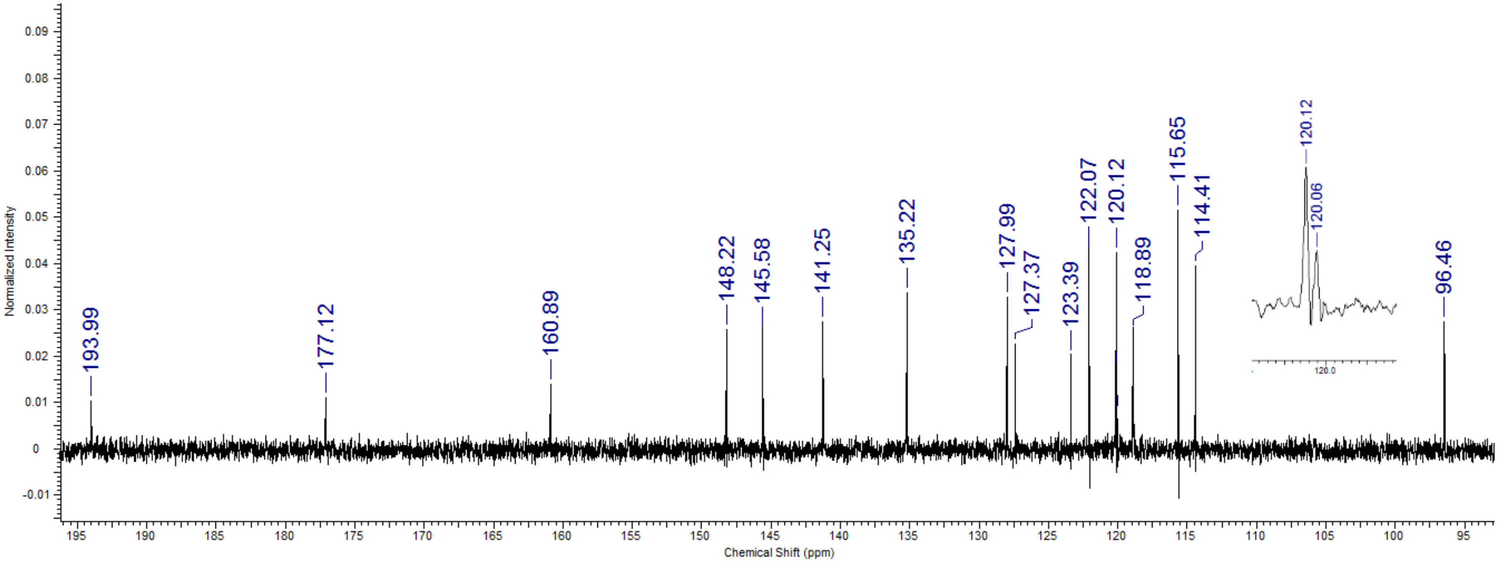

Figure S89. NMR spectra of compound **89**.

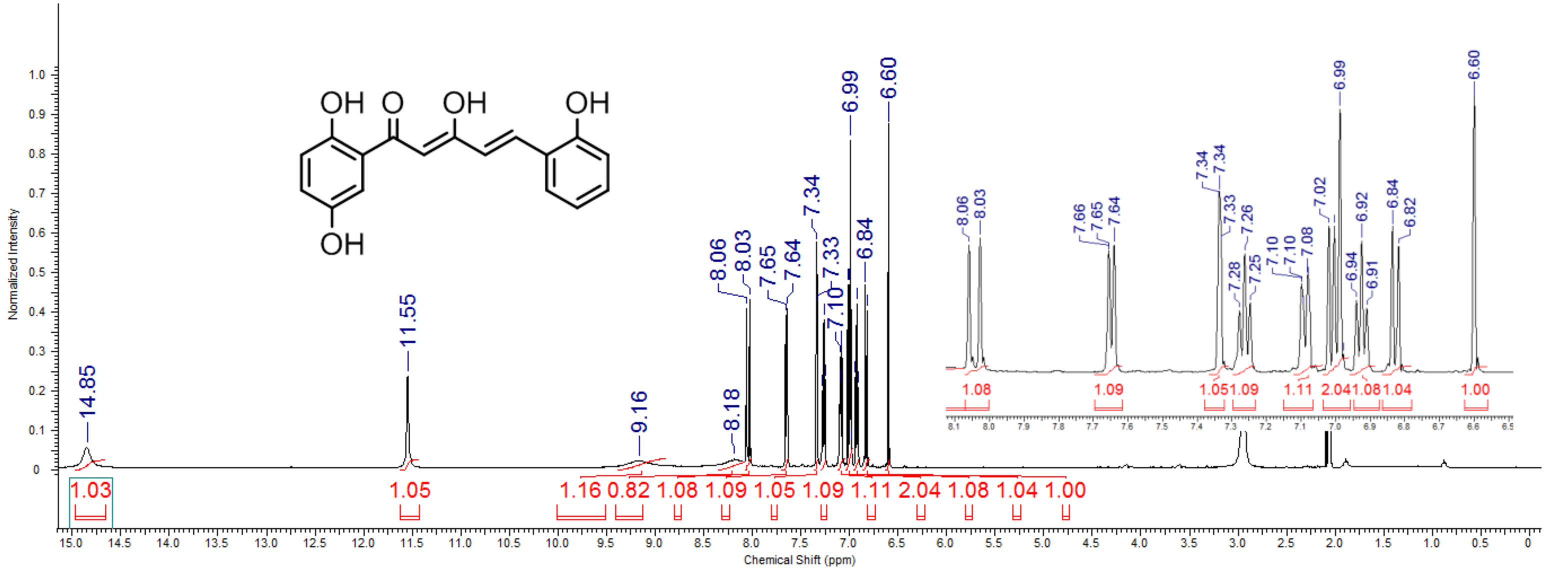

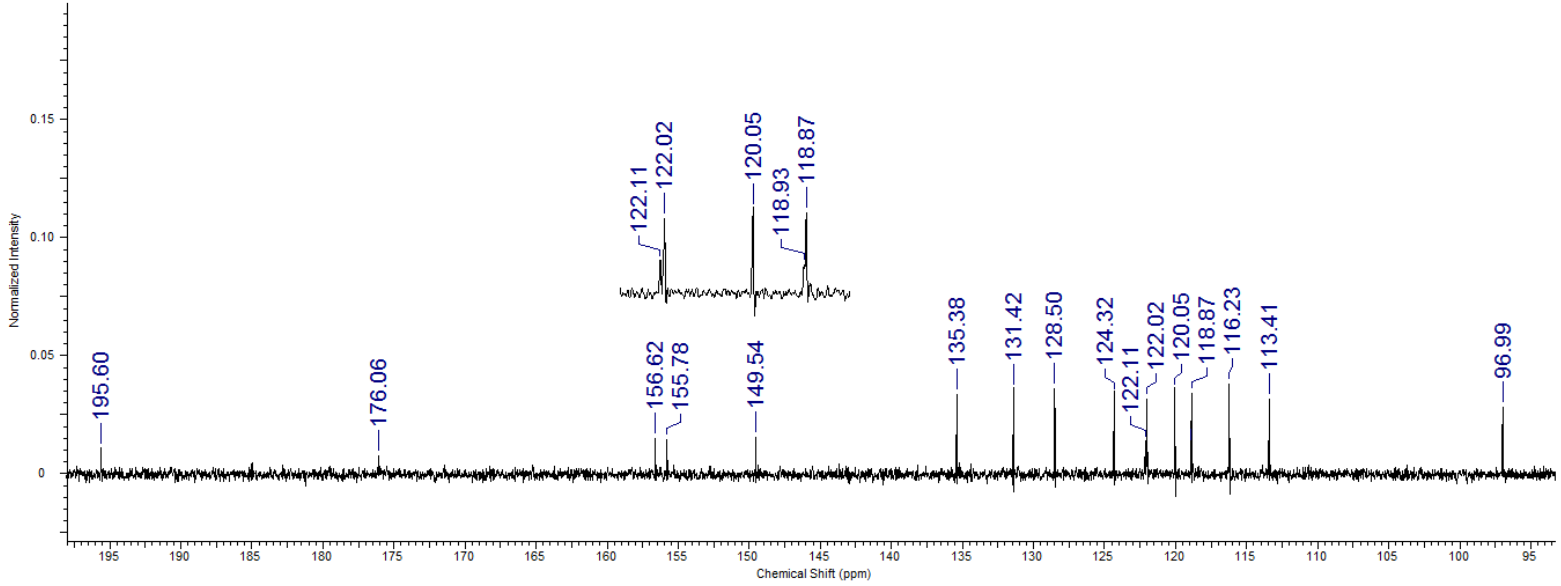

Figure S90. NMR spectra of compound **90**.

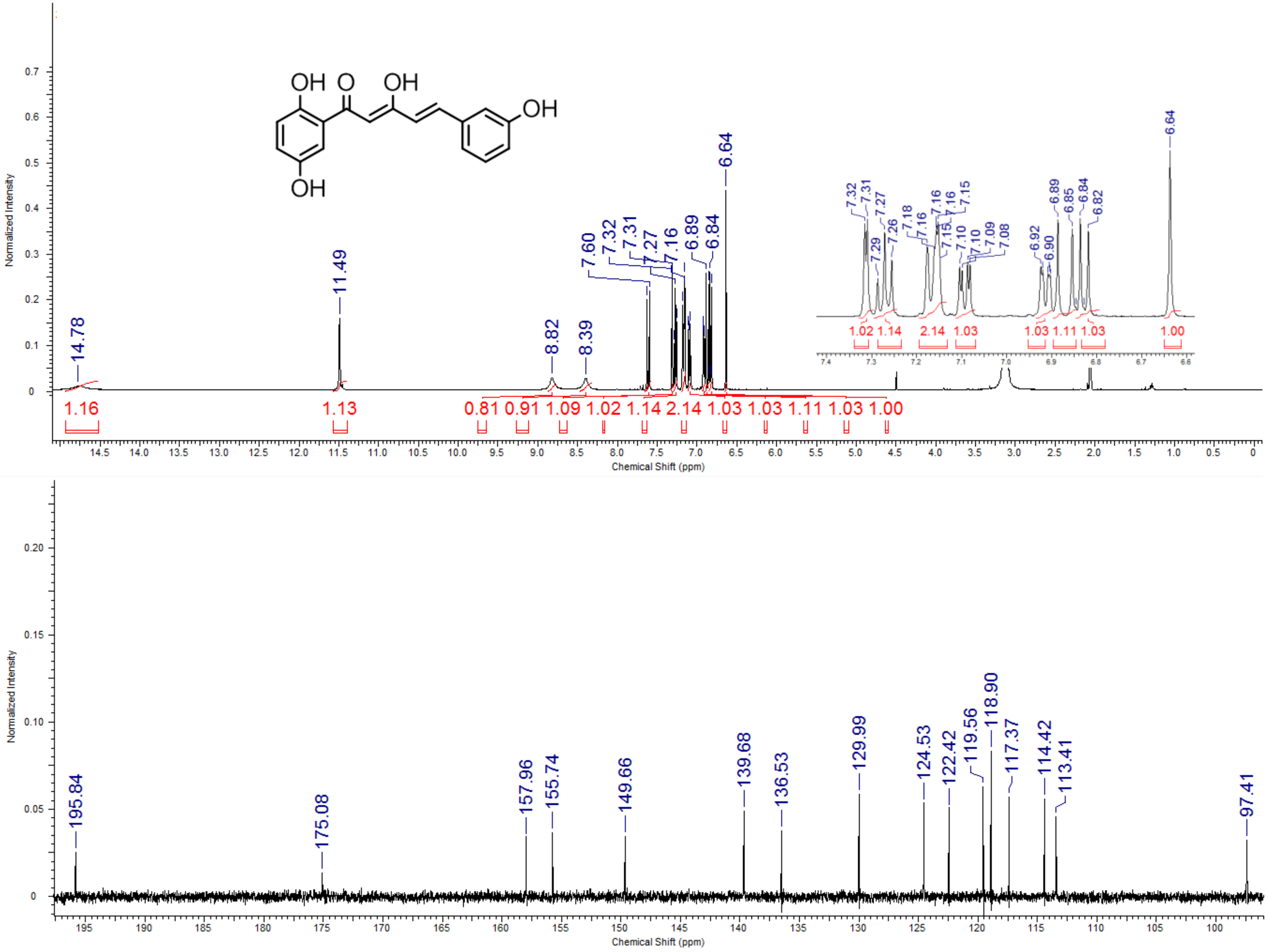

Figure S91. NMR spectra of compound **91**.

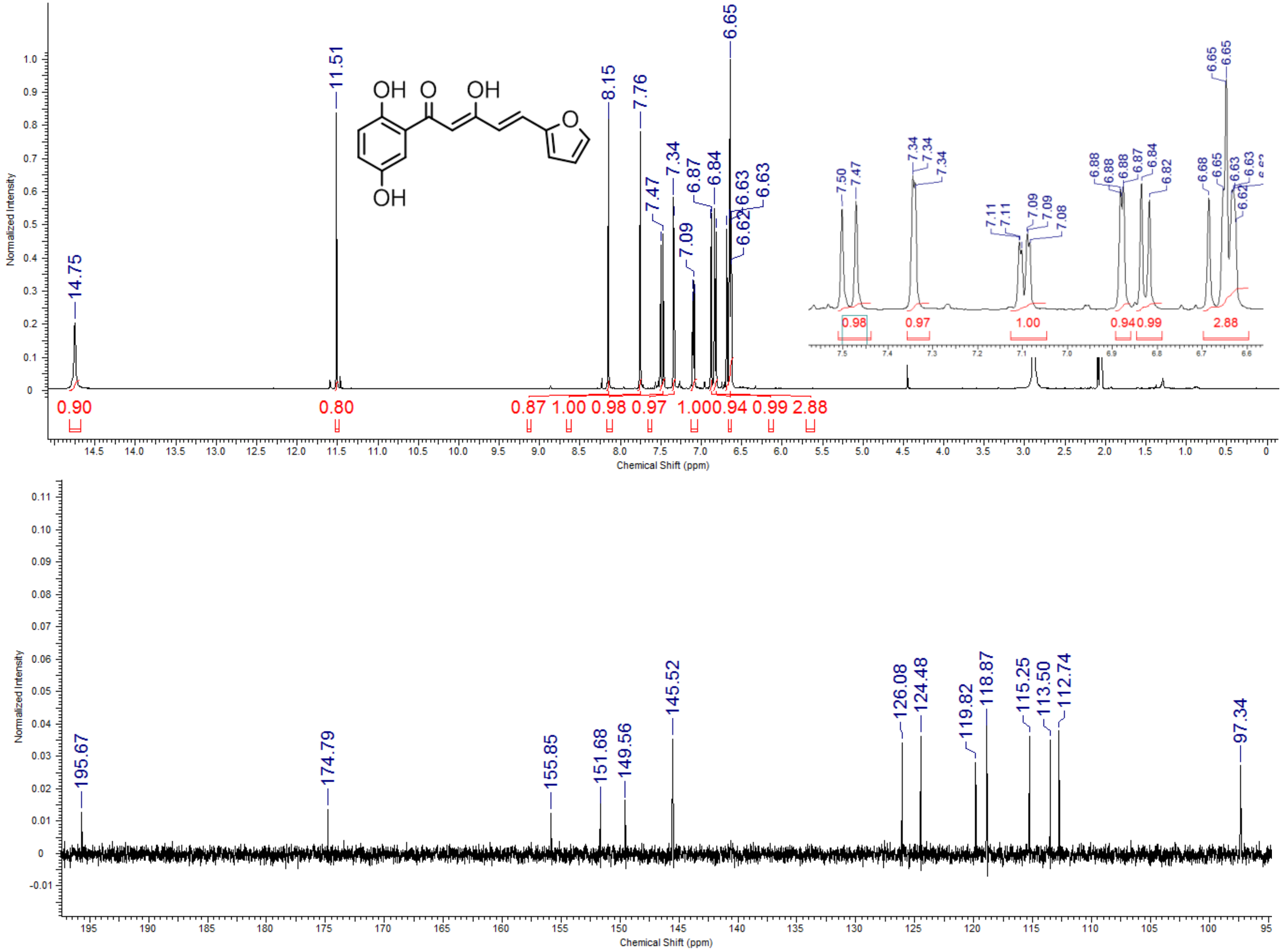

Figure S92. NMR spectra of compound **92**.

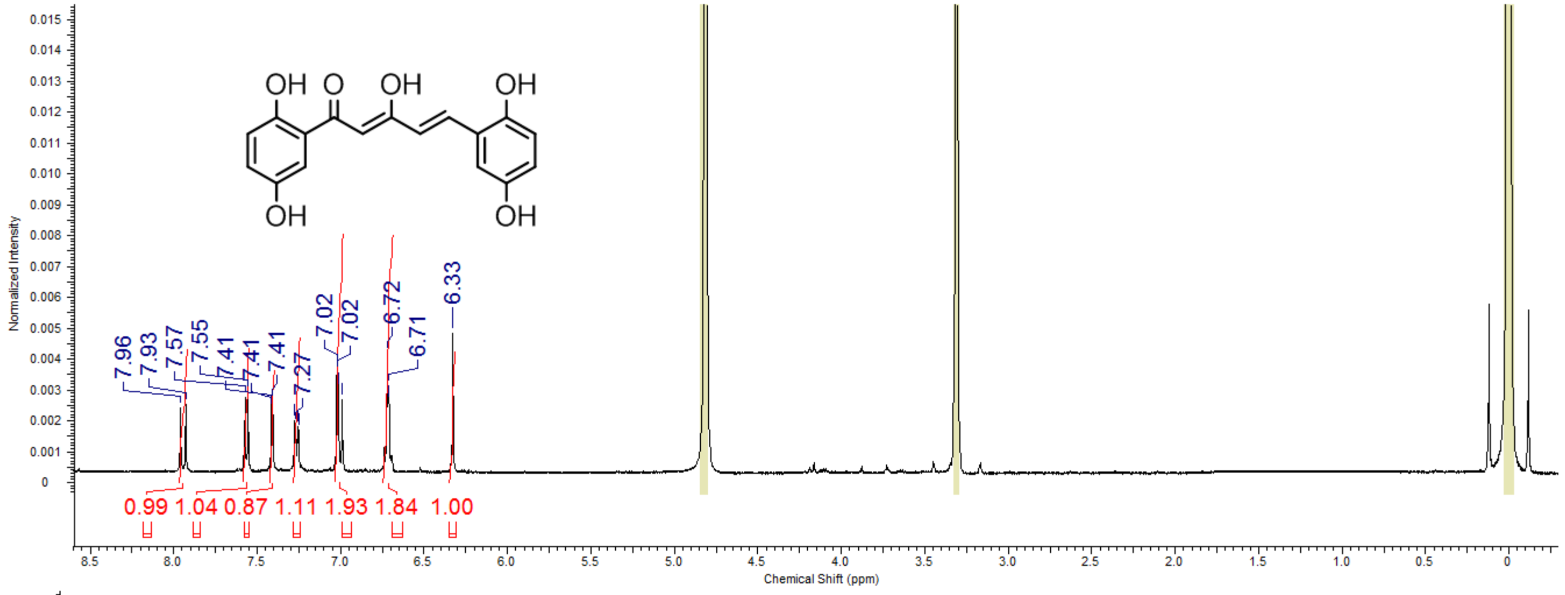

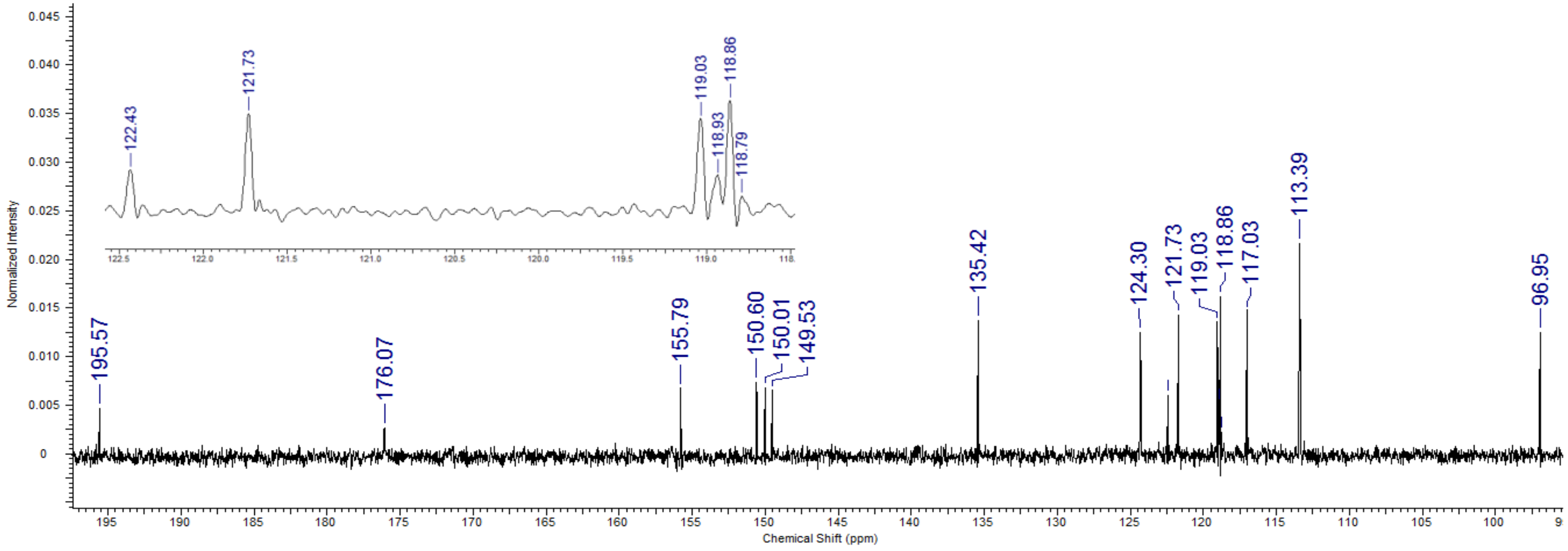

Figure S93. NMR spectra of compound **93**.

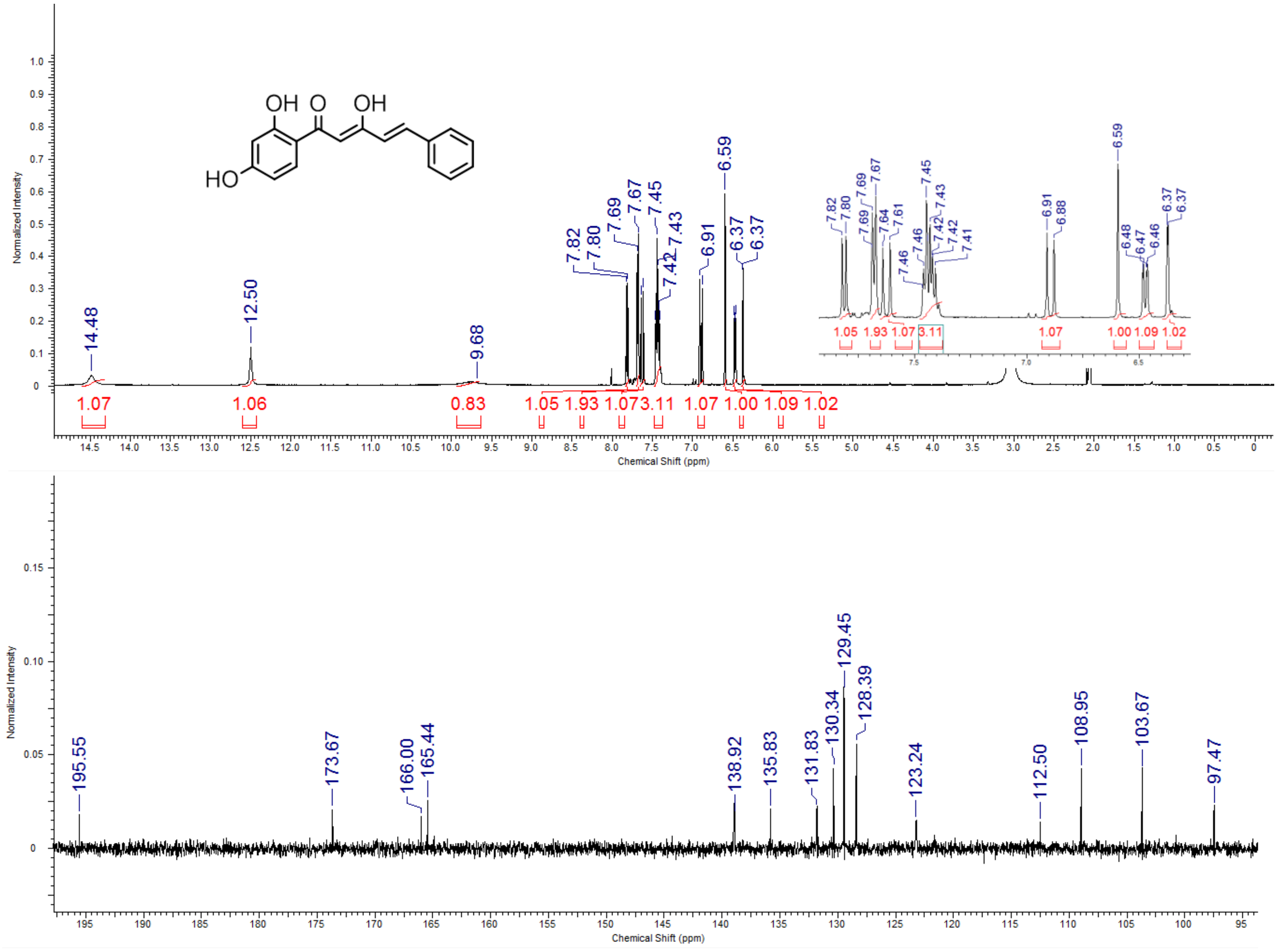

Figure S94. NMR spectra of compound **94**.

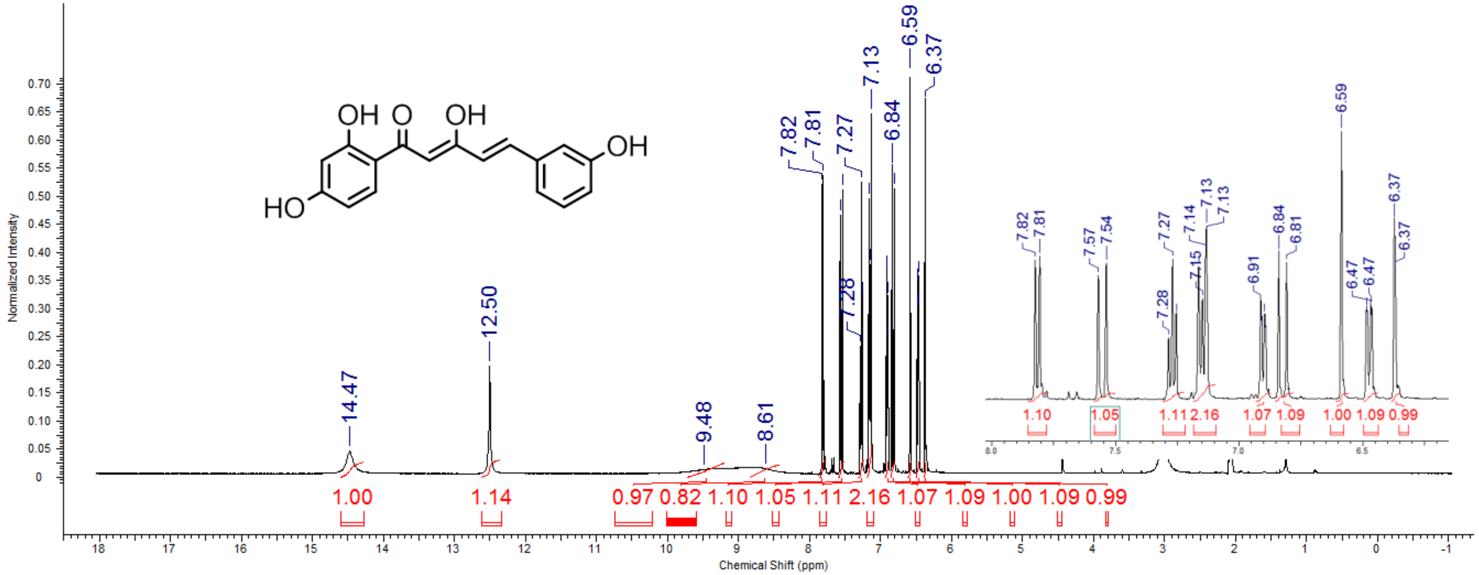

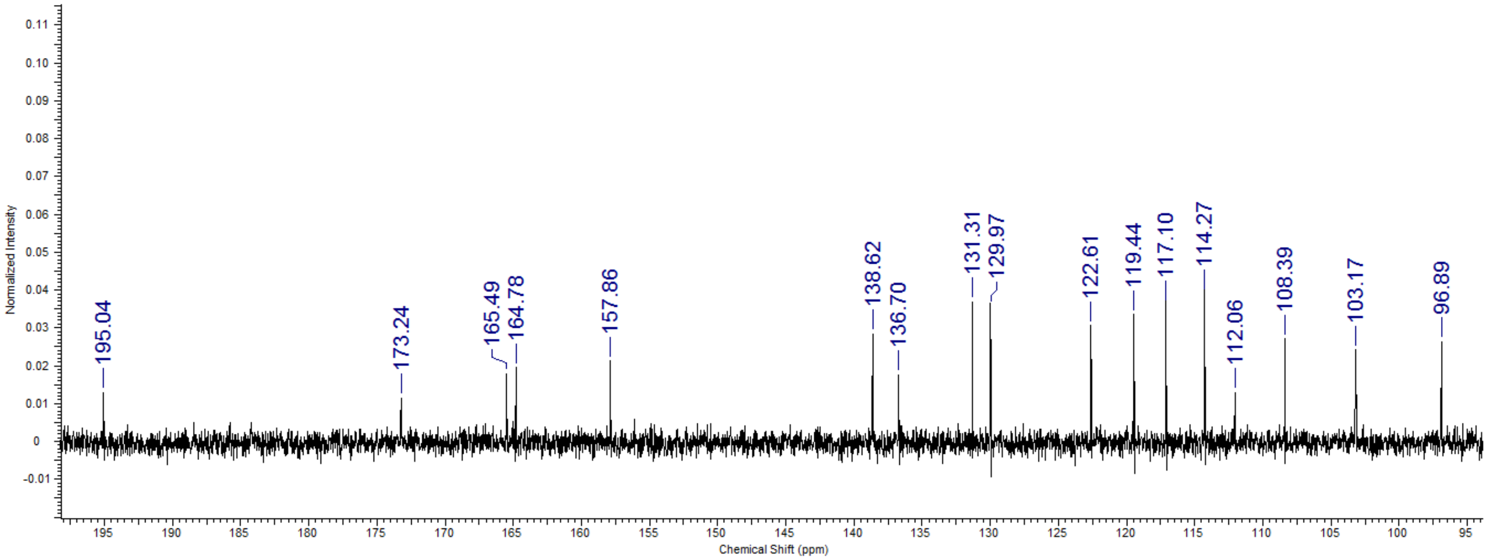

Figure S95. NMR spectra of compound **95**.

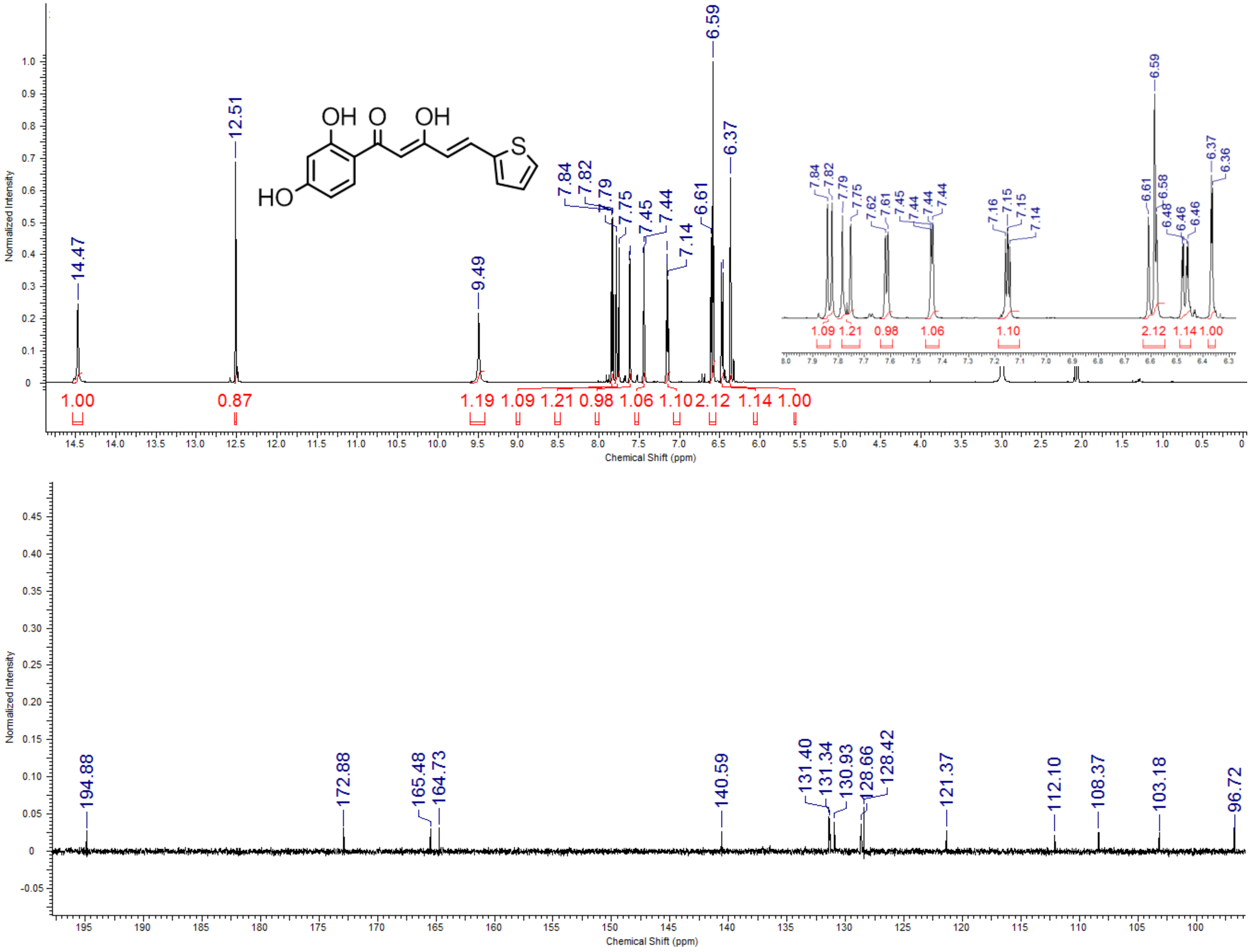

Figure S96. NMR spectra of compound **96**.

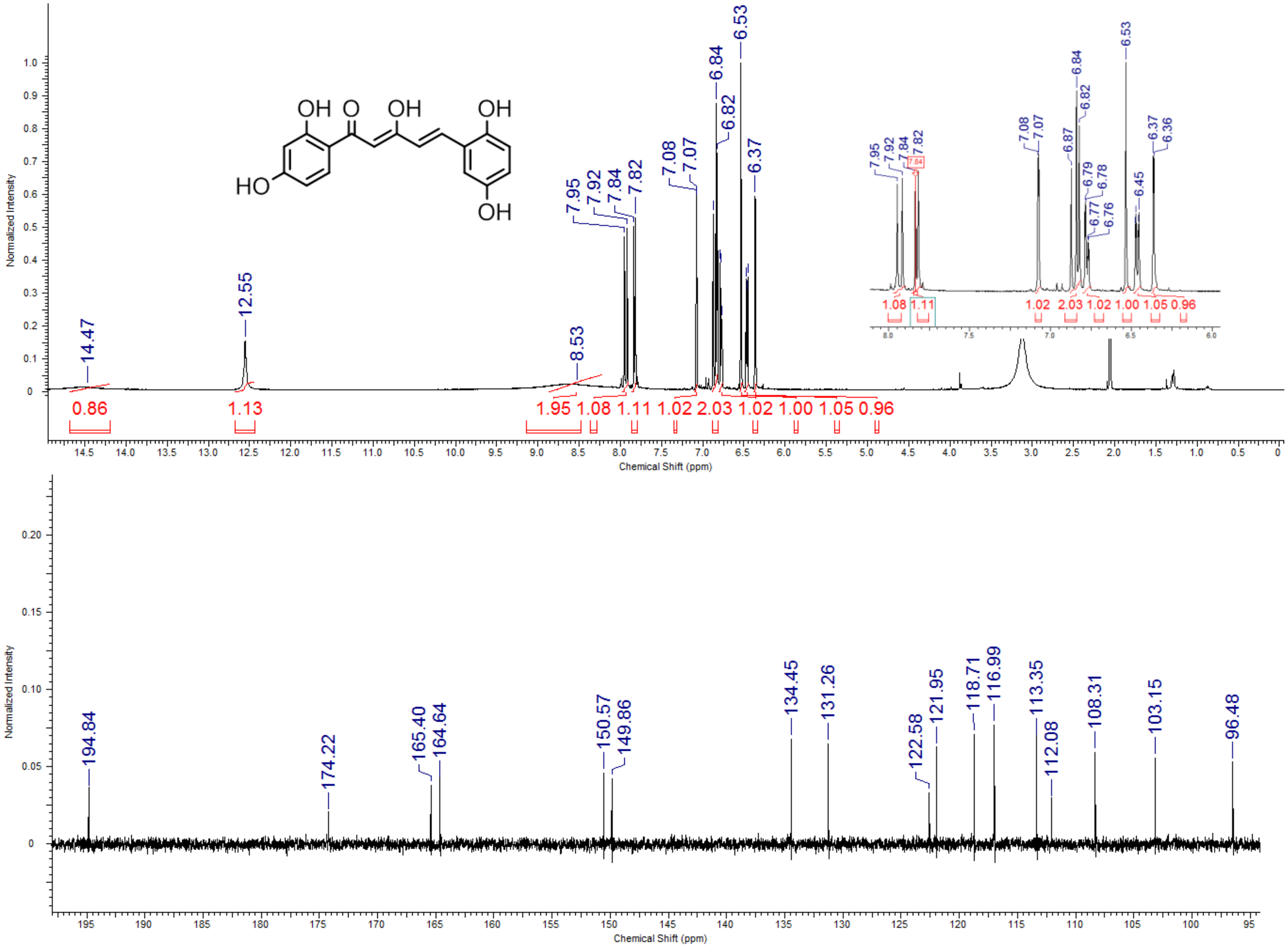

Figure S97. NMR spectra of compound **97**.

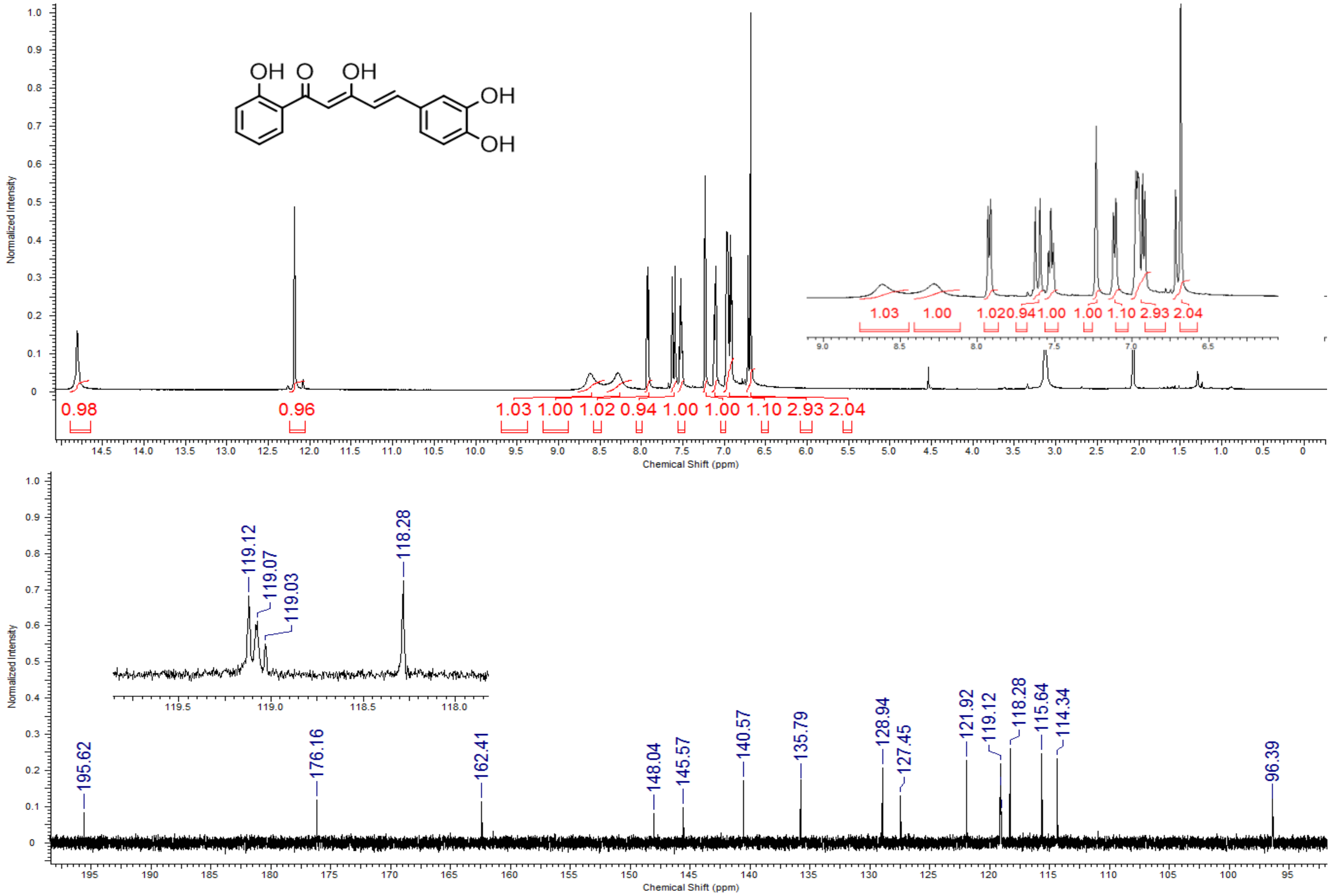

Supplement: Supplementary File 1 [file molecules-19-16058-s001.pdf]
